# High-content analysis of microRNAs involved in the phenotype regulation of vascular smooth muscle cells

**DOI:** 10.1038/s41598-022-07280-7

**Published:** 2022-03-03

**Authors:** Jian Zhang, Vytaute Starkuviene, Holger Erfle, Zhaohui Wang, Manuel Gunkel, Ziwei Zeng, Carsten Sticht, Kejia Kan, Nuh Rahbari, Michael Keese

**Affiliations:** 1 Chirurgische Klinik and European Center of Angioscience (ECAS), Medical Faculty Mannheim, Heidelberg University, Mannheim, Germany; 2 BioQuant, Heidelberg University, Heidelberg, Germany; 3 Institute of Biosciences, Vilnius University Life Sciences Center, Vilnius, Lithuania; 4 Medical Research Center, Medical Faculty Mannheim, Heidelberg University, Mannheim, Germany

**Keywords:** Biological techniques, Cell biology, Computational biology and bioinformatics, Drug discovery, Molecular biology

## Abstract

In response to vascular injury vascular smooth muscle cells (VSMCs) alternate between a differentiated (contractile) and a dedifferentiated (synthetic) state or phenotype. Although parts of the signaling cascade regulating the phenotypic switch have been described, the role of miRNAs is still incompletely understood. To systematically address this issue, we have established a microscopy-based quantitative assay and identified 23 miRNAs that induced contractile phenotypes when over-expressed. These were then correlated to miRNAs identified from RNA-sequencing when comparing cells in the contractile and synthetic states. Using both approaches, six miRNAs (miR-132-3p, miR-138-5p, miR-141-3p, miR-145-5p, miR-150-5p, and miR-22-3p) were filtered as candidates that induce the phenotypic switch from synthetic to contractile. To identify potentially common regulatory mechanisms of these six miRNAs, their predicted targets were compared with five miRNAs sharing ZBTB20, ZNF704, and EIF4EBP2 as common potential targets and four miRNAs sharing 16 common potential targets. The interaction network consisting of these 19 targets and additional 18 hub targets were created to facilitate validation of miRNA-mRNA interactions by suggesting the most plausible pairs. Furthermore, the information on drug candidates was integrated into the network to predict novel combinatorial therapies that encompass the complexity of miRNAs-mediated regulation. This is the first study that combines a phenotypic screening approach with RNA sequencing and bioinformatics to systematically identify miRNA-mediated pathways and to detect potential drug candidates to positively influence the phenotypic switch of VSMCs.

## Introduction

Cardiovascular disease is a leading cause of death worldwide and accounts for more than 17.3 million deaths per year, with an estimated increase in incidence to 23.6 million by 2030^1^. Peripheral artery disease, atherosclerosis after coronary artery disease and stroke are the most common manifestations^2^. Vascular smooth muscle cells (VSMCs) frequently account for related pathophysiological processes within the blood vessel wall. Unlike many other mature cell types in the adult body, VSMCs do not terminally differentiate but retain a remarkable plasticity^3^. Predominantly, they are found in two principal phenotypes: contractile and synthetic^4^. Under normal physiological conditions the cells maintain the muscular tone of blood vessels and acquire the quiescent/contractile phenotype. In response to vascular injuries or alterations in the local environment, contractile VSMCs can re-enter the cell cycle, undergo a phenotypic switch to the synthetic phenotype and drive the progression of vascular diseases^3^. The phenotype switch facilitates plaque formation, which is the prerequisite for atherosclerosis^5^. It is established by now, that inhibiting VSMCs phenotypic switching may be beneficial in advanced stages of this disease.

The cells in the quiescent/contractile phenotype show low levels of migration and proliferation. Morphologically, the contractile VSMCs display a fusiform or spindle-like shape, abundant myofilaments and a heterochromatic nucleus^6^. In contrast, the synthetic VSMCs adopt a rhomboid shape without specific filamentous cytoplasm, but with an extensive rough endoplasmic reticulum, Golgi complex, and a euchromatic nucleus^6,7^. The phenotype switch is tightly regulated on a molecular level. Oxidative stress, autophagy, the expression levels and repertoire of matrix metalloproteinases and integrins have been shown to play important roles in the phenotypic switch and, consequently, vascular remodeling^8^. For instance, the serum response factor acts directly or indirectly on most VSMC contractile genes^9^. Furthermore, a recent study shows that fibroblast growth factor 12 (FGF12) reduces cell proliferation through the p53 pathway and up-regulates key factors related to the differentiation of the VSMC lineage, such as myocardin and serum response factors^10^. At any rate, when acquiring one of these phenotypes, VSMCs alter the activation status of various pathways and, as a result, numerous proteins change their expression levels. For this reason, proteomics technology, in particular differential proteomics, was instrumental for identifying the molecular factors putatively involved in VSMC phenotypic modulation^11^.

Phenotypic switch is also regulated post-transcriptionally by microRNAs (miRNAs), that are endogenous short non-coding RNAs containing 21 ~ 23 nucleotides^12^. Several miRNAs are already proved to play essential roles in the modulation of VSMCs’ function and phenotypic switch in vitro and/or in animal models^13–17^. Yamaguchi et al.^14^ stated that miR-145 could induce a morphological change in VSMCs from a rhomboid- to a spindle-like shape in human ES-pre-SMCs. Furthermore, overexpression of miR-145 promoted differentiation and inhibited the proliferation of cultured VSMCs^13,18^. Platelet-Derived Growth Factor (PDGF), a potent stimulator of VSMCs’ migration, can downregulate the expression of miR-145, inducing podosome formation. This appears to be mediated through the activity of Src and p53^19^. miR-145-deficient VSMCs, on the other hand, failed to demonstrate a contractile phenotype in response to vasopressin stimuli^20^, indicating a context-dependent role of miRNAs. Huang et al.^15^ showed that miR-22 mimics significantly reduced proliferation and migration of (VSMCs) via targeting of methyl CpG-binding protein 2 (MECP2)^21^. In another example, Afzal et al.^16^ revealed that overexpression of miR-214 in VSMCs significantly decreased proliferation and migration via downregulation of NCK-associated protein 1 (NCKAP1) expression, which in turn diminished lamellipodia formation^22^. Pan et al.^17^ found that miR-663a was significantly downregulated in VSMCs after PDGF treatment, whereas its expression markedly increased during cell differentiation. Furthermore, it was demonstrated that overexpression of miR-663a increased expression of VSMC differentiation marker genes such as SM22α, α-SMA, calponin, and SM-MHC, which potently inhibit PDGF-induced proliferation and migration. Recent reports also suggest that miRNA application may be a potentially effective therapy. For example, Yang et al. applied in a wire-injury mouse model locally of AgomiR-22 or miR-22 inhibitor to demonstrate the modulation of the switch phenotype^23^.

Despite the above-mentioned examples, a comprehensive list of miRNAs known to induce the phenotypic switch is still missing^24^. In order to efficiently and systematically identify such molecules, large-scale methods were recently successfully harnessed. For instance, expression profiling of miRNAs^25^ and circular RNAs^26^ or miRNA sequencing^27^ discovered a number of yet unknown regulators. High-content microscopy-based screening is clearly the method of choice as it directly provides information about the cell morphology. Previously, RNAi screening of protein-coding genes^28^ was performed and, by quantifying the changes in proliferation and migration of human aortic vascular smooth muscle cells (HAoVSMCs), identified 23 genes involved in the phenotypic switch^28^. In this study, we have established a high-content analysis platform to identify and quantify contractile and synthetic phenotypes in cell populations. Our work can be easily upscaled to genome-level studies and opens the possibility for deeper understanding of the functions of miRNAs in the regulation of VSMC’s differentiation and phenotypic switch, providing new insights into the mechanisms of vascular development, function, and dysfunction.

## Materials and methods

### Cell culture

The HAoVSMCs were cultured in Smooth Muscle Cell Growth Medium-2 (SmGM-2) supplemented with 5% fetal calf serum (FCS), 0.1% epidermal growth factor (EGF), 0.1% basic fibroblast growth factor (bFGF) and 0.1% Insulin (all reagents and cells were purchased from Promocell, Germany). Cells were incubated in a humidified incubator at 37 °C with 5% CO^2^. The cells were used between passage 4 and 9 in accordance with our institutional guidelines for research on human tissues and cells. A mycoplasma test was performed regularly (at least once a month) and the cells were checked daily under a microscope. The freshly prepared cryopreservation medium contained 10% (v/v) dimethyl sulfoxide (DMSO) and 10% FCS dissolved in SmGM-2 medium. For starvation, the cells were cultured in 1% FCS for 48 h.

### Immunofluorescence

At 48 h after seeding, the HAoVSMCs were fixed with 3% paraformaldehyde (PFA) for 15 min, permeabilized with 0.1% Triton-X 100 in PBS for 10 min, and the background was blocked with 1% BSA in PBS for 1 h before staining with primary and secondary antibodies. The primary antibody for SM α-actin (1:200, Rabbit anti-alpha-actin smooth muscle, ab5694, Abcam, UK) was diluted with blocking buffer and incubated overnight at 4 °C. The secondary goat-anti-rabbit antibody (1:1000, 712,170, Life Technologies, USA) conjugated with AlexaFluor 488 was diluted with the blocking buffer and incubated for 1 h at RT. Then, TOTO (642/660, 1:1000, Invitrogen, Germany) was added to the secondary antibody solution for nuclear staining (incubation for 30 min at RT) in accordance with the manufacturer’s protocol. Phalloidin-Alexa647 (1:100 in PBS, A22287, Abcam, UK) was added for staining of actin cytoskeleton.

### Transfection of siRNAs and miRNAs

For the preparation of plates for reverse solid phase transfection in the multi-well plates, siRNA/miRNA transfection solution was dispensed on 96- and 384-well plates (BD Biosciences / Costar SIGMA, Germany) using a Microlab STAR pipetting robot (Hamilton, Reno, NV, USA)^29^. In brief, 3 µL OptiMEM (Invitrogen, Darmstadt, Germany) containing 0.4 M sucrose was transferred to each well of a 384-well plate. Then, 3.5 µL Lipofectamine 2000 (Thermo Fischer Scientific, Waltham, USA) was added. After that, 5 µL of the respective siRNA/miRNA stock solution (3 µM) followed by 7.25 µL of a 0.2% (w/v) gelatin solution containing 1 × 10–2% (v/v) fibronectin (Sigma-Aldrich, Taufkirchen, Germany) was added and mixed thoroughly. For 384-well plates, the transfection solution was diluted with H_2_O in the ratio of 1:10 and 5 µL of the diluted transfection solution was added to each well. The HAoVSMCs were seeded in the pre-coated 384-well plates at a density of 400–500 cells per well in 60 µL of culture medium per well and incubated for 72 h. Cells were stained with Hoechst 33,342 (0.2 µg/mL, Invitrogen, Germany) to label nuclei and DiIC12(3) (2 µg/mL, Invitrogen, Germany) was used to label the whole cell body. The fluorescently (Cy3) labelled on the 5ʹ end of oligos. (Thermo Fisher, Germany) and the list of miRNAs is provided in Supplemental Table 1.

### Western blot

The HAoVSMCs were seeded in the pre-coated 96-well plates at a density of 2000–2500 cells per well in 150 µL of culture medium per well and incubated for 72 h. To identify the expression of SM α-actin, the lysed samples were separated on polyacrylamide gels and transferred to a polyvinylidene fluoride (PVDF) membrane (Millipore, USA). Then the PVDF membranes were incubated with the primary antibodies SM α-actin (1:1000, Abcam) and α-Tubulin (1:2000, MABT205, Millipore) overnight at 4 °C. Afterwards, the membranes were incubated with horseradish peroxidase (HRP)-conjugated secondary antibodies at RT for 1 h. The signal was acquired by using Azure 400 biosystem (Azure, USA). Then, ImageJ software was used to calculate the band intensities, and α-Tubulin antibodies were used as an internal control for signal normalization.

### Microscopy and image processing

Cells were observed time-lapse under a fluorescence microscope (Olympus IX81, 10 × objective) for imaging. Photographs of the samples were taken dynamically using a fluorescence microscope at intervals of 24 h, 48 h, and 72 h. A higher magnification objective, 40 × on Olympus IX81 was used to visualize more details of actin cytoskeleton. The low magnification (4x) objective (Olympus inverted microscope CKX41) was used for the wound healing assay. The images were preprocessed by Fiji-imageJ software (National Institutes of Health, USA) and imported into the Konstanz Information MinEr software (KNIME, www.knime.org) for determining the morphological parameters (E = major axis/minor axis) and (CSI = 4*π*area/perimeter^2^)^30^ of the HAoVSMCs using its customized workflow (Supplemental Fig. 2). The optimizations for the workflow, including picture background optimization, cell image segmentation, gray value calculation, cell morphology comparison, cell phenotyping parameter calculation and classification were performed throughout the analysis. When optimized internal parameters fitted with the photos, the result was generated. For the immunofluorescence experiments, the expression intensity and percentage of positive cells were determined by counting the number of differentiated cells in 10 fields of view for each group.

### Wound healing assay

Following the incubation of HAoVSMCs in 5% FCS media for 48 h, a 96-pin scratch tool was used to apply a consistent mechanical scratch wound (0.4 mm width) to the cellular monolayer in each well. The cells were then washed twice with PBS and maintained in media with low serum (1% FCS) or high serum (10% FCS) amounts. Wound closure was monitored by an automated microscope (Olympus IX81, Munich, Germany) equipped with transmitted light at 0 h and 24 h after scratching cells. Wound closure was determined as percentage of scratch width compared to the respective negative control (regarded as 100%) by using ImageJ software (NIH, Bethesda, MD, USA).

### RNA sequencing

After being seeded in the T25 flasks, the HAoVSMCs were treated with the media containing low and normal amounts of FCS then they were incubated for 48 h. Then the HAoVSMCs were harvested, transferred into RNAlater™ RNA Stabilization Reagent (Qiagen, Germany) and homogenized immediately. MiRNAs were isolated using the Allprep RNA isolation kit (Qiagen, Germany) in accordance with the manufacturer’s protocol and analyzed using Agilent Bioanalyzer 2100 Expert (B.02.08.SI648, Agilent, Santa Clara, CA, USA). After the RNA integrity number (RIN) of miRNA had been identified, the RNA samples were sent to the Beijing Genomics Institute (BGI, Shenzhen, China) for miRNA detection and sequencing service. Filtered miRNAs were quantified per library by realigning reads of at least 17 bp length to predicted miRNAs in QuickMIRSeq^31^. QuickMIRSeq extensively filters the data by joint mapping to the transcriptome and ribosomal RNA to reduce false positives. Sequences were aligned to the reference genome GRCh38.p13. The count data was transformed to log2-counts per million (logCPM) using the voom-function from the limma package^32^ in R. Differential expression analysis was performed using the limma package. A false-positive rate of α = 0.05 with FDR correction was taken as the level of significance. Volcano plots and heatmaps were created using ggplot2 package (version 2.2.1) and the complex Heatmap^33^ (version 2.0.0).

### Statistical and bioinformatics analysis

All the genes derived from the PubMed searching were subjected to analysis using the miRWalk database (version 3.0)^34^. The data obtained from established cell lines are presented as means ± SD from at least three separate experiments, which were performed at least in triplicate. Statistical analysis was carried out using one-way analysis of variance (ANOVA) followed by Bonferroni post hoc for multiple groups or Student’s t-test between two groups. For the immunofluorescence experiments, we described the expression intensity and percentage of positive cells by counting the number of differentiated cells in 10 fields of view for each group.

Then, we used Drug–Gene Interaction Database (DGIdb) (http://www.dgidb.org/), including several drug databases (DrugBank, PharmGKB, ChEMBL), clinical trial databases and literature from PubMed^35^. The top 3 hub target genes of each miRNA were selected from the protein–protein interaction analysis in STRING database (www.string-db.org) with the maximal clique centrality (MCC) method using cytoHubba plugin software in Cytoscape^36^. Then, we imported these target hub genes into DGIdb to explore existing drugs or small organic compounds. Results were displayed using the R packages ggplot2^37^ (version 3.2.1) and ggalluvial (version 0.11.1). Meanwhile, target gene sets of the hit miRNAs were enriched by Gene Set Enrichment Analysis (GSEA) algorithm and KEGG Orthology-Based Annotation System (KOBAS) database (version 3.0). *p* < 0.05 was considered statistically significant (IBM SPSS Statistics 20). * *p* < 0.05, ** *p* < 0.01.

## Results and discussion

### Establishment of quantitative microscopy-based switch assay

VSMCs undergo phenotypic changes when stimulated by environmental changes. Phenotypic change may be induced by low serum starvation, leading the cells to change from the synthetic to the contractile state^38,39^. In our study, we used HAoVSMCs which were isolated from the media of plaque-free regions of the human aorta to model the behavior of VSMCs. For induction of the contractile state cells were incubated in the growth medium containing 5% FCS (see Materials and Methods). The cells stained positive for α-SMA under these conditions. Initially, we have tested whether changing FCS concentration in the growth medium to 1% would further strengthen contractile phenotype as expected^38,39^. Indeed, cells incubated in 1% FCS for 48 h displayed higher intensity of α-SMA specific signal than the control group (Supplemental Fig. 1A). We next tested the migratory potential of cells with the induced contractile phenotype. Following their incubation in low serum medium for 48 h, wounding with a 20 µL pipette tip was done and the gap closure was monitored after 24 h (for more detail description see Materials and Methods). Wound closure after serum starvation was significantly inhibited. In contrast, HAoVSMCs cultured in the high serum medium (10% FCS) showed an obvious increase in migration as compared to the control group (5% FCS) (Supplemental Fig. 1B, C). The potential stimulatory input of the increased serum concentration solely without phenotype change remains to be determined.

As cells in the contractile state were elongated (Supplemental Fig. 1A), we subsequently undertook a quantitative study of the morphology changes. The 384-well plates were fixed with 3% PFA and stained with Hoechst 33,342 to label nuclei and DiIC12(3) to label the whole cell body via targeting the lipid bilayer of plasma membrane^40^. After staining with DiIC12(3), the background of the photographs had a robust signal of interference with limited photobleaching and phototoxicity for optimal imaging. Hence, DiIC12(3) was used for 30 min when the cells were still in suspension before seeding. Machine learning methods were applied to study the definition and threshold optimization of elongation (E) and cell shape index (CSI) of cells cultured under normal or low serum concentrations. During the learning phase, we taught the KNIME software how to distinguish between the contractile and synthetic phenotype of HAoVSMCs and generated an optimized workflow (Supplemental Fig. 2). The larger the E value (E > 1 in the range) is, the slender the cell appears. CSI values indicated how likely the cell morphology resembled a closed circle. The larger the CSI (range 0 < CSI < 1) is, the closer the cell is to a circle. Accordingly, E and CSI values were E > 3 and 0 < CSI < 0.4, for the contractile phenotype, respectively; and 1 < E < 3 and 0.6 < CSI < 1, respectively for the synthetic phenotype. In order to confirm that the KNIME could precisely recognize the different phenotypes of HAoVSMCs, validation was performed by defining the cells manually (~ 1200 cells). By this, we could test and adapt this software to a high number of cells and could evaluate and predict the precision and feasibility of the entire workflow.

**Figure 1:**
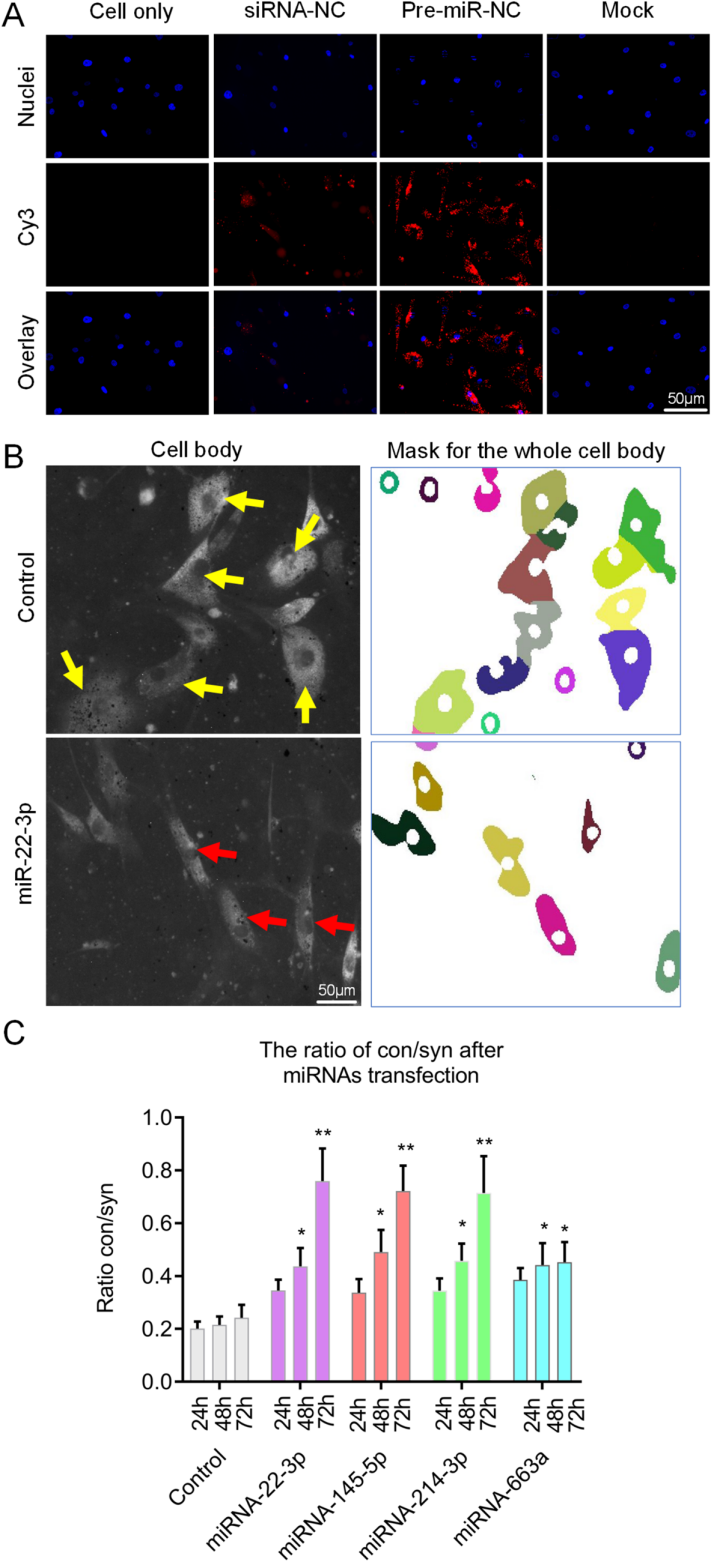
Establishment of microscopy-based phenotype switch assay. (**A**) Transfection efficiency of fluorescently labelled miRNA mimics. scale bar = 50 μm. (**B**) Automated detection of contractile and synthetic phenotypes. Contractile phenotype is indicated by red arrows and the synthetic phenotype is indicated by yellow arrows. scale bar = 50 μm. (**C**) Quantification of the phenotypic switch of HAoVSMCs after miRNA transfection. Compared with the control group, the transfection groups (miR-22, miR-145, miR-214, and miR-663a) appear to have significantly higher ratios of con / syn at 48 h and 72 h. *p < 0.05, **p < 0.01.

**Figure 2:**
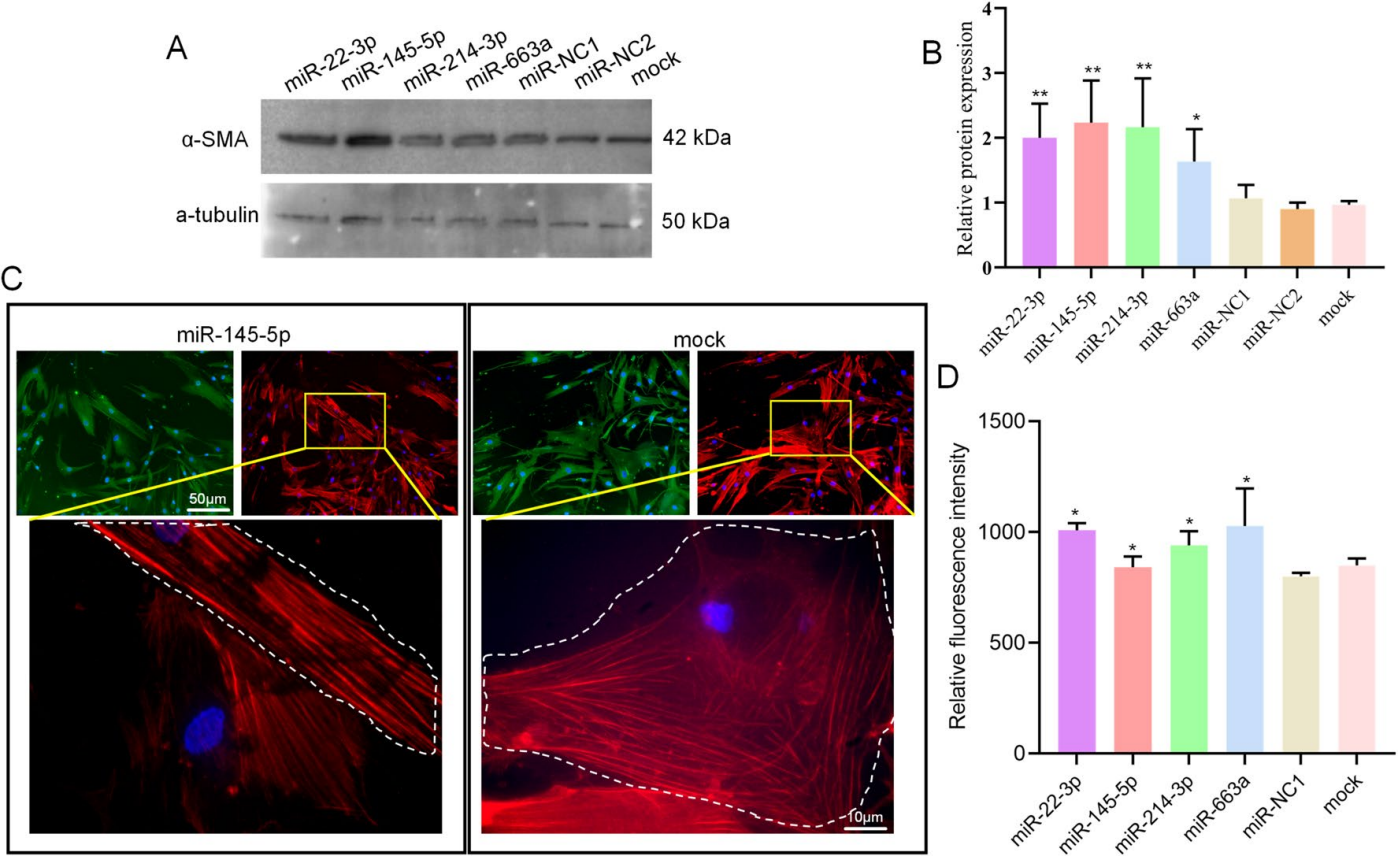
miRNA-induced switch phenotype in HAoVSMCs observed by different methods. An increased expression of α-SMA was measured after 72 h of over-expression of the selected control miRNAs as shown by the WB (**A**, **B**) and IF (**C, D**). The average values in (**B**) are derived from two independent replicates. (**C**) a-SMA and actin were labeled by the antibodies tagged to Alexa488 and phalloidin conjugated to Alexa 647, respectively. Scale bar of the IF images = 50 μm or 10 μm. *p < 0.05, **p < 0.01.

We then tested the transfection efficiency of miRNA mimics by the reverse solid-phase transfection method^41^. Fluorescently labelled miRNAs and previously tested siRNAs efficiently entered HaoVSMC cells grown in 5% FCS medium with nearly each cell being transfected after 24 h of incubation (Fig. 1A and Supplemental Fig. 3). In order to verify that the cell morphology can be used as an effective high-throughput screening method to detect a phenotypic switch, four miRNAs (miR-22-3p, miR-145-5p, miR-214-3p, and miR-663a, (miR-22, miR-145, miR-214, and miR-663a in the following)) that are known to effectively induce a phenotypic switch were transfected^13–17^. Images of the transfected and DiIC12(3) stained cells were taken at intervals of 24 h, 48 h, and 72 h and analyzed in KNIME. After segmentation, the cells were grouped to contractile, synthetic and undecided phenotypic groups (0.4 < CSI < 0.6). Finally, the ratio of contractile / synthetic (Ratio of con / syn) was calculated (Ratio = number of contractile / number of synthetic). The majority of our test miRNAs significantly promoted the conversion of cells into contractile phenotype as compared with the control group (Fig. 1B,C). The ratios of con / syn in HAoVSMCs were increasing after transfection of miR-22, miR-145, miR-214, and miR-663a. Furthermore, we validated the cell shape-based assay by testing α-SMA expression by WB and IF following transfection of the same four miRNAs for 72 h. In agreement to the available literature^8,23^, the cells, when moved to the contractile phenotype under these conditions, expressed up to two times more α-SMA if assessed by WB (Fig. 2A,B, and Supplemental Fig. 7). The increase of α-SMA measured by IF was less, but still statistically significant (Fig. 2C,D). Also, the elongation of the cells and the formation of well-expressed parallel aligned stress actin fibers was observed by using fluorescently tagged phalloidin (Fig. 2C). The elongation of cells can be routed down solely to the activity of the transfected miRNAs as cells treated with the transfection reagent alone (“mock”) or negative control showed no changes in cell morphology and more disorganized actin network. The same can be said about the increased expression levels of α-SMA. To summarize, we established an accurate, reliable, fast, and easy to apply screening method based on cell morphology, which could be up-scaled for screening multiple miRNAs. Until now, the morphological parameters, such as CSI, have not been used in any high-throughput screening approach.

### miRNA overexpression screen

To obtain a list of genes closely related to the phenotypic switch of HAoVSMCs, an extensive literature search was performed using the following search terms: “human vascular smooth muscle cell, phenotypic switch and miRNA (or miR)” in PubMed, covering the last 15 years. As a result, 101 candidate genes with possible direct impact or relation to the phenotypic switch were identified. Then, all the 101 genes were subjected to an analysis using the miRWalk database (version 3.0)^34^, and 87,364 miRNA potential binding sites for these genes were obtained using the filtering options: 3’-UTR, TargetScan, miRDB, and miRtarbase. From those, 10,154 miRNA-gene combinations with clearly evidenced effect in publications on HAoVSMCs were selected. After removing the duplicates, 1554 miRNAs were derived. Finally, by sorting the number of genes potentially influenced by these 1554 miRNAs, the top 50 miRNAs were selected for the screening according to the highest numbers of the predicted targeted genes / per miRNA (Supplemental Fig. 4). By this, we aimed to validate and reconstruct the complex miRNA-mediated regulatory networks required for the phenotypic switch.

The oligos for selected miRNAs over-expression were prepared (Dharmacon, CO, USA) (Supplemental Table 1). 384-well plates were pre-coated with these oligoes^29,42–44^ (three wells per oligo), HAoVSMCs were seeded and incubated for 72 h. Then, the plates were fixed with 3% PFA and stained with Hoechst 33,342 and DiIC12(3). Images were analyzed using the KNIME software and E, CSI, and ratio of con / syn of cells in the treatment group and control group were calculated (Supplemental Fig. 5). The heatmap (Supplemental Fig. 5A) shows the HAoVSMC phenotypic changes after miRNA transfection in each well of a 384-well plate (three independent experiments were performed and indicated as screen A, B, C). Two out of our three positive controls (miR-22 and miR-145) induced the contractile phenotype in all three replicates. We ranked the hits according to the averaged fold changes of their ratios of con / syn to that of the negative control (Supplemental Fig. 5B). According to this ranking, the overexpression of 8 miRNAs (miR-138-5p, miR-150-5p, miR-141-3p, miR-139-5p, miR-338-3p, miR-132-3p, miR-92a-3p and miR-130a-3p) induced a strong switch to the contractile phenotype in all three replicates (the averaged fold change to the negative control is > 1.5). Some of the recent studies match this finding, for example: Chen et al.^45^ indicated that miR-150-5p may exert inhibitory effects on excessive proliferation and migration of pulmonary artery smooth muscle cells (PASMCs). Yet another 15 miRNAs induced a weaker transition (the averaged fold change > 1.2). 15 miRNAs led to a decrease in the averaged ratio of con / syn when over-expressed, indicating that more cells remained in the synthetic state. Five of them (miR-148a-3p, miR-15b-5p, miR-205-5p, miR-486-3p, and miR-93-5p) were strong hits with a decreased ratio in all replicates. Our data correlates to the previously published work for some of these miRNAs; for instance, miR-93-5p is upregulated in proliferating rat HAoVSMCs both in vivo and *in vitro*^46^ and miR-93-5p inhibitor prevented HAoVSMC proliferation and migration in this study. Finally, 10 miRNAs induced no significant changes under these conditions.

### Validation of miRNAs that induce the contractile phenotype

**Figure 3:**
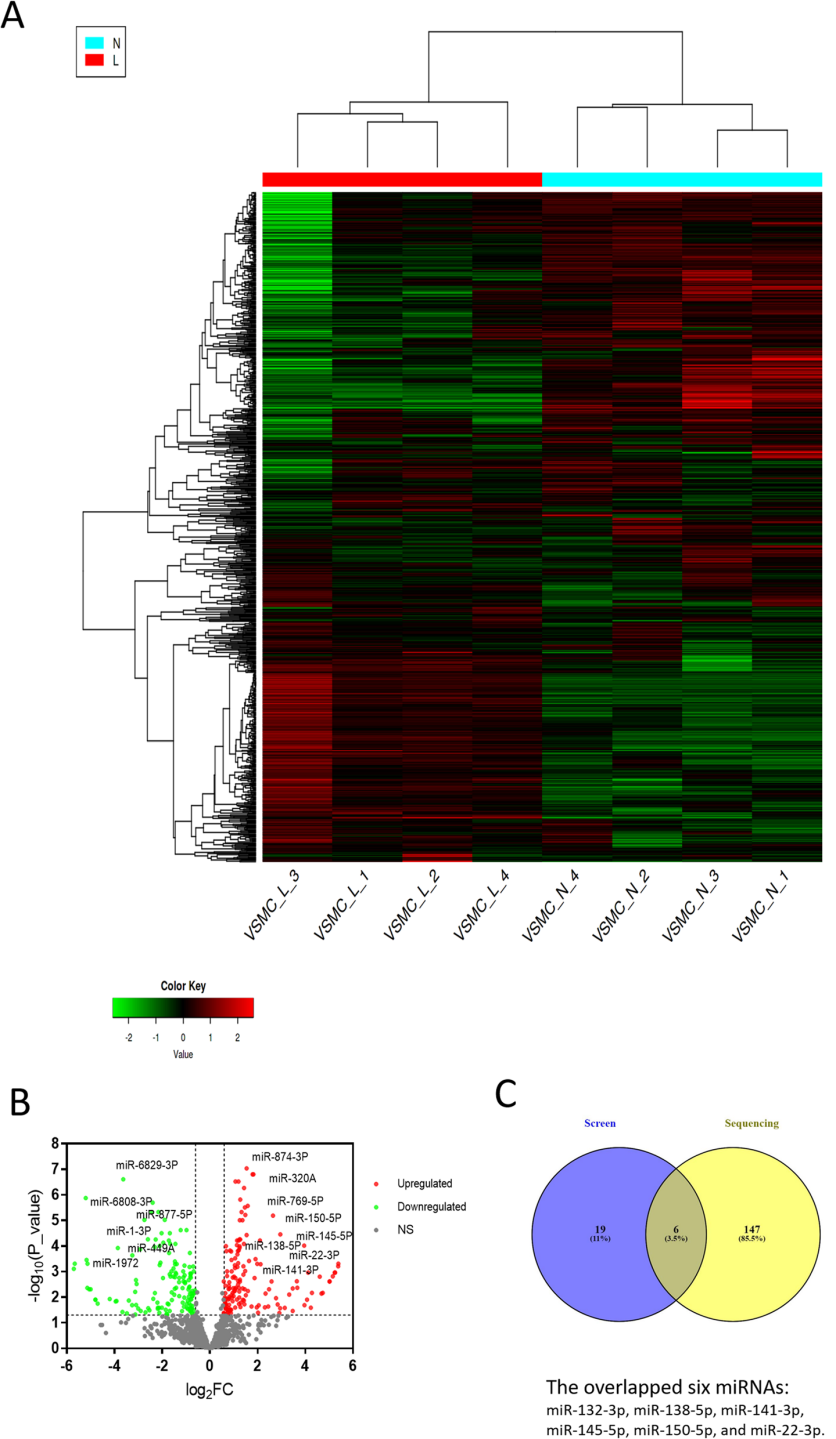
Differential expression of miRNAs in the contractile phenotype comparing with the control group. (**A**) Heatmap^37^ of each replicate, indicating closeness between these groups and the difference between them. Red color indicates high expression of miRNAs, and green color indicates low expression of miRNAs. N: normal serum, L: low serum. (**B**) Volcano plot^37^ shows that the individual up-regulated and down-regulated miRNAs after averaging replicates of the group with the contractile phenotype and the control group. Red dots indicate the upregulated miRNAs, and green dots represent downregulated miRNAs. The thresholds are: upregulated miRNAs (Log_2_FC > 0.6, FC > 1.5, *p* < 0.05), downregulated miRNAs (Log_2_FC < -0.6, FC < 2/3, *p* < 0.05). (**C**) Overlap between the hit miRNAs derived from microscopy-based screening and sequencing.

In order to validate miRNAs that induced the contractile phenotype when overexpressed by the transient transfection, we carried out the miRNA sequencing in contractile HAoVSMC. The HAoVSMC cells were collected under two conditions: the treatment group with 1% FCS, 48 h incubation and the control group with 5% FCS, 48 h of incubation. For each condition four individual replicates were collected. miRNAs were extracted and the RNA integrity number (RIN) of miRNAs was determined and sequenced (Supplemental Table 2). The overall information of these groups was shown in the heatmap of each replicate, indicating closeness and the difference between the groups (Fig. 3A). When averaged over all four replicates, 153 miRNAs were upregulated and 143 miRNAs were downregulated in the contractile cell group compared to the control group, as shown in the volcano plot (Fig. 3B). All miRNAs, analyzed in the microscopy-based screen, but one (hsa-miR-186-3p) were expressed in HAoVSMC cells. Furthermore, 11 miRNAs out of 50 taken for the microscopy screen were upregulated and six miRNAs – downregulated when treating cells with low serum conditions (Supplemental Table 3).

After comparing with the results of the microscopy-based screen and sequencing of miRNAs, we found six overlapping miRNAs (Fig. 3C). All six molecules were hits in the microscopy-based screen when overexpressed and shifted cells to contractile phenotype. In agreement to that, they also were found at elevated levels in cells undergoing a switch to contractile phenotype. Among the over-expressed miRNAs we could identify miR-22 and miR-145 that served as the robust positive controls for the microcopy-based analysis (Fig. 2). Besides, we identified four other molecules that induced the contractile phenotype when over-expressed (miR-132-3p, miR-138-5p, miR-141-3p, and miR-150-5p). The quality of the data is underlined by the observation that all four miRNAs were quantified as strong hits in microscopy-based switch assays (Supplemental Fig. 5).

Furthermore, our hits have been identified as regulators of migration and proliferation in the previous studies via targeting key regulators of gene expression or cell homeostasis, especially, that related to hypoxia-induced responses. For instance, Liu et al.^47^ indicated that overexpression of miR-138 reduced proliferation of human ASMCs (aortic smooth muscle cells), via targeting of 3’-UTR of PDK1 (pyruvate dehydrogenase kinase 1) mRNA. Guo et al.^48^ suggested that overexpression of miR-145 significantly inhibited the expression of CD40 and the differentiation of VSMCs, Chen et al.^45^ indicated that miR-150 may exert inhibitory effects on excessive proliferation and migration of PASMCs through down-regulation of hypoxia-induced factor 1α (HIF-1α).

### miRNA-mediated regulatory networks inducing the contractile phenotype

Having identified promising candidate miRNAs, we next analyzed what common regulatory mechanisms could potentially account for the induction of the contractile phenotype. Not only four top screen hits, but also our positive controls miR-22 and miR-145 were considered as overlapping hits for this analysis (Fig. 3C). All six miRNAs belong to different miRNA seed families and we used miRWalk^34^ to extract the putative target genes for every molecule. In total, we obtained 3,497 putative target genes via miRWalk database for our 6 miRNAs (Supplementary Table 4). Of which, miRNA-145 (n = 738) and miRNA-150 (n = 776) have more target genes than others (data not shown). In order to cluster these targets to the regulatory networks, two online analytic software tools, KOBAS^49^ and Metascape^50^, were employed. Combining the results of the two analyses, a number of pathways^51,52^ already connected to the phenotypic switch of VSMC were identified: MAPK^53^, p53^54^, PI3K-AKT^55^, the calcium signaling pathways^56^, regulators of the cell cycle^57^, TGF-beta signaling pathway^58^, or vascular smooth muscle contraction^59^, herewith demonstrating a high quality of our screens. More closely, we looked to the putative regulators of cell adhesion to the extracellular matrix (ECM) due to their crucial role in regulating cell shape and migration. For instance, there are studies demonstrating, that controlling cell adhesion and shape via micropatterned surfaces, could restore the contractile phenotype of VSMCs *in vitro*^30^. Indeed, 65 targets of these miRNAs are regulators of focal adhesion formation and dynamics (e.g., ARF6, STX16 or PEAK1) with over 40 predicted targets involved in the cell migration.

Next, we analysed whether six miRNAs share their targets and by this potentiate their functional activity. There are no overlapped genes among all six miRNAs, however, even three potential targets are shared among five miRNAs (Supplemental Table 5), despite varying seed sequences. ZBTB20 (Zinc finger and BTB domain-containing protein 20), ZNF704 (Zinc finger protein 704), and EIF4EBP2 (Eukaryotic translation initiation factor 4E-binding protein 2) are the proteins of broad functions in transcription and translation, respectively. No experimental validation data are available for these targets, therefore, further research is needed to test the possibility that switch regulating miRNAs mutually enhance their activities by targeting the same subset of mRNAs. Further 16 targets are shared among four miRNAs. Even six of them are transcription regulators (e.g., CREB1, phosphorylation dependent transcription factor), with other targets encoding regulators of signaling (e.g., MAP3K3 kinase) and protein modifications (e.g., ST3GAL6 sialyltransferase). So far, only two of them are validated by the strong methods including luciferase reporter assay, Western blot and qRT-PCR (CDK6^60^ as a target of miR-145 and CREB1^61^ as a target of miR-150). Therefore, more work is ahead for validation of the remaining predicted miRNA-mRNA interactions. Finally, 83 potential targets were shared by three miRNAs out of six analysed. The group is largely comprised of regulators of transcription, signaling, nucleic acid metabolism or ion transport (Supplemental Table 5), with only little fraction (15 individual miRNA-mRNA interactions) with strong validation proofs available so far.

We also analysed whether the shared targets among four and five miRNAs (19 transcripts collectively) build a network; for this, we used the STRING program^62,63^. The STRING database aims to collect, score and integrate all publicly available sources of protein–protein interaction information and to complement these with computational predictions. Its goal is to achieve a comprehensive and objective global network, including direct (physical) as well as indirect (functional) interactions^63^. The resulting interaction network revealed that few mutual interactions could be detected (data not shown). In order to integrate the overlapping targets into a potential regulatory network, we added 17 of the most interacting potential targets, so called “hub genes”: three for every six analyzed miRNAs (see Materials and Methods) (Supplemental Table 6). One potential target BTRC (F-box/WD repeat-containing protein 1A) is a hub for two miRNAs, namely miR-150 and miR-132. Curiously, CREB1 appeared to be not only the shared target among miR-22^64^, miR-138^65^, miR-141^66^, and miR-150^61^, but also acts as one of the most interacting genes targeted by miR-138^65^. In total, 11 out of 19 shared targets made no direct links, but the other eight targets could be directly combined into the hub-based network (Supplemental Fig. 6); for instance, MAP3K3 kinase by interaction to epidermal growth factor receptor EGFR. Although, MAP3K3 is validated as a weak target of miR-145, there is a good chance for strong and physiologically relevant interaction as EGFR, in turn, interacts with VEGFA (vascular endothelial growth factor) that is also targeted by miR-145. In addition, MAP3K3 and EGFR are predicted targets of miR-141 (Supplemental Table 6), suggesting a functionally meaningful network comprised out of miRNAs and mRNAs. Eight out of 17 hub targets are validated by strong methods (e.g., SIRT1 as a target of miR-22^67^ or SMAD2 as a target for miR-145^68^) and numerous mutual links were found in this group regardless of the present level of their validation. Many hub genes encode either transcription factors (e.g., SDAM2) or components of the ubiquitination machinery of proteins destined for degradation (e.g., KLHL11). The observation goes hand-in-hand with profound changes required for cells to make a global change of their properties when switch phenotype occurs. Not surprisingly, therefore, that many components of such regulatory network may be targeted by numerous miRNAs to ensure high fidelity and efficiency of the response.

### Drug targeting of miRNA-mediated regulatory networks

**Figure 4:**
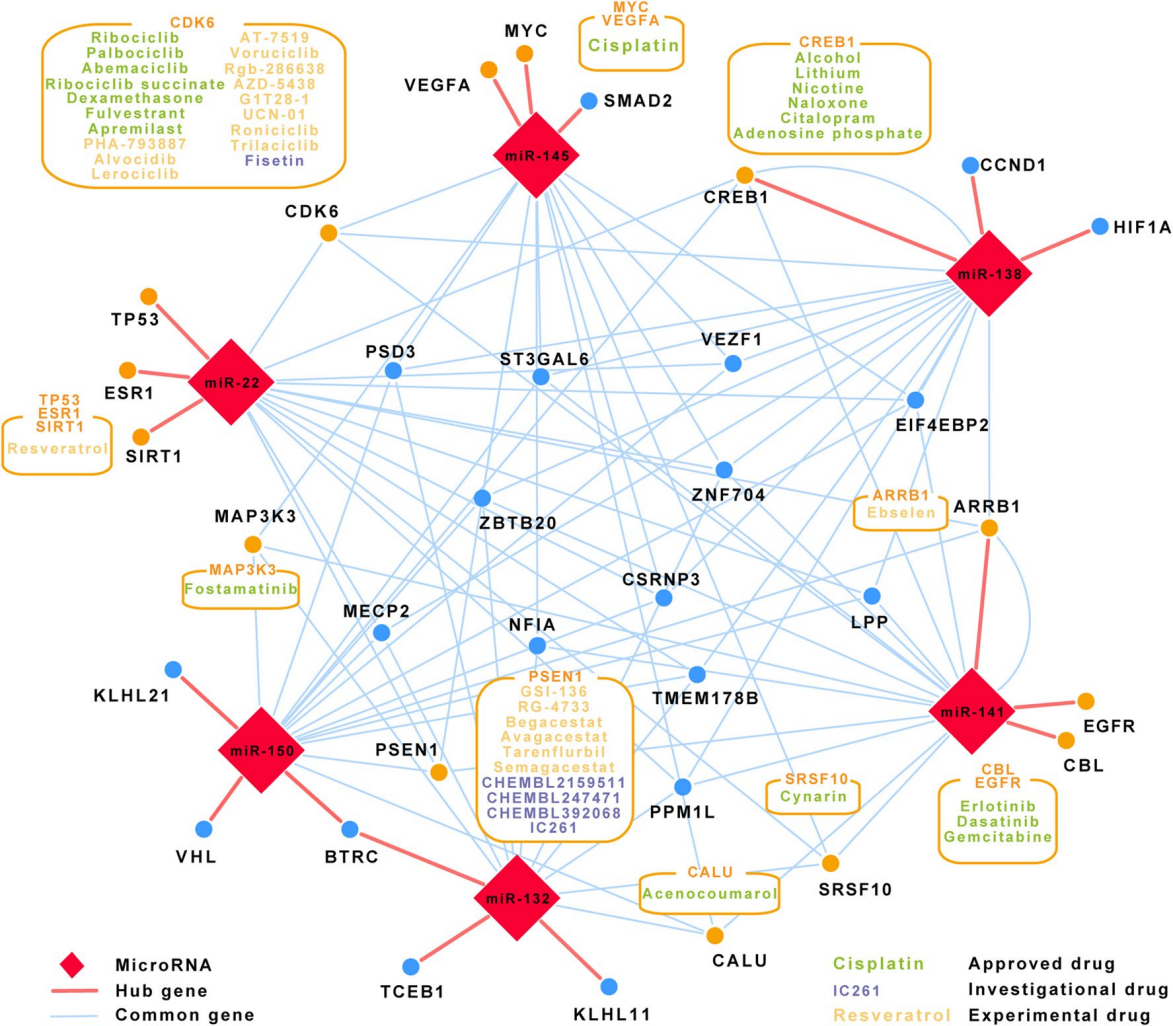
Predicted drugs affecting hub targets and the common targets among four and five miRNAs. (Cytoscape 3.7.2, https://cytoscape.org/index.html).

**Figure 5:**
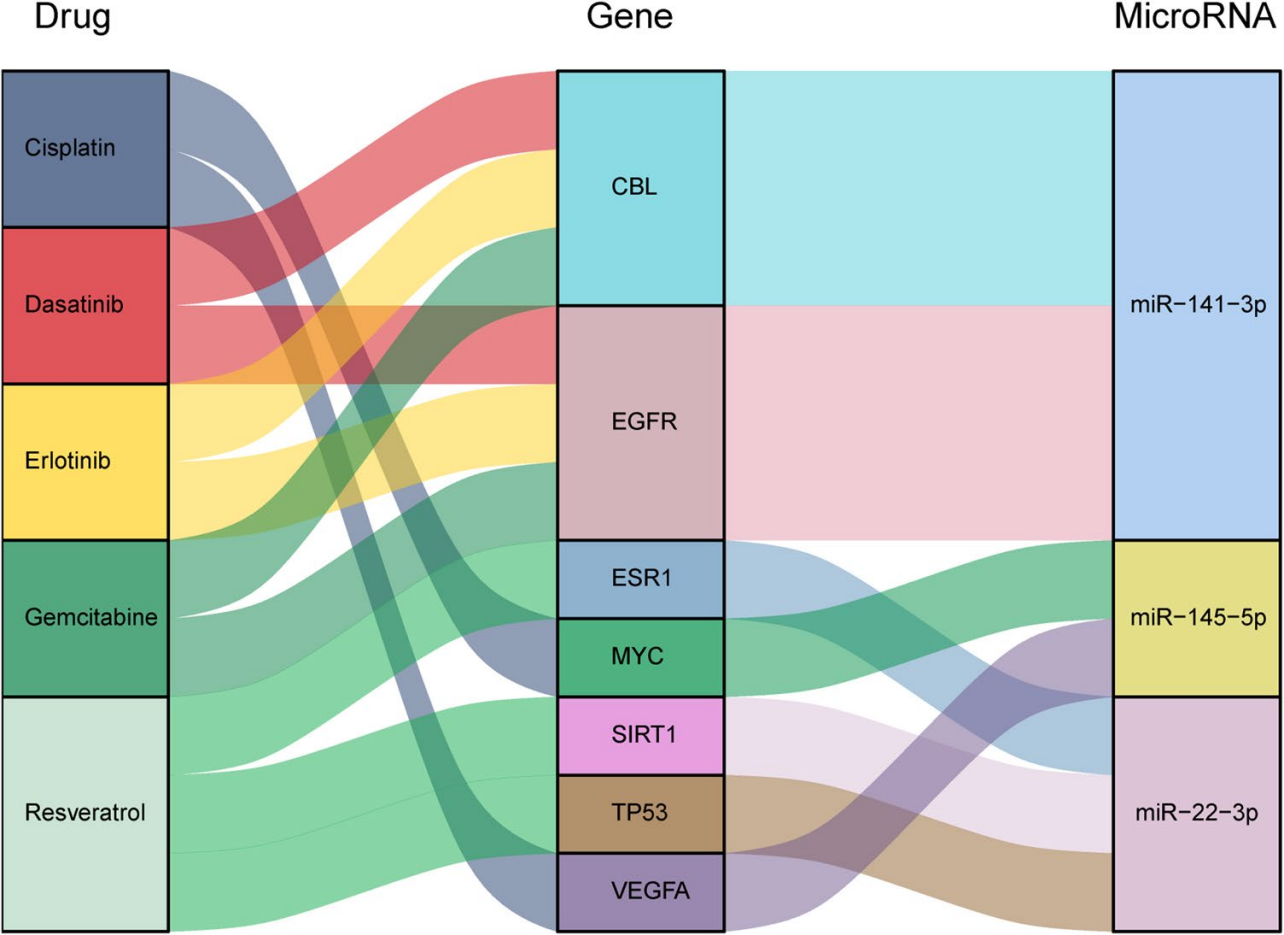
Subset of the combinatorial interactions among miRNAs-targets-drugs. (R packages ggplot2 (version 3.2.1) and ggalluvial (version 0.11.1)).

As the switch phenotype from the contractile to synthetic underlines numerous vascular pathologies, the pharmacological targeting of the key regulators could be a promising option to increase the therapeutic success. Consequently, we utilized the Drug–Gene Interaction Database (DGIdb) (http://www.dgidb.org/) to predict the candidate drugs for the targets of six miRNAs identified in this study (Fig. 4). Initially, we concentrated to find the drugs that influence the activity of the hub targets; and indeed we found that 14 out of 17 hub targets can be linked to approved or experimental drugs. Not surprisingly, one and the same target can be influenced by several drugs. For instance, dasatinib and gemcitabine could be potentially used as a cocktail to efficiently inhibit the activity of EGFR (Fig. 5).

On the other hand, some of these drugs may be effective on several hub proteins simultaneously. For example, Erlotinib could be effective on EGFR^69^ and CBL^70^, both regulated by miR-141, or resveratrol on TP53^71^ and SIRT1^72^, both targeted by miR-22 (Fig. 5). This type of drug-target interactions resemble pleiotropic activity of miRNAs and may be of particular interest if a given miRNA has a limited regulatory activity upon other processes, but switch phenotype. Ideally, a cocktail of drugs could be designed to inhibit key targets of the selected miRNA. Potentially, a lower dose of the individual drug can be used in such a mixture in order to achieve the desired effect, thereby, reducing unspecific and side effects. Knowledge on miRNAs-mRNA interactions within a particular context may serve as recipes for disease tailored or even personalized drug multiplexing.

## Supplementary Information

Supplementary Information.
