# Supplementary material for: High-content analysis of microRNAs involved in the phenotype regulation of vascular smooth muscle cells
Source: Sci Rep. 2022 Mar 3;12:3498. doi: 10.1038/s41598-022-07280-7 (PMC8894385; doi:10.1038/s41598-022-07280-7)
Supplement: Supplementary file 1 — Supplementary Information. [file 41598_2022_7280_MOESM1_ESM.docx]

### **Supplemental Data**


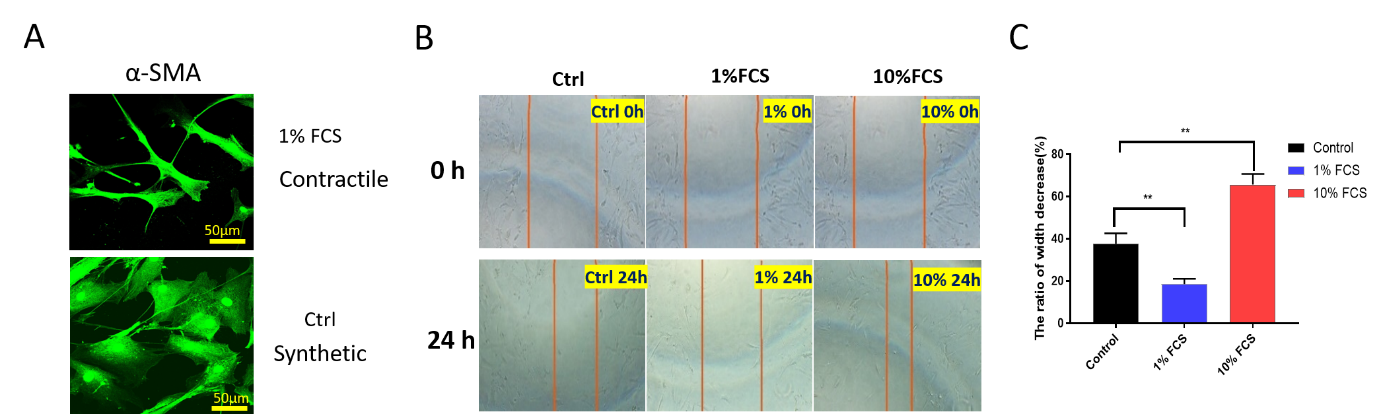


**Supplemental Figure 1. Validation of the contractile and synthetic phenotypes of HAoVSMCs induced by varying serum concentrations in the growth media.** **A.** Increase of α-SMA in HAoVSMCs in low-serum media conditions. **B**. HAoVSMCs showed reduced migration in the contractile phenotype (1% FCS) and increased migration in the synthetic phenotype (10% FCS). **C.** Quantification of the gap closure.


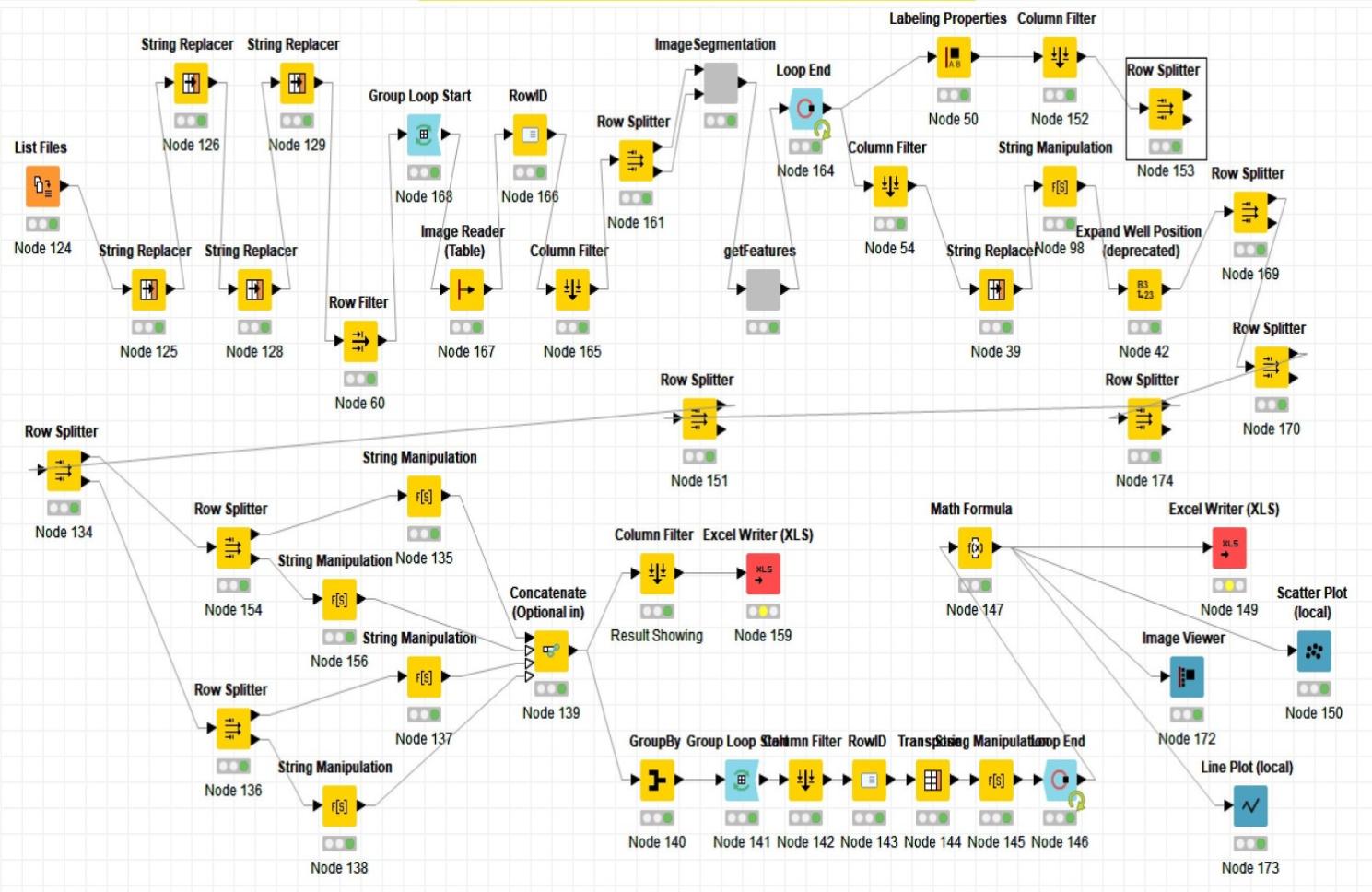


**Supplemental Figure 2. The workflows of KNIME**. It shows all notes in the workflow which can work on the raw data acquired from the fluorescence microscope. The process includes preprocessing and renaming the pictures, segmenting the cells, optimizing the background, and then classifying and counting the cells in these pictures (<https://www.knime.com>).


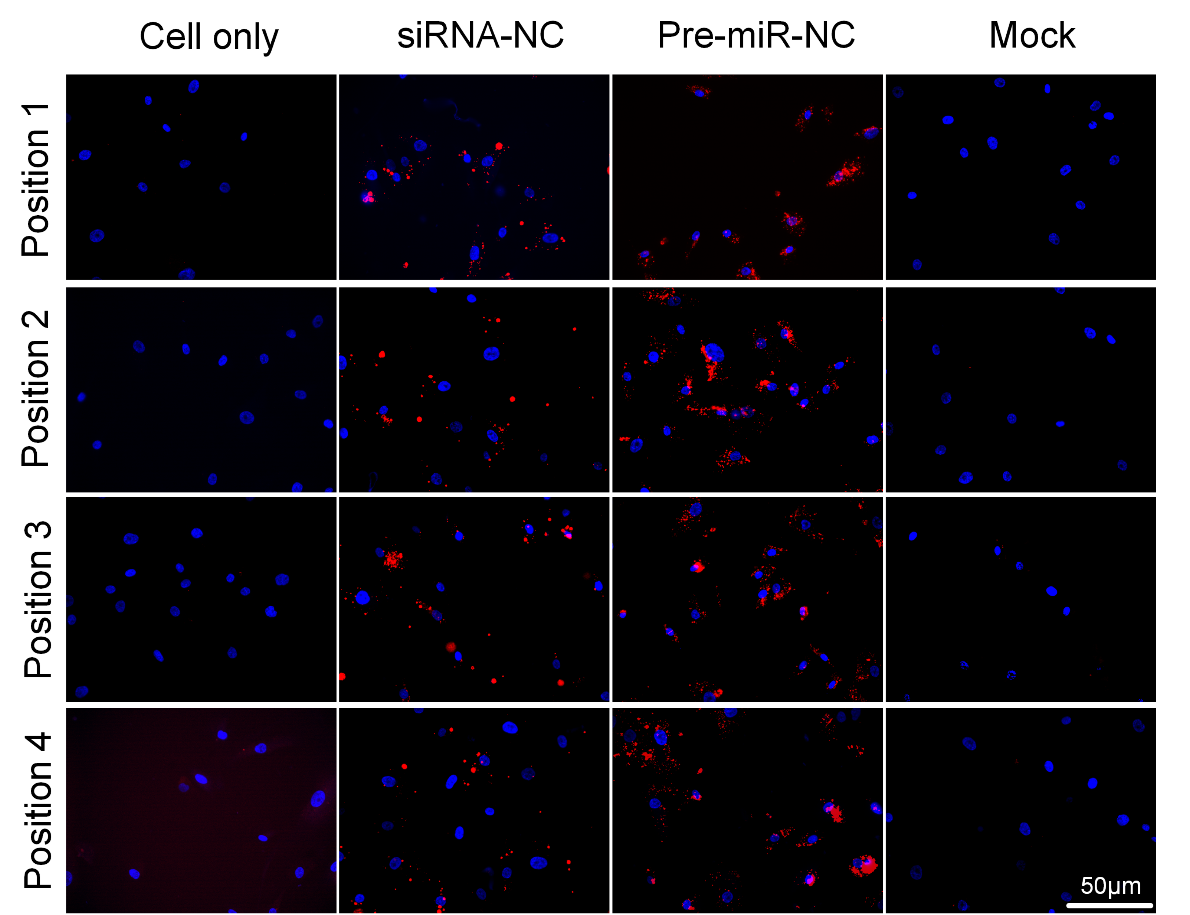


**Supplemental Figure 3. Transfection efficiency of miRNA mimics by the reverse solid-phase transfection method based on 4 random positions.** In each position, fluorescently labelled miRNAs and previously tested siRNAs efficiently entered HaoVSMC cells grown in 5% FCS medium with nearly each cell being transfected after 24h of incubation. scale bar=50μm.


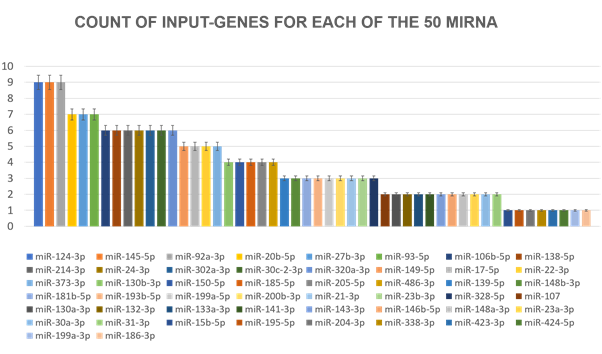


**Supplemental Figure 4. The selection of 50 miRNAs for microscopy-based analysis.** miRNAs were selected according to the number of the predicted targets.


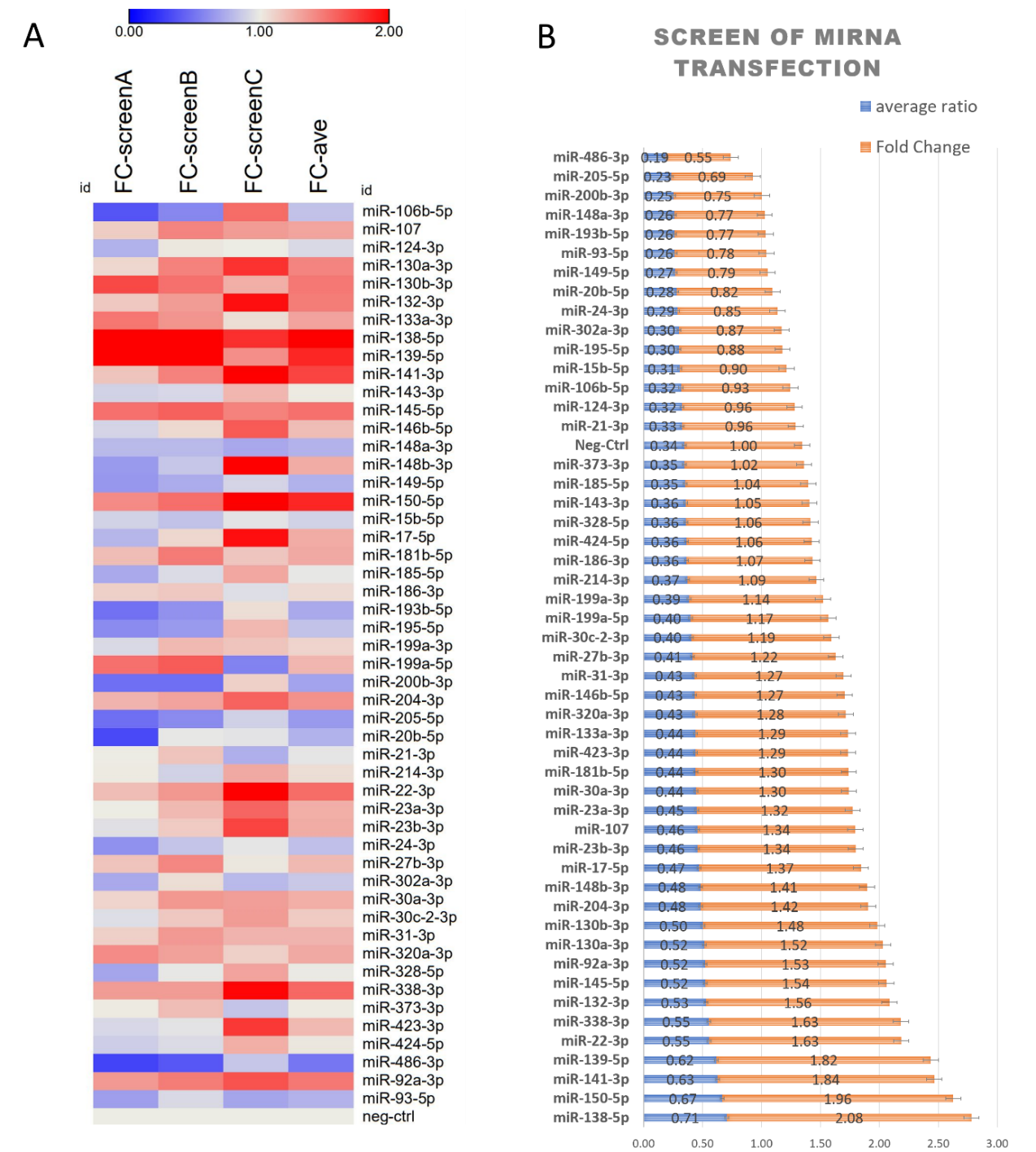


**Supplemental Figure 5. Results of microcopy-based screening of miRNAs.**  **A**. The ratio of con / syn of the HAoVSMCs in different replicates of screening (screen A, B, C) and the average value. The color of the heatmap displays the ratio of con / syn. (Wickham H, 2016). **B**. The ranked fold changes of ratios of con / syn for each miRNA to that of the negative control.


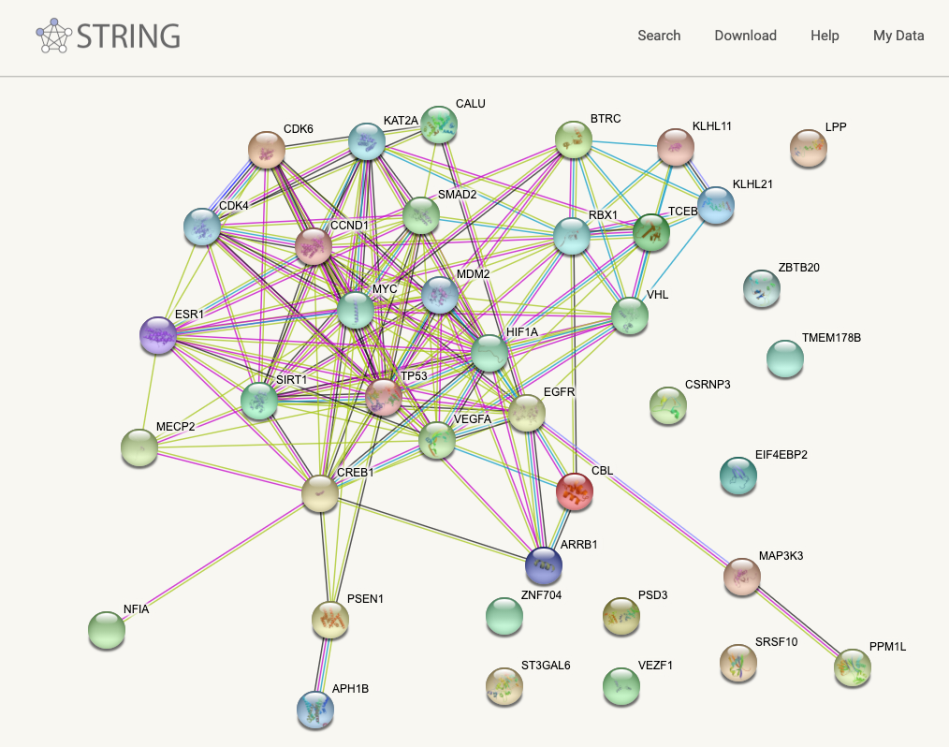


**Supplemental Figure 6.** STRING based interaction network of the potential targets shared by 5 and 4 miRNAs and the potential hub targets ([www.string-db.org](http://www.string-db.org)).


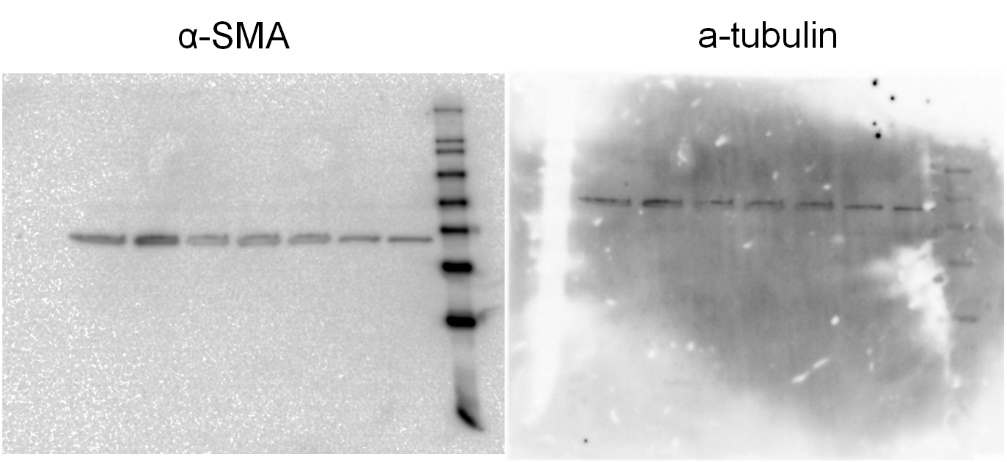


**Supplemental Figure 7.** An increased expression of α-SMA was measured after 72h of over-expression of the selected control miRNAs as shown by the WB.

**Supplemental Table 1:** List of 50 miRNAs for screening.

| **Name of 50 miRNAs** | **Seq from miRBase** | **Seq from miRNA-sequencing** | **Accession number** | **number of target gene** | **miRNAs-ID** | **average ratio of con/syn** | **Score of phenotypic switch** |
| --- | --- | --- | --- | --- | --- | --- | --- |
| hsa-miR-106b-5p | UAAAGUGCUGACAGUGCAGAU | TAAAGTGCTGACAGTGC | MIMAT0000680 | 6 | hsa-miR-106b-5p | 0.32 | 0.93 |
| hsa-miR-107 | AGCAGCAUUGUACAGGGCUAUCA | AGCAGCATTGTACAGGGCT | MIMAT0000104 | 2 | hsa-miR-107 | 0.46 | 1.34 |
| hsa-miR-124-3p | UAAGGCACGCGGUGAAUGCCAA | TAAGGCACGCGGTGAATGCC | MIMAT0000422 | 9 | hsa-miR-124-3p | 0.32 | 0.96 |
| hsa-miR-130a-3p | CAGUGCAAUGUUAAAAGGGCAU | CAGTGCAATGTTAAAAGGGC | MIMAT0000425 | 2 | hsa-miR-130a-3p | 0.52 | 1.52 |
| hsa-miR-130b-3p | CAGUGCAAUGAUGAAAGGGCAU | CAGTGCAATGATGAAAGGGC | MIMAT0000691 | 4 | hsa-miR-130b-3p | 0.50 | 1.48 |
| hsa-miR-132-3p | UAACAGUCUACAGCCAUGGUCG | TAACAGTCTACAGCCAAGG | MIMAT0000426 | 2 | hsa-miR-132-3p | 0.53 | 1.56 |
| hsa-miR-133a-3p | UUUGGUCCCCUUCAACCAGCUG | TTGGTCCCCTTCAACCAGCTGT | MIMAT0000427 | 2 | hsa-miR-133a-3p | 0.44 | 1.29 |
| hsa-miR-138-5p | AGCUGGUGUUGUGAAUCAGGCCG | AGCTGGTGTTGTGAATCAGGCCGT | MIMAT0000430 | 6 | hsa-miR-138-5p | 0.71 | 2.08 |
| hsa-miR-139-5p | UCUACAGUGCACGUGUCUCCAGU | TCTACAGTGCACGTGTCTCCAG | MIMAT0000250 | 3 | hsa-miR-139-5p | 0.62 | 1.82 |
| hsa-miR-141-3p | UAACACUGUCUGGUAAAGAUGG | TAACACTGTCTGGTAAAGA | MIMAT0000432 | 3 | hsa-miR-141-3p | 0.63 | 1.84 |
| hsa-miR-143-3p | UGAGAUGAAGCACUGUAGCUC | TGAGATGAAGCACTGTAGCTC | MIMAT0000435 | 2 | hsa-miR-143-3p | 0.36 | 1.05 |
| hsa-miR-145-5p | GUCCAGUUUUCCCAGGAAUCCCU | GTCCAGTTTTCCCAGGAATCCCT | MIMAT0000437 | 9 | hsa-miR-145-5p | 0.52 | 1.54 |
| hsa-miR-146b-5p | UGAGAACUGAAUUCCAUAGGCUG | TGAGAACTGAATTCCATAGGCTGT | MIMAT0002809 | 2 | hsa-miR-146b-5p | 0.43 | 1.27 |
| hsa-miR-148a-3p | UCAGUGCACUACAGAACUUUGU | TCAGTGCACTACAGAACTTTGTC | MIMAT0000243 | 2 | hsa-miR-148a-3p | 0.26 | 0.77 |
| hsa-miR-148b-3p | UCAGUGCAUCACAGAACUUUGU | TCAGTGCATCACAGAACTTTGTT | MIMAT0000759 | 3 | hsa-miR-148b-3p | 0.48 | 1.41 |
| hsa-miR-149-5p | UCUGGCUCCGUGUCUUCACUCCC | TCTGGCTCCGTGTCTTCACTCCC | MIMAT0000450 | 5 | hsa-miR-149-5p | 0.27 | 0.79 |
| hsa-miR-150-5p | UCUCCCAACCCUUGUACCAGUG | TCTCCCAACCCTTGTACCAGTG | MIMAT0000451 | 4 | hsa-miR-150-5p | 0.67 | 1.96 |
| hsa-miR-15b-5p | UAGCAGCACAUCAUGGUUUACA | TAGCAGCACATCATGGTTTAC | MIMAT0000417 | 1 | hsa-miR-15b-5p | 0.31 | 0.90 |
| hsa-miR-17-5p | CAAAGUGCUUACAGUGCAGGUAG | CAAAGTGCTTACAGTGCAGGTAGT | MIMAT0000070 | 5 | hsa-miR-17-5p | 0.47 | 1.37 |
| hsa-miR-181b-5p | AACAUUCAUUGCUGUCGGUGGGU | AACATTCATTGCTGTCGGTGGGT | MIMAT0000257 | 3 | hsa-miR-181b-5p | 0.44 | 1.30 |
| hsa-miR-185-5p | UGGAGAGAAAGGCAGUUCCUGA | TGGAGAGAAAGGCAGTTCCTG | MIMAT0000455 | 4 | hsa-miR-185-5p | 0.35 | 1.04 |
| hsa-miR-186-3p | GCCCAAAGGUGAAUUUUUUGGG | gcccaaaggugaauuuuuuggg | MIMAT0004612 | 1 | hsa-miR-186-3p | 0.36 | 1.07 |
| hsa-miR-193b-5p | CGGGGUUUUGAGGGCGAGAUGA | CGGGGTTTTGAGGGCGAGATG | MIMAT0004767 | 3 | hsa-miR-193b-5p | 0.26 | 0.77 |
| hsa-miR-195-5p | UAGCAGCACAGAAAUAUUGGC | TAGCAGCACAGAAATATTGGC | MIMAT0000461 | 1 | hsa-miR-195-5p | 0.30 | 0.88 |
| hsa-miR-199a-3p | ACAGUAGUCUGCACAUUGGUUA | ACAGTAGTCTGCACATTGGTT | MIMAT0000232 | 3 | hsa-miR-199a-3p | 0.39 | 1.14 |
| hsa-miR-199a-5p | CCCAGUGUUCAGACUACCUGUUC | CCCAGTGTTCAGACTACCTGTTC | MIMAT0000231 | 3 | hsa-miR-199a-5p | 0.40 | 1.17 |
| hsa-miR-200b-3p | UAAUACUGCCUGGUAAUGAUGA | TAATACTGCCTGGTAATGATGAC | MIMAT0000318 | 3 | hsa-miR-200b-3p | 0.25 | 0.75 |
| hsa-miR-204-3p | GCUGGGAAGGCAAAGGGACGU | GCTGGGAAGGCAAAGGGACGT | MIMAT0022693 | 2 | hsa-miR-204-3p | 0.48 | 1.42 |
| hsa-miR-205-5p | UCCUUCAUUCCACCGGAGUCUG | TCCTTCATTCCACCGGAGTCTG | MIMAT0000266 | 6 | hsa-miR-205-5p | 0.23 | 0.69 |
| hsa-miR-20b-5p | CAAAGUGCUCAUAGUGCAGGUAG | CAAAGTGCTCATAGTGCAGGTAG | MIMAT0001413 | 7 | hsa-miR-20b-5p | 0.28 | 0.82 |
| hsa-miR-21-3p | CAACACCAGUCGAUGGGCUGU | CAACACCAGTCGATGGGCTGT | MIMAT0004494 | 3 | hsa-miR-21-3p | 0.33 | 0.96 |
| hsa-miR-214-3p | ACAGCAGGCACAGACAGGCAGU | ACAGCAGGCACAGACAGGCAGT | MIMAT0000271 | 6 | hsa-miR-214-3p | 0.37 | 1.09 |
| hsa-miR-22-3p | AAGCUGCCAGUUGAAGAACUGU | AAGCTGCCAGTTGAAGAACTGT | MIMAT0000077 | 5 | hsa-miR-22-3p | 0.55 | 1.63 |
| hsa-miR-23a-3p | AUCACAUUGCCAGGGAUUUCC | ATCACATTGCCAGGGATTTCC | MIMAT0000078 | 3 | hsa-miR-23a-3p | 0.45 | 1.32 |
| hsa-miR-23b-3p | AUCACAUUGCCAGGGAUUACCAC | ATCACATTGCCAGGGATTACCACT | MIMAT0000418 | 4 | hsa-miR-23b-3p | 0.46 | 1.34 |
| hsa-miR-24-3p | UGGCUCAGUUCAGCAGGAACAG | TGGCTCAGTTCAGCAGGAACAG | MIMAT0000080 | 6 | hsa-miR-24-3p | 0.29 | 0.85 |
| hsa-miR-27b-3p | UUCACAGUGGCUAAGUUCUGC | TTCACAGTGGCTAAGTTCTGC | MIMAT0000419 | 7 | hsa-miR-27b-3p | 0.41 | 1.22 |
| hsa-miR-302a-3p | UAAGUGCUUCCAUGUUUUGGUGA | TAAGTGCTTCCATGTTTTGGTG | MIMAT0000684 | 6 | hsa-miR-302a-3p | 0.30 | 0.87 |
| hsa-miR-30a-3p | CUUUCAGUCGGAUGUUUGCAGC | CTTTCAGTCGGATGTTTGCAGC | MIMAT0000088 | 2 | hsa-miR-30a-3p | 0.44 | 1.30 |
| hsa-miR-30c-2-3p | CUGGGAGAAGGCUGUUUACUCU | CTGGGAGAAGGCTGTTTACTC | MIMAT0004550 | 6 | hsa-miR-30c-2-3p | 0.40 | 1.19 |
| hsa-miR-31-3p | UGCUAUGCCAACAUAUUGCCAU | TGCTATGCCAACATATTGCCAT | MIMAT0004504 | 2 | hsa-miR-31-3p | 0.43 | 1.27 |
| hsa-miR-320a-3p | AAAAGCUGGGUUGAGAGGGCGA | aaaagcuggguugagagggcga | MIMAT0000510 | 6 | hsa-miR-320a-3p | 0.43 | 1.28 |
| hsa-miR-328-5p | GGGGGGGCAGGAGGGGCUCAGGG | GGGGGGCAGGAGGGGCTCAGGGT | MIMAT0026486 | 3 | hsa-miR-328-5p | 0.36 | 1.06 |
| hsa-miR-338-3p | UCCAGCAUCAGUGAUUUUGUUG | TCCAGCATCAGTGATTTTGT | MIMAT0000763 | 2 | hsa-miR-338-3p | 0.55 | 1.63 |
| hsa-miR-373-3p | GAAGUGCUUCGAUUUUGGGGUGU | GAAGTGCTTCGATTTTGGGGTGT | MIMAT0000726 | 5 | hsa-miR-373-3p | 0.35 | 1.02 |
| hsa-miR-423-3p | AGCUCGGUCUGAGGCCCCUCAGU | AGCTCGGTCTGAGGCCCCTCAGT | MIMAT0001340 | 1 | hsa-miR-423-3p | 0.44 | 1.29 |
| hsa-miR-424-5p | CAGCAGCAAUUCAUGUUUUGAA | CAGCAGCAATTCATGTTTTG | MIMAT0001341 | 1 | hsa-miR-424-5p | 0.36 | 1.06 |
| hsa-miR-486-3p | CGGGGCAGCUCAGUACAGGAU | CGGGGCAGCTCAGTACAGGAT | MIMAT0004762 | 4 | hsa-miR-486-3p | 0.19 | 0.55 |
| hsa-miR-92a-3p | UAUUGCACUUGUCCCGGCCUGU | TATTGCACTTGTCCCGGCCTGT | MIMAT0000092 | 9 | hsa-miR-92a-3p | 0.52 | 1.53 |
| hsa-miR-93-5p | CAAAGUGCUGUUCGUGCAGGUAG | CAAAGTGCTGTTCGTGCAGGT | MIMAT0000093 | 7 | hsa-miR-93-5p | 0.26 | 0.78 |
|  |  |  |  |  |  |  |  |
| Cy3 labelled oligoes | Sense: 5'->3' | Antisense: 5'->3' |  |  |  |  |  |
| siRNA-NC | UAACGACGCGACGACGUAAtt | UUACGUCGUCGCGUCGUUAtt |  |  |  |  |  |
| Pre-miR-NC | random sequence |  |  |  |  |  |  |

**Supplemental Table 2:** The result of sequencing data with accession number and sequences .

| Name of miRNA | Seq | Accession | miRNA ID | VSMC_N_1 | VSMC_N_2 | VSMC_N_3 | VSMC_N_4 | VSMC_L_1 | VSMC_L_2 | VSMC_L_3 | VSMC_L_4 |
| --- | --- | --- | --- | --- | --- | --- | --- | --- | --- | --- | --- |
| hsa-let-7a-2-3p | CTGTACAGCCTCCTAGCTTTCC | MIMAT0010195 | hsa-let-7a-2-3p | 859 | 1132 | 828 | 924 | 905 | 764 | 704 | 967 |
| hsa-let-7a-3p | CTATACAATCTACTGTCTTTCT | MIMAT0004481 | hsa-let-7a-3p | 97 | 180 | 127 | 121 | 106 | 124 | 188 | 163 |
| hsa-let-7a-5p | TGAGGTAGTAGGTTGTATAGTT | MIMAT0000062 | hsa-let-7a-5p | 1892010 | 2113477 | 1845790 | 2569972 | 2630073 | 1957166 | 291867 | 2182552 |
| hsa-let-7b-3p | CTATACAACCTACTGCCTTCCT | MIMAT0004482 | hsa-let-7b-3p | 806 | 1882 | 474 | 1468 | 1948 | 2039 | 1379 | 2331 |
| hsa-let-7b-5p | TGAGGTAGTAGGTTGTGTGGTT | MIMAT0000063 | hsa-let-7b-5p | 3262068 | 4659653 | 3727936 | 4782586 | 5653725 | 4946411 | 2832873 | 3978825 |
| hsa-let-7c-3p | CTGTACAACCTTCTAGCTTTCC | MIMAT0026472 | hsa-let-7c-3p | 4 | 4 | 0 | 0 | 10 | 10 | 27 | 11 |
| hsa-let-7c-5p | TGAGGTAGTAGGTTGTATGGTT | MIMAT0000064 | hsa-let-7c-5p | 113329 | 141547 | 123584 | 162288 | 189125 | 151339 | 45636 | 148902 |
| hsa-let-7d-3p | CTATACGACCTGCTGCCTTTCT | MIMAT0004484 | hsa-let-7d-3p | 43604 | 97186 | 47013 | 55323 | 79369 | 76044 | 141057 | 107480 |
| hsa-let-7d-5p | AGAGGTAGTAGGTTGCATAGTT | MIMAT0000065 | hsa-let-7d-5p | 65299 | 73991 | 64662 | 69329 | 72100 | 52796 | 13280 | 67604 |
| hsa-let-7e-3p | CTATACGGCCTCCTAGCTTTCC | MIMAT0004485 | hsa-let-7e-3p | 1171 | 1700 | 1132 | 1465 | 1454 | 1333 | 1393 | 1346 |
| hsa-let-7e-5p | TGAGGTAGGAGGTTGTATAGTT | MIMAT0000066 | hsa-let-7e-5p | 189093 | 248527 | 179807 | 239738 | 259353 | 228539 | 45575 | 284459 |
| hsa-let-7f-1-3p | CTATACAATCTATTGCCTTCCT | MIMAT0004486 | hsa-let-7f-1-3p | 34 | 80 | 29 | 55 | 49 | 38 | 24 | 67 |
| hsa-let-7f-2-3p | CTATACAGTCTACTGTCTTTCT | MIMAT0004487 | hsa-let-7f-2-3p | 7 | 12 | 5 | 4 | 0 | 3 | 0 | 1 |
| hsa-let-7f-5p | TGAGGTAGTAGATTGTATAGTT | MIMAT0000067 | hsa-let-7f-5p | 56162 | 74768 | 52275 | 72636 | 59119 | 41925 | 8876 | 65424 |
| hsa-let-7g-3p | CTGTACAGGCCACTGCCTTGCC | MIMAT0004584 | hsa-let-7g-3p | 1 | 11 | 4 | 11 | 16 | 10 | 26 | 21 |
| hsa-let-7g-5p | TGAGGTAGTAGTTTGTACAGTT | MIMAT0000414 | hsa-let-7g-5p | 91224 | 110720 | 83938 | 125997 | 146454 | 101641 | 43266 | 109759 |
| hsa-let-7i-3p | CTGCGCAAGCTACTGCCTTGTT | MIMAT0004585 | hsa-let-7i-3p | 363 | 873 | 430 | 887 | 1218 | 884 | 2137 | 1087 |
| hsa-let-7i-5p | TGAGGTAGTAGTTTGTGCTGTT | MIMAT0000415 | hsa-let-7i-5p | 954858 | 1112176 | 842016 | 1349921 | 1601075 | 1274012 | 781763 | 986908 |
| hsa-miR-1-3p | TGGAATGTAAAGAAGTATGT | MIMAT0000416 | hsa-miR-1-3p | 33 | 136 | 29 | 36 | 10 | 6 | 10 | 16 |
| hsa-miR-100-3p | CAAGCTTGTATCTATAGGTATG | MIMAT0004512 | hsa-miR-100-3p | 2 | 1 | 5 | 2 | 0 | 0 | 0 | 4 |
| hsa-miR-100-5p | AACCCGTAGATCCGAACTTGTG | MIMAT0000098 | hsa-miR-100-5p | 529647 | 831479 | 507105 | 618335 | 501857 | 423251 | 367062 | 511771 |
| hsa-miR-101-3p | GTACAGTACTGTGATAACTGA | MIMAT0000099 | hsa-miR-101-3p | 3 | 1 | 2 | 0 | 2 | 5 | 0 | 2 |
| hsa-miR-103a-2-5p | AGCTTCTTTACAGTGCTGCCTTG | MIMAT0009196 | hsa-miR-103a-2-5p | 2 | 3 | 3 | 10 | 11 | 8 | 27 | 8 |
| hsa-miR-103a-3p | AGCAGCATTGTACAGGGCTATG | MIMAT0000101 | hsa-miR-103a-3p | 5484 | 6744 | 5790 | 5771 | 5642 | 4304 | 4948 | 4477 |
| hsa-miR-105-5p | AAATGCTCAGACTCCTGTGGTG | MIMAT0000102 | hsa-miR-105-5p | 0 | 0 | 0 | 0 | 0 | 0 | 0 | 0 |
| hsa-miR-106a-5p | AAAGTGCTTACAGTGCAGGT | MIMAT0000103 | hsa-miR-106a-5p | 1 | 1 | 5 | 1 | 0 | 0 | 0 | 4 |
| hsa-miR-106b-3p | CCGCACTGTGGGTACTTGCT | MIMAT0004672 | hsa-miR-106b-3p | 596 | 826 | 603 | 852 | 1319 | 1204 | 2010 | 1105 |
| hsa-miR-106b-5p | TAAAGTGCTGACAGTGC | MIMAT0000680 | hsa-miR-106b-5p | 6 | 5 | 10 | 10 | 8 | 10 | 0 | 4 |
| hsa-miR-107 | AGCAGCATTGTACAGGGCT | MIMAT0000104 | hsa-miR-107 | 11 | 16 | 11 | 27 | 29 | 31 | 51 | 25 |
| hsa-miR-10a-3p | CAAATTCGTATCTAGGGGAAT | MIMAT0004555 | hsa-miR-10a-3p | 66 | 95 | 105 | 88 | 29 | 29 | 25 | 82 |
| hsa-miR-10a-5p | ACCCTGTAGATCCGAATTTGTG | MIMAT0000253 | hsa-miR-10a-5p | 5519 | 8366 | 5883 | 7447 | 5765 | 4972 | 5135 | 6745 |
| hsa-miR-10b-3p | AGATTCGATTCTAGGGGAAT | MIMAT0004556 | hsa-miR-10b-3p | 0 | 0 | 0 | 0 | 0 | 0 | 0 | 0 |
| hsa-miR-10b-5p | TACCCTGTAGAACCGAATTTGTG | MIMAT0000254 | hsa-miR-10b-5p | 9 | 33 | 17 | 19 | 16 | 10 | 29 | 13 |
| hsa-miR-1178-3p | TTGCTCACTGTTCTTCCCTAGT | MIMAT0005823 | hsa-miR-1178-3p | 0 | 0 | 0 | 0 | 0 | 0 | 0 | 0 |
| hsa-miR-1179 | AAGCATTCTTTCATTGGTTGGT | MIMAT0005824 | hsa-miR-1179 | 0 | 3 | 0 | 4 | 2 | 0 | 0 | 0 |
| hsa-miR-1180-3p | TTTCCGGCTCGCGTGGGTGTGT | MIMAT0005825 | hsa-miR-1180-3p | 78062 | 137411 | 85559 | 94955 | 165818 | 150168 | 313399 | 173646 |
| hsa-miR-1180-5p | GGACCCACCCGGCCGGGAAT | MIMAT0026735 | hsa-miR-1180-5p | 2 | 2 | 9 | 3 | 2 | 0 | 0 | 0 |
| hsa-miR-1183 | CACUGUAGGUGAUGGUGAGAGUGGGCA | MIMAT0005828 | hsa-miR-1183 | 0 | 0 | 0 | 0 | 0 | 0 | 0 | 0 |
| hsa-miR-1185-1-3p | ATATACAGGGGGAGACTCTTAT | MIMAT0022838 | hsa-miR-1185-1-3p | 14 | 29 | 11 | 28 | 62 | 66 | 48 | 65 |
| hsa-miR-1185-2-3p | ATATACAGGGGGAGACTCTCAT | MIMAT0022713 | hsa-miR-1185-2-3p | 11 | 8 | 9 | 10 | 11 | 6 | 0 | 0 |
| hsa-miR-1185-5p | AGAGGATACCCTTTGTATGTTC | MIMAT0005798 | hsa-miR-1185-5p | 9 | 16 | 16 | 9 | 12 | 17 | 20 | 9 |
| hsa-miR-1193 | GGGATGGTAGACCGGTGACGTGC | MIMAT0015049 | hsa-miR-1193 | 8 | 4 | 4 | 7 | 18 | 10 | 22 | 15 |
| hsa-miR-1197 | TAGGACACATGGTCTACTTCT | MIMAT0005955 | hsa-miR-1197 | 3 | 19 | 6 | 3 | 0 | 0 | 0 | 2 |
| hsa-miR-1207-5p | CAGGGAGGCTGGGCGGGG | MIMAT0005871 | hsa-miR-1207-5p | 0 | 0 | 0 | 0 | 0 | 0 | 0 | 0 |
| hsa-miR-122-3p | AACGCCATTATCACACTAAAT | MIMAT0004590 | hsa-miR-122-3p | 2 | 3 | 0 | 2 | 9 | 10 | 19 | 7 |
| hsa-miR-122-5p | TGGAGTGTGACAATGGTGTTT | MIMAT0000421 | hsa-miR-122-5p | 96 | 111 | 77 | 60 | 164 | 136 | 288 | 173 |
| hsa-miR-1224-3p | CCCCGCCTCCTCTCTCCTC | MIMAT0005459 | hsa-miR-1224-3p | 1 | 0 | 1 | 0 | 0 | 0 | 0 | 0 |
| hsa-miR-1224-5p | GTGAGGACTCGGGAGGTGGAGG | MIMAT0005458 | hsa-miR-1224-5p | 40 | 28 | 42 | 32 | 64 | 51 | 103 | 44 |
| hsa-miR-1225-3p | GAGCCCCTGTGCCGCCCCCAG | MIMAT0005573 | hsa-miR-1225-3p | 0 | 0 | 0 | 0 | 0 | 0 | 0 | 0 |
| hsa-miR-1225-5p | GTGGGTACGGCCCAGTGGGG | MIMAT0005572 | hsa-miR-1225-5p | 36 | 18 | 62 | 28 | 10 | 7 | 0 | 3 |
| hsa-miR-1226-3p | TCACCAGCCCTGTGTTCCCTAG | MIMAT0005577 | hsa-miR-1226-3p | 478 | 601 | 970 | 608 | 282 | 321 | 170 | 312 |
| hsa-miR-1226-5p | GTGAGGGCATGCAGGCCTGGATGGGG | MIMAT0005576 | hsa-miR-1226-5p | 17 | 49 | 35 | 18 | 41 | 43 | 59 | 33 |
| hsa-miR-1227-3p | CGTGCCACCCTTTTCCCC | MIMAT0005580 | hsa-miR-1227-3p | 205 | 307 | 134 | 206 | 242 | 184 | 254 | 261 |
| hsa-miR-1228-3p | TCACACCTGCCTCGCCCCCC | MIMAT0005583 | hsa-miR-1228-3p | 35 | 78 | 41 | 45 | 82 | 82 | 129 | 75 |
| hsa-miR-1228-5p | TGGGCGGGGGCAGGTGTG | MIMAT0005582 | hsa-miR-1228-5p | 23 | 37 | 23 | 25 | 49 | 54 | 42 | 66 |
| hsa-miR-1229-3p | CTCTCACCACTGCCCTCCCACAG | MIMAT0005584 | hsa-miR-1229-3p | 24 | 55 | 13 | 29 | 27 | 31 | 12 | 38 |
| hsa-miR-1229-5p | GTGGGTAGGGTTTGGGGGAGAGCG | MIMAT0022942 | hsa-miR-1229-5p | 2 | 0 | 0 | 2 | 0 | 3 | 0 | 0 |
| hsa-miR-1231 | GTCAGTGTCTGGGCGGACAGC | MIMAT0005586 | hsa-miR-1231 | 0 | 0 | 0 | 0 | 0 | 0 | 0 | 4 |
| hsa-miR-1233-3p | CTGAGCCCTGTCCTCCCGC | MIMAT0005588 | hsa-miR-1233-3p | 16 | 28 | 28 | 11 | 5 | 3 | 1 | 5 |
| hsa-miR-1233-5p | AGTGGGAGGCCAGGGCACGG | MIMAT0022943 | hsa-miR-1233-5p | 3 | 5 | 4 | 1 | 0 | 0 | 0 | 0 |
| hsa-miR-1234-3p | TCGGCCTGACCACCCACCCCAC | MIMAT0005589 | hsa-miR-1234-3p | 50 | 90 | 52 | 73 | 60 | 42 | 66 | 65 |
| hsa-miR-1236-3p | CCTCTTCCCCTTGTCTCTCCAG | MIMAT0005591 | hsa-miR-1236-3p | 6 | 3 | 6 | 2 | 1 | 2 | 5 | 1 |
| hsa-miR-1236-5p | GTGAGTGACAGGGGAAA | MIMAT0022945 | hsa-miR-1236-5p | 0 | 0 | 0 | 0 | 0 | 0 | 0 | 0 |
| hsa-miR-1237-3p | TCCTTCTGCTCCGTCCCCC | MIMAT0005592 | hsa-miR-1237-3p | 58 | 77 | 74 | 53 | 44 | 60 | 46 | 44 |
| hsa-miR-1237-5p | TCCCGGGGGCGGGGCCGAAG | MIMAT0022946 | hsa-miR-1237-5p | 14 | 16 | 7 | 7 | 7 | 0 | 0 | 11 |
| hsa-miR-1238-3p | CCTTCCTCGTCTGTCTGCCCCAG | MIMAT0005593 | hsa-miR-1238-3p | 0 | 0 | 0 | 0 | 0 | 1 | 1 | 0 |
| hsa-miR-124-3p | TAAGGCACGCGGTGAATGCC | MIMAT0000422 | hsa-miR-124-3p | 77 | 80 | 111 | 71 | 57 | 29 | 69 | 34 |
| hsa-miR-1243 | AACTGGATCAATTATAGGAGT | MIMAT0005894 | hsa-miR-1243 | 1 | 0 | 3 | 0 | 4 | 1 | 0 | 0 |
| hsa-miR-1244 | GTTGGTTTGTATGAGATGGTT | MIMAT0005896 | hsa-miR-1244 | 0 | 0 | 0 | 1 | 0 | 0 | 0 | 0 |
| hsa-miR-1245a | TTAAGTGATCTAAAGGCCTAC | MIMAT0005897 | hsa-miR-1245a | 0 | 2 | 0 | 0 | 2 | 6 | 8 | 2 |
| hsa-miR-1246 | ATGGATTTTTGGAGCAGGG | MIMAT0005898 | hsa-miR-1246 | 808 | 516 | 129 | 507 | 773 | 880 | 633 | 679 |
| hsa-miR-1247-3p | CGGGAACGTCGAGACTGGAGC | MIMAT0022721 | hsa-miR-1247-3p | 0 | 0 | 0 | 0 | 0 | 0 | 1 | 0 |
| hsa-miR-1247-5p | ACCCGTCCCGTTCGTCCCCGG | MIMAT0005899 | hsa-miR-1247-5p | 2 | 1 | 6 | 2 | 4 | 2 | 7 | 2 |
| hsa-miR-1249-3p | ACGCCCTTCCCCCCCTTCTTC | MIMAT0005901 | hsa-miR-1249-3p | 161 | 458 | 155 | 423 | 771 | 771 | 960 | 591 |
| hsa-miR-1249-5p | AGGAGGGAGGAGATGGGCCAAGTTC | MIMAT0032029 | hsa-miR-1249-5p | 12 | 14 | 14 | 30 | 38 | 24 | 39 | 22 |
| hsa-miR-1250-5p | ACGGTGCTGGATGTGGCCTTT | MIMAT0005902 | hsa-miR-1250-5p | 1 | 0 | 0 | 0 | 1 | 0 | 0 | 2 |
| hsa-miR-1251-5p | ACTCTGGCTGCCAAAGGCGCT | MIMAT0005903 | hsa-miR-1251-5p | 0 | 0 | 1 | 0 | 0 | 0 | 0 | 0 |
| hsa-miR-1254 | AGCCTGGAAGCTGGAGCCTGCAGT | MIMAT0005905 | hsa-miR-1254 | 491 | 984 | 659 | 731 | 998 | 978 | 1655 | 1123 |
| hsa-miR-1255a | AGGATGAGCAAAGAAAGTAGATT | MIMAT0005906 | hsa-miR-1255a | 0 | 0 | 0 | 0 | 0 | 0 | 0 | 0 |
| hsa-miR-1255b-5p | CGGATGAGCAAAGAAAGTGGTT | MIMAT0005945 | hsa-miR-1255b-5p | 2 | 3 | 5 | 1 | 0 | 0 | 0 | 0 |
| hsa-miR-1256 | CCAGGCATTGACTTCTCACT | MIMAT0005907 | hsa-miR-1256 | 0 | 0 | 0 | 0 | 0 | 1 | 0 | 0 |
| hsa-miR-125a-3p | ACAGGTGAGGTTCTTGGGAGCC | MIMAT0004602 | hsa-miR-125a-3p | 381 | 706 | 415 | 590 | 712 | 842 | 1093 | 526 |
| hsa-miR-125a-5p | TCCCTGAGACCCTTTAACCTGTG | MIMAT0000443 | hsa-miR-125a-5p | 57622 | 90199 | 52687 | 82034 | 105449 | 97828 | 107162 | 85039 |
| hsa-miR-125b-1-3p | ACGGGTTAGGCTCTTGGGAGT | MIMAT0004592 | hsa-miR-125b-1-3p | 12984 | 17023 | 10089 | 12686 | 19763 | 17005 | 16772 | 17378 |
| hsa-miR-125b-2-3p | ACAAGTCAGGCTCTTGGGACCT | MIMAT0004603 | hsa-miR-125b-2-3p | 564 | 875 | 548 | 787 | 754 | 938 | 1340 | 919 |
| hsa-miR-125b-5p | TCCCTGAGACCCTAACTTGTG | MIMAT0000423 | hsa-miR-125b-5p | 33653 | 47197 | 26749 | 50855 | 75652 | 79072 | 52594 | 43874 |
| hsa-miR-126-3p | TCGTACCGTGAGTAATAATGCG | MIMAT0000445 | hsa-miR-126-3p | 3223 | 4584 | 2554 | 3734 | 2799 | 2482 | 2485 | 4176 |
| hsa-miR-126-5p | ATTATTACTTTTGGTACGCGCT | MIMAT0000444 | hsa-miR-126-5p | 22 | 32 | 29 | 24 | 12 | 18 | 15 | 20 |
| hsa-miR-1260a | ATCCCACCGCTGCCACCAA | MIMAT0005911 | hsa-miR-1260a | 2955 | 3382 | 3188 | 2763 | 4631 | 4179 | 3615 | 4340 |
| hsa-miR-1260b | ATCCCACCGCTGCCACCA | MIMAT0015041 | hsa-miR-1260b | 17030 | 17855 | 52287 | 17632 | 3366 | 1888 | 2199 | 2957 |
| hsa-miR-1261 | ATGGATAAGGCATTGGCT | MIMAT0005913 | hsa-miR-1261 | 63 | 45 | 10 | 105 | 160 | 150 | 251 | 239 |
| hsa-miR-1262 | TGATGGGTGAATTTGTAGAAGG | MIMAT0005914 | hsa-miR-1262 | 491 | 347 | 499 | 263 | 278 | 161 | 66 | 245 |
| hsa-miR-1265 | CAGGATGTGGTCAAGTGTTGTT | MIMAT0005918 | hsa-miR-1265 | 9 | 7 | 6 | 8 | 7 | 4 | 0 | 2 |
| hsa-miR-1266-5p | CCTCAGGGCTGTAGAACAGGGCTT | MIMAT0005920 | hsa-miR-1266-5p | 0 | 0 | 0 | 2 | 0 | 0 | 1 | 0 |
| hsa-miR-1267 | CTGTTGAAGTGTAATCCCCACCTC | MIMAT0005921 | hsa-miR-1267 | 0 | 0 | 0 | 0 | 0 | 0 | 0 | 0 |
| hsa-miR-1268a | CGGGCGTGGTGGTGGGGG | MIMAT0005922 | hsa-miR-1268a | 10632 | 6319 | 8729 | 3893 | 5163 | 6166 | 8649 | 8063 |
| hsa-miR-1269a | CCTGGACTGAGCCATGCTACTG | MIMAT0005923 | hsa-miR-1269a | 1 | 0 | 1 | 0 | 4 | 4 | 9 | 4 |
| hsa-miR-1269b | CTGGACTGAGCCATGCTAC | MIMAT0019059 | hsa-miR-1269b | 79 | 182 | 87 | 112 | 160 | 147 | 222 | 126 |
| hsa-miR-127-3p | TCGGATCCGTCTGAGCTTGGCT | MIMAT0000446 | hsa-miR-127-3p | 371952 | 484833 | 415175 | 424952 | 934248 | 971700 | 2310297 | 1066295 |
| hsa-miR-127-5p | CTGAAGCTCAGAGGGCTCTGAT | MIMAT0004604 | hsa-miR-127-5p | 1 | 4 | 6 | 6 | 3 | 4 | 0 | 4 |
| hsa-miR-1270 | CTGGAGATATGGAAGAGCTGTGT | MIMAT0005924 | hsa-miR-1270 | 61 | 91 | 45 | 104 | 110 | 108 | 73 | 127 |
| hsa-miR-1271-3p | AGTGCCTGCTATGTGCCAGG | MIMAT0022712 | hsa-miR-1271-3p | 9 | 4 | 7 | 3 | 5 | 7 | 17 | 6 |
| hsa-miR-1271-5p | CTTGGCACCTAGCAAGCACTC | MIMAT0005796 | hsa-miR-1271-5p | 1774 | 2439 | 1606 | 2263 | 2659 | 2003 | 3699 | 2277 |
| hsa-miR-1273a | CTGGGCGACAGAGCAAGACTC | MIMAT0005926 | hsa-miR-1273a | 0 | 0 | 0 | 0 | 0 | 4 | 0 | 0 |
| hsa-miR-1273c | GGCGACAAAACGAGACCCTGTC | MIMAT0015017 | hsa-miR-1273c | 20 | 26 | 29 | 21 | 33 | 37 | 63 | 54 |
| hsa-miR-1273d | GAACCCATGAGGTTGAGGCTGCAGT | MIMAT0015090 | hsa-miR-1273d | 14 | 18 | 14 | 13 | 10 | 15 | 9 | 31 |
| hsa-miR-1273e | TGCTTGAACCCAGGAGGTGGAGG | MIMAT0018079 | hsa-miR-1273e | 1 | 11 | 4 | 1 | 3 | 2 | 0 | 0 |
| hsa-miR-1273f | GGAGACGGAGGTTGCAGTGAGC | NA | hsa-miR-1273f | 9 | 35 | 19 | 33 | 15 | 23 | 0 | 9 |
| hsa-miR-1273g-3p | CACTGCACTCCAGCCTGGG | NA | hsa-miR-1273g-3p | 30 | 24 | 49 | 32 | 13 | 2 | 0 | 26 |
| hsa-miR-1273g-5p | GGGAGGTTGAGGCTGCAGT | NA | hsa-miR-1273g-5p | 0 | 0 | 0 | 0 | 0 | 1 | 0 | 0 |
| hsa-miR-1273h-3p | CAGCCTCGACCTCCCAGG | MIMAT0030416 | hsa-miR-1273h-3p | 10 | 0 | 0 | 0 | 0 | 0 | 0 | 0 |
| hsa-miR-1273h-5p | CTGGGAGGTCGAGGCTGC | MIMAT0030415 | hsa-miR-1273h-5p | 0 | 4 | 0 | 0 | 11 | 10 | 17 | 11 |
| hsa-miR-1275 | GTGGGGGAGAGGCTGTCG | MIMAT0005929 | hsa-miR-1275 | 1005 | 1439 | 854 | 634 | 1578 | 1506 | 3613 | 2326 |
| hsa-miR-1276 | TAAAGAGCCCTGTGGAGACACC | MIMAT0005930 | hsa-miR-1276 | 10 | 37 | 21 | 11 | 19 | 18 | 33 | 21 |
| hsa-miR-1277-3p | TACGTAGATATATATGTAT | MIMAT0005933 | hsa-miR-1277-3p | 0 | 0 | 0 | 0 | 0 | 0 | 0 | 0 |
| hsa-miR-1277-5p | TATATATATATATGTACGTAT | MIMAT0022724 | hsa-miR-1277-5p | 1 | 1 | 1 | 1 | 0 | 0 | 0 | 0 |
| hsa-miR-1278 | TAGTACTGTGCATATCATC | MIMAT0005936 | hsa-miR-1278 | 0 | 0 | 0 | 0 | 0 | 0 | 0 | 2 |
| hsa-miR-128-1-5p | CGGGGCCGTAGCACTGTCTG | MIMAT0026477 | hsa-miR-128-1-5p | 100 | 108 | 79 | 70 | 170 | 173 | 436 | 188 |
| hsa-miR-128-2-5p | GGGGGCCGATACACTGTACG | MIMAT0031095 | hsa-miR-128-2-5p | 547 | 712 | 825 | 561 | 719 | 580 | 1003 | 908 |
| hsa-miR-128-3p | TCACAGTGAACCGGTCTCTTT | MIMAT0000424 | hsa-miR-128-3p | 1676 | 3391 | 1494 | 2426 | 2332 | 1820 | 1729 | 2220 |
| hsa-miR-1284 | TCTATACAGACCCTGGCTTTTC | MIMAT0005941 | hsa-miR-1284 | 18 | 11 | 14 | 14 | 17 | 9 | 16 | 14 |
| hsa-miR-1285-3p | TCTGGGCAACAAAGTGAGAC | MIMAT0005876 | hsa-miR-1285-3p | 5 | 14 | 15 | 6 | 2 | 7 | 0 | 11 |
| hsa-miR-1285-5p | ATCTCACTTTGTTGCCCAGGCT | MIMAT0022719 | hsa-miR-1285-5p | 648 | 469 | 560 | 392 | 334 | 285 | 313 | 348 |
| hsa-miR-1287-3p | CTCTAGCCACAGATGCAGTGAT | MIMAT0026738 | hsa-miR-1287-3p | 10 | 11 | 8 | 12 | 2 | 4 | 0 | 4 |
| hsa-miR-1287-5p | TGCTGGATCAGTGGTTCGAGT | MIMAT0005878 | hsa-miR-1287-5p | 259 | 371 | 262 | 352 | 768 | 732 | 1787 | 738 |
| hsa-miR-1288-3p | TGGACTGCCCTGATCTGG | MIMAT0005942 | hsa-miR-1288-3p | 4 | 7 | 7 | 9 | 24 | 26 | 59 | 23 |
| hsa-miR-1289 | GGTGGAGTCCAGGAATCTGCATT | MIMAT0005879 | hsa-miR-1289 | 0 | 0 | 0 | 0 | 2 | 0 | 0 | 0 |
| hsa-miR-129-2-3p | AAGCCCTTACCCCAAAAAGC | MIMAT0004605 | hsa-miR-129-2-3p | 3 | 0 | 1 | 0 | 0 | 2 | 0 | 6 |
| hsa-miR-129-5p | CTTTTTGCGGTCTGGGCTTGC | MIMAT0000242 | hsa-miR-129-5p | 1008 | 2038 | 878 | 978 | 1946 | 1917 | 3815 | 2345 |
| hsa-miR-1290 | TGGATTTTTGGAGCAGGGA | MIMAT0005880 | hsa-miR-1290 | 25 | 25 | 84 | 25 | 1 | 0 | 0 | 0 |
| hsa-miR-1291 | GTGGCCCTGACTGAAGACCAGCAGTTGT | MIMAT0005881 | hsa-miR-1291 | 3 | 13 | 3 | 5 | 8 | 16 | 11 | 7 |
| hsa-miR-1292-3p | CGCGCCCCGGCTCCCGTTCCAGT | MIMAT0022948 | hsa-miR-1292-3p | 31 | 44 | 34 | 29 | 43 | 48 | 41 | 50 |
| hsa-miR-1292-5p | TGGGAACGGGTTCCGGCAGACGCTG | MIMAT0005943 | hsa-miR-1292-5p | 836 | 2003 | 1079 | 1389 | 1577 | 1520 | 2313 | 2138 |
| hsa-miR-1293 | TGGGTGGTCTGGAGATTTGTGC | MIMAT0005883 | hsa-miR-1293 | 790 | 1209 | 730 | 800 | 1666 | 1585 | 2780 | 1603 |
| hsa-miR-1294 | TGTGAGGTTGGCATTGTTGTCT | MIMAT0005884 | hsa-miR-1294 | 246 | 421 | 263 | 326 | 602 | 608 | 1075 | 659 |
| hsa-miR-1295a | TTAGGCCGCAGATCTGGGTGA | MIMAT0005885 | hsa-miR-1295a | 1 | 1 | 4 | 1 | 0 | 0 | 0 | 0 |
| hsa-miR-1296-3p | GGGAGTGGGGCTTCGACCCTT | MIMAT0026637 | hsa-miR-1296-3p | 0 | 3 | 0 | 0 | 0 | 0 | 0 | 0 |
| hsa-miR-1296-5p | TTAGGGCCCTGGCTCCATCTCC | MIMAT0005794 | hsa-miR-1296-5p | 3533 | 10606 | 2892 | 5937 | 9158 | 7932 | 16365 | 8236 |
| hsa-miR-1298-5p | TTCATTCGGCTGTCCAGATGT | MIMAT0005800 | hsa-miR-1298-5p | 0 | 1 | 1 | 0 | 0 | 0 | 0 | 1 |
| hsa-miR-1299 | TTCTGGAATTCTGTGTGAGGG | MIMAT0005887 | hsa-miR-1299 | 0 | 0 | 2 | 0 | 0 | 0 | 0 | 0 |
| hsa-miR-1301-3p | TTGCAGCTGCCTGGGAGTGACT | MIMAT0005797 | hsa-miR-1301-3p | 5087 | 11832 | 5371 | 8515 | 13655 | 12449 | 22088 | 13494 |
| hsa-miR-1301-5p | GGGTCGCTCTAGGCACCGCAGC | MIMAT0026639 | hsa-miR-1301-5p | 0 | 0 | 0 | 2 | 4 | 3 | 0 | 0 |
| hsa-miR-1302 | TTGGGACATACTTATGCTAAA | MIMAT0005890 | hsa-miR-1302 | 2 | 0 | 0 | 3 | 4 | 0 | 0 | 5 |
| hsa-miR-1303 | TTTTAGAGACGGGGTCTTGCTCT | MIMAT0005891 | hsa-miR-1303 | 591 | 1541 | 652 | 1090 | 1581 | 1447 | 2911 | 1725 |
| hsa-miR-1304-3p | TCTCACTGTAGCATCGAACCCC | MIMAT0022720 | hsa-miR-1304-3p | 561 | 867 | 438 | 494 | 440 | 370 | 440 | 554 |
| hsa-miR-1304-5p | CGGTTTGAGGCTACAGTG | MIMAT0005892 | hsa-miR-1304-5p | 1018 | 1520 | 956 | 1001 | 1020 | 981 | 649 | 1030 |
| hsa-miR-1306-3p | GACGTTGGCTCTGGTGGTGAT | MIMAT0005950 | hsa-miR-1306-3p | 574 | 777 | 709 | 577 | 929 | 997 | 1805 | 1133 |
| hsa-miR-1306-5p | CCACCTCCCCTGCAAACGTCC | MIMAT0022726 | hsa-miR-1306-5p | 163 | 368 | 180 | 241 | 315 | 275 | 179 | 314 |
| hsa-miR-1307-3p | CTCGGCGTGGCGTCGGTCG | MIMAT0005951 | hsa-miR-1307-3p | 98982 | 100503 | 98383 | 79400 | 126123 | 118447 | 289932 | 146143 |
| hsa-miR-1307-5p | TCGACCGGACCTCGACCGGCTCG | MIMAT0022727 | hsa-miR-1307-5p | 7 | 30 | 10 | 34 | 25 | 12 | 25 | 11 |
| hsa-miR-130a-3p | CAGTGCAATGTTAAAAGGGC | MIMAT0000425 | hsa-miR-130a-3p | 512 | 479 | 438 | 498 | 501 | 382 | 354 | 580 |
| hsa-miR-130a-5p | GCUCUUUUCACAUUGUGCUACU | MIMAT0004593 | hsa-miR-130a-5p | 0 | 0 | 0 | 0 | 0 | 0 | 0 | 0 |
| hsa-miR-130b-3p | CAGTGCAATGATGAAAGGGC | MIMAT0000691 | hsa-miR-130b-3p | 93 | 57 | 55 | 97 | 95 | 97 | 37 | 69 |
| hsa-miR-130b-5p | ACTCTTTCCCTGTTGCACTACT | MIMAT0004680 | hsa-miR-130b-5p | 4267 | 6803 | 4526 | 4905 | 4955 | 3776 | 3457 | 4245 |
| hsa-miR-132-3p | TAACAGTCTACAGCCAAGG | MIMAT0000426 | hsa-miR-132-3p | 42 | 47 | 47 | 65 | 735 | 238 | 595 | 227 |
| hsa-miR-132-5p | ACCGTGGCTTTCGATTGTTACT | MIMAT0004594 | hsa-miR-132-5p | 3783 | 6074 | 3979 | 5103 | 6626 | 6128 | 10574 | 6870 |
| hsa-miR-1322 | GATGATGCTGCTGATGATGT | MIMAT0005953 | hsa-miR-1322 | 0 | 0 | 2 | 0 | 0 | 0 | 0 | 0 |
| hsa-miR-1323 | TCAAAACTGAGGGGCATTTTCT | MIMAT0005795 | hsa-miR-1323 | 0 | 0 | 0 | 2 | 0 | 0 | 0 | 0 |
| hsa-miR-133a-3p | TTGGTCCCCTTCAACCAGCTGT | MIMAT0000427 | hsa-miR-133a-3p | 27 | 175 | 14 | 23 | 16 | 22 | 33 | 24 |
| hsa-miR-133b | TTGGTCCCCTTCAACCAGC | MIMAT0000770 | hsa-miR-133b | 0 | 0 | 2 | 0 | 0 | 0 | 0 | 0 |
| hsa-miR-134-3p | CTGTGGGCCACCTAGTCACC | MIMAT0026481 | hsa-miR-134-3p | 405 | 754 | 379 | 623 | 747 | 672 | 885 | 614 |
| hsa-miR-134-5p | TGTGACTGGTTGACCAGAGGGG | MIMAT0000447 | hsa-miR-134-5p | 14889 | 10553 | 12463 | 8699 | 14503 | 12642 | 29168 | 12140 |
| hsa-miR-1343-3p | CTCCTGGGGCCCGCACTCTCG | MIMAT0019776 | hsa-miR-1343-3p | 460 | 978 | 428 | 556 | 783 | 648 | 1004 | 864 |
| hsa-miR-1343-5p | TGGGGAGCGGCCCCCGGGCGGG | MIMAT0027038 | hsa-miR-1343-5p | 133 | 123 | 186 | 81 | 47 | 57 | 78 | 75 |
| hsa-miR-135a-3p | ATATAGGGATTGGAGCCG | MIMAT0004595 | hsa-miR-135a-3p | 0 | 0 | 0 | 1 | 0 | 0 | 0 | 0 |
| hsa-miR-135a-5p | TATGGCTTTTTATTCCTATGTG | MIMAT0000428 | hsa-miR-135a-5p | 2 | 0 | 2 | 0 | 5 | 7 | 17 | 5 |
| hsa-miR-135b-3p | TGTAGGGCTAAAAGCCATGGG | MIMAT0004698 | hsa-miR-135b-3p | 3 | 3 | 10 | 3 | 0 | 0 | 0 | 0 |
| hsa-miR-135b-5p | TATGGCTTTTCATTCCTATGTG | MIMAT0000758 | hsa-miR-135b-5p | 51 | 38 | 57 | 43 | 32 | 19 | 15 | 12 |
| hsa-miR-136-3p | ATCATCGTCTCAAATGAGTCT | MIMAT0004606 | hsa-miR-136-3p | 5 | 16 | 12 | 7 | 19 | 18 | 40 | 16 |
| hsa-miR-136-5p | AGGACTCCATTTGTTTTGATGAT | MIMAT0000448 | hsa-miR-136-5p | 27 | 40 | 27 | 41 | 34 | 15 | 18 | 31 |
| hsa-miR-137 | TATTGCTTAAGAATACGCGTAGT | MIMAT0000429 | hsa-miR-137 | 44 | 116 | 55 | 64 | 81 | 55 | 70 | 81 |
| hsa-miR-138-1-3p | GCTACTTCACAACACCAGGGC | MIMAT0004607 | hsa-miR-138-1-3p | 365 | 113 | 248 | 101 | 80 | 62 | 24 | 219 |
| hsa-miR-138-2-3p | GCTATTTCACGACACCAGGGTT | MIMAT0004596 | hsa-miR-138-2-3p | 10 | 1 | 0 | 2 | 2 | 0 | 0 | 1 |
| hsa-miR-138-5p | AGCTGGTGTTGTGAATCAGGCCGT | MIMAT0000430 | hsa-miR-138-5p | 3347 | 6563 | 3532 | 6927 | 13568 | 12427 | 25891 | 11328 |
| hsa-miR-139-3p | TGGAGACGCGGCCCTGTTGGAGT | MIMAT0004552 | hsa-miR-139-3p | 1013 | 1749 | 987 | 730 | 1157 | 1119 | 1958 | 1437 |
| hsa-miR-139-5p | TCTACAGTGCACGTGTCTCCAG | MIMAT0000250 | hsa-miR-139-5p | 2006 | 1508 | 1247 | 2537 | 3699 | 4700 | 3461 | 2614 |
| hsa-miR-140-3p | ACCACAGGGTAGAACCACGGAC | MIMAT0004597 | hsa-miR-140-3p | 747 | 674 | 741 | 888 | 773 | 598 | 552 | 483 |
| hsa-miR-140-5p | CAGTGGTTTTACCCTATGGT | MIMAT0000431 | hsa-miR-140-5p | 362 | 518 | 395 | 450 | 550 | 496 | 368 | 354 |
| hsa-miR-141-3p | TAACACTGTCTGGTAAAGA | MIMAT0000432 | hsa-miR-141-3p | 18 | 10 | 22 | 12 | 50 | 50 | 170 | 80 |
| hsa-miR-141-5p | CATCTTCCAGTACAGTGT | MIMAT0004598 | hsa-miR-141-5p | 0 | 0 | 2 | 1 | 0 | 0 | 3 | 0 |
| hsa-miR-142-3p | TGTAGTGTTTCCTACTTTA | MIMAT0000434 | hsa-miR-142-3p | 1 | 0 | 2 | 0 | 0 | 9 | 0 | 2 |
| hsa-miR-142-5p | CCCATAAAGTAGAAAGCACT | MIMAT0000433 | hsa-miR-142-5p | 0 | 3 | 2 | 2 | 1 | 17 | 1 | 0 |
| hsa-miR-143-3p | TGAGATGAAGCACTGTAGCTC | MIMAT0000435 | hsa-miR-143-3p | 11495 | 16974 | 12265 | 14004 | 21950 | 19092 | 37266 | 21966 |
| hsa-miR-143-5p | GGTGCAGTGCTGCATCTCTGGTC | MIMAT0004599 | hsa-miR-143-5p | 11 | 15 | 4 | 11 | 15 | 28 | 31 | 27 |
| hsa-miR-144-3p | TACAGTATAGATGATGTACT | MIMAT0000436 | hsa-miR-144-3p | 1 | 1 | 1 | 0 | 0 | 4 | 0 | 2 |
| hsa-miR-144-5p | GGATATCATCATATACTGTAAGT | MIMAT0004600 | hsa-miR-144-5p | 0 | 0 | 1 | 0 | 0 | 0 | 0 | 0 |
| hsa-miR-145-3p | GGATTCCTGGAAATACTGTTCT | MIMAT0004601 | hsa-miR-145-3p | 122 | 225 | 158 | 206 | 255 | 216 | 360 | 241 |
| hsa-miR-145-5p | GTCCAGTTTTCCCAGGAATCCCT | MIMAT0000437 | hsa-miR-145-5p | 1363 | 1363 | 1219 | 1219 | 4165 | 4182 | 3251 | 3171 |
| hsa-miR-1468-5p | CTCCGTTTGCCTGTTTCGCTG | MIMAT0006789 | hsa-miR-1468-5p | 181 | 252 | 177 | 268 | 523 | 536 | 1225 | 503 |
| hsa-miR-1469 | CGCGGGGCGCGGGCGCCGGGT | MIMAT0007347 | hsa-miR-1469 | 0 | 0 | 0 | 0 | 0 | 0 | 0 | 0 |
| hsa-miR-146a-3p | GACCTCTGAAATTCAGTTCTTC | MIMAT0004608 | hsa-miR-146a-3p | 0 | 0 | 2 | 0 | 0 | 0 | 0 | 0 |
| hsa-miR-146a-5p | TGAGAACTGAATTCCATGGGTT | MIMAT0000449 | hsa-miR-146a-5p | 4183 | 6567 | 4470 | 6490 | 7848 | 6746 | 8715 | 6802 |
| hsa-miR-146b-3p | TGCCCTGTGGACTCAGTTC | MIMAT0004766 | hsa-miR-146b-3p | 21 | 71 | 32 | 32 | 47 | 52 | 80 | 33 |
| hsa-miR-146b-5p | TGAGAACTGAATTCCATAGGCTGT | MIMAT0002809 | hsa-miR-146b-5p | 549 | 849 | 516 | 750 | 770 | 510 | 336 | 604 |
| hsa-miR-147b | GTGTGCGGAAATGCTTCTG | MIMAT0004928 | hsa-miR-147b | 0 | 0 | 0 | 0 | 2 | 0 | 0 | 0 |
| hsa-miR-148a-3p | TCAGTGCACTACAGAACTTTGTC | MIMAT0000243 | hsa-miR-148a-3p | 20 | 33 | 18 | 44 | 39 | 31 | 21 | 35 |
| hsa-miR-148a-5p | AAAGTTCTGAGACACTCCG | MIMAT0004549 | hsa-miR-148a-5p | 2 | 0 | 0 | 0 | 4 | 4 | 14 | 4 |
| hsa-miR-148b-3p | TCAGTGCATCACAGAACTTTGTT | MIMAT0000759 | hsa-miR-148b-3p | 211 | 399 | 236 | 229 | 437 | 277 | 321 | 353 |
| hsa-miR-148b-5p | GAAGTTCTGTTATACACTCAGG | MIMAT0004699 | hsa-miR-148b-5p | 0 | 3 | 3 | 2 | 0 | 0 | 0 | 2 |
| hsa-miR-149-3p | GAGGGAGGGACGGGGGCTGTGC | MIMAT0004609 | hsa-miR-149-3p | 20 | 19 | 20 | 19 | 52 | 58 | 93 | 46 |
| hsa-miR-149-5p | TCTGGCTCCGTGTCTTCACTCCC | MIMAT0000450 | hsa-miR-149-5p | 38499 | 92402 | 41048 | 68457 | 80712 | 72245 | 118424 | 72142 |
| hsa-miR-150-3p | CTGGTACAGGCCTGGGGGAC | MIMAT0004610 | hsa-miR-150-3p | 0 | 0 | 0 | 0 | 0 | 0 | 0 | 0 |
| hsa-miR-150-5p | TCTCCCAACCCTTGTACCAGTG | MIMAT0000451 | hsa-miR-150-5p | 11 | 7 | 3 | 3 | 57 | 82 | 25 | 72 |
| hsa-miR-151a-3p | CTAGACTGAAGCTCCTTGAGG | MIMAT0000757 | hsa-miR-151a-3p | 1652 | 1156 | 1284 | 1482 | 1779 | 1554 | 2040 | 1424 |
| hsa-miR-151a-5p | TCGAGGAGCTCACAGTCTAGT | MIMAT0004697 | hsa-miR-151a-5p | 55293 | 79795 | 54904 | 67759 | 55737 | 42565 | 37775 | 52659 |
| hsa-miR-152-3p | TCAGTGCATGACAGAACTTGGG | MIMAT0000438 | hsa-miR-152-3p | 772 | 968 | 663 | 957 | 1048 | 862 | 640 | 661 |
| hsa-miR-152-5p | CAGGTTCTGTGATACACTCCGACT | MIMAT0026479 | hsa-miR-152-5p | 0 | 2 | 0 | 8 | 1 | 2 | 0 | 0 |
| hsa-miR-153-3p | TTGCATAGTCACAAAAGTGATC | MIMAT0000439 | hsa-miR-153-3p | 14 | 17 | 19 | 5 | 0 | 2 | 0 | 0 |
| hsa-miR-153-5p | GTCATTTTTGTGATGTTGCAGCTT | MIMAT0026480 | hsa-miR-153-5p | 0 | 0 | 0 | 0 | 0 | 0 | 0 | 0 |
| hsa-miR-1537-3p | AAACCGTCTAGTTACAGTTGT | MIMAT0007399 | hsa-miR-1537-3p | 1 | 0 | 0 | 0 | 0 | 4 | 0 | 0 |
| hsa-miR-1537-5p | AGCTGTAATTAGTCAGTTTTCT | MIMAT0026765 | hsa-miR-1537-5p | 0 | 0 | 0 | 0 | 1 | 0 | 0 | 0 |
| hsa-miR-1538 | CGGCCCGGGCTGCTGCTGTTC | MIMAT0007400 | hsa-miR-1538 | 86 | 154 | 100 | 124 | 246 | 270 | 611 | 298 |
| hsa-miR-154-3p | AATCATACACGGTTGACCTATT | MIMAT0000453 | hsa-miR-154-3p | 57 | 112 | 63 | 79 | 58 | 42 | 19 | 96 |
| hsa-miR-154-5p | TAGGTTATCCGTGTTGCCTTCG | MIMAT0000452 | hsa-miR-154-5p | 1445 | 1802 | 1798 | 1739 | 2655 | 2508 | 5524 | 2318 |
| hsa-miR-155-3p | CTCCTACATATTAGCATTAAC | MIMAT0004658 | hsa-miR-155-3p | 0 | 0 | 0 | 0 | 0 | 0 | 0 | 0 |
| hsa-miR-155-5p | TTAATGCTAATCGTGATAGGGGTT | MIMAT0000646 | hsa-miR-155-5p | 87924 | 145520 | 103628 | 97631 | 87574 | 57500 | 26968 | 120898 |
| hsa-miR-1587 | TGGGCTGGGCTGGGTTGGG | MIMAT0019077 | hsa-miR-1587 | 0 | 0 | 0 | 0 | 3 | 0 | 1 | 1 |
| hsa-miR-15a-3p | CAGGCCATATTGTGCTGCCTC | MIMAT0004488 | hsa-miR-15a-3p | 2 | 4 | 0 | 4 | 2 | 0 | 0 | 0 |
| hsa-miR-15a-5p | TAGCAGCACATAATGGTTTGT | MIMAT0000068 | hsa-miR-15a-5p | 139 | 218 | 124 | 180 | 107 | 107 | 49 | 108 |
| hsa-miR-15b-3p | CGAATCATTATTTGCTGCTCT | MIMAT0004586 | hsa-miR-15b-3p | 165 | 189 | 180 | 149 | 79 | 76 | 44 | 119 |
| hsa-miR-15b-5p | TAGCAGCACATCATGGTTTAC | MIMAT0000417 | hsa-miR-15b-5p | 17001 | 24631 | 14918 | 24053 | 18278 | 12468 | 3973 | 16403 |
| hsa-miR-16-1-3p | CTCCAGTATTAACTGTGCTGCTG | MIMAT0004489 | hsa-miR-16-1-3p | 0 | 0 | 0 | 0 | 0 | 0 | 0 | 0 |
| hsa-miR-16-2-3p | ACCAATATTACTGTGCTGCTT | MIMAT0004518 | hsa-miR-16-2-3p | 32 | 46 | 38 | 31 | 36 | 45 | 61 | 56 |
| hsa-miR-16-5p | TAGCAGCACGTAAATATTGGCG | MIMAT0000069 | hsa-miR-16-5p | 5893 | 8007 | 5963 | 6408 | 4296 | 3720 | 2486 | 5714 |
| hsa-miR-17-3p | ACTGCAGTGAAGGCACTTGTAGC | MIMAT0000071 | hsa-miR-17-3p | 24 | 64 | 33 | 36 | 59 | 69 | 128 | 67 |
| hsa-miR-17-5p | CAAAGTGCTTACAGTGCAGGTAGT | MIMAT0000070 | hsa-miR-17-5p | 153 | 232 | 128 | 210 | 214 | 171 | 116 | 152 |
| hsa-miR-181a-2-3p | ACCACTGACCGTTGACTGTACC | MIMAT0004558 | hsa-miR-181a-2-3p | 4936 | 7081 | 5394 | 6207 | 7652 | 6309 | 9594 | 7176 |
| hsa-miR-181a-3p | ACCATCGACCGTTGATTGTACC | MIMAT0000270 | hsa-miR-181a-3p | 589 | 883 | 694 | 714 | 1198 | 1070 | 2395 | 1105 |
| hsa-miR-181a-5p | AACATTCAACGCTGTCGGTGAGT | MIMAT0000256 | hsa-miR-181a-5p | 716500 | 1327358 | 778597 | 1461083 | 1989884 | 1743989 | 2713311 | 1458481 |
| hsa-miR-181b-2-3p | CTCACTGATCAATGAATGC | MIMAT0031893 | hsa-miR-181b-2-3p | 0 | 0 | 3 | 0 | 0 | 0 | 0 | 0 |
| hsa-miR-181b-3p | CUCACUGAACAAUGAAUGCAA | MIMAT0022692 | hsa-miR-181b-3p | 0 | 0 | 0 | 0 | 0 | 0 | 0 | 0 |
| hsa-miR-181b-5p | AACATTCATTGCTGTCGGTGGGT | MIMAT0000257 | hsa-miR-181b-5p | 113538 | 168048 | 127354 | 140070 | 155265 | 142839 | 121240 | 157204 |
| hsa-miR-181c-3p | ACCATCGACCGTTGAGTGGACC | MIMAT0004559 | hsa-miR-181c-3p | 6 | 18 | 8 | 17 | 15 | 15 | 41 | 13 |
| hsa-miR-181c-5p | AACATTCAACCTGTCGGTGAGT | MIMAT0000258 | hsa-miR-181c-5p | 159 | 167 | 186 | 319 | 389 | 369 | 749 | 333 |
| hsa-miR-181d-3p | CCACCGGGGGATGAATGTC | MIMAT0026608 | hsa-miR-181d-3p | 2 | 1 | 5 | 1 | 0 | 0 | 0 | 0 |
| hsa-miR-181d-5p | AACATTCATTGTTGTCGGTGGGTT | MIMAT0002821 | hsa-miR-181d-5p | 535 | 1008 | 707 | 955 | 1009 | 1053 | 1093 | 969 |
| hsa-miR-182-3p | UGGUUCUAGACUUGCCAACUA | MIMAT0000260 | hsa-miR-182-3p | 0 | 0 | 1 | 0 | 0 | 0 | 1 | 0 |
| hsa-miR-182-5p | TTTGGCAATGGTAGAACTCACAC | MIMAT0000259 | hsa-miR-182-5p | 144 | 92 | 168 | 119 | 88 | 206 | 179 | 115 |
| hsa-miR-1827 | TGAGGCAGTAGATTGTAT | MIMAT0006767 | hsa-miR-1827 | 0 | 0 | 0 | 0 | 0 | 0 | 0 | 0 |
| hsa-miR-183-3p | TGAATTACCGAAGGGCCATAA | MIMAT0004560 | hsa-miR-183-3p | 3 | 3 | 4 | 4 | 0 | 5 | 2 | 1 |
| hsa-miR-183-5p | TATGGCACTGGTAGAATTCACT | MIMAT0000261 | hsa-miR-183-5p | 95 | 78 | 113 | 124 | 76 | 87 | 76 | 76 |
| hsa-miR-184 | TGGACGGAGAACTGATAAGGGT | MIMAT0000454 | hsa-miR-184 | 301 | 385 | 283 | 241 | 314 | 271 | 208 | 352 |
| hsa-miR-185-3p | AGGGGCTGGCTTTCCTCTGGT | MIMAT0004611 | hsa-miR-185-3p | 30 | 97 | 45 | 51 | 150 | 115 | 204 | 103 |
| hsa-miR-185-5p | TGGAGAGAAAGGCAGTTCCTG | MIMAT0000455 | hsa-miR-185-5p | 765 | 1227 | 719 | 964 | 1504 | 1437 | 2361 | 1555 |
| hsa-miR-186-5p | CAAAGAATTCTCCTTTTGGGCTT | MIMAT0000456 | hsa-miR-186-5p | 84 | 146 | 59 | 122 | 196 | 254 | 227 | 179 |
| hsa-miR-187-3p | TCGTGTCTTGTGTTGCAGCCGGT | MIMAT0000262 | hsa-miR-187-3p | 26 | 26 | 15 | 31 | 22 | 16 | 39 | 29 |
| hsa-miR-187-5p | GCTACAACACAGGACCCGGGCG | MIMAT0004561 | hsa-miR-187-5p | 4 | 4 | 0 | 0 | 2 | 0 | 0 | 2 |
| hsa-miR-188-3p | CTCCCACATGCAGGGTTTGC | MIMAT0004613 | hsa-miR-188-3p | 0 | 3 | 3 | 6 | 2 | 0 | 0 | 0 |
| hsa-miR-188-5p | CATCCCTTGCATGGTGGAGGG | MIMAT0000457 | hsa-miR-188-5p | 103 | 97 | 61 | 73 | 71 | 60 | 42 | 48 |
| hsa-miR-18b-3p | ACTGCCCTAAATGCCCCTTC | MIMAT0004751 | hsa-miR-18a-3p | 57 | 100 | 31 | 33 | 76 | 87 | 92 | 81 |
| hsa-miR-18b-5p | TAAGGTGCATCTAGTGCAGTT | MIMAT0001412 | hsa-miR-18a-5p | 14 | 43 | 20 | 37 | 33 | 27 | 45 | 21 |
| hsa-miR-18b-3p | UGCCCUAAAUGCCCCUUCUGGC | MIMAT0004751 | hsa-miR-18b-3p | 0 | 0 | 0 | 0 | 0 | 0 | 0 | 0 |
| hsa-miR-18b-5p | UAAGGUGCAUCUAGUGCAGUUAG | MIMAT0001412 | hsa-miR-18b-5p | 0 | 0 | 1 | 2 | 0 | 1 | 0 | 0 |
| hsa-miR-1908-3p | CCGGCCGCCGGCTCCGCCCCG | MIMAT0026916 | hsa-miR-1908-3p | 38 | 60 | 31 | 72 | 163 | 155 | 316 | 136 |
| hsa-miR-1908-5p | CGGCGGGGACGGCGATTGGTC | MIMAT0007881 | hsa-miR-1908-5p | 3896 | 5784 | 4300 | 7482 | 16438 | 15193 | 29967 | 15288 |
| hsa-miR-1909-3p | CGCAGGGGCCGGGTGCTCACCG | MIMAT0007883 | hsa-miR-1909-3p | 3 | 11 | 8 | 16 | 9 | 9 | 12 | 11 |
| hsa-miR-1909-5p | GTGAGTGCCGGTGCCTGCCCTGT | MIMAT0007882 | hsa-miR-1909-5p | 24 | 33 | 10 | 17 | 35 | 22 | 66 | 33 |
| hsa-miR-190a-3p | CUAUAUAUCAAACAUAUUCCU | MIMAT0026482 | hsa-miR-190a-3p | 0 | 0 | 0 | 0 | 0 | 0 | 0 | 0 |
| hsa-miR-190a-5p | TGATATGTTTGATATATTAGGTTG | MIMAT0000458 | hsa-miR-190a-5p | 22 | 27 | 31 | 33 | 44 | 46 | 52 | 40 |
| hsa-miR-190b | TGATATGTTTGATATTGGGTTG | MIMAT0037332 | hsa-miR-190b | 86 | 92 | 85 | 130 | 131 | 108 | 110 | 107 |
| hsa-miR-191-3p | GCTGCGCTTGGATTTCGTCCC | MIMAT0001618 | hsa-miR-191-3p | 1325 | 2123 | 1248 | 1235 | 2224 | 2208 | 4899 | 2547 |
| hsa-miR-191-5p | CAACGGAATCCCAAAAGCAGCTG | MIMAT0000440 | hsa-miR-191-5p | 183343 | 340495 | 182074 | 256021 | 228578 | 156757 | 91222 | 231648 |
| hsa-miR-1910-3p | GAGGCAGAAGCAGGATGAC | MIMAT0026917 | hsa-miR-1910-3p | 11 | 4 | 0 | 7 | 2 | 4 | 0 | 0 |
| hsa-miR-1910-5p | CCAGTCCTGTGCCTGCCGCCT | MIMAT0007884 | hsa-miR-1910-5p | 515 | 1046 | 371 | 409 | 542 | 576 | 805 | 871 |
| hsa-miR-1913 | TCTGCCCCCTCCGCTGCTGCC | MIMAT0007888 | hsa-miR-1913 | 70 | 168 | 61 | 70 | 140 | 156 | 159 | 129 |
| hsa-miR-1914-3p | AGGAGGGGTCCCGCACTGGGAGT | MIMAT0007890 | hsa-miR-1914-3p | 34 | 43 | 11 | 18 | 31 | 42 | 51 | 38 |
| hsa-miR-1914-5p | CCCTGTGCCCGGCCCACTTCTG | MIMAT0007889 | hsa-miR-1914-5p | 43 | 103 | 41 | 26 | 45 | 48 | 74 | 78 |
| hsa-miR-1915-3p | CCCCAGGGCGACGCGGCGGG | MIMAT0007892 | hsa-miR-1915-3p | 4 | 0 | 0 | 0 | 0 | 0 | 0 | 2 |
| hsa-miR-1915-5p | CACCTTGCCTTGCTGCCCGGGCC | MIMAT0007891 | hsa-miR-1915-5p | 3 | 19 | 3 | 4 | 21 | 21 | 59 | 21 |
| hsa-miR-192-3p | CTGCCAATTCCATAGGTCACAGT | MIMAT0004543 | hsa-miR-192-3p | 0 | 2 | 0 | 0 | 2 | 0 | 0 | 0 |
| hsa-miR-192-5p | CTGACCTATGAATTGACAGCC | MIMAT0000222 | hsa-miR-192-5p | 107 | 182 | 96 | 115 | 140 | 199 | 136 | 129 |
| hsa-miR-193a-3p | AACTGGCCTACAAAGTCCC | MIMAT0000459 | hsa-miR-193a-3p | 66 | 94 | 76 | 105 | 143 | 110 | 134 | 99 |
| hsa-miR-193a-5p | TGGGTCTTTGCGGGCGAGATG | MIMAT0004614 | hsa-miR-193a-5p | 97744 | 117060 | 102723 | 115134 | 247063 | 240234 | 583980 | 237925 |
| hsa-miR-193b-3p | AACTGGCCCTCAAAGTCCCGCT | MIMAT0002819 | hsa-miR-193b-3p | 21923 | 41731 | 21917 | 37743 | 43248 | 42802 | 71150 | 37725 |
| hsa-miR-193b-5p | CGGGGTTTTGAGGGCGAGATG | MIMAT0004767 | hsa-miR-193b-5p | 1915 | 2918 | 2228 | 2603 | 4634 | 3923 | 6734 | 4941 |
| hsa-miR-194-3p | CAGTGGGGCTGCTGTTATCTGGT | MIMAT0004671 | hsa-miR-194-3p | 7 | 14 | 3 | 19 | 32 | 34 | 51 | 31 |
| hsa-miR-194-5p | TGTAACAGCAACTCCATG | MIMAT0000460 | hsa-miR-194-5p | 57 | 21 | 38 | 56 | 64 | 77 | 84 | 58 |
| hsa-miR-195-3p | CCAATATTGGCTGTGCTGCTCC | MIMAT0004615 | hsa-miR-195-3p | 76 | 147 | 88 | 82 | 100 | 75 | 82 | 68 |
| hsa-miR-195-5p | TAGCAGCACAGAAATATTGGC | MIMAT0000461 | hsa-miR-195-5p | 25 | 22 | 20 | 26 | 13 | 18 | 0 | 19 |
| hsa-miR-196a-5p | TAGGTAGTTTCATGTTGTTGGG | MIMAT0000226 | hsa-miR-196a-5p | 158 | 123 | 136 | 156 | 141 | 94 | 62 | 97 |
| hsa-miR-196b-3p | TCGACAGCACGACACTGCCTTC | MIMAT0009201 | hsa-miR-196b-3p | 0 | 0 | 0 | 0 | 0 | 0 | 0 | 0 |
| hsa-miR-196b-5p | TAGGTAGTTTCCTGTTGTTGGG | MIMAT0001080 | hsa-miR-196b-5p | 42 | 41 | 28 | 48 | 80 | 73 | 174 | 71 |
| hsa-miR-197-3p | TTCACCACCTTCTCCACCCAGC | MIMAT0000227 | hsa-miR-197-3p | 3970 | 9862 | 3165 | 5660 | 6616 | 9758 | 2710 | 4735 |
| hsa-miR-197-5p | CGGGTAGAGAGGGCAGTGGGAGG | MIMAT0022691 | hsa-miR-197-5p | 258 | 226 | 259 | 157 | 199 | 193 | 246 | 308 |
| hsa-miR-1972 | AGGCACGGTGGCTCATGC | MIMAT0009447 | hsa-miR-1972 | 9 | 6 | 21 | 16 | 2 | 0 | 0 | 0 |
| hsa-miR-1973 | CTAACCGTGCAAAGGTAGC | MIMAT0009448 | hsa-miR-1973 | 0 | 0 | 2 | 0 | 1 | 4 | 2 | 0 |
| hsa-miR-1976 | CTCCTGCCCTCCTTGCTGT | MIMAT0009451 | hsa-miR-1976 | 2 | 0 | 0 | 0 | 0 | 0 | 0 | 0 |
| hsa-miR-198 | ATTGGTCCAGAGGGGAGATAGGTTC | MIMAT0000228 | hsa-miR-198 | 0 | 6 | 0 | 0 | 0 | 0 | 0 | 0 |
| hsa-miR-199a-3p | ACAGTAGTCTGCACATTGGTT | MIMAT0000232 | hsa-miR-199a-3p | 119616 | 154026 | 112068 | 132955 | 114028 | 88462 | 66246 | 116622 |
| hsa-miR-199a-5p | CCCAGTGTTCAGACTACCTGTTC | MIMAT0000231 | hsa-miR-199a-5p | 3459 | 6259 | 3963 | 7176 | 6228 | 5353 | 7477 | 4218 |
| hsa-miR-199b-5p | CCCAGTGTTTAGACTATCTGTTC | MIMAT0000263 | hsa-miR-199b-5p | 10 | 19 | 10 | 19 | 5 | 11 | 4 | 10 |
| hsa-miR-19a-3p | TGTGCAAATCTATGCAAAACTG | MIMAT0000073 | hsa-miR-19a-3p | 0 | 0 | 1 | 1 | 0 | 1 | 1 | 0 |
| hsa-miR-19b-1-5p | AGTTTTGCAGGTTTGCATCCAGC | MIMAT0004491 | hsa-miR-19b-1-5p | 0 | 1 | 0 | 3 | 5 | 5 | 12 | 3 |
| hsa-miR-19b-3p | TGTGCAAATCCATGCAAAACTG | MIMAT0000074 | hsa-miR-19b-3p | 40 | 35 | 54 | 31 | 54 | 69 | 63 | 28 |
| hsa-miR-200a-3p | TAACACTGTCTGGTAACGATGTT | MIMAT0000682 | hsa-miR-200a-3p | 56 | 47 | 75 | 201 | 165 | 165 | 511 | 168 |
| hsa-miR-200a-5p | CATCTTACCGGACAGTGC | MIMAT0001620 | hsa-miR-200a-5p | 1 | 0 | 1 | 5 | 4 | 0 | 3 | 0 |
| hsa-miR-200b-3p | TAATACTGCCTGGTAATGATGAC | MIMAT0000318 | hsa-miR-200b-3p | 54 | 33 | 52 | 123 | 84 | 83 | 209 | 88 |
| hsa-miR-200b-5p | CATCTTACTGGGCAGCAT | MIMAT0004571 | hsa-miR-200b-5p | 12 | 15 | 8 | 11 | 6 | 5 | 11 | 3 |
| hsa-miR-200c-3p | TAATACTGCCGGGTAATGA | MIMAT0000617 | hsa-miR-200c-3p | 293 | 252 | 455 | 523 | 367 | 374 | 899 | 413 |
| hsa-miR-200c-5p | CGTCTTACCCAGCAGTGTT | MIMAT0004657 | hsa-miR-200c-5p | 1 | 0 | 1 | 0 | 0 | 0 | 1 | 0 |
| hsa-miR-202-3p | AGAGGTATAGGGCATGGGAA | MIMAT0002811 | hsa-miR-202-3p | 5 | 0 | 3 | 0 | 5 | 2 | 0 | 4 |
| hsa-miR-202-5p | TTCCTATGCATATACTTCTT | MIMAT0002810 | hsa-miR-202-5p | 0 | 0 | 0 | 0 | 0 | 1 | 0 | 0 |
| hsa-miR-203a-3p | TGAAATGTTTAGGACCACTAGT | MIMAT0000264 | hsa-miR-203a-3p | 26 | 86 | 33 | 40 | 58 | 39 | 63 | 65 |
| hsa-miR-203a-5p | AGTGGTTCTTAACAGTTCAACA | MIMAT0031890 | hsa-miR-203a-5p | 0 | 0 | 0 | 0 | 0 | 0 | 0 | 0 |
| hsa-miR-203b-3p | TTGAACTGTTAAGAACCACTG | MIMAT0019814 | hsa-miR-203b-3p | 0 | 0 | 0 | 0 | 0 | 0 | 0 | 0 |
| hsa-miR-204-3p | GCTGGGAAGGCAAAGGGACGT | MIMAT0022693 | hsa-miR-204-3p | 1939 | 1329 | 1559 | 1449 | 2705 | 2489 | 5104 | 2171 |
| hsa-miR-204-5p | TTCCCTTTGTCATCCTATGCCTG | MIMAT0000265 | hsa-miR-204-5p | 14802 | 25119 | 17041 | 29447 | 19355 | 27415 | 25570 | 20897 |
| hsa-miR-205-3p | GATTTCAGTGGAGTGAAGT | MIMAT0009197 | hsa-miR-205-3p | 1 | 1 | 1 | 0 | 3 | 0 | 0 | 0 |
| hsa-miR-205-5p | TCCTTCATTCCACCGGAGTCTG | MIMAT0000266 | hsa-miR-205-5p | 29 | 34 | 37 | 26 | 34 | 36 | 71 | 47 |
| hsa-miR-206 | TGGAATGTAAGGAAGTGTG | MIMAT0000462 | hsa-miR-206 | 2 | 3 | 7 | 5 | 2 | 7 | 0 | 0 |
| hsa-miR-208a-5p | GAGCTTTTGGCCCGGGTTATAC | MIMAT0026474 | hsa-miR-208a-5p | 2 | 0 | 0 | 0 | 0 | 0 | 0 | 0 |
| hsa-miR-20a-3p | ACTGCATTATGAGCACTTAAAGT | MIMAT0004493 | hsa-miR-20a-3p | 0 | 0 | 0 | 0 | 0 | 2 | 0 | 0 |
| hsa-miR-20a-5p | TAAAGTGCTTATAGTGCAGGTAGT | MIMAT0000075 | hsa-miR-20a-5p | 162 | 258 | 139 | 195 | 179 | 113 | 42 | 161 |
| hsa-miR-20b-3p | ACTGTAGTATGGGCACTTC | MIMAT0004752 | hsa-miR-20b-3p | 0 | 0 | 0 | 0 | 0 | 0 | 0 | 0 |
| hsa-miR-20b-5p | CAAAGTGCTCATAGTGCAGGTAG | MIMAT0001413 | hsa-miR-20b-5p | 1 | 0 | 0 | 0 | 0 | 0 | 0 | 0 |
| hsa-miR-21-3p | CAACACCAGTCGATGGGCTGT | MIMAT0004494 | hsa-miR-21-3p | 1176 | 1629 | 1417 | 1517 | 2189 | 1800 | 3235 | 1888 |
| hsa-miR-21-5p | TAGCTTATCAGACTGATGTTGAC | MIMAT0000076 | hsa-miR-21-5p | 128216 | 237135 | 136940 | 176790 | 170008 | 118186 | 166676 | 146246 |
| hsa-miR-210-3p | CTGTGCGTGTGACAGCGGCTG | MIMAT0000267 | hsa-miR-210-3p | 166 | 379 | 188 | 310 | 590 | 558 | 1104 | 477 |
| hsa-miR-210-5p | AGCCCCTGCCCACCGCACACTG | MIMAT0026475 | hsa-miR-210-5p | 390 | 721 | 402 | 877 | 1162 | 1487 | 630 | 753 |
| hsa-miR-211-5p | TTCCCTTTGTCATCCTTTGCCT | MIMAT0000268 | hsa-miR-211-5p | 7 | 17 | 6 | 13 | 3 | 9 | 12 | 9 |
| hsa-miR-2110 | TTGGGGAAACGGCCGCTGAGTG | MIMAT0010133 | hsa-miR-2110 | 13000 | 22961 | 15907 | 20391 | 32563 | 30046 | 47395 | 30692 |
| hsa-miR-2114-3p | CGAGCCTCAAGCAAGGGACTTC | MIMAT0011157 | hsa-miR-2114-3p | 0 | 0 | 0 | 0 | 0 | 0 | 0 | 0 |
| hsa-miR-2115-5p | CAGCTTCCATGACTCCTGA | MIMAT0011158 | hsa-miR-2115-5p | 0 | 0 | 0 | 0 | 0 | 0 | 0 | 0 |
| hsa-miR-2116-3p | TCCTCCCATGCCAAGAACTCC | MIMAT0011161 | hsa-miR-2116-3p | 171 | 196 | 181 | 169 | 136 | 109 | 132 | 143 |
| hsa-miR-2116-5p | GGGGTTCTTAGCATAGGAGGTC | MIMAT0011160 | hsa-miR-2116-5p | 20 | 11 | 6 | 24 | 25 | 25 | 25 | 18 |
| hsa-miR-212-3p | TAACAGTCTCCAGTCACGGCC | MIMAT0000269 | hsa-miR-212-3p | 1378 | 1757 | 1424 | 1246 | 948 | 855 | 594 | 1166 |
| hsa-miR-212-5p | ACCTTGGCTCTAGACTGCTTAC | MIMAT0022695 | hsa-miR-212-5p | 54 | 100 | 76 | 51 | 55 | 64 | 125 | 73 |
| hsa-miR-214-3p | ACAGCAGGCACAGACAGGCAGT | MIMAT0000271 | hsa-miR-214-3p | 9929 | 12214 | 7013 | 10912 | 12894 | 12671 | 6977 | 10782 |
| hsa-miR-214-5p | TGCCTGTCTACACTTGCTGTG | MIMAT0004564 | hsa-miR-214-5p | 1148 | 1320 | 1274 | 1532 | 1837 | 1836 | 2799 | 1435 |
| hsa-miR-215-5p | ATGACCTATGAATTGACAGCC | MIMAT0000272 | hsa-miR-215-5p | 0 | 0 | 0 | 0 | 0 | 0 | 0 | 0 |
| hsa-miR-216a-3p | CACAGTGGTCTCTGGGATTATG | MIMAT0022844 | hsa-miR-216a-3p | 2 | 2 | 7 | 7 | 8 | 10 | 27 | 14 |
| hsa-miR-216a-5p | TAATCTCAGCTGGCAACTGTG | MIMAT0000273 | hsa-miR-216a-5p | 0 | 0 | 1 | 1 | 0 | 0 | 0 | 0 |
| hsa-miR-216b-3p | CACACTTACCCGTAGAGATTCT | MIMAT0026721 | hsa-miR-216b-3p | 0 | 0 | 0 | 0 | 0 | 0 | 0 | 0 |
| hsa-miR-216b-5p | AAATCTCTGCAGGCAAATGTG | MIMAT0004959 | hsa-miR-216b-5p | 3 | 2 | 8 | 6 | 0 | 0 | 0 | 2 |
| hsa-miR-217 | TACTGCATCAGGAACTGAT | MIMAT0037308 | hsa-miR-217 | 0 | 0 | 0 | 0 | 0 | 0 | 0 | 0 |
| hsa-miR-218-1-3p | AAACATGGTTCCGTCAAGCAC | MIMAT0004565 | hsa-miR-218-1-3p | 2756 | 3636 | 2943 | 3602 | 3244 | 3728 | 3924 | 3825 |
| hsa-miR-218-2-3p | CATGGTTCTGTCAAGCACCGCGT | MIMAT0004566 | hsa-miR-218-2-3p | 9 | 19 | 12 | 40 | 53 | 64 | 146 | 56 |
| hsa-miR-218-5p | TTGTGCTTGATCTAACCATGTG | MIMAT0000275 | hsa-miR-218-5p | 11604 | 13668 | 11579 | 8147 | 6291 | 5664 | 5470 | 7570 |
| hsa-miR-219a-1-3p | AGAGTTGAGTCTGGACGTCCCG | MIMAT0004567 | hsa-miR-219a-1-3p | 23 | 47 | 18 | 20 | 46 | 43 | 98 | 44 |
| hsa-miR-219a-2-3p | AGAATTGTGGCTGGACATCTG | MIMAT0004675 | hsa-miR-219a-2-3p | 13 | 5 | 19 | 5 | 0 | 0 | 0 | 0 |
| hsa-miR-219a-5p | GATTGTCCAAACGCAATTCTTG | MIMAT0000276 | hsa-miR-219a-5p | 3 | 2 | 8 | 5 | 0 | 0 | 0 | 0 |
| hsa-miR-219b-5p | AGATGTCCAGCCACAATTCTCG | MIMAT0019747 | hsa-miR-219b-5p | 2 | 8 | 4 | 1 | 6 | 7 | 0 | 0 |
| hsa-miR-22-3p | AAGCTGCCAGTTGAAGAACTGT | MIMAT0000077 | hsa-miR-22-3p | 81692 | 122690 | 85296 | 110115 | 211740 | 194917 | 488753 | 223301 |
| hsa-miR-22-5p | AGTTCTTCAGTGGCAAGCTTT | MIMAT0004495 | hsa-miR-22-5p | 771 | 1067 | 703 | 1081 | 1144 | 1031 | 1675 | 1040 |
| hsa-miR-221-3p | AGCTACATTGTCTGCTGGGTTTC | MIMAT0000278 | hsa-miR-221-3p | 39997 | 78149 | 42397 | 62564 | 105258 | 99200 | 203585 | 98716 |
| hsa-miR-221-5p | ACCTGGCATACAATGTAGATTTCTGT | MIMAT0004568 | hsa-miR-221-5p | 2393 | 7615 | 2456 | 5138 | 4685 | 2374 | 1034 | 5999 |
| hsa-miR-222-3p | AGCTACATCTGGCTACTGGGTCTC | MIMAT0000279 | hsa-miR-222-3p | 812115 | 1850646 | 1041778 | 1464845 | 1417740 | 1104203 | 1393201 | 1437981 |
| hsa-miR-222-5p | TCAGTAGCCAGTGTAGATCCTG | MIMAT0004569 | hsa-miR-222-5p | 1 | 9 | 5 | 10 | 0 | 4 | 0 | 10 |
| hsa-miR-223-3p | TGTCAGTTTGTCAAATACCCC | MIMAT0000280 | hsa-miR-223-3p | 6 | 24 | 3 | 2 | 31 | 99 | 53 | 20 |
| hsa-miR-224-3p | AAATGGTGCCCTAGTGACTAC | MIMAT0009198 | hsa-miR-224-3p | 168 | 294 | 157 | 222 | 342 | 269 | 211 | 220 |
| hsa-miR-224-5p | CAAGTCACTAGTGGTTCCGTTT | MIMAT0000281 | hsa-miR-224-5p | 42511 | 72635 | 42414 | 59937 | 40979 | 30699 | 20225 | 40414 |
| hsa-miR-2276-3p | TCTGCAAGTGTCAGAGGCGAGG | MIMAT0011775 | hsa-miR-2276-3p | 4 | 9 | 4 | 5 | 6 | 13 | 18 | 5 |
| hsa-miR-2276-5p | CCGCCCTCTGTCACCTTGC | MIMAT0026921 | hsa-miR-2276-5p | 0 | 0 | 0 | 2 | 2 | 0 | 0 | 0 |
| hsa-miR-2277-3p | TGACAGCGCCCTGCCTGGCTCG | MIMAT0011777 | hsa-miR-2277-3p | 113 | 225 | 122 | 147 | 327 | 293 | 697 | 334 |
| hsa-miR-2277-5p | AGCGCGGGCTGAGCGCTGCCAGTC | MIMAT0017352 | hsa-miR-2277-5p | 9 | 47 | 6 | 34 | 22 | 19 | 39 | 25 |
| hsa-miR-2278 | GAGAGCAGTGTGTGTTGCCTGGG | MIMAT0011778 | hsa-miR-2278 | 71 | 72 | 40 | 50 | 142 | 104 | 229 | 127 |
| hsa-miR-2355-3p | ATTGTCCTTGCTGTTTGG | MIMAT0017950 | hsa-miR-2355-3p | 29 | 33 | 46 | 53 | 111 | 103 | 195 | 84 |
| hsa-miR-23a-3p | ATCACATTGCCAGGGATTTCC | MIMAT0000078 | hsa-miR-23a-3p | 100788 | 123435 | 96930 | 124020 | 98485 | 75833 | 66839 | 83802 |
| hsa-miR-23a-5p | GGGGTTCCTGGGGATGGGATT | MIMAT0004496 | hsa-miR-23a-5p | 512 | 710 | 463 | 561 | 1264 | 1361 | 2239 | 1359 |
| hsa-miR-23b-3p | ATCACATTGCCAGGGATTACCACT | MIMAT0000418 | hsa-miR-23b-3p | 54193 | 88503 | 54268 | 81553 | 73737 | 54609 | 30568 | 67570 |
| hsa-miR-23b-5p | TGGGTTCCTGGCATGCTGATTT | MIMAT0004587 | hsa-miR-23b-5p | 4973 | 10229 | 5521 | 6654 | 8729 | 8316 | 8841 | 8213 |
| hsa-miR-23c | ATCACATTGCCAGTGATTTCC | MIMAT0018000 | hsa-miR-23c | 18 | 39 | 9 | 41 | 45 | 33 | 79 | 37 |
| hsa-miR-24-1-5p | GGTGCCTACTGAGCTGATATC | MIMAT0000079 | hsa-miR-24-1-5p | 5 | 12 | 10 | 8 | 15 | 25 | 20 | 13 |
| hsa-miR-24-2-5p | GTGCCTACTGAGCTGAAACAC | MIMAT0004497 | hsa-miR-24-2-5p | 64 | 103 | 54 | 79 | 43 | 84 | 45 | 66 |
| hsa-miR-24-3p | TGGCTCAGTTCAGCAGGAACAG | MIMAT0000080 | hsa-miR-24-3p | 42807 | 64195 | 36897 | 55629 | 59410 | 87235 | 47711 | 48674 |
| hsa-miR-2467-3p | AGCAGAGGCAGAGAGGCTCAGG | MIMAT0019953 | hsa-miR-2467-3p | 0 | 5 | 2 | 0 | 0 | 0 | 0 | 0 |
| hsa-miR-2467-5p | TGAGGCTCTGTTAGCCTTGGCTT | MIMAT0019952 | hsa-miR-2467-5p | 0 | 0 | 0 | 2 | 0 | 0 | 0 | 0 |
| hsa-miR-25-3p | CATTGCACTTGTCTCGGTCTG | MIMAT0000081 | hsa-miR-25-3p | 1102 | 1331 | 1188 | 1195 | 1059 | 990 | 976 | 1098 |
| hsa-miR-25-5p | AGGCGGAGACTTGGGCAATTG | MIMAT0004498 | hsa-miR-25-5p | 957 | 2123 | 763 | 1079 | 1569 | 1746 | 2637 | 1720 |
| hsa-miR-2682-3p | ACGCCTCTTCAGCGCTGTCTT | MIMAT0013518 | hsa-miR-2682-3p | 172 | 277 | 163 | 226 | 381 | 310 | 727 | 360 |
| hsa-miR-2682-5p | CAGGCAGTGACTGTTCAGACGTC | MIMAT0013517 | hsa-miR-2682-5p | 14 | 34 | 13 | 20 | 28 | 18 | 31 | 21 |
| hsa-miR-26a-1-3p | CCTATTCTTGGTTACTTGCACG | MIMAT0004499 | hsa-miR-26a-1-3p | 14 | 5 | 8 | 17 | 5 | 3 | 0 | 16 |
| hsa-miR-26a-2-3p | AGGCCTATTCTTGATTACTTGTTT | MIMAT0004681 | hsa-miR-26a-2-3p | 2 | 4 | 2 | 5 | 4 | 4 | 9 | 11 |
| hsa-miR-26a-5p | TTCAAGTAATCCAGGATAGGCT | MIMAT0000082 | hsa-miR-26a-5p | 119134 | 187847 | 132292 | 191263 | 159842 | 127464 | 61792 | 152533 |
| hsa-miR-26b-3p | CCTGTTCTCCATTACTTGGCTC | MIMAT0004500 | hsa-miR-26b-3p | 78 | 150 | 48 | 108 | 174 | 142 | 295 | 204 |
| hsa-miR-26b-5p | TTCAAGTAATTCAGGATAGGTT | MIMAT0000083 | hsa-miR-26b-5p | 10107 | 14365 | 13312 | 15907 | 15091 | 10216 | 2394 | 12716 |
| hsa-miR-27a-3p | TTCACAGTGGCTAAGTTCCG | MIMAT0000084 | hsa-miR-27a-3p | 23207 | 35782 | 25409 | 26687 | 23624 | 16975 | 17303 | 23858 |
| hsa-miR-27a-5p | AGGGCTTAGCTGCTTGTGAGC | MIMAT0004501 | hsa-miR-27a-5p | 81 | 133 | 170 | 105 | 132 | 181 | 253 | 131 |
| hsa-miR-27b-3p | TTCACAGTGGCTAAGTTCTGC | MIMAT0000419 | hsa-miR-27b-3p | 13933 | 19776 | 15760 | 18801 | 17616 | 13442 | 12985 | 14927 |
| hsa-miR-27b-5p | AGAGCTTAGCTGATTGGTGAAC | MIMAT0004588 | hsa-miR-27b-5p | 497 | 956 | 273 | 598 | 1228 | 935 | 1872 | 1160 |
| hsa-miR-28-3p | CACTAGATTGTGAGCTCC | MIMAT0004502 | hsa-miR-28-3p | 1723 | 2273 | 1632 | 2439 | 4367 | 4119 | 7759 | 3864 |
| hsa-miR-28-5p | AAGGAGCTCACAGTCTATTG | MIMAT0000085 | hsa-miR-28-5p | 658 | 1306 | 639 | 920 | 930 | 890 | 755 | 863 |
| hsa-miR-2861 | TGGCGGCGGGCGGCGGGC | MIMAT0013802 | hsa-miR-2861 | 0 | 0 | 0 | 0 | 0 | 0 | 0 | 0 |
| hsa-miR-296-3p | GAGGGTTGGGTGGAGGCTCT | MIMAT0004679 | hsa-miR-296-3p | 1504 | 2123 | 1059 | 1130 | 2734 | 2742 | 5867 | 3587 |
| hsa-miR-296-5p | AGGGCCCCCCCTCAATCCTGT | MIMAT0000690 | hsa-miR-296-5p | 1002 | 1210 | 801 | 1445 | 2278 | 2527 | 1385 | 2206 |
| hsa-miR-297 | AUGUAUGUGUGCAUGUGCAUG | MIMAT0004450 | hsa-miR-297 | 0 | 0 | 0 | 0 | 0 | 0 | 0 | 0 |
| hsa-miR-299-3p | TATGTGGGATGGTAAACCGCTT | MIMAT0000687 | hsa-miR-299-3p | 25 | 42 | 28 | 31 | 43 | 44 | 55 | 43 |
| hsa-miR-299-5p | TGGTTTACCGTCCCACATACAT | MIMAT0002890 | hsa-miR-299-5p | 91 | 149 | 68 | 100 | 100 | 77 | 42 | 77 |
| hsa-miR-29a-3p | CTAGCACCATCTGAAATCGGTT | MIMAT0000086 | hsa-miR-29a-3p | 26005 | 42794 | 29434 | 49334 | 49053 | 44658 | 43561 | 39273 |
| hsa-miR-29a-5p | TGATTTCTTTTGGTGTTCAG | MIMAT0004503 | hsa-miR-29a-5p | 178 | 186 | 208 | 214 | 258 | 246 | 389 | 264 |
| hsa-miR-29b-1-5p | GCTGGTTTCATATGGTGGTTT | MIMAT0004514 | hsa-miR-29b-1-5p | 15199 | 25629 | 20141 | 17508 | 16371 | 11774 | 4793 | 21764 |
| hsa-miR-29b-2-5p | CTGGTTTCACATGGTGGCTT | MIMAT0004515 | hsa-miR-29b-2-5p | 253 | 428 | 337 | 336 | 474 | 383 | 705 | 462 |
| hsa-miR-29b-3p | TAGCACCATTTGAAATCAGTGTT | MIMAT0000100 | hsa-miR-29b-3p | 77 | 191 | 60 | 128 | 122 | 118 | 58 | 104 |
| hsa-miR-29c-3p | TAGCACCATTTGAAATCGGTT | MIMAT0000681 | hsa-miR-29c-3p | 19 | 30 | 39 | 35 | 38 | 55 | 48 | 23 |
| hsa-miR-29c-5p | ACCGATTTCTCCTGGTGTTC | MIMAT0004673 | hsa-miR-29c-5p | 736 | 986 | 837 | 1295 | 1669 | 1641 | 2287 | 1284 |
| hsa-miR-301a-3p | CAGTGCAATAGTATTGTCAAAGC | MIMAT0000688 | hsa-miR-301a-3p | 2 | 6 | 2 | 6 | 5 | 5 | 17 | 9 |
| hsa-miR-301a-5p | GCTCTGACTTTATTGCACTACT | MIMAT0022696 | hsa-miR-301a-5p | 0 | 0 | 1 | 0 | 0 | 2 | 3 | 0 |
| hsa-miR-301b-3p | CAGTGCAATGATATTGTCAAAGC | MIMAT0004958 | hsa-miR-301b-3p | 2 | 1 | 6 | 10 | 0 | 3 | 3 | 3 |
| hsa-miR-301b-5p | GCTCTGACGAGGTTGCACTACT | MIMAT0032026 | hsa-miR-301b-5p | 0 | 0 | 0 | 0 | 0 | 0 | 0 | 0 |
| hsa-miR-302a-3p | TAAGTGCTTCCATGTTTTGGTG | MIMAT0000684 | hsa-miR-302a-3p | 169 | 269 | 197 | 193 | 257 | 184 | 362 | 208 |
| hsa-miR-302a-5p | TAAACGTGGATGTACTTGCTTT | MIMAT0000683 | hsa-miR-302a-5p | 262 | 377 | 259 | 258 | 318 | 237 | 98 | 389 |
| hsa-miR-302b-3p | TAAGTGCTTCCATGTTTTAGT | MIMAT0000715 | hsa-miR-302b-3p | 153 | 202 | 132 | 143 | 102 | 86 | 51 | 141 |
| hsa-miR-302c-3p | TAAGTGCTTCCATGTTTCAGT | MIMAT0000717 | hsa-miR-302c-3p | 77 | 140 | 82 | 91 | 71 | 81 | 135 | 125 |
| hsa-miR-302c-5p | TTTAACATGGGGGTACCTGCT | MIMAT0000716 | hsa-miR-302c-5p | 26 | 35 | 35 | 25 | 42 | 29 | 60 | 73 |
| hsa-miR-302d-3p | TAAGTGCTTCCATGTTTGAGTGT | MIMAT0000718 | hsa-miR-302d-3p | 160 | 324 | 177 | 209 | 192 | 119 | 163 | 192 |
| hsa-miR-302e | UAAGUGCUUCCAUGCUU | MIMAT0005931 | hsa-miR-302e | 0 | 0 | 0 | 0 | 0 | 0 | 0 | 0 |
| hsa-miR-3064-3p | TGCCACACTGCAACACCTTAC | MIMAT0019865 | hsa-miR-3064-3p | 0 | 0 | 0 | 0 | 0 | 0 | 0 | 0 |
| hsa-miR-3064-5p | TCTGGCTGTTGTGGTGTGCAA | MIMAT0019864 | hsa-miR-3064-5p | 237 | 342 | 270 | 230 | 400 | 314 | 442 | 332 |
| hsa-miR-3065-3p | TCAGCACCAGGATATTGTTGGAGT | MIMAT0015378 | hsa-miR-3065-3p | 0 | 0 | 2 | 0 | 0 | 0 | 0 | 4 |
| hsa-miR-3065-5p | TCAACAAAATCACTGATGCTGGAGT | MIMAT0015066 | hsa-miR-3065-5p | 0 | 3 | 0 | 1 | 2 | 0 | 0 | 0 |
| hsa-miR-3074-3p | GATATCAGCTCAGTAGGCACCGT | MIMAT0015027 | hsa-miR-3074-3p | 1 | 0 | 2 | 0 | 2 | 0 | 0 | 2 |
| hsa-miR-3074-5p | GTTCCTGCTGAACTGAGCCAGT | MIMAT0019208 | hsa-miR-3074-5p | 13 | 14 | 11 | 18 | 20 | 16 | 28 | 14 |
| hsa-miR-30a-3p | CTTTCAGTCGGATGTTTGCAGC | MIMAT0000088 | hsa-miR-30a-3p | 2409 | 3534 | 2512 | 3544 | 2930 | 3039 | 2153 | 3494 |
| hsa-miR-30a-5p | TGTAAACATCCTCGACTGGAAGCT | MIMAT0000087 | hsa-miR-30a-5p | 4953 | 11331 | 5936 | 8998 | 8579 | 8131 | 8336 | 7446 |
| hsa-miR-30b-3p | CTGGGAGGTGGATGTTTACTTC | MIMAT0004589 | hsa-miR-30b-3p | 202 | 386 | 184 | 257 | 601 | 500 | 851 | 512 |
| hsa-miR-30b-5p | TGTAAACATCCTACACTCAGCT | MIMAT0000420 | hsa-miR-30b-5p | 80 | 75 | 75 | 77 | 90 | 87 | 30 | 83 |
| hsa-miR-30c-1-3p | CTGGGAGAGGGTTGTTTACTCC | MIMAT0004674 | hsa-miR-30c-1-3p | 74 | 123 | 77 | 74 | 230 | 226 | 406 | 239 |
| hsa-miR-30c-2-3p | CTGGGAGAAGGCTGTTTACTC | MIMAT0004550 | hsa-miR-30c-2-3p | 1495 | 2264 | 1606 | 1744 | 3409 | 3000 | 5326 | 3389 |
| hsa-miR-30c-5p | TGTAAACATCCTACACTCTCAGCT | MIMAT0000244 | hsa-miR-30c-5p | 174 | 295 | 179 | 185 | 191 | 200 | 105 | 151 |
| hsa-miR-30d-3p | CTTTCAGTCAGATGTTTGCTGCT | MIMAT0004551 | hsa-miR-30d-3p | 24 | 37 | 21 | 34 | 21 | 33 | 25 | 22 |
| hsa-miR-30d-5p | TGTAAACATCCCCGACTGGAAGCT | MIMAT0000245 | hsa-miR-30d-5p | 24787 | 64262 | 26898 | 51380 | 53772 | 49069 | 31047 | 48129 |
| hsa-miR-30e-3p | CTTTCAGTCGGATGTTTACAGC | MIMAT0000693 | hsa-miR-30e-3p | 172 | 202 | 219 | 221 | 196 | 137 | 100 | 165 |
| hsa-miR-30e-5p | TGTAAACATCCTTGACTGGAAGCT | MIMAT0000692 | hsa-miR-30e-5p | 36 | 51 | 39 | 40 | 50 | 51 | 21 | 30 |
| hsa-miR-31-3p | TGCTATGCCAACATATTGCCAT | MIMAT0004504 | hsa-miR-31-3p | 1448 | 3559 | 1315 | 2000 | 1331 | 1183 | 1141 | 1872 |
| hsa-miR-31-5p | AGGCAAGATGCTGGCATAGCTGT | MIMAT0000089 | hsa-miR-31-5p | 133743 | 196264 | 119505 | 151325 | 166811 | 131811 | 181544 | 153486 |
| hsa-miR-3115 | ATATGGGTTTACTAGTTGGTG | MIMAT0014977 | hsa-miR-3115 | 7 | 5 | 10 | 10 | 4 | 1 | 4 | 8 |
| hsa-miR-3117-3p | TAAGACTCATATAGTGCCAGGT | MIMAT0014979 | hsa-miR-3117-3p | 866 | 524 | 727 | 442 | 361 | 298 | 112 | 302 |
| hsa-miR-3118 | TGTGACTGGATTATGAAAATT | MIMAT0014980 | hsa-miR-3118 | 0 | 0 | 0 | 0 | 0 | 0 | 0 | 0 |
| hsa-miR-3119 | UGGCUUUUAACUUUGAUGGC | MIMAT0014981 | hsa-miR-3119 | 0 | 0 | 0 | 0 | 0 | 0 | 0 | 0 |
| hsa-miR-3120-5p | CCTGTCTGTGCCTGCTGTAC | MIMAT0019198 | hsa-miR-3120-5p | 0 | 0 | 0 | 0 | 0 | 0 | 0 | 0 |
| hsa-miR-3121-3p | TAAATAGAGTAGGCAAAGGAC | MIMAT0014983 | hsa-miR-3121-3p | 0 | 0 | 0 | 0 | 4 | 2 | 0 | 0 |
| hsa-miR-3122 | GTTGGGACAAGAGGACGGTCTT | MIMAT0014984 | hsa-miR-3122 | 0 | 1 | 0 | 0 | 2 | 2 | 7 | 2 |
| hsa-miR-3124-3p | CTTTCCTCACTCCCGTGAAGT | MIMAT0019200 | hsa-miR-3124-3p | 1 | 2 | 0 | 2 | 0 | 0 | 0 | 0 |
| hsa-miR-3124-5p | TTCGCGGGCGAAGGCAAAGTCG | MIMAT0014986 | hsa-miR-3124-5p | 87 | 94 | 118 | 95 | 108 | 92 | 203 | 100 |
| hsa-miR-3125 | TAGAGGAAGCTGTGGAGAGAAC | MIMAT0014988 | hsa-miR-3125 | 39 | 43 | 48 | 57 | 81 | 54 | 66 | 59 |
| hsa-miR-3126-3p | CATCTGGCATCCGTCACAC | MIMAT0015377 | hsa-miR-3126-3p | 133 | 179 | 95 | 108 | 93 | 78 | 89 | 98 |
| hsa-miR-3126-5p | TGAGGGACAGATGCCAGAAGC | MIMAT0014989 | hsa-miR-3126-5p | 179 | 235 | 184 | 171 | 183 | 121 | 191 | 180 |
| hsa-miR-3127-3p | TCCCCTTCTGCAGGCCTGC | MIMAT0019201 | hsa-miR-3127-3p | 39 | 27 | 21 | 16 | 11 | 21 | 18 | 20 |
| hsa-miR-3127-5p | ATCAGGGCTTGTGGAATGGGA | MIMAT0014990 | hsa-miR-3127-5p | 44 | 70 | 21 | 29 | 46 | 30 | 18 | 45 |
| hsa-miR-3129-3p | AACTAATCTCTACACTGCTGCC | MIMAT0019202 | hsa-miR-3129-3p | 22 | 33 | 10 | 17 | 22 | 30 | 19 | 23 |
| hsa-miR-3129-5p | GCAGTAGTGTAGAGATTGGTT | MIMAT0014992 | hsa-miR-3129-5p | 14 | 32 | 18 | 17 | 16 | 8 | 0 | 21 |
| hsa-miR-3130-3p | TGCACCGGAGACTGGGTAAGAC | MIMAT0014994 | hsa-miR-3130-3p | 2 | 0 | 0 | 9 | 11 | 11 | 35 | 11 |
| hsa-miR-3130-5p | TACCCAGTCTCCGGTGCAGCCT | MIMAT0014995 | hsa-miR-3130-5p | 37 | 69 | 40 | 56 | 38 | 43 | 50 | 41 |
| hsa-miR-3131 | TCGAGGACTGGTGGAAGGGCC | MIMAT0014996 | hsa-miR-3131 | 0 | 0 | 0 | 0 | 0 | 0 | 0 | 0 |
| hsa-miR-3132 | TGGGTAGAGAAGGAGCTCAGAGGT | MIMAT0014997 | hsa-miR-3132 | 1 | 0 | 0 | 0 | 0 | 1 | 0 | 0 |
| hsa-miR-3134 | ATGGATAAAAGACTACATAT | MIMAT0015000 | hsa-miR-3134 | 0 | 0 | 0 | 0 | 0 | 0 | 0 | 0 |
| hsa-miR-3135a | TGCCTAGGCTGAGACTGCAGTGT | MIMAT0015001 | hsa-miR-3135a | 0 | 0 | 0 | 0 | 0 | 0 | 0 | 0 |
| hsa-miR-3135b | GGCTGGACCGAGTGCAGTGGTG | MIMAT0018985 | hsa-miR-3135b | 6 | 4 | 5 | 16 | 18 | 15 | 49 | 15 |
| hsa-miR-3136-3p | TGGCCCAACCTATTCAGTTAGT | MIMAT0019203 | hsa-miR-3136-3p | 0 | 0 | 1 | 1 | 0 | 0 | 0 | 0 |
| hsa-miR-3136-5p | CTGACTGAATAGGTAGGGTCAT | MIMAT0015003 | hsa-miR-3136-5p | 0 | 0 | 0 | 0 | 6 | 7 | 22 | 7 |
| hsa-miR-3137 | CTGTAGCCTGGGAGCAATGGGGT | MIMAT0015005 | hsa-miR-3137 | 1 | 11 | 4 | 1 | 5 | 7 | 10 | 7 |
| hsa-miR-3138 | ACAGTGAGGTAGAGGGAGTGC | MIMAT0015006 | hsa-miR-3138 | 244 | 434 | 206 | 203 | 287 | 217 | 115 | 297 |
| hsa-miR-3139 | TAGGAGCTCAACAGATGCCTGT | MIMAT0015007 | hsa-miR-3139 | 0 | 0 | 0 | 2 | 0 | 0 | 0 | 0 |
| hsa-miR-3140-3p | AGCTTTTGGGAATTCAGGTAGT | MIMAT0015008 | hsa-miR-3140-3p | 5 | 6 | 8 | 11 | 8 | 2 | 0 | 11 |
| hsa-miR-3141 | GAGGGCGGGTGGAGGAGG | MIMAT0015010 | hsa-miR-3141 | 0 | 0 | 0 | 7 | 3 | 4 | 0 | 3 |
| hsa-miR-3142 | AAGGCCUUUCUGAACCUUCAGA | MIMAT0015011 | hsa-miR-3142 | 0 | 0 | 0 | 0 | 0 | 0 | 0 | 0 |
| hsa-miR-3143 | AUAACAUUGUAAAGCGCUUCUUUCG | MIMAT0015012 | hsa-miR-3143 | 0 | 0 | 0 | 0 | 0 | 0 | 0 | 0 |
| hsa-miR-3145-3p | TATTTTGAGTGTTTGGAATTG | MIMAT0015016 | hsa-miR-3145-3p | 0 | 0 | 0 | 0 | 0 | 0 | 0 | 0 |
| hsa-miR-3146 | CATGCTAGGATAGAAAGAATGGG | MIMAT0015018 | hsa-miR-3146 | 2 | 6 | 6 | 8 | 2 | 1 | 5 | 8 |
| hsa-miR-3148 | TGGAAAAAACTGGTGTGTGCTT | MIMAT0015021 | hsa-miR-3148 | 0 | 6 | 0 | 3 | 3 | 1 | 0 | 2 |
| hsa-miR-3149 | TATGTGTGTGTGTGTGTG | MIMAT0015022 | hsa-miR-3149 | 0 | 0 | 0 | 2 | 2 | 2 | 0 | 2 |
| hsa-miR-3150a-3p | TGGGGAGATCCTCGAGGTTGGC | MIMAT0015023 | hsa-miR-3150a-3p | 38 | 35 | 29 | 40 | 89 | 84 | 250 | 95 |
| hsa-miR-3150a-5p | CAACCTCGACGATCTCCTCAGC | MIMAT0019206 | hsa-miR-3150a-5p | 8 | 5 | 1 | 1 | 9 | 0 | 0 | 8 |
| hsa-miR-3150b-3p | TGAGGAGATCGTCGAGGTTGGC | MIMAT0018194 | hsa-miR-3150b-3p | 1 | 0 | 0 | 2 | 2 | 2 | 0 | 0 |
| hsa-miR-3150b-5p | AACCTCGAGGATCTCCCCAGCCAT | MIMAT0019226 | hsa-miR-3150b-5p | 0 | 0 | 2 | 0 | 0 | 0 | 0 | 0 |
| hsa-miR-3151-5p | GTGGGGCAATGGGATCAGGTGT | MIMAT0015024 | hsa-miR-3151-5p | 1 | 7 | 0 | 0 | 0 | 2 | 0 | 0 |
| hsa-miR-3152-3p | TGTGTTAGAATAGGGGCAA | MIMAT0015025 | hsa-miR-3152-3p | 0 | 0 | 0 | 0 | 0 | 3 | 0 | 0 |
| hsa-miR-3152-5p | ATTGCCTCTGTTCTAACACA | MIMAT0019207 | hsa-miR-3152-5p | 45 | 34 | 20 | 16 | 27 | 22 | 51 | 40 |
| hsa-miR-3153 | GGAAAGCGAGTAGGGACATTTT | MIMAT0015026 | hsa-miR-3153 | 0 | 7 | 0 | 0 | 0 | 0 | 0 | 0 |
| hsa-miR-3154 | CAGAAGGGGAGTTGGGAGC | MIMAT0015028 | hsa-miR-3154 | 14 | 49 | 19 | 25 | 34 | 17 | 0 | 66 |
| hsa-miR-3155a | CCAGGCTCTGCAGTGGGAACTGA | MIMAT0015029 | hsa-miR-3155a | 0 | 0 | 0 | 0 | 0 | 0 | 0 | 0 |
| hsa-miR-3156-3p | TGGCTCCCACTTCCAGATCTT | MIMAT0019209 | hsa-miR-3156-3p | 0 | 0 | 0 | 0 | 0 | 0 | 0 | 0 |
| hsa-miR-3156-5p | AAAGATCTGGAAGTGGGAGACC | MIMAT0015030 | hsa-miR-3156-5p | 0 | 0 | 0 | 0 | 5 | 5 | 18 | 5 |
| hsa-miR-3157-3p | CTGCCCTAGTCTAGCTGAAGC | MIMAT0019210 | hsa-miR-3157-3p | 0 | 5 | 2 | 0 | 4 | 0 | 0 | 2 |
| hsa-miR-3157-5p | TTCAGCCAGGCTAGTGCAGTCT | MIMAT0015031 | hsa-miR-3157-5p | 12 | 49 | 26 | 32 | 18 | 16 | 38 | 21 |
| hsa-miR-3158-3p | AAGGGCTTCCTCTCTGCAGGA | MIMAT0015032 | hsa-miR-3158-3p | 4 | 1 | 4 | 11 | 10 | 19 | 33 | 10 |
| hsa-miR-3158-5p | CCTGCAGAGAGGAAGCCCTTCT | MIMAT0019211 | hsa-miR-3158-5p | 3 | 1 | 6 | 3 | 2 | 0 | 0 | 4 |
| hsa-miR-3159 | TAGGATTACAAGTGTCGGCCAC | MIMAT0015033 | hsa-miR-3159 | 0 | 0 | 0 | 2 | 0 | 0 | 0 | 2 |
| hsa-miR-3162-3p | TCCCTACCCCTCCACTCCCCAA | MIMAT0019213 | hsa-miR-3162-3p | 0 | 0 | 0 | 0 | 0 | 0 | 0 | 0 |
| hsa-miR-3162-5p | TTAGGGAGTAGAAGGGTGGGG | MIMAT0015036 | hsa-miR-3162-5p | 5 | 4 | 5 | 9 | 0 | 0 | 0 | 4 |
| hsa-miR-3163 | TATAAAATGAGGGCAGTA | MIMAT0015037 | hsa-miR-3163 | 2 | 0 | 3 | 0 | 0 | 0 | 0 | 0 |
| hsa-miR-3164 | TGTGACTTTAAGGGAAATGGCG | MIMAT0015038 | hsa-miR-3164 | 4 | 2 | 0 | 2 | 6 | 0 | 0 | 7 |
| hsa-miR-3165 | AGGTGGATGCAATGTGAC | MIMAT0015039 | hsa-miR-3165 | 0 | 0 | 0 | 0 | 0 | 0 | 0 | 0 |
| hsa-miR-3166 | ACGCAGACAATGCCTACTGGCC | MIMAT0015040 | hsa-miR-3166 | 0 | 0 | 0 | 0 | 0 | 0 | 0 | 0 |
| hsa-miR-3170 | CTGGGGTTCTGAGACAGACAGT | MIMAT0015045 | hsa-miR-3170 | 1 | 4 | 0 | 2 | 2 | 0 | 0 | 5 |
| hsa-miR-3173-3p | AAGGAGGAAATAGGCAGGCC | MIMAT0015048 | hsa-miR-3173-3p | 23 | 22 | 8 | 10 | 18 | 10 | 0 | 19 |
| hsa-miR-3173-5p | TGCCCTGCCTGTTTTCTCCTTT | MIMAT0019214 | hsa-miR-3173-5p | 184 | 418 | 185 | 195 | 313 | 347 | 662 | 442 |
| hsa-miR-3174 | TAGTGAGTTAGAGATGCAGAGCC | MIMAT0015051 | hsa-miR-3174 | 1 | 8 | 4 | 2 | 0 | 0 | 0 | 0 |
| hsa-miR-3175 | CGGGGAGAGAACGCAGTGACGT | MIMAT0015052 | hsa-miR-3175 | 2 | 2 | 8 | 10 | 7 | 4 | 0 | 4 |
| hsa-miR-3176 | ACTGGCCTGGGACTACCGGGGG | MIMAT0015053 | hsa-miR-3176 | 41 | 35 | 44 | 18 | 29 | 40 | 66 | 24 |
| hsa-miR-3177-3p | TGCACGGCACTGGGGACACGT | MIMAT0015054 | hsa-miR-3177-3p | 459 | 706 | 455 | 357 | 535 | 506 | 511 | 707 |
| hsa-miR-3177-5p | TGTGTACACACGTGCCAGGCGCT | MIMAT0019215 | hsa-miR-3177-5p | 35 | 63 | 38 | 30 | 41 | 34 | 55 | 38 |
| hsa-miR-3179 | TAGAAGGGGTGAAATTTAAACGT | MIMAT0015056 | hsa-miR-3179 | 5 | 12 | 5 | 10 | 10 | 7 | 15 | 22 |
| hsa-miR-3180-5p | CTTCCAGACGCTCCGCCCCACG | MIMAT0015057 | hsa-miR-3180-5p | 67 | 60 | 43 | 61 | 83 | 65 | 14 | 102 |
| hsa-miR-3180 | TGGGGCGGAGCTTCCGGAGGCC | MIMAT0018178 | hsa-miR-3180 | 707 | 1210 | 663 | 887 | 2032 | 2016 | 4597 | 2254 |
| hsa-miR-3181 | ATCGGGCCCTCGGCGCCGGCC | MIMAT0015061 | hsa-miR-3181 | 6 | 8 | 10 | 4 | 2 | 0 | 0 | 7 |
| hsa-miR-3183 | GCCTCTCTCGGAGTCGCTCGG | MIMAT0015063 | hsa-miR-3183 | 0 | 0 | 0 | 2 | 0 | 1 | 0 | 0 |
| hsa-miR-3184-5p | TGAGGGGCCTCAGACCGAGCTTT | MIMAT0015064 | hsa-miR-3184-5p | 6 | 4 | 6 | 6 | 7 | 7 | 13 | 13 |
| hsa-miR-3186-5p | ACAGGCGTCTGTCTACGTGGCTA | MIMAT0015067 | hsa-miR-3186-5p | 0 | 0 | 0 | 0 | 0 | 0 | 0 | 0 |
| hsa-miR-3187-3p | TTGGCCATGGGGCTGCGCGGGG | MIMAT0015069 | hsa-miR-3187-3p | 3789 | 3192 | 4069 | 2270 | 2047 | 1953 | 3753 | 3075 |
| hsa-miR-3187-5p | CCTGGGCAGCGTGTGGCTGAAG | MIMAT0019216 | hsa-miR-3187-5p | 1 | 1 | 5 | 1 | 0 | 0 | 0 | 0 |
| hsa-miR-3188 | AGAGGCTTTGTGCGGATACGGG | MIMAT0015070 | hsa-miR-3188 | 39 | 17 | 28 | 22 | 34 | 39 | 28 | 23 |
| hsa-miR-3189-3p | CCCTTGGGTCTGATGGGGTAGC | MIMAT0015071 | hsa-miR-3189-3p | 22 | 19 | 25 | 22 | 50 | 30 | 50 | 37 |
| hsa-miR-3189-5p | TGCCCCATCTGTGCCCTGGGTAGG | MIMAT0019217 | hsa-miR-3189-5p | 0 | 7 | 0 | 0 | 0 | 0 | 0 | 0 |
| hsa-miR-3190-3p | TGGAAGGTAGACGGCCAGAGAGG | MIMAT0022839 | hsa-miR-3190-3p | 19 | 9 | 10 | 19 | 19 | 11 | 16 | 14 |
| hsa-miR-3190-5p | TGTCTGGCCAGCTACGTCCCC | MIMAT0015073 | hsa-miR-3190-5p | 18 | 21 | 23 | 24 | 8 | 8 | 1 | 15 |
| hsa-miR-3191-3p | TGGGGACGTAGCTGGCCAGAC | MIMAT0015075 | hsa-miR-3191-3p | 21 | 34 | 18 | 30 | 28 | 27 | 16 | 6 |
| hsa-miR-3191-5p | CTCTGGCCGTCTACCTTCCAC | MIMAT0022732 | hsa-miR-3191-5p | 17 | 38 | 28 | 51 | 55 | 42 | 100 | 68 |
| hsa-miR-3192-3p | TCCTCTGATCGCCCTCTCAGCT | MIMAT0027027 | hsa-miR-3192-3p | 0 | 0 | 0 | 0 | 0 | 0 | 0 | 0 |
| hsa-miR-3192-5p | TCTGGGAGGTTGTAGCAG | MIMAT0015076 | hsa-miR-3192-5p | 10 | 14 | 4 | 22 | 13 | 13 | 25 | 12 |
| hsa-miR-3193 | TCCTGCGTAGGATCTGAGGAGT | MIMAT0015077 | hsa-miR-3193 | 1 | 3 | 0 | 0 | 8 | 8 | 29 | 13 |
| hsa-miR-3194-3p | AGCTCTGCTGCTCACTGGCAGT | MIMAT0019218 | hsa-miR-3194-3p | 0 | 0 | 0 | 0 | 0 | 0 | 0 | 1 |
| hsa-miR-3194-5p | GGCCAGCCACCAGGAGGGCTGC | MIMAT0015078 | hsa-miR-3194-5p | 0 | 0 | 0 | 0 | 0 | 0 | 0 | 0 |
| hsa-miR-3195 | CGGCGCGCCGGGCCCGGG | MIMAT0015079 | hsa-miR-3195 | 21 | 21 | 45 | 19 | 20 | 24 | 59 | 25 |
| hsa-miR-3196 | GGGTCGGGGCGGCAGGGGCC | MIMAT0015080 | hsa-miR-3196 | 15 | 13 | 32 | 43 | 73 | 70 | 112 | 43 |
| hsa-miR-3197 | GGAGGCGCAGGCTCGGAAAGGCG | MIMAT0015082 | hsa-miR-3197 | 0 | 0 | 0 | 0 | 5 | 9 | 19 | 5 |
| hsa-miR-3198 | GTGGAGTCCTGGGGAA | MIMAT0015083 | hsa-miR-3198 | 13 | 33 | 5 | 7 | 21 | 17 | 21 | 21 |
| hsa-miR-3199 | ACTGCCTTAGGAGAAAGTTTCT | MIMAT0015084 | hsa-miR-3199 | 14 | 29 | 14 | 31 | 32 | 25 | 56 | 23 |
| hsa-miR-32-3p | CAATTTAGTGTGTGTGATATT | MIMAT0004505 | hsa-miR-32-3p | 13 | 14 | 15 | 13 | 26 | 11 | 2 | 23 |
| hsa-miR-32-5p | TATTGCACATTACTAAGTTGC | MIMAT0000090 | hsa-miR-32-5p | 4 | 3 | 2 | 2 | 0 | 1 | 2 | 1 |
| hsa-miR-3200-3p | CACCTTGCGCTACTCAGGTCTGC | MIMAT0015085 | hsa-miR-3200-3p | 146 | 267 | 174 | 195 | 221 | 180 | 205 | 162 |
| hsa-miR-3200-5p | AATCTGAGAAGGCGCACAAGGTT | MIMAT0017392 | hsa-miR-3200-5p | 36 | 34 | 41 | 17 | 13 | 12 | 13 | 34 |
| hsa-miR-3202 | UGGAAGGGAGAAGAGCUUUAAU | MIMAT0015089 | hsa-miR-3202 | 0 | 0 | 0 | 0 | 0 | 0 | 0 | 0 |
| hsa-miR-320a | AAAAGCTGGGTTGAGAGGGCG | MIMAT0000510 | hsa-miR-320a | 69406 | 117508 | 77276 | 95300 | 218032 | 227082 | 421362 | 219426 |
| hsa-miR-320b | AAAAGCTGGGTTGAGAGGG | MIMAT0005792 | hsa-miR-320b | 3331 | 2780 | 3044 | 2140 | 3941 | 4112 | 7561 | 4523 |
| hsa-miR-320c | AAAAGCTGGGTTGAGAGGGA | MIMAT0005793 | hsa-miR-320c | 748 | 720 | 680 | 631 | 1208 | 1308 | 2197 | 1305 |
| hsa-miR-320d | AAAAGCTGGGTTGAGAGGA | MIMAT0006764 | hsa-miR-320d | 53 | 78 | 129 | 59 | 53 | 51 | 84 | 38 |
| hsa-miR-320e | AAAAGCTGGGTTGAGAAGG | MIMAT0015072 | hsa-miR-320e | 74 | 18 | 55 | 20 | 12 | 18 | 32 | 11 |
| hsa-miR-323a-3p | GCACATTACACGGTCGACCTCT | MIMAT0000755 | hsa-miR-323a-3p | 5202 | 7952 | 4799 | 5457 | 5490 | 4943 | 3350 | 6469 |
| hsa-miR-323a-5p | AGGTGGTCCGTGGCGCGTTCG | MIMAT0004696 | hsa-miR-323a-5p | 4951 | 7961 | 5203 | 4300 | 7635 | 7491 | 15807 | 9418 |
| hsa-miR-323b-3p | CCCAATACACGGTCGACCTCT | MIMAT0015050 | hsa-miR-323b-3p | 505 | 850 | 384 | 537 | 559 | 519 | 573 | 744 |
| hsa-miR-323b-5p | AGGTTGTCCGTGGTGAGTTCGC | MIMAT0001630 | hsa-miR-323b-5p | 213 | 296 | 274 | 229 | 343 | 344 | 514 | 378 |
| hsa-miR-324-3p | ACTGCCCCAGGTGCTGCTGGT | MIMAT0000762 | hsa-miR-324-3p | 2688 | 3707 | 2268 | 3670 | 6037 | 6344 | 11877 | 4495 |
| hsa-miR-324-5p | CGCATCCCCTAGGGCATTGGTG | MIMAT0000761 | hsa-miR-324-5p | 303 | 465 | 319 | 451 | 455 | 451 | 556 | 396 |
| hsa-miR-326 | CCTCTGGGCCCTTCCTCCAGT | MIMAT0000756 | hsa-miR-326 | 68 | 94 | 66 | 77 | 158 | 145 | 257 | 133 |
| hsa-miR-328-3p | CTGGCCCTCTCTGCCCTTCCGT | MIMAT0000752 | hsa-miR-328-3p | 26081 | 53522 | 28062 | 37706 | 73386 | 72606 | 143526 | 66995 |
| hsa-miR-328-5p | GGGGGGCAGGAGGGGCTCAGGGT | MIMAT0026486 | hsa-miR-328-5p | 16 | 11 | 23 | 6 | 11 | 8 | 18 | 13 |
| hsa-miR-329-3p | AACACACCTGGTTAACCTCTT | MIMAT0001629 | hsa-miR-329-3p | 89 | 111 | 46 | 90 | 100 | 106 | 63 | 96 |
| hsa-miR-329-5p | GAGGTTTTCTGGGTTTCTGTTTC | MIMAT0026555 | hsa-miR-329-5p | 128 | 190 | 162 | 203 | 283 | 238 | 412 | 206 |
| hsa-miR-330-3p | GCAAAGCACACGGCCTGCAG | MIMAT0000751 | hsa-miR-330-3p | 696 | 749 | 685 | 503 | 568 | 465 | 442 | 432 |
| hsa-miR-330-5p | TCTCTGGGCCTGTGTCTTAGG | MIMAT0004693 | hsa-miR-330-5p | 490 | 530 | 343 | 359 | 617 | 540 | 1257 | 594 |
| hsa-miR-331-3p | GCCCCTGGGCCTATCCTAGAAT | MIMAT0000760 | hsa-miR-331-3p | 338 | 745 | 359 | 685 | 869 | 1235 | 351 | 456 |
| hsa-miR-331-5p | CTAGGTATGGTCCCAGGGATC | MIMAT0004700 | hsa-miR-331-5p | 208 | 135 | 191 | 119 | 98 | 142 | 81 | 71 |
| hsa-miR-335-3p | TTTTTCATTATTGCTCCTGACC | MIMAT0004703 | hsa-miR-335-3p | 63 | 108 | 43 | 109 | 123 | 103 | 36 | 65 |
| hsa-miR-335-5p | TCAAGAGCAATAACGAAAAATG | MIMAT0000765 | hsa-miR-335-5p | 5 | 3 | 3 | 7 | 2 | 0 | 0 | 11 |
| hsa-miR-337-3p | CTCCTATATGATGCCTTTCTTC | MIMAT0000754 | hsa-miR-337-3p | 1046 | 1572 | 1359 | 1143 | 1045 | 809 | 903 | 957 |
| hsa-miR-337-5p | GAACGGCTTCATACAGGAGT | MIMAT0004695 | hsa-miR-337-5p | 78 | 86 | 92 | 81 | 165 | 189 | 217 | 135 |
| hsa-miR-338-3p | TCCAGCATCAGTGATTTTGT | MIMAT0000763 | hsa-miR-338-3p | 0 | 0 | 1 | 0 | 0 | 0 | 0 | 2 |
| hsa-miR-338-5p | AACAATATCCTGGTGCTGAGT | MIMAT0004701 | hsa-miR-338-5p | 21 | 15 | 25 | 12 | 25 | 18 | 35 | 18 |
| hsa-miR-339-3p | TGAGCGCCTCGACGACAGAGCC | MIMAT0004702 | hsa-miR-339-3p | 330 | 620 | 286 | 594 | 806 | 712 | 1577 | 699 |
| hsa-miR-339-5p | TCCCTGTCCTCCAGGAGCTCACG | MIMAT0000764 | hsa-miR-339-5p | 52 | 58 | 30 | 64 | 123 | 350 | 170 | 81 |
| hsa-miR-33a-3p | CAATGTTTCCACAGTGCATC | MIMAT0004506 | hsa-miR-33a-3p | 1 | 0 | 0 | 0 | 0 | 1 | 0 | 0 |
| hsa-miR-33a-5p | GTGCATTGTAGTTGCATTGC | MIMAT0000091 | hsa-miR-33a-5p | 153 | 180 | 93 | 293 | 480 | 431 | 982 | 403 |
| hsa-miR-33b-3p | CAGTGCCTCGGCAGTGCAGCC | MIMAT0004811 | hsa-miR-33b-3p | 254 | 413 | 332 | 350 | 367 | 246 | 585 | 316 |
| hsa-miR-33b-5p | TGTGCATTGCTGTTGCATTGC | MIMAT0003301 | hsa-miR-33b-5p | 0 | 0 | 0 | 0 | 0 | 0 | 0 | 0 |
| hsa-miR-340-3p | TCCGTCTCAGTTACTTTATAGCC | MIMAT0000750 | hsa-miR-340-3p | 87 | 223 | 74 | 132 | 81 | 90 | 1 | 101 |
| hsa-miR-340-5p | TTATAAAGCAATGAGACTGATT | MIMAT0004692 | hsa-miR-340-5p | 19 | 26 | 24 | 17 | 32 | 34 | 37 | 27 |
| hsa-miR-342-3p | TCTCACACAGAAATCGCACCCGTC | MIMAT0000753 | hsa-miR-342-3p | 4784 | 12611 | 5130 | 8917 | 7389 | 5934 | 2658 | 9556 |
| hsa-miR-342-5p | AGGGGTGCTATCTGTGATTGAGG | MIMAT0004694 | hsa-miR-342-5p | 867 | 817 | 733 | 862 | 1042 | 1048 | 1181 | 873 |
| hsa-miR-345-3p | CCTGAACGAGGGGTCTGGAGT | MIMAT0022698 | hsa-miR-345-3p | 0 | 0 | 1 | 0 | 0 | 0 | 0 | 0 |
| hsa-miR-345-5p | GCTGACTCCTAGTCCAGGGCT | MIMAT0000772 | hsa-miR-345-5p | 9857 | 6507 | 9043 | 5512 | 6761 | 6288 | 15249 | 6671 |
| hsa-miR-346 | TGTCTGCCCGCATGCCTGCCTCT | MIMAT0000773 | hsa-miR-346 | 11 | 0 | 0 | 0 | 7 | 7 | 15 | 13 |
| hsa-miR-34a-3p | AATCAGCAAGTATACTGCCCTA | MIMAT0004557 | hsa-miR-34a-3p | 3 | 7 | 6 | 6 | 2 | 0 | 0 | 2 |
| hsa-miR-34a-5p | TGGCAGTGTCTTAGCTGGTTGT | MIMAT0000255 | hsa-miR-34a-5p | 109090 | 179249 | 135488 | 189129 | 431787 | 399019 | 843087 | 363993 |
| hsa-miR-34b-3p | AATCACTAACTCCACTGCCATC | MIMAT0004676 | hsa-miR-34b-3p | 279 | 444 | 318 | 119 | 388 | 186 | 312 | 326 |
| hsa-miR-34b-5p | AGGCAGTGTCATTAGCTGATTGT | MIMAT0000685 | hsa-miR-34b-5p | 6 | 7 | 9 | 11 | 7 | 2 | 0 | 5 |
| hsa-miR-34c-3p | AATCACTAACCACACGGCCAGG | MIMAT0004677 | hsa-miR-34c-3p | 1715 | 608 | 1236 | 650 | 537 | 450 | 516 | 416 |
| hsa-miR-34c-5p | AGGCAGTGTAGTTAGCTGATTGC | MIMAT0000686 | hsa-miR-34c-5p | 2 | 4 | 4 | 7 | 7 | 3 | 0 | 0 |
| hsa-miR-3529-5p | AGGTAGACTGGGATTTGTTGTT | MIMAT0019828 | hsa-miR-3529-5p | 0 | 0 | 0 | 0 | 6 | 2 | 0 | 0 |
| hsa-miR-3591-5p | TTTAGTGTGATAATGGCGTTTG | NA | hsa-miR-3591-5p | 4 | 0 | 1 | 0 | 2 | 0 | 0 | 0 |
| hsa-miR-3605-3p | CCTCCGTGTTACCTGTCCTCT | MIMAT0017982 | hsa-miR-3605-3p | 1478 | 2782 | 1509 | 1908 | 1913 | 1565 | 1493 | 1934 |
| hsa-miR-3605-5p | TGAGGATGGATAGCAAGGAAGCC | MIMAT0017981 | hsa-miR-3605-5p | 311 | 171 | 284 | 148 | 80 | 61 | 33 | 110 |
| hsa-miR-3607-3p | ACUGUAAACGCUUUCUGAUG | MIMAT0017985 | hsa-miR-3607-3p | 0 | 0 | 0 | 0 | 0 | 0 | 0 | 0 |
| hsa-miR-361-3p | UCCCCCAGGUGUGAUUCUGAUUU | MIMAT0004682 | hsa-miR-361-3p | 4 | 10 | 1 | 17 | 7 | 7 | 0 | 1 |
| hsa-miR-361-5p | TTATCAGAATCTCCAGGGGTAC | MIMAT0000703 | hsa-miR-361-5p | 890 | 943 | 859 | 908 | 1021 | 793 | 798 | 874 |
| hsa-miR-3610 | CGGAAAGGAGGCGCCGGCTGT | MIMAT0017987 | hsa-miR-3610 | 0 | 0 | 0 | 0 | 0 | 0 | 0 | 0 |
| hsa-miR-3613-3p | ACAAAAAAAAAAGCCCAACCCT | MIMAT0017991 | hsa-miR-3613-3p | 1 | 3 | 0 | 3 | 3 | 2 | 0 | 6 |
| hsa-miR-3613-5p | TGTTGTACTTTTTTTTTTGTTC | MIMAT0017990 | hsa-miR-3613-5p | 32 | 61 | 41 | 29 | 72 | 38 | 55 | 45 |
| hsa-miR-3614-3p | UAGCCUUCAGAUCUUGGUGUUUU | MIMAT0017993 | hsa-miR-3614-3p | 0 | 0 | 0 | 0 | 0 | 0 | 0 | 0 |
| hsa-miR-3614-5p | CCACTTGGATCTGAAGGCTGCC | MIMAT0017992 | hsa-miR-3614-5p | 11 | 9 | 14 | 16 | 21 | 25 | 21 | 14 |
| hsa-miR-3615 | TCTCTCGGCTCCTCGCGGCTCG | MIMAT0017994 | hsa-miR-3615 | 3518 | 6354 | 2710 | 4010 | 8643 | 6396 | 19106 | 8879 |
| hsa-miR-3617-3p | TCATCAGCACCCTATGTCCTTT | MIMAT0022966 | hsa-miR-3617-3p | 2 | 1 | 5 | 1 | 2 | 2 | 0 | 2 |
| hsa-miR-3617-5p | AAAGACATAGTTGCAAGATGGG | MIMAT0017997 | hsa-miR-3617-5p | 16 | 23 | 0 | 17 | 9 | 10 | 0 | 5 |
| hsa-miR-3619-3p | GGGACCATCCTGCCTGCTG | MIMAT0019219 | hsa-miR-3619-3p | 118 | 129 | 108 | 80 | 95 | 74 | 133 | 98 |
| hsa-miR-3619-5p | TCAGCAGGCAGGCTGGTGCAGCC | MIMAT0017999 | hsa-miR-3619-5p | 4 | 13 | 11 | 9 | 1 | 7 | 0 | 4 |
| hsa-miR-362-3p | AACACACCTATTCAAGGATT | MIMAT0004683 | hsa-miR-362-3p | 0 | 0 | 0 | 0 | 0 | 0 | 0 | 0 |
| hsa-miR-362-5p | AATCCTTGGAACCTAGGTGTGAGT | MIMAT0000705 | hsa-miR-362-5p | 1010 | 2494 | 1045 | 1519 | 1903 | 1446 | 1652 | 1791 |
| hsa-miR-3620-3p | TCACCCTGCATCCCGCACCCAG | MIMAT0018001 | hsa-miR-3620-3p | 14 | 22 | 32 | 15 | 13 | 6 | 0 | 11 |
| hsa-miR-3620-5p | GTGGGCTGGGCTGGGCTGGGC | MIMAT0022967 | hsa-miR-3620-5p | 244 | 194 | 286 | 150 | 278 | 329 | 489 | 412 |
| hsa-miR-3621 | GCAGGTCGGGGTCTGCAG | MIMAT0018002 | hsa-miR-3621 | 0 | 0 | 0 | 0 | 0 | 0 | 0 | 0 |
| hsa-miR-3622a-3p | CACCTGACCTCCCATGCCTGTG | MIMAT0018004 | hsa-miR-3622a-3p | 43 | 52 | 22 | 17 | 19 | 17 | 30 | 31 |
| hsa-miR-3622a-5p | CAGGCACGGGAGCTCAGGTGAG | MIMAT0018003 | hsa-miR-3622a-5p | 22 | 22 | 4 | 9 | 12 | 7 | 14 | 4 |
| hsa-miR-3622b-3p | TGAGCTCCCGTGCCTGTGC | MIMAT0018006 | hsa-miR-3622b-3p | 0 | 0 | 0 | 0 | 0 | 0 | 0 | 0 |
| hsa-miR-3622b-5p | CACAGGCATGGGAGGTCAGG | MIMAT0018005 | hsa-miR-3622b-5p | 0 | 2 | 0 | 0 | 0 | 0 | 0 | 0 |
| hsa-miR-363-3p | AATTGCACGGTATCCATCTGT | MIMAT0000707 | hsa-miR-363-3p | 9 | 11 | 13 | 4 | 5 | 73 | 10 | 7 |
| hsa-miR-363-5p | CGGGUGGAUCACGAUGCAAUUU | MIMAT0003385 | hsa-miR-363-5p | 0 | 0 | 0 | 0 | 0 | 0 | 0 | 0 |
| hsa-miR-3648 | GGGATCGCCGAGGGCCGG | MIMAT0018068 | hsa-miR-3648 | 35 | 38 | 50 | 37 | 14 | 12 | 33 | 30 |
| hsa-miR-3651 | CCCGGTCGCTGGTACATG | MIMAT0018071 | hsa-miR-3651 | 0 | 0 | 0 | 0 | 0 | 0 | 0 | 0 |
| hsa-miR-3652 | GCTGGAGGTGTGAGGATC | MIMAT0018072 | hsa-miR-3652 | 1 | 0 | 0 | 0 | 0 | 4 | 0 | 0 |
| hsa-miR-3653-3p | GCCTCTAAGAAGTTGACTGA | NA | hsa-miR-3653-3p | 8 | 13 | 2 | 5 | 11 | 17 | 17 | 17 |
| hsa-miR-3653-5p | CCCTCCTGATGATTCTTCTTCCTGAG | NA | hsa-miR-3653-5p | 0 | 0 | 0 | 0 | 0 | 0 | 0 | 0 |
| hsa-miR-3654 | GCGACTGGACAAGCTGAGG | MIMAT0018074 | hsa-miR-3654 | 0 | 0 | 0 | 0 | 0 | 0 | 0 | 0 |
| hsa-miR-3655 | GTCGCTGCGGTGTTGCTGT | MIMAT0018075 | hsa-miR-3655 | 0 | 0 | 0 | 0 | 0 | 0 | 0 | 0 |
| hsa-miR-3656 | GCGGGTGCGGGGGTGGGC | NA | hsa-miR-3656 | 51 | 41 | 42 | 51 | 96 | 101 | 131 | 104 |
| hsa-miR-3659 | TGAGTGTTGTCTACGAGGGCATT | MIMAT0018080 | hsa-miR-3659 | 2 | 0 | 0 | 0 | 0 | 0 | 0 | 0 |
| hsa-miR-365a-3p | TAATGCCCCTAAAAATCCTTAT | MIMAT0000710 | hsa-miR-365a-3p | 48 | 96 | 27 | 114 | 111 | 103 | 27 | 85 |
| hsa-miR-365a-5p | AGGGACTTTTGGGGGCAGATGTG | MIMAT0009199 | hsa-miR-365a-5p | 415 | 601 | 509 | 483 | 610 | 594 | 550 | 612 |
| hsa-miR-365b-5p | AGGGACTTTCAGGGGCAGCTGTG | MIMAT0022833 | hsa-miR-365b-5p | 12504 | 18666 | 14497 | 13143 | 15732 | 14500 | 17459 | 18525 |
| hsa-miR-3661 | TGACCTGGGACTCGGACAGCTG | MIMAT0018082 | hsa-miR-3661 | 161 | 236 | 223 | 172 | 226 | 231 | 322 | 325 |
| hsa-miR-3662 | GAAAATGATGAGTAGTGACTGATG | MIMAT0018083 | hsa-miR-3662 | 3 | 0 | 2 | 1 | 0 | 0 | 0 | 7 |
| hsa-miR-3663-5p | GCTGGTCTGCGTGGTGCTCGGG | MIMAT0018084 | hsa-miR-3663-5p | 0 | 0 | 0 | 0 | 0 | 0 | 0 | 0 |
| hsa-miR-3664-3p | TCTCAGGAGTAAAGACAGAGTT | MIMAT0019220 | hsa-miR-3664-3p | 1 | 6 | 5 | 1 | 0 | 0 | 0 | 0 |
| hsa-miR-3667-3p | ACCTTCCTCTCCATGGGTCTT | MIMAT0018090 | hsa-miR-3667-3p | 0 | 0 | 0 | 0 | 0 | 0 | 0 | 0 |
| hsa-miR-3675-5p | ATGGGGCTTCTGTAGAGATTTC | MIMAT0018098 | hsa-miR-3675-5p | 20 | 24 | 22 | 23 | 29 | 22 | 36 | 35 |
| hsa-miR-3677-3p | CTCGTGGGCTCTGGCCACGGCC | MIMAT0018101 | hsa-miR-3677-3p | 0 | 0 | 0 | 2 | 0 | 0 | 0 | 0 |
| hsa-miR-3677-5p | CAGTGGCCAGAGCCCTGCAGTG | MIMAT0019221 | hsa-miR-3677-5p | 0 | 0 | 0 | 0 | 0 | 0 | 0 | 0 |
| hsa-miR-3678-3p | TGCAGAGTTTGTACGGACCGGT | MIMAT0018103 | hsa-miR-3678-3p | 0 | 0 | 0 | 0 | 0 | 0 | 0 | 0 |
| hsa-miR-3678-5p | TCCGTACAAACTCTGCTGTGTT | MIMAT0018102 | hsa-miR-3678-5p | 2 | 0 | 0 | 0 | 2 | 0 | 0 | 0 |
| hsa-miR-3679-3p | CTTCCCCCCAGTAATCTTCATCT | MIMAT0018105 | hsa-miR-3679-3p | 0 | 0 | 0 | 1 | 0 | 0 | 0 | 0 |
| hsa-miR-3679-5p | TGAGGATATGGCAGGGAAGGG | MIMAT0018104 | hsa-miR-3679-5p | 1030 | 699 | 909 | 429 | 301 | 299 | 123 | 608 |
| hsa-miR-3680-3p | TTTGCATGACCCTGGGAGT | MIMAT0018107 | hsa-miR-3680-3p | 4 | 19 | 7 | 4 | 0 | 0 | 0 | 10 |
| hsa-miR-3680-5p | GACTCACTCACAGGATTGTG | MIMAT0018106 | hsa-miR-3680-5p | 0 | 0 | 0 | 0 | 0 | 0 | 0 | 0 |
| hsa-miR-3682-3p | TGATGATACAGGTGGAGGTAGT | MIMAT0018110 | hsa-miR-3682-3p | 18 | 18 | 17 | 10 | 19 | 21 | 27 | 27 |
| hsa-miR-3687 | TCCCGGACAGGCGTTCGTGCGAC | NA | hsa-miR-3687 | 16 | 22 | 25 | 18 | 35 | 37 | 86 | 45 |
| hsa-miR-3688-3p | TATGGAAAGACTTTGCCACTCT | MIMAT0018116 | hsa-miR-3688-3p | 4 | 15 | 4 | 6 | 0 | 2 | 0 | 9 |
| hsa-miR-3688-5p | TGGCAAAGTCTTTCCATATGTG | MIMAT0019223 | hsa-miR-3688-5p | 0 | 0 | 0 | 0 | 0 | 0 | 0 | 0 |
| hsa-miR-3689b-5p | TGTGATATCATGGTTCCTGGGAT | MIMAT0018180 | hsa-miR-3689b-5p | 0 | 0 | 0 | 0 | 0 | 0 | 0 | 0 |
| hsa-miR-3691-3p | CAAGTCTGCGTCATCCTCTCC | MIMAT0019224 | hsa-miR-369-3p | 90 | 165 | 72 | 131 | 69 | 57 | 29 | 124 |
| hsa-miR-3691-5p | TAGTGGATGATGGAGACTCGGT | MIMAT0018120 | hsa-miR-369-5p | 1262 | 2240 | 1434 | 1363 | 939 | 900 | 989 | 1238 |
| hsa-miR-369-3p | AATAATACATGGTTGATCTTT | MIMAT0000721 | hsa-miR-3691-3p | 4 | 9 | 2 | 13 | 9 | 6 | 16 | 6 |
| hsa-miR-369-5p | AGATCGACCGTGTTATATTCG | MIMAT0001621 | hsa-miR-3691-5p | 82 | 128 | 116 | 106 | 140 | 100 | 120 | 187 |
| hsa-miR-370-3p | GCCTGCTGGGGTGGAACCTGGT | MIMAT0000722 | hsa-miR-370-3p | 10835 | 12205 | 8736 | 8887 | 14762 | 19007 | 19256 | 23143 |
| hsa-miR-370-5p | CAGGTCACGTCTCTGCAGTTACAC | MIMAT0026483 | hsa-miR-370-5p | 103 | 157 | 150 | 168 | 157 | 99 | 113 | 113 |
| hsa-miR-371a-3p | AAGTGCCGCCATCTTTTGAGTGT | MIMAT0000723 | hsa-miR-371a-3p | 60 | 139 | 47 | 123 | 144 | 151 | 172 | 147 |
| hsa-miR-371a-5p | ACTCAAACTGTGGGGGCACTT | MIMAT0004687 | hsa-miR-371a-5p | 1 | 0 | 0 | 0 | 3 | 2 | 0 | 2 |
| hsa-miR-371b-3p | AGTGCCCCCACAGTTTGAGTGC | MIMAT0019893 | hsa-miR-371b-3p | 0 | 0 | 0 | 0 | 0 | 0 | 0 | 0 |
| hsa-miR-371b-5p | CACTCAAAAGATGGCGGCACT | MIMAT0019892 | hsa-miR-371b-5p | 0 | 0 | 0 | 0 | 0 | 0 | 0 | 0 |
| hsa-miR-372-3p | AAAGTGCTGCGACATTTGAGCGT | MIMAT0000724 | hsa-miR-372-3p | 0 | 0 | 0 | 1 | 0 | 0 | 0 | 0 |
| hsa-miR-373-3p | GAAGTGCTTCGATTTTGGGGTGT | MIMAT0000726 | hsa-miR-373-3p | 2 | 1 | 5 | 2 | 12 | 9 | 12 | 7 |
| hsa-miR-373-5p | TACTCAAAATGGGGGCGCTT | MIMAT0000725 | hsa-miR-373-5p | 3 | 0 | 1 | 0 | 3 | 1 | 1 | 3 |
| hsa-miR-374a-3p | CTTATCAGATTGTATTGTAATT | MIMAT0004688 | hsa-miR-374a-3p | 0 | 0 | 0 | 0 | 0 | 0 | 0 | 1 |
| hsa-miR-374a-5p | TTATAATACAACCTGATAAGTGT | MIMAT0000727 | hsa-miR-374a-5p | 2 | 17 | 4 | 3 | 7 | 5 | 0 | 15 |
| hsa-miR-374b-3p | CTTAGCAGGTTGTATTATCATT | MIMAT0004956 | hsa-miR-374b-3p | 6 | 6 | 5 | 6 | 8 | 5 | 10 | 7 |
| hsa-miR-374b-5p | ATATAATACAACCTGCTAAGTG | MIMAT0004955 | hsa-miR-374b-5p | 60 | 43 | 56 | 45 | 35 | 30 | 19 | 17 |
| hsa-miR-374c-5p | ATACAACCTGCTAAGTGA | MIMAT0018443 | hsa-miR-374c-5p | 0 | 0 | 0 | 0 | 0 | 0 | 0 | 0 |
| hsa-miR-375 | TTTGTTCGTTCGGCTCGCGTG | NA | hsa-miR-375 | 87 | 206 | 97 | 120 | 152 | 154 | 215 | 149 |
| hsa-miR-376a-2-5p | GTGGATTTTCCTTCTATGGTT | MIMAT0022928 | hsa-miR-376a-2-5p | 2 | 0 | 2 | 1 | 9 | 9 | 19 | 10 |
| hsa-miR-376a-3p | ATCATAGAGGAAAATCCACGT | MIMAT0000729 | hsa-miR-376a-3p | 111 | 204 | 104 | 118 | 127 | 91 | 123 | 169 |
| hsa-miR-376a-5p | TAGATTCTCCTTCTATGAGT | MIMAT0003386 | hsa-miR-376a-5p | 1 | 4 | 5 | 2 | 8 | 9 | 14 | 4 |
| hsa-miR-376b-3p | ATCATAGAGGAAAATCCATGT | MIMAT0002172 | hsa-miR-376b-3p | 105 | 246 | 63 | 104 | 91 | 58 | 82 | 237 |
| hsa-miR-376b-5p | GTGGATATTCCTTCTATGTT | MIMAT0022923 | hsa-miR-376b-5p | 1 | 1 | 4 | 1 | 6 | 3 | 10 | 3 |
| hsa-miR-376c-3p | AACATAGAGGAAATTCCACGT | MIMAT0000720 | hsa-miR-376c-3p | 345 | 751 | 359 | 481 | 483 | 415 | 546 | 550 |
| hsa-miR-376c-5p | GTGGATATTCCTTCTATGTTT | MIMAT0022861 | hsa-miR-376c-5p | 5 | 5 | 3 | 5 | 6 | 1 | 0 | 2 |
| hsa-miR-377-3p | ATCACACAAAGGCAACTTTTGT | MIMAT0000730 | hsa-miR-377-3p | 2 | 21 | 0 | 8 | 18 | 17 | 15 | 21 |
| hsa-miR-377-5p | AGAGGTTGCCCTTGGTGAATTC | MIMAT0004689 | hsa-miR-377-5p | 1255 | 2723 | 1295 | 1995 | 3782 | 3665 | 8101 | 3924 |
| hsa-miR-378a-3p | ACTGGACTTGGAGTCAGAAGGC | MIMAT0000732 | hsa-miR-378a-3p | 36938 | 22399 | 33553 | 21965 | 40071 | 37329 | 97489 | 37431 |
| hsa-miR-378a-5p | CTCCTGACTCCAGGTCCTGTGT | MIMAT0000731 | hsa-miR-378a-5p | 320 | 500 | 276 | 339 | 618 | 635 | 1398 | 610 |
| hsa-miR-378b | ACTGGACTTGGAGGCAGAAGG | MIMAT0014999 | hsa-miR-378b | 0 | 0 | 1 | 0 | 1 | 0 | 3 | 0 |
| hsa-miR-378c | ACTGGACTTGGAGTCAGAAGAC | MIMAT0016847 | hsa-miR-378c | 70 | 48 | 30 | 66 | 122 | 119 | 286 | 117 |
| hsa-miR-378d | ACTGGACTTGGAGTCAGAAAGC | MIMAT0018926 | hsa-miR-378d | 4 | 3 | 2 | 1 | 1 | 2 | 5 | 3 |
| hsa-miR-378e | ACTGGACTTGGAGTCAGG | MIMAT0018927 | hsa-miR-378e | 4 | 1 | 4 | 1 | 0 | 0 | 1 | 1 |
| hsa-miR-378f | ACTGGACTTGGAGCCAGAAGGC | MIMAT0018932 | hsa-miR-378f | 11 | 3 | 10 | 5 | 9 | 9 | 21 | 7 |
| hsa-miR-378g | ACTGGGCTTGGAGTCAGAAGGC | MIMAT0018937 | hsa-miR-378g | 32 | 15 | 18 | 13 | 81 | 74 | 191 | 69 |
| hsa-miR-378h | ACTGGACTTGGTGTCAGAAGG | MIMAT0018984 | hsa-miR-378h | 7 | 1 | 5 | 2 | 0 | 1 | 3 | 0 |
| hsa-miR-378i | ACTGGACTAGGAGTCAGAAGGC | MIMAT0019074 | hsa-miR-378i | 7 | 2 | 9 | 3 | 3 | 3 | 8 | 6 |
| hsa-miR-379-3p | TATGTAACATGGTCCACTAAC | MIMAT0004690 | hsa-miR-379-3p | 262 | 327 | 274 | 285 | 144 | 124 | 53 | 207 |
| hsa-miR-379-5p | TGGTAGACTATGGAACGTAGG | MIMAT0000733 | hsa-miR-379-5p | 20491 | 11223 | 17635 | 10752 | 9257 | 6478 | 7262 | 8264 |
| hsa-miR-380-3p | TATGTAATATGGTCCACATCT | MIMAT0000735 | hsa-miR-380-3p | 26 | 17 | 22 | 14 | 10 | 8 | 1 | 13 |
| hsa-miR-380-5p | ATGGTTGACCATAGAACATGCG | MIMAT0000734 | hsa-miR-380-5p | 0 | 0 | 0 | 0 | 0 | 1 | 0 | 1 |
| hsa-miR-381-3p | TATACAAGGGCAAGCTCTCTGT | MIMAT0000736 | hsa-miR-381-3p | 426 | 776 | 373 | 558 | 678 | 642 | 1201 | 693 |
| hsa-miR-381-5p | AGCGAGGTTGCCCTTTGTATATT | MIMAT0022862 | hsa-miR-381-5p | 242 | 584 | 262 | 454 | 367 | 258 | 176 | 390 |
| hsa-miR-382-3p | AATCATTCACGGACAACACTT | MIMAT0022697 | hsa-miR-382-3p | 12 | 10 | 5 | 15 | 23 | 4 | 0 | 10 |
| hsa-miR-382-5p | GAAGTTGTTCGTGGTGGATTCG | MIMAT0000737 | hsa-miR-382-5p | 9702 | 13198 | 9449 | 11563 | 19854 | 19423 | 34349 | 17929 |
| hsa-miR-383-5p | AGATCAGAAGGTGATTGTGGCT | MIMAT0000738 | hsa-miR-383-5p | 11 | 44 | 18 | 20 | 55 | 52 | 119 | 54 |
| hsa-miR-384 | ATTCCTAGAAATTGTTCACAAT | MIMAT0001075 | hsa-miR-384 | 0 | 5 | 0 | 0 | 0 | 0 | 0 | 0 |
| hsa-miR-3907 | AGGTGCTCCAGGCTGGCTC | MIMAT0018179 | hsa-miR-3907 | 0 | 0 | 0 | 0 | 0 | 0 | 0 | 0 |
| hsa-miR-3908 | GAGCAATGTAGGTAGACTGTTT | MIMAT0018182 | hsa-miR-3908 | 0 | 2 | 0 | 0 | 0 | 0 | 0 | 1 |
| hsa-miR-3909 | TGTCCTCTAGGGCCTGCAGTCT | MIMAT0018183 | hsa-miR-3909 | 387 | 698 | 329 | 443 | 386 | 256 | 336 | 356 |
| hsa-miR-3911 | TGTGTGGATCCTGGAGGAGGC | MIMAT0018185 | hsa-miR-3911 | 11 | 9 | 11 | 4 | 16 | 22 | 45 | 18 |
| hsa-miR-3912-3p | TAACGCATAATATGGACATGTTAT | MIMAT0018186 | hsa-miR-3912-3p | 0 | 0 | 1 | 1 | 0 | 0 | 0 | 0 |
| hsa-miR-3912-5p | CATATTATGGGTTAGTTG | MIMAT0027036 | hsa-miR-3912-5p | 0 | 0 | 0 | 0 | 2 | 0 | 0 | 0 |
| hsa-miR-3913-3p | ACATCAAGATCAGTCCCAAAT | MIMAT0019225 | hsa-miR-3913-3p | 0 | 5 | 0 | 0 | 0 | 0 | 0 | 0 |
| hsa-miR-3913-5p | ATTTGGGACTGATCTTGATGTC | MIMAT0018187 | hsa-miR-3913-5p | 0 | 2 | 1 | 0 | 15 | 10 | 34 | 10 |
| hsa-miR-3916 | GAAGAGGAAGAAATGGCTGGTTCTC | MIMAT0018190 | hsa-miR-3916 | 0 | 0 | 0 | 0 | 2 | 0 | 0 | 2 |
| hsa-miR-3917 | CGGCTCGGACTGAGCAGGTGGGT | MIMAT0018191 | hsa-miR-3917 | 0 | 0 | 0 | 0 | 6 | 6 | 20 | 6 |
| hsa-miR-3918 | ACAGGGCCGCAGATGGAGACT | MIMAT0018192 | hsa-miR-3918 | 2 | 6 | 0 | 5 | 7 | 0 | 0 | 0 |
| hsa-miR-3921 | TCTCTGAGTACCATATGCCTTG | MIMAT0018196 | hsa-miR-3921 | 0 | 0 | 0 | 0 | 0 | 0 | 0 | 2 |
| hsa-miR-3922-3p | TCTGGCCTTGACTTGACTCTTC | MIMAT0018197 | hsa-miR-3922-3p | 1 | 10 | 1 | 0 | 5 | 5 | 17 | 10 |
| hsa-miR-3922-5p | TCAAGGCCAGAGGTCCCACAGC | MIMAT0019227 | hsa-miR-3922-5p | 4 | 0 | 2 | 2 | 0 | 0 | 0 | 4 |
| hsa-miR-3925-5p | AAGAGAACTGAAAGTGGAGCCT | MIMAT0018200 | hsa-miR-3925-5p | 0 | 0 | 0 | 0 | 0 | 0 | 0 | 0 |
| hsa-miR-3928-3p | GGAGGAACCTTGGAGCTTCGGC | MIMAT0018205 | hsa-miR-3928-3p | 38 | 101 | 43 | 78 | 154 | 141 | 331 | 140 |
| hsa-miR-3928-5p | GAAGCTCTAAGGTTCCGCCTG | MIMAT0027037 | hsa-miR-3928-5p | 7 | 5 | 3 | 2 | 7 | 4 | 0 | 10 |
| hsa-miR-3929 | GAGGCTGATGTGAGTAGACCACT | MIMAT0018206 | hsa-miR-3929 | 10 | 20 | 5 | 9 | 8 | 5 | 1 | 24 |
| hsa-miR-3934-3p | TGCTCAGGTTGCACAGCAGG | MIMAT0022975 | hsa-miR-3934-3p | 0 | 0 | 0 | 0 | 0 | 0 | 0 | 0 |
| hsa-miR-3934-5p | TCAGGTGTGGAAACTGAGGCAGG | MIMAT0018349 | hsa-miR-3934-5p | 1 | 6 | 4 | 6 | 1 | 7 | 0 | 9 |
| hsa-miR-3935 | AGATACGAGCACCAGCCACCC | MIMAT0018350 | hsa-miR-3935 | 2 | 2 | 8 | 2 | 0 | 0 | 0 | 0 |
| hsa-miR-3936 | TAAGGGGTGTATGGCAGATGC | MIMAT0018351 | hsa-miR-3936 | 0 | 0 | 0 | 0 | 5 | 0 | 0 | 2 |
| hsa-miR-3937 | CAACAGGCGGCTGTAGCAATGGT | MIMAT0018352 | hsa-miR-3937 | 0 | 3 | 0 | 0 | 0 | 0 | 0 | 0 |
| hsa-miR-3938 | AATTCCCTTGTAGATAACCCGG | MIMAT0018353 | hsa-miR-3938 | 0 | 0 | 0 | 0 | 0 | 0 | 0 | 0 |
| hsa-miR-3939 | TACGCGCAGACCACAGGATGTC | MIMAT0018355 | hsa-miR-3939 | 2 | 13 | 2 | 6 | 0 | 2 | 0 | 2 |
| hsa-miR-3940-3p | CAGCCCGGATCCCAGCCCACT | MIMAT0018356 | hsa-miR-3940-3p | 254 | 388 | 232 | 205 | 216 | 256 | 156 | 321 |
| hsa-miR-3940-5p | GAGGTGGGTTGGGCCGGGCT | MIMAT0019229 | hsa-miR-3940-5p | 0 | 0 | 0 | 0 | 13 | 9 | 32 | 11 |
| hsa-miR-3941 | TTACACACAACTGAGGATCATA | MIMAT0018357 | hsa-miR-3941 | 0 | 0 | 0 | 0 | 0 | 0 | 0 | 0 |
| hsa-miR-3944-3p | TTCGGGCTGGCCTGCTGCTCCGG | MIMAT0018360 | hsa-miR-3944-3p | 66 | 136 | 73 | 72 | 159 | 158 | 436 | 220 |
| hsa-miR-3944-5p | TGTGCAGCAGGCCAACCG | MIMAT0019231 | hsa-miR-3944-5p | 404 | 278 | 386 | 328 | 306 | 203 | 432 | 322 |
| hsa-miR-3945 | GGGAGGGCATAGGATA | MIMAT0018361 | hsa-miR-3945 | 0 | 0 | 0 | 0 | 0 | 0 | 0 | 0 |
| hsa-miR-3960 | GCGGCGGCGGCGGCGGGGG | MIMAT0019337 | hsa-miR-3960 | 26 | 29 | 62 | 38 | 50 | 39 | 90 | 42 |
| hsa-miR-409-3p | GAATGTTGCTCGGTGAACCCCT | MIMAT0001639 | hsa-miR-409-3p | 8799 | 15831 | 7667 | 14252 | 22642 | 20324 | 40194 | 23031 |
| hsa-miR-409-5p | AGGTTACCCGAGCAACTTTGCATC | MIMAT0001638 | hsa-miR-409-5p | 67 | 142 | 46 | 92 | 84 | 60 | 68 | 84 |
| hsa-miR-410-3p | AATATAACACAGATGGCCTGT | MIMAT0002171 | hsa-miR-410-3p | 881 | 1079 | 940 | 689 | 711 | 550 | 648 | 1000 |
| hsa-miR-410-5p | AGGTTGTCTGTGATGAGTTCG | MIMAT0026558 | hsa-miR-410-5p | 224 | 240 | 206 | 192 | 364 | 417 | 609 | 310 |
| hsa-miR-411-3p | TATGTAACACGGTCCACTAAC | MIMAT0004813 | hsa-miR-411-3p | 896 | 1018 | 1083 | 945 | 401 | 297 | 182 | 674 |
| hsa-miR-411-5p | TAGTAGACCGTATAGCGTACG | MIMAT0003329 | hsa-miR-411-5p | 1808 | 2406 | 1720 | 1611 | 1225 | 848 | 939 | 1637 |
| hsa-miR-412-3p | GTACTTCACCTGGTCCACTAGC | MIMAT0002170 | hsa-miR-412-3p | 497 | 729 | 433 | 377 | 321 | 282 | 193 | 400 |
| hsa-miR-412-5p | TGGTCGACCAGTTGGAAAGTAAT | MIMAT0026557 | hsa-miR-412-5p | 834 | 1560 | 923 | 955 | 798 | 737 | 621 | 986 |
| hsa-miR-421 | ATCAACAGACATTAATTGGGCGC | MIMAT0003339 | hsa-miR-421 | 70 | 58 | 89 | 110 | 109 | 82 | 58 | 95 |
| hsa-miR-422a | ACTGGACTTAGAGTCAGAAGGC | MIMAT0001339 | hsa-miR-422a | 0 | 1 | 0 | 0 | 2 | 1 | 3 | 1 |
| hsa-miR-423-3p | AGCTCGGTCTGAGGCCCCTCAGT | MIMAT0001340 | hsa-miR-423-3p | 337172 | 666013 | 350117 | 568468 | 782352 | 735231 | 1242699 | 847642 |
| hsa-miR-423-5p | TGAGGGGCAGAGAGCGAGACTTT | MIMAT0004748 | hsa-miR-423-5p | 43974 | 121946 | 16144 | 57606 | 117812 | 78008 | 115407 | 123813 |
| hsa-miR-424-3p | CAAAACGTGAGGCGCTGCTAT | MIMAT0004749 | hsa-miR-424-3p | 19395 | 37437 | 19367 | 29484 | 36563 | 29789 | 30149 | 36635 |
| hsa-miR-424-5p | CAGCAGCAATTCATGTTTTG | MIMAT0001341 | hsa-miR-424-5p | 156 | 346 | 132 | 300 | 235 | 180 | 218 | 189 |
| hsa-miR-425-3p | CATCGGGAATGTCGTGTCCGCC | MIMAT0001343 | hsa-miR-425-3p | 828 | 1522 | 929 | 1262 | 1686 | 1684 | 3233 | 1772 |
| hsa-miR-425-5p | AATGACACGATCACTCCCGTTGAGT | MIMAT0003393 | hsa-miR-425-5p | 2960 | 6310 | 2891 | 4174 | 4379 | 3684 | 4482 | 3977 |
| hsa-miR-4254 | GCCTGGAGCTACTCCACCATCT | MIMAT0016884 | hsa-miR-4254 | 0 | 0 | 0 | 0 | 4 | 4 | 14 | 4 |
| hsa-miR-4266 | AGGAGGACTTGGCCAAGG | MIMAT0016892 | hsa-miR-4266 | 1 | 0 | 0 | 0 | 0 | 0 | 0 | 0 |
| hsa-miR-4284 | GACGGGCTCACATCACCC | MIMAT0016915 | hsa-miR-4284 | 0 | 0 | 3 | 0 | 1 | 10 | 0 | 0 |
| hsa-miR-4286 | ACCCCACTCCTGGTACCA | MIMAT0016916 | hsa-miR-4286 | 8 | 66 | 12 | 54 | 32 | 27 | 11 | 28 |
| hsa-miR-429 | TAATACTGTCTGGTAAAACCGT | MIMAT0001536 | hsa-miR-429 | 16 | 9 | 23 | 76 | 34 | 37 | 107 | 34 |
| hsa-miR-4291 | TGGCTTCAGCAGGAACAG | MIMAT0016922 | hsa-miR-4291 | 0 | 0 | 0 | 0 | 0 | 0 | 0 | 0 |
| hsa-miR-4301 | CTCCCACTGCTTCACTTG | MIMAT0016850 | hsa-miR-4301 | 0 | 0 | 0 | 0 | 0 | 1 | 0 | 1 |
| hsa-miR-4302 | CCAGUGUGGCUCAGCGAG | MIMAT0016855 | hsa-miR-4302 | 0 | 0 | 0 | 0 | 2 | 0 | 0 | 0 |
| hsa-miR-431-3p | TTGCAGGTCGTCTTGCAGGGCTTC | MIMAT0004757 | hsa-miR-431-3p | 685 | 1483 | 781 | 1026 | 2187 | 2079 | 4707 | 2413 |
| hsa-miR-431-5p | TGTCTTGCAGGCCGTCATGC | MIMAT0001625 | hsa-miR-431-5p | 2414 | 2346 | 1735 | 1611 | 1938 | 1573 | 2596 | 1968 |
| hsa-miR-432-3p | CTGGATGGCTCCTCCATGTCT | MIMAT0002815 | hsa-miR-432-3p | 38 | 59 | 29 | 40 | 74 | 77 | 170 | 70 |
| hsa-miR-432-5p | TCTTGGAGTAGGTCATTGGGT | MIMAT0002814 | hsa-miR-432-5p | 27542 | 41192 | 23853 | 30445 | 37161 | 29132 | 14583 | 32706 |
| hsa-miR-4323 | AGGAAGCCCCACAGCCTCAG | MIMAT0016875 | hsa-miR-4323 | 0 | 0 | 0 | 0 | 0 | 0 | 0 | 0 |
| hsa-miR-4324 | ACCCTGAGACCCTAACTT | MIMAT0016876 | hsa-miR-4324 | 0 | 0 | 0 | 0 | 0 | 0 | 0 | 0 |
| hsa-miR-4326 | TGTTCCTCTGTCTCCCAGACTCTG | MIMAT0016888 | hsa-miR-4326 | 27 | 97 | 23 | 40 | 64 | 54 | 134 | 62 |
| hsa-miR-433-3p | ATCATGATGGGCTCCTCGGTGT | MIMAT0001627 | hsa-miR-433-3p | 4318 | 7017 | 3933 | 4346 | 5284 | 4714 | 5222 | 5733 |
| hsa-miR-433-5p | TACGGTGAGCCTGTCATTATT | MIMAT0026554 | hsa-miR-433-5p | 5 | 13 | 13 | 8 | 17 | 16 | 36 | 16 |
| hsa-miR-4417 | CGGTGGGCTTCCCGGAGG | NA | hsa-miR-4417 | 0 | 0 | 0 | 0 | 0 | 0 | 0 | 0 |
| hsa-miR-4419b | AGGCTGAAGGAAGATGGC | NA | hsa-miR-4419b | 0 | 0 | 0 | 0 | 0 | 0 | 0 | 0 |
| hsa-miR-4420 | GTCACTGATGTCTGTAGCTG | MIMAT0018933 | hsa-miR-4420 | 0 | 0 | 0 | 0 | 0 | 0 | 0 | 0 |
| hsa-miR-4421 | CCTGTCTGTGGAAAGGAGC | MIMAT0018934 | hsa-miR-4421 | 2 | 1 | 5 | 1 | 0 | 0 | 0 | 0 |
| hsa-miR-4422 | AAAAGCATCAGGAAGTACCCAC | MIMAT0018935 | hsa-miR-4422 | 0 | 0 | 0 | 0 | 3 | 1 | 0 | 0 |
| hsa-miR-4423-5p | AGTTGCCTTTTTGTTCCCATGC | MIMAT0019232 | hsa-miR-4423-5p | 264 | 442 | 248 | 381 | 360 | 321 | 315 | 331 |
| hsa-miR-4425 | UGUUGGGAUUCAGCAGGACCAU | MIMAT0018940 | hsa-miR-4425 | 0 | 0 | 0 | 0 | 0 | 0 | 0 | 0 |
| hsa-miR-4426 | GCTGGAAGATGGACGTACTTTG | MIMAT0018941 | hsa-miR-4426 | 0 | 0 | 0 | 0 | 0 | 0 | 0 | 0 |
| hsa-miR-4429 | AAAAGCTGGGTTGAGAGGCGA | MIMAT0018944 | hsa-miR-4429 | 4 | 8 | 4 | 7 | 17 | 13 | 17 | 12 |
| hsa-miR-4433a-3p | ACAGGAGTGGGGGTGGGACA | MIMAT0018949 | hsa-miR-4433a-3p | 0 | 0 | 0 | 0 | 0 | 0 | 0 | 0 |
| hsa-miR-4433a-5p | CGTCCCACCCCCCACTCCTGTT | MIMAT0020956 | hsa-miR-4433a-5p | 0 | 0 | 0 | 0 | 0 | 0 | 0 | 0 |
| hsa-miR-4433b-5p | ATGTCCCACCCCCACTCCTGTT | MIMAT0030413 | hsa-miR-4433b-5p | 0 | 1 | 0 | 2 | 7 | 4 | 14 | 4 |
| hsa-miR-4435 | ATGGCCAGAGCTCACACAGAGG | MIMAT0018951 | hsa-miR-4435 | 569 | 329 | 382 | 209 | 211 | 158 | 140 | 187 |
| hsa-miR-4436b-3p | CAGGGCAGGAAGAAGTGGAC | MIMAT0019941 | hsa-miR-4436b-3p | 5 | 16 | 7 | 14 | 14 | 0 | 0 | 0 |
| hsa-miR-4436b-5p | TCCACTTCTGCCTGCCCTGCC | MIMAT0019940 | hsa-miR-4436b-5p | 12 | 7 | 12 | 5 | 8 | 6 | 13 | 15 |
| hsa-miR-4437 | TTGTGGGCTCAGGGTACAAAGG | MIMAT0018953 | hsa-miR-4437 | 4 | 4 | 16 | 4 | 0 | 0 | 0 | 0 |
| hsa-miR-4439 | GTGACTGATACCTTGGAGGCA | MIMAT0018957 | hsa-miR-4439 | 0 | 0 | 0 | 0 | 0 | 0 | 0 | 0 |
| hsa-miR-4440 | TGTCGTGGGGCTTGCTGGCTTG | MIMAT0018958 | hsa-miR-4440 | 39 | 92 | 58 | 47 | 61 | 52 | 75 | 46 |
| hsa-miR-4442 | TATGCCGGACAAGAGGGAGGTG | MIMAT0018960 | hsa-miR-4442 | 0 | 0 | 0 | 0 | 0 | 0 | 0 | 0 |
| hsa-miR-4443 | CTTGGAGGCGTGGGTTTT | MIMAT0018961 | hsa-miR-4443 | 19 | 53 | 10 | 27 | 75 | 81 | 137 | 62 |
| hsa-miR-4446-3p | CAGGGCTGGCAGTGACATGGGT | MIMAT0018965 | hsa-miR-4446-3p | 2 | 0 | 0 | 0 | 0 | 0 | 0 | 0 |
| hsa-miR-4446-5p | CATTTCCCTGCCATTCCCTTGGTT | MIMAT0019233 | hsa-miR-4446-5p | 0 | 0 | 0 | 0 | 0 | 0 | 0 | 0 |
| hsa-miR-4447 | TGGTGTGGGCTGTTGTTT | MIMAT0018966 | hsa-miR-4447 | 0 | 0 | 0 | 0 | 0 | 0 | 2 | 0 |
| hsa-miR-4448 | GGCTCGTTGGTCTAGGGG | MIMAT0018967 | hsa-miR-4448 | 1349 | 606 | 1476 | 1106 | 1628 | 2012 | 2877 | 1640 |
| hsa-miR-4449 | CGGGGCTGCGCGAGGCACAGGC | MIMAT0018968 | hsa-miR-4449 | 3 | 6 | 4 | 1 | 0 | 0 | 0 | 0 |
| hsa-miR-4450 | TGGGGATTTGGAGAAGTGGTG | MIMAT0018971 | hsa-miR-4450 | 4 | 4 | 2 | 4 | 4 | 2 | 0 | 0 |
| hsa-miR-4451 | AGCTGGTAGAGCTGAGGAC | MIMAT0018973 | hsa-miR-4451 | 0 | 0 | 2 | 0 | 4 | 4 | 15 | 8 |
| hsa-miR-4452 | UUGAAUUCUUGGCCUUAAGUGAU | MIMAT0018974 | hsa-miR-4452 | 0 | 0 | 0 | 0 | 0 | 0 | 0 | 0 |
| hsa-miR-4453 | TGGAGAGCTTGGTCTGTAGC | MIMAT0018975 | hsa-miR-4453 | 0 | 0 | 0 | 0 | 0 | 0 | 0 | 0 |
| hsa-miR-4454 | TCGGATCCGAGTCACGGCACC | MIMAT0018976 | hsa-miR-4454 | 16 | 7 | 11 | 45 | 36 | 28 | 41 | 23 |
| hsa-miR-4455 | TAGGGTGTGTGTGTTTTT | MIMAT0018977 | hsa-miR-4455 | 5 | 3 | 1 | 1 | 7 | 2 | 2 | 6 |
| hsa-miR-4458 | GAGGTAGGTGTGGAAAAA | MIMAT0018980 | hsa-miR-4458 | 23 | 10 | 36 | 24 | 23 | 30 | 19 | 47 |
| hsa-miR-4459 | CCAGGAGGCGGAGGAGGTGGAGGA | NA | hsa-miR-4459 | 0 | 0 | 0 | 0 | 0 | 0 | 0 | 0 |
| hsa-miR-4461 | GATTGAGACTAGTAGGGCTAGGCC | NA | hsa-miR-4461 | 64 | 108 | 60 | 75 | 81 | 36 | 11 | 90 |
| hsa-miR-4463 | GAGACTGGGGTGGGGCCT | MIMAT0018987 | hsa-miR-4463 | 31 | 48 | 27 | 27 | 35 | 27 | 62 | 70 |
| hsa-miR-4466 | GGGTGCGGGCCGGCGGGGT | MIMAT0018993 | hsa-miR-4466 | 225 | 248 | 295 | 274 | 407 | 448 | 897 | 394 |
| hsa-miR-4467 | TGGCGGCGGTAGTTATGGGCTTCT | MIMAT0018994 | hsa-miR-4467 | 32 | 69 | 32 | 39 | 50 | 55 | 21 | 54 |
| hsa-miR-4469 | GCTCCCTCTAGGGTCGCTCGGT | MIMAT0018996 | hsa-miR-4469 | 3 | 1 | 6 | 1 | 0 | 0 | 0 | 0 |
| hsa-miR-4470 | TGGCAAACGTGGAAGCCGAGAGGT | MIMAT0018997 | hsa-miR-4470 | 4 | 1 | 6 | 1 | 0 | 3 | 0 | 0 |
| hsa-miR-4471 | TGGGAACTTAGTAGAGGTTTA | MIMAT0018998 | hsa-miR-4471 | 0 | 0 | 0 | 0 | 0 | 0 | 0 | 0 |
| hsa-miR-4473 | TAGTGCTCTCCGTTACAAGTAT | MIMAT0019000 | hsa-miR-4473 | 31 | 21 | 10 | 14 | 15 | 12 | 0 | 11 |
| hsa-miR-4474-3p | TTGTGGCTGGTCATGAGGCT | MIMAT0019001 | hsa-miR-4474-3p | 4 | 6 | 4 | 1 | 2 | 16 | 0 | 6 |
| hsa-miR-4478 | AGGCTGAGCTGAGGAGTC | MIMAT0019006 | hsa-miR-4478 | 0 | 0 | 0 | 0 | 0 | 0 | 0 | 0 |
| hsa-miR-4479 | CGCGCGGCCGTGCTCGGAGC | MIMAT0019011 | hsa-miR-4479 | 49 | 62 | 59 | 47 | 95 | 85 | 231 | 114 |
| hsa-miR-4482-3p | TTTCTATTTCTCAGTGGGGCTCT | MIMAT0020958 | hsa-miR-4482-3p | 0 | 0 | 0 | 0 | 0 | 0 | 0 | 0 |
| hsa-miR-4483 | GGGGTGGTCTGTTGTTTT | MIMAT0019017 | hsa-miR-4483 | 0 | 0 | 0 | 0 | 0 | 0 | 0 | 2 |
| hsa-miR-4484 | AAAAAAGGCGGGAGAAGC | MIMAT0019018 | hsa-miR-4484 | 21 | 26 | 40 | 31 | 40 | 29 | 64 | 33 |
| hsa-miR-4485-3p | TGTTTAACGGCCGCGGTACCC | MIMAT0019019 | hsa-miR-4485-3p | 10273 | 18838 | 8544 | 7464 | 4471 | 3072 | 4356 | 9489 |
| hsa-miR-4485-5p | ACCGCCTGCCCAGTGACAC | MIMAT0032116 | hsa-miR-4485-5p | 80 | 161 | 98 | 115 | 102 | 48 | 90 | 106 |
| hsa-miR-4488 | GAGGGGGCGGGCTCCGGCG | MIMAT0019022 | hsa-miR-4488 | 7 | 17 | 24 | 33 | 36 | 18 | 46 | 40 |
| hsa-miR-4489 | TGGGGCTAGTGATGCAGGACG | MIMAT0019023 | hsa-miR-4489 | 4 | 0 | 3 | 5 | 6 | 6 | 13 | 3 |
| hsa-miR-4497 | GCTCCGGGACGGCTGGGC | MIMAT0019032 | hsa-miR-4497 | 0 | 0 | 0 | 0 | 0 | 0 | 0 | 0 |
| hsa-miR-4498 | TGGGCTGGCAGGGCAAGTGC | MIMAT0019033 | hsa-miR-4498 | 0 | 0 | 0 | 0 | 4 | 1 | 0 | 6 |
| hsa-miR-449a | TGGCAGTGTATTGTTAGCTGGT | MIMAT0001541 | hsa-miR-449a | 608 | 336 | 783 | 295 | 138 | 148 | 227 | 123 |
| hsa-miR-449b-3p | CAGCCACAACTACCCTGCCAC | MIMAT0009203 | hsa-miR-449b-3p | 6 | 1 | 6 | 1 | 2 | 2 | 0 | 2 |
| hsa-miR-449b-5p | AGGCAGTGTATTGTTAGCTGGCTG | MIMAT0003327 | hsa-miR-449b-5p | 20 | 14 | 32 | 9 | 4 | 2 | 1 | 0 |
| hsa-miR-449c-3p | AGTAGCTAGTTGCACTCCTCTC | MIMAT0013771 | hsa-miR-449c-3p | 0 | 0 | 0 | 0 | 0 | 0 | 0 | 0 |
| hsa-miR-449c-5p | AGGCAGTGTATTGCTAGCGGCTGTT | MIMAT0010251 | hsa-miR-449c-5p | 25 | 15 | 27 | 17 | 10 | 12 | 6 | 9 |
| hsa-miR-4501 | TATGTGACCTCGGATGAATCA | MIMAT0019037 | hsa-miR-4501 | 0 | 0 | 0 | 0 | 0 | 0 | 0 | 0 |
| hsa-miR-4502 | GGCTGATGATGATGGTGCTGAAGG | MIMAT0019038 | hsa-miR-4502 | 10 | 4 | 3 | 0 | 0 | 2 | 0 | 0 |
| hsa-miR-4506 | AAATGGGTGGTCTGAGGCAAGTT | MIMAT0019042 | hsa-miR-4506 | 0 | 0 | 0 | 0 | 0 | 0 | 0 | 3 |
| hsa-miR-4507 | CTGGGTTGGGTTGGGC | MIMAT0019044 | hsa-miR-4507 | 2 | 2 | 7 | 4 | 2 | 2 | 7 | 2 |
| hsa-miR-4508 | AAGCGGGGCTGGGCGCGC | MIMAT0019045 | hsa-miR-4508 | 2 | 2 | 7 | 18 | 1 | 3 | 2 | 6 |
| hsa-miR-450a-1-3p | ATTGGGAACATTTTGCATGTAT | MIMAT0022700 | hsa-miR-450a-1-3p | 0 | 1 | 0 | 0 | 0 | 0 | 0 | 0 |
| hsa-miR-450a-2-3p | TTGGGGACATTTTGCATTCAT | MIMAT0031074 | hsa-miR-450a-2-3p | 36 | 57 | 60 | 61 | 37 | 28 | 19 | 28 |
| hsa-miR-450a-5p | TTTTGCGATGTGTTCCTAAT | MIMAT0001545 | hsa-miR-450a-5p | 24 | 51 | 34 | 43 | 40 | 23 | 25 | 32 |
| hsa-miR-450b-5p | TTTTGCAATATGTTCCTGAAT | MIMAT0004909 | hsa-miR-450b-5p | 1 | 0 | 3 | 0 | 1 | 0 | 0 | 0 |
| hsa-miR-4510 | TGAGGGAGTAGGTTGTATGGTT | MIMAT0019047 | hsa-miR-4510 | 23 | 16 | 21 | 25 | 14 | 11 | 1 | 36 |
| hsa-miR-4511 | GAAGAACUGUUGCAUUUGCCCU | MIMAT0019048 | hsa-miR-4511 | 0 | 0 | 0 | 0 | 0 | 0 | 0 | 0 |
| hsa-miR-4512 | CAGGGCCTCACTGTATCGCC | MIMAT0019049 | hsa-miR-4512 | 10 | 21 | 26 | 16 | 30 | 38 | 60 | 27 |
| hsa-miR-4514 | GACGGGCAGGATTGGGGA | MIMAT0019051 | hsa-miR-4514 | 0 | 0 | 0 | 0 | 0 | 0 | 0 | 0 |
| hsa-miR-4515 | AGGACTGGACTCCCGGCAGC | MIMAT0019052 | hsa-miR-4515 | 0 | 0 | 0 | 0 | 0 | 0 | 0 | 0 |
| hsa-miR-4516 | GGAGAAGGGTCGGGGCGG | MIMAT0019053 | hsa-miR-4516 | 27 | 17 | 18 | 18 | 26 | 16 | 50 | 29 |
| hsa-miR-4517 | TAAATATGATGAAACTCACAGCTGAGG | MIMAT0019054 | hsa-miR-4517 | 0 | 0 | 0 | 0 | 0 | 0 | 0 | 0 |
| hsa-miR-451a | AAACCGTTACCATTACTGAGT | MIMAT0001631 | hsa-miR-451a | 3679 | 3206 | 4569 | 2378 | 9497 | 74021 | 11980 | 5166 |
| hsa-miR-452-5p | AACUGUUUGCAGAGGAAACUGA | MIMAT0001635 | hsa-miR-452-5p | 11 | 23 | 5 | 20 | 25 | 23 | 28 | 13 |
| hsa-miR-4521 | GCTAAGGAAGTCCTGTGCTCAGTTT | MIMAT0019058 | hsa-miR-4521 | 87 | 268 | 104 | 178 | 189 | 155 | 123 | 174 |
| hsa-miR-4522 | CCGCTGACTCTGCCTGTAGGC | MIMAT0019060 | hsa-miR-4522 | 0 | 0 | 0 | 0 | 0 | 0 | 0 | 0 |
| hsa-miR-4523 | GACCGAGAGGGCCTCGGCTGTTT | MIMAT0019061 | hsa-miR-4523 | 0 | 0 | 0 | 2 | 3 | 0 | 0 | 0 |
| hsa-miR-4524a-3p | TGAGACAGGCTTATGCTGCTAT | MIMAT0019063 | hsa-miR-4524a-3p | 56 | 103 | 66 | 143 | 138 | 115 | 169 | 124 |
| hsa-miR-4524a-5p | AGCAGCATGAACCTGTCTCACT | MIMAT0019062 | hsa-miR-4524a-5p | 2 | 9 | 2 | 11 | 8 | 10 | 0 | 10 |
| hsa-miR-4524b-3p | GAGACAGGUUCAUGCUGCUA | MIMAT0022256 | hsa-miR-4524b-3p | 0 | 0 | 0 | 0 | 0 | 0 | 0 | 0 |
| hsa-miR-4525 | GGGGGATGTGCATGCTGGT | MIMAT0019064 | hsa-miR-4525 | 47 | 58 | 61 | 39 | 44 | 49 | 12 | 54 |
| hsa-miR-4526 | AGGGCTGGCCGCGAACGT | MIMAT0019065 | hsa-miR-4526 | 0 | 0 | 0 | 0 | 0 | 0 | 0 | 0 |
| hsa-miR-4529-3p | ATTGGACTGCTGATGGCCTGTC | MIMAT0019068 | hsa-miR-4529-3p | 0 | 2 | 0 | 0 | 0 | 0 | 0 | 0 |
| hsa-miR-4529-5p | AGGCCAUCAGCAGUCCAAUGAA | MIMAT0019236 | hsa-miR-4529-5p | 0 | 0 | 0 | 0 | 0 | 0 | 0 | 0 |
| hsa-miR-4532 | CCCGGGGAGCCCGGCGGG | NA | hsa-miR-4532 | 0 | 1 | 2 | 0 | 0 | 2 | 1 | 1 |
| hsa-miR-4535 | AGTGGACCTGGCTGGGAC | MIMAT0019075 | hsa-miR-4535 | 0 | 0 | 0 | 0 | 0 | 0 | 0 | 0 |
| hsa-miR-4536-3p | TATCGTGCATATATCTACCACA | MIMAT0020959 | hsa-miR-4536-3p | 0 | 0 | 0 | 0 | 0 | 0 | 0 | 0 |
| hsa-miR-4536-5p | UGUGGUAGAUAUAUGCACGAU | MIMAT0019078 | hsa-miR-4536-5p | 0 | 0 | 0 | 0 | 0 | 0 | 0 | 0 |
| hsa-miR-454-3p | TAGTGCAATATTGCTTATAGGGTT | MIMAT0003885 | hsa-miR-454-3p | 28 | 81 | 16 | 53 | 35 | 36 | 2 | 24 |
| hsa-miR-454-5p | ACCCTATCAATATTGTCTCTGCT | MIMAT0003884 | hsa-miR-454-5p | 3 | 0 | 1 | 1 | 8 | 4 | 14 | 4 |
| hsa-miR-455-3p | GCAGTCCATGGGCATATACACC | MIMAT0004784 | hsa-miR-455-3p | 13472 | 29294 | 11923 | 23391 | 22254 | 18145 | 14165 | 16611 |
| hsa-miR-455-5p | TATGTGCCTTTGGACTACATCGT | MIMAT0003150 | hsa-miR-455-5p | 81 | 78 | 53 | 82 | 92 | 81 | 93 | 86 |
| hsa-miR-4632-3p | TGCCGCCCTCTCGCTGCTCTAG | MIMAT0019688 | hsa-miR-4632-3p | 0 | 0 | 0 | 0 | 0 | 0 | 0 | 0 |
| hsa-miR-4632-5p | GAGGGCAGCGTGGGTGTGGCGG | MIMAT0022977 | hsa-miR-4632-5p | 0 | 2 | 0 | 0 | 0 | 0 | 0 | 0 |
| hsa-miR-4635 | GTCTTGAAGTCAGAACCCGCAAT | MIMAT0019692 | hsa-miR-4635 | 0 | 0 | 0 | 0 | 0 | 0 | 0 | 0 |
| hsa-miR-4636 | AACTCGTGTTCAAAGCCTTT | MIMAT0019693 | hsa-miR-4636 | 2 | 23 | 6 | 15 | 4 | 7 | 0 | 8 |
| hsa-miR-4638-3p | CCTGGACACCGCTCAGCCGGCCG | MIMAT0019696 | hsa-miR-4638-3p | 17 | 18 | 7 | 11 | 16 | 14 | 0 | 14 |
| hsa-miR-4638-5p | ACTCGGCTGCGGTGGACAAGG | MIMAT0019695 | hsa-miR-4638-5p | 30 | 22 | 47 | 25 | 8 | 8 | 18 | 6 |
| hsa-miR-4640-3p | CACCCCCTGTTTCCTGGCCCACT | MIMAT0019700 | hsa-miR-4640-3p | 15 | 36 | 21 | 42 | 51 | 43 | 44 | 36 |
| hsa-miR-4640-5p | CCAGGGAGCAGCTGGTGGGTG | MIMAT0019699 | hsa-miR-4640-5p | 2 | 7 | 2 | 2 | 12 | 8 | 22 | 10 |
| hsa-miR-4641 | TGCCCATGCCATACTTTTGCCTC | MIMAT0019701 | hsa-miR-4641 | 0 | 7 | 0 | 5 | 5 | 5 | 15 | 8 |
| hsa-miR-4642 | ATGGCATCGTCCCCTGGTGGC | MIMAT0019702 | hsa-miR-4642 | 1 | 7 | 0 | 2 | 3 | 2 | 0 | 2 |
| hsa-miR-4644 | TGGAGAGAGAAAAGAGAC | MIMAT0019704 | hsa-miR-4644 | 2 | 0 | 0 | 0 | 0 | 0 | 0 | 5 |
| hsa-miR-4645-3p | AGACAGTAGTTCTTGCCTGGTT | MIMAT0019706 | hsa-miR-4645-3p | 0 | 4 | 0 | 0 | 11 | 17 | 20 | 13 |
| hsa-miR-4646-3p | TTGTCCCTCTCCCTTCCCAG | MIMAT0019708 | hsa-miR-4646-3p | 0 | 0 | 2 | 0 | 0 | 3 | 0 | 1 |
| hsa-miR-4646-5p | ACTGGGAAGAGGAGCTGAGGGAC | MIMAT0019707 | hsa-miR-4646-5p | 0 | 0 | 0 | 0 | 0 | 1 | 0 | 0 |
| hsa-miR-4647 | GAAGATGGTGCTGTGCTGAGG | MIMAT0019709 | hsa-miR-4647 | 172 | 116 | 172 | 97 | 114 | 102 | 84 | 83 |
| hsa-miR-4648 | TGTGGGACTGCAAATGGGAG | MIMAT0019710 | hsa-miR-4648 | 0 | 2 | 0 | 0 | 0 | 0 | 0 | 0 |
| hsa-miR-4649-3p | CTGCTCTGAGGCCTGCCTCTC | MIMAT0019712 | hsa-miR-4649-3p | 0 | 0 | 1 | 3 | 5 | 5 | 18 | 7 |
| hsa-miR-4649-5p | TGGGCGAGGGGTGGGCTCTCAG | MIMAT0019711 | hsa-miR-4649-5p | 8 | 8 | 6 | 5 | 13 | 8 | 3 | 17 |
| hsa-miR-4650-5p | TATCAGGCCTCTTTCTACCTTC | MIMAT0019713 | hsa-miR-4650-5p | 0 | 0 | 0 | 0 | 0 | 0 | 0 | 0 |
| hsa-miR-4651 | CGGGGTGGGTGAGGTCGGG | MIMAT0019715 | hsa-miR-4651 | 0 | 8 | 0 | 1 | 8 | 10 | 20 | 6 |
| hsa-miR-4653-3p | TGGAGTTAAGGGTTGCT | MIMAT0019719 | hsa-miR-4653-3p | 10 | 0 | 3 | 3 | 8 | 2 | 0 | 0 |
| hsa-miR-4654 | TGTGGGATCTGGAGGCATC | MIMAT0019720 | hsa-miR-4654 | 0 | 0 | 0 | 0 | 0 | 0 | 0 | 0 |
| hsa-miR-4655-5p | ACACCGGGGATGGCAGAGGGT | MIMAT0019721 | hsa-miR-4655-5p | 19 | 28 | 43 | 15 | 9 | 3 | 0 | 18 |
| hsa-miR-4656 | TGGGCTGAGGGCAGGAGGC | MIMAT0019723 | hsa-miR-4656 | 0 | 0 | 0 | 0 | 0 | 0 | 0 | 0 |
| hsa-miR-4657 | AATGTGGAAGTGGTCTGAGGC | MIMAT0019724 | hsa-miR-4657 | 2 | 8 | 8 | 6 | 11 | 10 | 17 | 12 |
| hsa-miR-4658 | GTGAGTGTGGATCCTGGAGG | MIMAT0019725 | hsa-miR-4658 | 0 | 0 | 0 | 2 | 1 | 0 | 0 | 2 |
| hsa-miR-4659a-3p | TTTCTTCTTAGACATGGCAACG | MIMAT0019727 | hsa-miR-4659a-3p | 142 | 60 | 84 | 59 | 43 | 30 | 1 | 17 |
| hsa-miR-4659b-3p | UUUCUUCUUAGACAUGGCAGCU | MIMAT0019734 | hsa-miR-4659b-3p | 0 | 0 | 0 | 0 | 0 | 1 | 0 | 0 |
| hsa-miR-466 | AUACACAUACACGCAACACACAU | MIMAT0015002 | hsa-miR-466 | 0 | 0 | 0 | 0 | 0 | 0 | 0 | 0 |
| hsa-miR-4660 | TGCAGCTCTGGTGGAAAA | MIMAT0019728 | hsa-miR-4660 | 5 | 8 | 3 | 2 | 2 | 5 | 0 | 0 |
| hsa-miR-4661-5p | AACTAGCTCTGTGGATCCTGAC | MIMAT0019729 | hsa-miR-4661-5p | 20 | 20 | 16 | 12 | 21 | 14 | 14 | 17 |
| hsa-miR-4662a-5p | TTAGCCAATTGTCCATCTTTAG | MIMAT0019731 | hsa-miR-4662a-5p | 0 | 3 | 3 | 0 | 4 | 0 | 0 | 0 |
| hsa-miR-4663 | AGCTGAGCTCCATGGACGTGC | MIMAT0019735 | hsa-miR-4663 | 1 | 3 | 0 | 0 | 6 | 7 | 21 | 6 |
| hsa-miR-4664-3p | CTTCCGGTCTGTGAGCCCCGTC | MIMAT0019738 | hsa-miR-4664-3p | 1 | 7 | 5 | 3 | 10 | 10 | 4 | 15 |
| hsa-miR-4664-5p | TGGGGTGCCCACTCCGCAAGTTT | MIMAT0019737 | hsa-miR-4664-5p | 0 | 0 | 0 | 1 | 3 | 7 | 10 | 7 |
| hsa-miR-4665-3p | CCGCGGCGCGTAGCCCCCGCC | MIMAT0019740 | hsa-miR-4665-3p | 0 | 4 | 0 | 0 | 0 | 0 | 0 | 0 |
| hsa-miR-4665-5p | CTGGGGGACGCGTGAGCGCGAGC | MIMAT0019739 | hsa-miR-4665-5p | 40 | 62 | 35 | 52 | 96 | 79 | 191 | 103 |
| hsa-miR-4667-3p | TCCCTCCTTCTGTCCCCACAG | MIMAT0019744 | hsa-miR-4667-3p | 0 | 2 | 0 | 0 | 0 | 0 | 0 | 2 |
| hsa-miR-4667-5p | GACTGGGGAGCAGAAGGAGAAC | MIMAT0019743 | hsa-miR-4667-5p | 3 | 0 | 0 | 2 | 13 | 4 | 9 | 5 |
| hsa-miR-4668-5p | AGGGAAAAAAAAAAGGATTTGTC | MIMAT0019745 | hsa-miR-4668-5p | 0 | 0 | 0 | 3 | 5 | 4 | 15 | 5 |
| hsa-miR-4669 | TGTGTCCGGGAAGTGGAGGAGT | MIMAT0019749 | hsa-miR-4669 | 36 | 37 | 50 | 23 | 45 | 37 | 48 | 34 |
| hsa-miR-4670-3p | TACATCATGGTCGCTTCCT | MIMAT0019751 | hsa-miR-4670-3p | 0 | 0 | 0 | 0 | 0 | 0 | 0 | 1 |
| hsa-miR-4670-5p | AGCGACCATGATGTAACTTCAG | MIMAT0019750 | hsa-miR-4670-5p | 0 | 2 | 0 | 2 | 0 | 0 | 0 | 0 |
| hsa-miR-4672 | TTACACAGCTGGACAGAGGCACG | MIMAT0019754 | hsa-miR-4672 | 0 | 0 | 0 | 0 | 0 | 0 | 0 | 0 |
| hsa-miR-4674 | CTGGGCTCGGGACGCGCGGCT | MIMAT0019756 | hsa-miR-4674 | 3 | 4 | 0 | 4 | 4 | 0 | 0 | 4 |
| hsa-miR-4676-3p | ACTGTTTCACCACTGGCTCT | MIMAT0019759 | hsa-miR-4676-3p | 0 | 1 | 0 | 0 | 0 | 0 | 0 | 1 |
| hsa-miR-4676-5p | AGAGCCAGTGGTGAGACAGTG | MIMAT0019758 | hsa-miR-4676-5p | 22 | 36 | 18 | 22 | 40 | 25 | 25 | 26 |
| hsa-miR-4677-3p | TCTGTGAGACCAAAGAACTACT | MIMAT0019761 | hsa-miR-4677-3p | 0 | 5 | 0 | 0 | 0 | 0 | 0 | 1 |
| hsa-miR-4677-5p | TTGTTCTTTGGTCTTTCAGCC | MIMAT0019760 | hsa-miR-4677-5p | 7 | 29 | 8 | 8 | 12 | 11 | 16 | 15 |
| hsa-miR-4681 | AACGGGAATGCAGGCTGTATCT | MIMAT0019766 | hsa-miR-4681 | 0 | 5 | 0 | 0 | 0 | 0 | 0 | 0 |
| hsa-miR-4682 | TCTGAGTTCCTGGAGCCTGGTCT | MIMAT0019767 | hsa-miR-4682 | 3 | 4 | 2 | 8 | 6 | 4 | 5 | 5 |
| hsa-miR-4683 | TGGAGATCCAGTGCTCGCCCGAT | MIMAT0019768 | hsa-miR-4683 | 59 | 96 | 67 | 93 | 126 | 134 | 260 | 117 |
| hsa-miR-4684-3p | TGTTGCAAGTCGGTGGAGACGT | MIMAT0019770 | hsa-miR-4684-3p | 0 | 4 | 0 | 0 | 5 | 3 | 12 | 7 |
| hsa-miR-4684-5p | CTCTCTACTGACTTGCAACAT | MIMAT0019769 | hsa-miR-4684-5p | 0 | 4 | 0 | 0 | 0 | 0 | 0 | 0 |
| hsa-miR-4685-3p | TCTCCCTTCCTGCCCTGGCT | MIMAT0019772 | hsa-miR-4685-3p | 109 | 231 | 141 | 144 | 212 | 213 | 449 | 206 |
| hsa-miR-4685-5p | CAGGGCTTGGAGTGGGGCAAGG | MIMAT0019771 | hsa-miR-4685-5p | 0 | 0 | 0 | 1 | 0 | 0 | 0 | 0 |
| hsa-miR-4687-3p | TGGCTGTTGGAGGGGGCAGGC | MIMAT0019775 | hsa-miR-4687-3p | 111 | 64 | 35 | 19 | 47 | 58 | 118 | 78 |
| hsa-miR-4687-5p | CAGCCCTCCTCCCGCACCCAA | MIMAT0019774 | hsa-miR-4687-5p | 1 | 4 | 0 | 0 | 1 | 2 | 0 | 2 |
| hsa-miR-4688 | TAGGGGCAGCAGAGGACCTGGG | MIMAT0019777 | hsa-miR-4688 | 4 | 4 | 15 | 4 | 2 | 2 | 7 | 2 |
| hsa-miR-4689 | TTGAGGAGACATGGTGGGGGC | MIMAT0019778 | hsa-miR-4689 | 44 | 36 | 34 | 15 | 29 | 22 | 28 | 30 |
| hsa-miR-4690-5p | AGGCTGGGCTGAACCCGT | MIMAT0019779 | hsa-miR-4690-5p | 0 | 0 | 0 | 0 | 0 | 0 | 0 | 0 |
| hsa-miR-4691-5p | GTCCTCCAGGCCATGAGCTGCGG | MIMAT0019781 | hsa-miR-4691-5p | 0 | 0 | 0 | 0 | 0 | 0 | 0 | 2 |
| hsa-miR-4695-3p | TGATCTCACCGCTGCCTCCTTCT | MIMAT0019789 | hsa-miR-4695-3p | 27 | 45 | 20 | 26 | 54 | 36 | 50 | 64 |
| hsa-miR-4695-5p | AGGCAGTGAGCGAGCAGGC | MIMAT0019788 | hsa-miR-4695-5p | 0 | 0 | 0 | 0 | 0 | 0 | 0 | 0 |
| hsa-miR-4697-3p | TGTCAGTGACTCCTGCCCCTTGGTT | MIMAT0019792 | hsa-miR-4697-3p | 0 | 0 | 0 | 2 | 0 | 0 | 0 | 0 |
| hsa-miR-4697-5p | AGGGGGCGCAGTCACTGACGTG | MIMAT0019791 | hsa-miR-4697-5p | 3 | 0 | 0 | 0 | 0 | 0 | 0 | 0 |
| hsa-miR-4700-5p | UCUGGGGAUGAGGACAGUGUGU | MIMAT0019796 | hsa-miR-4700-5p | 0 | 0 | 0 | 0 | 0 | 0 | 1 | 0 |
| hsa-miR-4701-3p | TGGGTGATGGGTGTGGTGTCCAC | MIMAT0019799 | hsa-miR-4701-3p | 31 | 44 | 27 | 24 | 55 | 40 | 96 | 49 |
| hsa-miR-4701-5p | TTGGCCACCACACCTACCCCTT | MIMAT0019798 | hsa-miR-4701-5p | 9 | 27 | 8 | 20 | 20 | 14 | 13 | 20 |
| hsa-miR-4704-3p | TCAGTCACATACCTAGTGTCT | MIMAT0019804 | hsa-miR-4704-3p | 0 | 0 | 0 | 1 | 0 | 0 | 0 | 0 |
| hsa-miR-4704-5p | GACACTAGGCATGTGAGTGAT | MIMAT0019803 | hsa-miR-4704-5p | 0 | 0 | 0 | 0 | 0 | 0 | 0 | 0 |
| hsa-miR-4706 | AGCGGGGAGGAAGTGGGCGCTGCTT | MIMAT0019806 | hsa-miR-4706 | 9 | 24 | 6 | 18 | 34 | 27 | 82 | 37 |
| hsa-miR-4707-3p | AGCCCGCCCCAGCCGAGGTTCT | MIMAT0019808 | hsa-miR-4707-3p | 2 | 0 | 0 | 0 | 0 | 0 | 0 | 0 |
| hsa-miR-4707-5p | GCCCCGGCGCGGGCGGGTTC | MIMAT0019807 | hsa-miR-4707-5p | 0 | 0 | 0 | 0 | 0 | 0 | 0 | 0 |
| hsa-miR-4708-3p | AGCAAGGCGGCATCTCTCTGAT | MIMAT0019810 | hsa-miR-4708-3p | 0 | 2 | 0 | 2 | 17 | 0 | 0 | 1 |
| hsa-miR-4708-5p | AGAGATGCCGCCTTGCTCCTT | MIMAT0019809 | hsa-miR-4708-5p | 2 | 0 | 0 | 0 | 0 | 0 | 0 | 0 |
| hsa-miR-4709-3p | TTGAAGAGGAGGTGCTCTGTAGC | MIMAT0019812 | hsa-miR-4709-3p | 0 | 0 | 0 | 4 | 8 | 7 | 26 | 7 |
| hsa-miR-4709-5p | ACAACAGTGACTTGCTCTCCA | MIMAT0019811 | hsa-miR-4709-5p | 0 | 0 | 0 | 2 | 3 | 2 | 0 | 0 |
| hsa-miR-4710 | GGGTGAGGGCAGGTGGTT | MIMAT0019815 | hsa-miR-4710 | 1 | 18 | 4 | 7 | 4 | 3 | 6 | 4 |
| hsa-miR-4711-3p | TCTCGTGTCTTCTGGCTTGATT | MIMAT0019817 | hsa-miR-4711-3p | 0 | 0 | 0 | 0 | 0 | 0 | 0 | 3 |
| hsa-miR-4711-5p | TGCATCAGGCCAGAAGACATGAGT | MIMAT0019816 | hsa-miR-4711-5p | 0 | 0 | 0 | 4 | 0 | 0 | 0 | 0 |
| hsa-miR-4712-3p | AAATGAGAGACCTGTACTGTAT | MIMAT0019819 | hsa-miR-4712-3p | 0 | 0 | 0 | 0 | 0 | 0 | 0 | 0 |
| hsa-miR-4713-5p | TTCTCCCACTACCAGGCTCCCAT | MIMAT0019820 | hsa-miR-4713-5p | 0 | 0 | 0 | 0 | 0 | 0 | 0 | 0 |
| hsa-miR-4714-3p | CCAACCTAGGTGGTCAGAGTTG | MIMAT0019823 | hsa-miR-4714-3p | 0 | 0 | 0 | 0 | 8 | 6 | 20 | 8 |
| hsa-miR-4715-3p | CACCTTAACTGCAGCCAATTC | MIMAT0019825 | hsa-miR-4715-3p | 2 | 1 | 5 | 2 | 0 | 2 | 0 | 0 |
| hsa-miR-4716-3p | AAGGGGGAAGGAAACA | MIMAT0019827 | hsa-miR-4716-3p | 4 | 5 | 0 | 4 | 5 | 4 | 0 | 0 |
| hsa-miR-4716-5p | CTCCATGTTTCCTTCCCCCTT | MIMAT0019826 | hsa-miR-4716-5p | 0 | 0 | 0 | 0 | 0 | 0 | 0 | 2 |
| hsa-miR-4717-3p | ACACATGGGTGGCTGTGGCCT | MIMAT0019830 | hsa-miR-4717-3p | 9 | 12 | 16 | 11 | 7 | 5 | 0 | 2 |
| hsa-miR-4717-5p | AGGCCACAGCCACCCATGTGT | MIMAT0019829 | hsa-miR-4717-5p | 1 | 0 | 0 | 0 | 7 | 1 | 0 | 0 |
| hsa-miR-4718 | ACCTGAAACCGAGCACCT | MIMAT0019831 | hsa-miR-4718 | 0 | 0 | 0 | 0 | 0 | 0 | 0 | 4 |
| hsa-miR-4721 | TGAGGGCTCCAGGTGACGGTG | MIMAT0019835 | hsa-miR-4721 | 9 | 0 | 0 | 2 | 0 | 0 | 0 | 3 |
| hsa-miR-4722-5p | GGCAGGAGGGCTGTGCCAGGTTG | MIMAT0019836 | hsa-miR-4722-5p | 8 | 17 | 11 | 20 | 22 | 26 | 60 | 27 |
| hsa-miR-4723-3p | CCCTCTCTGGCTCCTCCCCA | MIMAT0019839 | hsa-miR-4723-3p | 1 | 6 | 2 | 6 | 1 | 0 | 0 | 2 |
| hsa-miR-4723-5p | TGGGGGAGCCATGAGATAAGAGC | MIMAT0019838 | hsa-miR-4723-5p | 5 | 11 | 0 | 7 | 12 | 11 | 18 | 11 |
| hsa-miR-4725-3p | TGGGGAAGGCGTCAGTGTCGGG | MIMAT0019844 | hsa-miR-4725-3p | 1714 | 1880 | 1876 | 1252 | 1993 | 1805 | 3396 | 2298 |
| hsa-miR-4725-5p | AGACCCTGCAGCCTTCCCACC | MIMAT0019843 | hsa-miR-4725-5p | 50 | 93 | 49 | 58 | 55 | 72 | 28 | 48 |
| hsa-miR-4726-3p | ACCCAGGTTCCCTCTGGCCGC | MIMAT0019846 | hsa-miR-4726-3p | 0 | 2 | 0 | 0 | 0 | 0 | 0 | 2 |
| hsa-miR-4726-5p | AGGGCCAGAGGAGCCTGGAG | MIMAT0019845 | hsa-miR-4726-5p | 2 | 5 | 8 | 10 | 13 | 8 | 23 | 11 |
| hsa-miR-4727-3p | ATAGTGGGAAGCTGGCAGATT | MIMAT0019848 | hsa-miR-4727-3p | 1 | 0 | 1 | 0 | 1 | 0 | 0 | 0 |
| hsa-miR-4728-3p | CATGCTGACCTCCCTCCTGCCCC | MIMAT0019850 | hsa-miR-4728-3p | 50 | 67 | 56 | 55 | 69 | 85 | 95 | 86 |
| hsa-miR-4728-5p | TGGGAGGGGAGAGGCAGCAAGC | MIMAT0019849 | hsa-miR-4728-5p | 44 | 10 | 0 | 9 | 15 | 7 | 11 | 9 |
| hsa-miR-4730 | CTGGCGGAGCCCATTCCATGCC | MIMAT0019852 | hsa-miR-4730 | 0 | 0 | 0 | 2 | 3 | 0 | 0 | 1 |
| hsa-miR-4731-3p | CACACAAGTGGCCCCCAACACT | MIMAT0019854 | hsa-miR-4731-3p | 0 | 0 | 0 | 0 | 0 | 0 | 0 | 8 |
| hsa-miR-4731-5p | TGCTGGGGGCCACATGAGTGTG | MIMAT0019853 | hsa-miR-4731-5p | 290 | 521 | 260 | 311 | 577 | 514 | 1056 | 638 |
| hsa-miR-4732-3p | GCCCTGACCTGTCCTGTTCTG | MIMAT0019856 | hsa-miR-4732-3p | 2 | 2 | 7 | 2 | 5 | 21 | 0 | 0 |
| hsa-miR-4732-5p | CTGTAGAGCAGGGAGGAGG | MIMAT0019855 | hsa-miR-4732-5p | 0 | 0 | 0 | 0 | 0 | 0 | 0 | 0 |
| hsa-miR-4733-3p | CACCGGGTCTAGCATTGGGAT | MIMAT0019858 | hsa-miR-4733-3p | 0 | 4 | 3 | 3 | 8 | 6 | 13 | 4 |
| hsa-miR-4733-5p | TCCCAATGCTAGACCCGGTGGC | MIMAT0019857 | hsa-miR-4733-5p | 0 | 0 | 0 | 2 | 0 | 2 | 0 | 0 |
| hsa-miR-4734 | GCTGCGGGCTGCGGTCAGGGCGT | MIMAT0019859 | hsa-miR-4734 | 0 | 0 | 0 | 0 | 18 | 16 | 37 | 11 |
| hsa-miR-4738-3p | GAAACTGGAGCGCCTGGAGG | MIMAT0019867 | hsa-miR-4738-3p | 39 | 12 | 26 | 13 | 19 | 28 | 36 | 21 |
| hsa-miR-4739 | CGGGAGGAGGAGCGGAGGG | MIMAT0019868 | hsa-miR-4739 | 0 | 0 | 0 | 0 | 0 | 0 | 0 | 0 |
| hsa-miR-4740-3p | CCGCCCGAGAGGATCCGTCCCA | MIMAT0019870 | hsa-miR-4740-3p | 1 | 0 | 0 | 0 | 0 | 0 | 0 | 0 |
| hsa-miR-4740-5p | AGGACTGATCCTCTCGGGCAGG | MIMAT0019869 | hsa-miR-4740-5p | 0 | 0 | 0 | 0 | 3 | 3 | 10 | 3 |
| hsa-miR-4741 | CGGGCTGTCCGGAGGGGTCGG | MIMAT0019871 | hsa-miR-4741 | 123 | 106 | 91 | 53 | 172 | 151 | 368 | 187 |
| hsa-miR-4742-3p | TCTGTATTCTCCTTTGCCTGC | MIMAT0019873 | hsa-miR-4742-3p | 10 | 18 | 20 | 12 | 12 | 11 | 7 | 11 |
| hsa-miR-4742-5p | TCAGGCAAAGGGATATTTAC | MIMAT0019872 | hsa-miR-4742-5p | 0 | 0 | 0 | 0 | 0 | 0 | 0 | 0 |
| hsa-miR-4743-3p | TGCCTTTCTGTCTTTTCTGGTC | MIMAT0022978 | hsa-miR-4743-3p | 3 | 2 | 8 | 5 | 3 | 6 | 10 | 3 |
| hsa-miR-4743-5p | TGGCCGGATGGGACAGGAGG | MIMAT0019874 | hsa-miR-4743-5p | 42 | 24 | 15 | 8 | 14 | 22 | 20 | 17 |
| hsa-miR-4744 | TAAAGACTAGACTTCGCTATG | MIMAT0019875 | hsa-miR-4744 | 0 | 0 | 0 | 0 | 0 | 0 | 0 | 0 |
| hsa-miR-4745-3p | CCTGGCCCGGCGACGTCTCACGGT | MIMAT0019879 | hsa-miR-4745-3p | 31 | 26 | 45 | 36 | 27 | 31 | 36 | 21 |
| hsa-miR-4745-5p | TGAGTGGGGCTCCCGGGACGGCG | MIMAT0019878 | hsa-miR-4745-5p | 21 | 32 | 3 | 17 | 30 | 28 | 40 | 41 |
| hsa-miR-4746-5p | CCGGTCCCAGGAGAACCTGC | MIMAT0019880 | hsa-miR-4746-5p | 4 | 0 | 0 | 2 | 2 | 0 | 0 | 3 |
| hsa-miR-4747-3p | AAGGCCCGGGCTTTCCTCCC | MIMAT0019883 | hsa-miR-4747-3p | 1 | 1 | 1 | 0 | 9 | 13 | 23 | 6 |
| hsa-miR-4747-5p | AGGGAAGGAGGCTTGGTCTT | MIMAT0019882 | hsa-miR-4747-5p | 4 | 11 | 0 | 9 | 8 | 6 | 11 | 10 |
| hsa-miR-4748 | GAGGTTTGGGGAGGATTTGCT | MIMAT0019884 | hsa-miR-4748 | 31 | 42 | 25 | 33 | 111 | 113 | 241 | 135 |
| hsa-miR-4749-3p | CGCCCCTCCTGCCCCCACAG | MIMAT0019886 | hsa-miR-4749-3p | 3 | 1 | 5 | 1 | 6 | 4 | 0 | 0 |
| hsa-miR-4749-5p | TGCGGGGACAGGCCAGGGC | MIMAT0019885 | hsa-miR-4749-5p | 30 | 37 | 19 | 27 | 26 | 30 | 49 | 14 |
| hsa-miR-4750-3p | CCTGACCCACCCCCTCCCGCT | MIMAT0022979 | hsa-miR-4750-3p | 0 | 0 | 0 | 2 | 0 | 2 | 0 | 0 |
| hsa-miR-4750-5p | CTCGGGCGGAGGTGGTTGAGTG | MIMAT0019887 | hsa-miR-4750-5p | 25 | 71 | 22 | 21 | 55 | 56 | 87 | 63 |
| hsa-miR-4751 | AGAGGACCCGTAGCTGCT | MIMAT0019888 | hsa-miR-4751 | 4 | 3 | 11 | 5 | 0 | 1 | 0 | 1 |
| hsa-miR-4753-3p | TGTTCTCTTTCTTTAGCCTTGT | MIMAT0019891 | hsa-miR-4753-3p | 0 | 0 | 1 | 0 | 2 | 0 | 0 | 0 |
| hsa-miR-4753-5p | AGGCCAAAGGAAGAGAAC | MIMAT0019890 | hsa-miR-4753-5p | 0 | 0 | 2 | 4 | 4 | 0 | 0 | 0 |
| hsa-miR-4754 | ATGCGGACCTGGGTTAGCGGAGT | MIMAT0019894 | hsa-miR-4754 | 0 | 0 | 2 | 2 | 0 | 0 | 0 | 0 |
| hsa-miR-4755-3p | AGCCAGGCTCTGAAGGGAAAGTT | MIMAT0019896 | hsa-miR-4755-3p | 1 | 0 | 1 | 0 | 0 | 0 | 0 | 0 |
| hsa-miR-4755-5p | TTTCCCTTCAGAGCCTGGCTTT | MIMAT0019895 | hsa-miR-4755-5p | 3 | 9 | 6 | 10 | 4 | 5 | 0 | 7 |
| hsa-miR-4757-3p | AGACCATGACGTCACAGAGGCT | MIMAT0019902 | hsa-miR-4757-3p | 0 | 0 | 0 | 2 | 0 | 0 | 0 | 0 |
| hsa-miR-4757-5p | AGGCCTCTGTGACGTCACGGT | MIMAT0019901 | hsa-miR-4757-5p | 11 | 24 | 6 | 14 | 21 | 9 | 33 | 16 |
| hsa-miR-4758-3p | TGCCCCACCTGCTGACCACCCT | MIMAT0019904 | hsa-miR-4758-3p | 4 | 6 | 13 | 8 | 10 | 12 | 3 | 2 |
| hsa-miR-4758-5p | GTGAGTGGGAGCCGGTGGGG | MIMAT0019903 | hsa-miR-4758-5p | 7 | 3 | 0 | 0 | 0 | 0 | 0 | 2 |
| hsa-miR-4761-3p | GAGGGCATGCGCACTTTGTC | MIMAT0019909 | hsa-miR-4761-3p | 0 | 0 | 0 | 0 | 0 | 0 | 0 | 0 |
| hsa-miR-4761-5p | ACAAGGTGTGCATGCCTGACCCGT | MIMAT0019908 | hsa-miR-4761-5p | 0 | 0 | 0 | 0 | 0 | 0 | 0 | 0 |
| hsa-miR-4762-3p | AGGCTTCTGATCAAGATTTG | MIMAT0019911 | hsa-miR-4762-3p | 0 | 0 | 0 | 0 | 0 | 0 | 0 | 0 |
| hsa-miR-4762-5p | CCAAATCTTGATCAGAAGCCTTG | MIMAT0019910 | hsa-miR-4762-5p | 0 | 0 | 0 | 0 | 0 | 0 | 0 | 0 |
| hsa-miR-4763-3p | AGGCAGGGGCTGGTGCTGGG | MIMAT0019913 | hsa-miR-4763-3p | 28 | 10 | 10 | 3 | 7 | 7 | 0 | 15 |
| hsa-miR-4763-5p | CTGCCCAGCCCTCCTGCTCTGGT | MIMAT0019912 | hsa-miR-4763-5p | 16 | 9 | 15 | 17 | 40 | 41 | 82 | 29 |
| hsa-miR-4764-3p | TTAACTCCTTTCACACCCATGGT | MIMAT0019915 | hsa-miR-4764-3p | 0 | 0 | 0 | 1 | 1 | 0 | 0 | 0 |
| hsa-miR-4764-5p | TGGATGTGGAAGGAGTTATCT | MIMAT0019914 | hsa-miR-4764-5p | 0 | 0 | 0 | 0 | 0 | 2 | 0 | 2 |
| hsa-miR-4766-5p | TCTGAAAGAGCAGTTGGTGTTTT | MIMAT0019917 | hsa-miR-4766-5p | 0 | 0 | 0 | 0 | 0 | 0 | 0 | 0 |
| hsa-miR-4767 | CCGCGGGCGCTCCTGGCCGCCGT | MIMAT0019919 | hsa-miR-4767 | 19 | 39 | 28 | 31 | 63 | 57 | 127 | 74 |
| hsa-miR-4768-3p | TCCAGGAGATCCAGAGAGAATC | MIMAT0019921 | hsa-miR-4768-3p | 2 | 0 | 0 | 0 | 0 | 0 | 0 | 0 |
| hsa-miR-4768-5p | TTCTCTCTGGATCCCATGGAT | MIMAT0019920 | hsa-miR-4768-5p | 24 | 11 | 23 | 16 | 14 | 11 | 12 | 8 |
| hsa-miR-4769-3p | TCTGCCATCCTCCCTCCCCTACT | MIMAT0019923 | hsa-miR-4769-3p | 11 | 35 | 15 | 15 | 8 | 8 | 0 | 9 |
| hsa-miR-4769-5p | GTGGGATGGAGAGAAGGTATGAGC | MIMAT0019922 | hsa-miR-4769-5p | 9 | 15 | 4 | 11 | 15 | 8 | 20 | 15 |
| hsa-miR-4772-3p | CCTGCAACTTTGCCTGATCAGT | MIMAT0019927 | hsa-miR-4772-3p | 0 | 0 | 0 | 0 | 0 | 0 | 0 | 0 |
| hsa-miR-4773 | CAGAACAGGAGCATAGAAAGGC | MIMAT0019928 | hsa-miR-4773 | 0 | 0 | 0 | 0 | 2 | 2 | 0 | 0 |
| hsa-miR-4775 | TTAATTTTTTGTTTCGGTCACT | MIMAT0019931 | hsa-miR-4775 | 5 | 19 | 8 | 5 | 6 | 8 | 8 | 6 |
| hsa-miR-4776-3p | CCTTGCCATCCTGGTCCACTGC | MIMAT0019933 | hsa-miR-4776-3p | 0 | 0 | 0 | 0 | 0 | 0 | 0 | 1 |
| hsa-miR-4776-5p | GTGGACCAGGATGGCAAAGG | MIMAT0019932 | hsa-miR-4776-5p | 3 | 2 | 9 | 3 | 7 | 4 | 14 | 6 |
| hsa-miR-4778-5p | AAUUCUGUAAAGGAAGAAGAGG | MIMAT0019936 | hsa-miR-4778-5p | 0 | 0 | 0 | 0 | 0 | 0 | 0 | 0 |
| hsa-miR-4779 | TAGGAGGGAATAGTAAAAGCAGT | MIMAT0019938 | hsa-miR-4779 | 0 | 0 | 0 | 0 | 0 | 0 | 0 | 0 |
| hsa-miR-4781-3p | TTGGAATCCCCGCTAGAGCGTG | MIMAT0019943 | hsa-miR-4781-3p | 1 | 9 | 0 | 5 | 0 | 3 | 0 | 1 |
| hsa-miR-4781-5p | TAGCGGGGATTCCAATATTGGG | MIMAT0019942 | hsa-miR-4781-5p | 1 | 0 | 0 | 8 | 9 | 2 | 0 | 7 |
| hsa-miR-4783-3p | CCCCGGTGTTGGGGCGCGTCTGT | MIMAT0019947 | hsa-miR-4783-3p | 2 | 18 | 6 | 7 | 12 | 18 | 38 | 14 |
| hsa-miR-4784 | TGAGGAGATGCTGGGACTG | MIMAT0019948 | hsa-miR-4784 | 2 | 0 | 1 | 1 | 4 | 0 | 0 | 3 |
| hsa-miR-4785 | GGAGAGTCGGCGACGCCGCCAGT | MIMAT0019949 | hsa-miR-4785 | 10 | 20 | 6 | 10 | 47 | 42 | 120 | 50 |
| hsa-miR-4786-3p | TGAAGCCAGCTCTGGTCTGGG | MIMAT0019955 | hsa-miR-4786-3p | 0 | 0 | 0 | 0 | 1 | 0 | 0 | 0 |
| hsa-miR-4786-5p | TGAGACCAGGACTGGATGCAC | MIMAT0019954 | hsa-miR-4786-5p | 1 | 7 | 6 | 3 | 9 | 5 | 0 | 3 |
| hsa-miR-4787-3p | GATGCGCCGCCCACTGCCCCGCGC | MIMAT0019957 | hsa-miR-4787-3p | 99 | 151 | 39 | 164 | 336 | 297 | 479 | 245 |
| hsa-miR-4787-5p | GGCGGCGGTGGCGGCGGC | MIMAT0019956 | hsa-miR-4787-5p | 7 | 3 | 10 | 7 | 0 | 3 | 0 | 11 |
| hsa-miR-4788 | TACGGACCAGCTAAGGGAGG | MIMAT0019958 | hsa-miR-4788 | 24 | 8 | 15 | 10 | 5 | 2 | 0 | 2 |
| hsa-miR-4789-3p | CACACATAGCAGGTGTATATA | MIMAT0019960 | hsa-miR-4789-3p | 0 | 0 | 0 | 0 | 0 | 0 | 0 | 0 |
| hsa-miR-4791 | TGGATATGATGACTGAAA | MIMAT0019963 | hsa-miR-4791 | 0 | 0 | 1 | 1 | 0 | 0 | 0 | 0 |
| hsa-miR-4792 | CGGTGAGCTCTCGCTGGCC | NA | hsa-miR-4792 | 4 | 2 | 2 | 7 | 5 | 7 | 17 | 12 |
| hsa-miR-4793-3p | TCTGCACTGTGAGTTGGCTGGC | MIMAT0019966 | hsa-miR-4793-3p | 0 | 0 | 2 | 2 | 0 | 0 | 0 | 0 |
| hsa-miR-4795-3p | ATATTATTAGCCACTTCTGGAT | MIMAT0019969 | hsa-miR-4795-3p | 0 | 0 | 0 | 0 | 0 | 0 | 0 | 0 |
| hsa-miR-4796-5p | TGTCTATACTCTGTCACTTTAC | MIMAT0019970 | hsa-miR-4796-5p | 0 | 0 | 0 | 0 | 0 | 0 | 0 | 0 |
| hsa-miR-4797-3p | CAGTAAGTGGCACTCTGTCTTA | MIMAT0019973 | hsa-miR-4797-3p | 0 | 1 | 0 | 0 | 0 | 0 | 0 | 0 |
| hsa-miR-4797-5p | AAGACAGAGTGCCACTTACT | MIMAT0019972 | hsa-miR-4797-5p | 0 | 0 | 0 | 0 | 2 | 0 | 0 | 0 |
| hsa-miR-4799-5p | ATCTAAATGCAGCATGCCAGTC | MIMAT0019976 | hsa-miR-4799-5p | 48 | 80 | 61 | 42 | 45 | 28 | 0 | 70 |
| hsa-miR-4800-3p | CATCCGTCCGTCTGTCCACCTAT | MIMAT0019979 | hsa-miR-4800-3p | 1 | 5 | 5 | 1 | 2 | 0 | 0 | 0 |
| hsa-miR-4800-5p | AGTGGACCGAGGAAGGAAGG | MIMAT0019978 | hsa-miR-4800-5p | 0 | 0 | 0 | 1 | 0 | 0 | 0 | 0 |
| hsa-miR-4804-3p | TTGCTTAACCTTGCCCTCGAA | MIMAT0019985 | hsa-miR-4804-3p | 6 | 7 | 0 | 0 | 2 | 1 | 0 | 0 |
| hsa-miR-4804-5p | TTGGAGGGTAAGGTTAAGCA | MIMAT0019984 | hsa-miR-4804-5p | 612 | 252 | 404 | 226 | 127 | 69 | 60 | 167 |
| hsa-miR-483-3p | TCACTCCTCTCCTCCCGTCTTC | MIMAT0002173 | hsa-miR-483-3p | 0 | 4 | 0 | 0 | 6 | 6 | 17 | 8 |
| hsa-miR-483-5p | AAGACGGGAGGAAAGAAGGG | MIMAT0004761 | hsa-miR-483-5p | 2 | 8 | 5 | 11 | 16 | 9 | 19 | 5 |
| hsa-miR-484 | TCAGGCTCAGTCCCCTCCCGAT | MIMAT0002174 | hsa-miR-484 | 10156 | 22512 | 10280 | 17007 | 16013 | 13720 | 15677 | 14065 |
| hsa-miR-485-3p | GTCATACACGGCTCTCCTCTCT | MIMAT0002176 | hsa-miR-485-3p | 684 | 1372 | 575 | 572 | 775 | 1357 | 773 | 1184 |
| hsa-miR-485-5p | AGAGGCTGGCCGTGATGAATTCG | MIMAT0002175 | hsa-miR-485-5p | 18985 | 37005 | 21062 | 28185 | 51068 | 45249 | 81815 | 46329 |
| hsa-miR-486-3p | CGGGGCAGCTCAGTACAGGAT | MIMAT0004762 | hsa-miR-486-3p | 21 | 32 | 34 | 14 | 14 | 31 | 0 | 0 |
| hsa-miR-486-5p | TCCTGTACTGAGCTGCCCCG | MIMAT0002177 | hsa-miR-486-5p | 312 | 377 | 256 | 322 | 394 | 787 | 570 | 347 |
| hsa-miR-487a-3p | AATCATACAGGGACATCCAGTT | MIMAT0002178 | hsa-miR-487a-3p | 59 | 100 | 52 | 42 | 64 | 52 | 20 | 103 |
| hsa-miR-487a-5p | GTGGTTATCCCTGCTGTGTTCG | MIMAT0026559 | hsa-miR-487a-5p | 3953 | 7372 | 4848 | 5844 | 6708 | 7261 | 10309 | 5614 |
| hsa-miR-487b-3p | AATCGTACAGGGTCATCCACTT | MIMAT0003180 | hsa-miR-487b-3p | 7344 | 11029 | 7179 | 9003 | 9259 | 7177 | 4177 | 8721 |
| hsa-miR-487b-5p | GTGGTTATCCCTGTCCTGTTCG | MIMAT0026614 | hsa-miR-487b-5p | 279 | 408 | 388 | 313 | 293 | 362 | 552 | 316 |
| hsa-miR-488-3p | TTTGAAAGGCTATTTCT | MIMAT0004763 | hsa-miR-488-3p | 1 | 1 | 4 | 1 | 0 | 0 | 0 | 0 |
| hsa-miR-489-3p | GTGACATCACATATACGGCAGC | MIMAT0002805 | hsa-miR-489-3p | 1 | 2 | 5 | 3 | 0 | 0 | 0 | 0 |
| hsa-miR-490-3p | CAACCTGGAGGACTCCATGCTGT | MIMAT0002806 | hsa-miR-490-3p | 10 | 5 | 5 | 5 | 6 | 1 | 0 | 0 |
| hsa-miR-490-5p | CCATGGATCTCCAGGTGGGT | MIMAT0004764 | hsa-miR-490-5p | 989 | 983 | 1167 | 640 | 1028 | 832 | 2230 | 1284 |
| hsa-miR-491-5p | AGTGGGGAACCCTTCCATGAGG | MIMAT0002807 | hsa-miR-491-5p | 236 | 268 | 195 | 246 | 464 | 455 | 907 | 414 |
| hsa-miR-492 | ACCTGCGGGACAAGATTCT | MIMAT0002812 | hsa-miR-492 | 0 | 0 | 0 | 0 | 0 | 0 | 0 | 0 |
| hsa-miR-493-3p | TGAAGGTCTACTGTGTGCCAGG | MIMAT0003161 | hsa-miR-493-3p | 3214 | 3809 | 2722 | 4514 | 5779 | 6941 | 11400 | 5842 |
| hsa-miR-493-5p | TTGTACATGGTAGGCTTTCATT | MIMAT0002813 | hsa-miR-493-5p | 10394 | 21408 | 13729 | 13190 | 11395 | 8379 | 3589 | 20398 |
| hsa-miR-494-3p | TGAAACATACACGGGAAACCTCT | MIMAT0002816 | hsa-miR-494-3p | 32 | 44 | 32 | 56 | 84 | 87 | 6 | 85 |
| hsa-miR-494-5p | AGGTTGTCCGTGTTGTCTTCTC | MIMAT0026607 | hsa-miR-494-5p | 951 | 1445 | 1168 | 899 | 1655 | 1723 | 2999 | 1632 |
| hsa-miR-495-3p | AAACAAACATGGTGCACTTCTT | MIMAT0002817 | hsa-miR-495-3p | 113 | 235 | 143 | 136 | 91 | 87 | 0 | 151 |
| hsa-miR-495-5p | GAAGTTGCCCATGTTATTTTCG | MIMAT0022924 | hsa-miR-495-5p | 713 | 1146 | 631 | 829 | 631 | 467 | 288 | 535 |
| hsa-miR-496 | TGAGTATTACATGGCCAATCTC | MIMAT0002818 | hsa-miR-496 | 783 | 1127 | 737 | 736 | 592 | 472 | 480 | 827 |
| hsa-miR-497-5p | CAGCAGCACACTGTGGTTTGT | MIMAT0002820 | hsa-miR-497-5p | 1386 | 2185 | 1225 | 1698 | 2083 | 1657 | 2891 | 2007 |
| hsa-miR-498 | AAAGCACCUCCAGAGCUUGAAGC | MIMAT0037323 | hsa-miR-498 | 0 | 0 | 0 | 1 | 0 | 0 | 0 | 0 |
| hsa-miR-4999-5p | TGCTGTATTGTCAGGTAGTGAT | MIMAT0021017 | hsa-miR-4999-5p | 2 | 1 | 5 | 1 | 7 | 3 | 0 | 3 |
| hsa-miR-499a-5p | TTAAGACTTGCAGTGATGTTT | MIMAT0002870 | hsa-miR-499a-5p | 10 | 15 | 13 | 14 | 17 | 23 | 23 | 18 |
| hsa-miR-499b-5p | ACAGACTTGCTGTGATGTTC | MIMAT0019897 | hsa-miR-499b-5p | 0 | 0 | 0 | 2 | 0 | 0 | 0 | 0 |
| hsa-miR-5001-3p | TTCTGCCTCTGTCCAGGTCCTTT | MIMAT0021022 | hsa-miR-5001-3p | 28 | 95 | 30 | 44 | 61 | 52 | 106 | 59 |
| hsa-miR-5001-5p | AGGGCTGGACTCAGCGGCGGAGCTGT | MIMAT0021021 | hsa-miR-5001-5p | 13 | 52 | 13 | 27 | 32 | 32 | 51 | 45 |
| hsa-miR-5002-3p | TGACTGCCTCACTGACCACTTC | MIMAT0021024 | hsa-miR-5002-3p | 0 | 0 | 0 | 2 | 0 | 0 | 0 | 0 |
| hsa-miR-5002-5p | AATTTGGTTTCTGAGGCACTT | MIMAT0021023 | hsa-miR-5002-5p | 0 | 0 | 0 | 0 | 0 | 0 | 0 | 0 |
| hsa-miR-5003-3p | TACTTTTCTAGGTTGTTGGGG | MIMAT0021026 | hsa-miR-5003-3p | 8 | 6 | 13 | 3 | 0 | 1 | 0 | 4 |
| hsa-miR-5004-3p | TTGGATTTTCCTGGGCCTC | MIMAT0021028 | hsa-miR-5004-3p | 8 | 9 | 13 | 3 | 10 | 17 | 29 | 19 |
| hsa-miR-5004-5p | TGAGGACAGGGCAAATTCACG | MIMAT0021027 | hsa-miR-5004-5p | 3 | 3 | 3 | 1 | 0 | 0 | 0 | 0 |
| hsa-miR-5006-3p | TTTCCCTTTCCATCCTGGCAGT | MIMAT0021034 | hsa-miR-5006-3p | 7 | 2 | 7 | 4 | 7 | 0 | 0 | 4 |
| hsa-miR-5006-5p | TTGCCAGGGCAGGAGGTGGAAGG | MIMAT0021033 | hsa-miR-5006-5p | 0 | 0 | 0 | 0 | 1 | 0 | 0 | 0 |
| hsa-miR-5008-3p | GTGCTCCCAGGGCCTCGCCTGTT | MIMAT0021040 | hsa-miR-5008-3p | 0 | 4 | 0 | 0 | 0 | 0 | 0 | 0 |
| hsa-miR-5008-5p | CAGGTGAGGCCCTTGGTG | MIMAT0021039 | hsa-miR-5008-5p | 0 | 0 | 0 | 0 | 0 | 0 | 0 | 0 |
| hsa-miR-5009-5p | TGGACTTTTTCAGATTTGGGGAT | MIMAT0021041 | hsa-miR-5009-5p | 0 | 2 | 0 | 0 | 0 | 2 | 0 | 0 |
| hsa-miR-500a-3p | ATGCACCTGGGCAAGGATTCTG | MIMAT0002871 | hsa-miR-500a-3p | 179 | 114 | 91 | 88 | 122 | 137 | 160 | 67 |
| hsa-miR-500a-5p@ | TAATCCTTGCTACCTGGGTGAGAGT | NA | hsa-miR-500a-5p@ | 47 | 160 | 31 | 93 | 122 | 140 | 108 | 123 |
| hsa-miR-500b-3p | AGTGCACCCAGGCAAGGATTC | MIMAT0027032 | hsa-miR-500b-3p | 2 | 5 | 3 | 3 | 6 | 1 | 0 | 0 |
| hsa-miR-5010-3p | TTTTGTGTCTCCCATTCCCCAGT | MIMAT0021044 | hsa-miR-501-3p | 1785 | 1189 | 1212 | 859 | 985 | 991 | 1077 | 823 |
| hsa-miR-5010-5p | AGGGGGATGGCAGAGCAAAATT | MIMAT0021043 | hsa-miR-501-5p | 83 | 212 | 67 | 153 | 334 | 329 | 481 | 294 |
| hsa-miR-501-3p | AATGCACCCGGGCAAGGATTCT | MIMAT0004774 | hsa-miR-5010-3p | 44 | 89 | 32 | 78 | 133 | 132 | 102 | 69 |
| hsa-miR-501-5p | AATCCTTTGTCCCTGGGTGAGAGT | MIMAT0002872 | hsa-miR-5010-5p | 95 | 197 | 129 | 163 | 260 | 239 | 86 | 243 |
| hsa-miR-502-3p | AATGCACCTGGGCAAGGATTC | MIMAT0004775 | hsa-miR-502-3p | 219 | 193 | 196 | 161 | 164 | 194 | 186 | 136 |
| hsa-miR-502-5p | AATCCTTGCTATCTGGGTGCTAGT | MIMAT0002873 | hsa-miR-502-5p | 13 | 12 | 6 | 17 | 25 | 23 | 9 | 37 |
| hsa-miR-503-3p | GGGGTATTGTTTCCGCTGCCAGG | MIMAT0022925 | hsa-miR-503-3p | 649 | 326 | 549 | 318 | 186 | 210 | 224 | 149 |
| hsa-miR-503-5p | TAGCAGCGGGAACAGTTCTG | MIMAT0002874 | hsa-miR-503-5p | 388 | 723 | 394 | 560 | 412 | 362 | 161 | 548 |
| hsa-miR-504-3p | GGGAGTGCAGGGCAGGGTTT | MIMAT0026612 | hsa-miR-504-3p | 9 | 10 | 16 | 6 | 0 | 13 | 0 | 25 |
| hsa-miR-504-5p | GACCCTGGTCTGCACTCTATC | MIMAT0002875 | hsa-miR-504-5p | 43 | 80 | 50 | 45 | 18 | 16 | 0 | 43 |
| hsa-miR-505-3p | CGTCAACACTTGCTGGTTTCCTCT | MIMAT0002876 | hsa-miR-505-3p | 1721 | 4133 | 1682 | 2881 | 1290 | 2393 | 2507 | 2172 |
| hsa-miR-505-5p | GGGAGCCAGGAAGTATTGATGTT | MIMAT0004776 | hsa-miR-505-5p | 2738 | 4651 | 2324 | 3523 | 2939 | 2485 | 794 | 3507 |
| hsa-miR-506-3p | TGTAAGGCACCCTTCTGAGT | MIMAT0002878 | hsa-miR-506-3p | 0 | 0 | 0 | 0 | 0 | 2 | 0 | 0 |
| hsa-miR-508-3p | ATTGTAGCCTTTTGGAGT | MIMAT0002880 | hsa-miR-508-3p | 0 | 0 | 0 | 1 | 0 | 0 | 0 | 0 |
| hsa-miR-508-5p | TACTCCAGAGGGCGTCACTCATG | MIMAT0004778 | hsa-miR-508-5p | 0 | 3 | 1 | 0 | 0 | 0 | 0 | 0 |
| hsa-miR-5087 | ACGGGTTTGTAGCTTTGC | MIMAT0021079 | hsa-miR-5087 | 0 | 0 | 0 | 0 | 0 | 0 | 0 | 0 |
| hsa-miR-5088-3p | CTCCCTTCTTCCTGGGCCCTCA | MIMAT0027041 | hsa-miR-5088-3p | 0 | 2 | 0 | 0 | 0 | 0 | 2 | 0 |
| hsa-miR-5088-5p | CAGGGCTCAGGGATTGGATGGAGG | MIMAT0021080 | hsa-miR-5088-5p | 16 | 14 | 19 | 7 | 9 | 11 | 7 | 5 |
| hsa-miR-509-3-5p | TACTGCAGACGTGGCAATCATG | MIMAT0004975 | hsa-miR-509-3-5p | 0 | 0 | 0 | 3 | 0 | 0 | 0 | 0 |
| hsa-miR-509-3p | TGATTGGTACGTCTGTGGGT | MIMAT0002881 | hsa-miR-509-3p | 11 | 12 | 17 | 8 | 18 | 13 | 30 | 10 |
| hsa-miR-509-5p | TACTGCAGACAGTGGCAATCATG | MIMAT0004779 | hsa-miR-509-5p | 0 | 2 | 0 | 0 | 0 | 0 | 0 | 0 |
| hsa-miR-5090 | CCGGGGCAGATTGGTGTAGGGT | MIMAT0021082 | hsa-miR-5090 | 36 | 34 | 27 | 17 | 43 | 36 | 65 | 48 |
| hsa-miR-5091 | GACGGAGACGACAAGACTGTGC | MIMAT0021083 | hsa-miR-5091 | 0 | 0 | 3 | 0 | 1 | 0 | 0 | 4 |
| hsa-miR-5092 | CACGCTGAGCTTGGCATCTGAT | MIMAT0021084 | hsa-miR-5092 | 0 | 0 | 0 | 0 | 0 | 1 | 0 | 0 |
| hsa-miR-5093 | AGGAAATGAGGCTGGCTAGGAGT | MIMAT0021085 | hsa-miR-5093 | 1 | 0 | 0 | 0 | 2 | 0 | 0 | 0 |
| hsa-miR-5094 | ATCAGTGAATGCCTTGAACCTAAC | MIMAT0021086 | hsa-miR-5094 | 0 | 0 | 0 | 0 | 0 | 0 | 0 | 0 |
| hsa-miR-5095 | GGGATTACAGGCGTGAGC | NA | hsa-miR-5095 | 3 | 5 | 7 | 7 | 8 | 1 | 0 | 0 |
| hsa-miR-5096 | TTCACCATGTTGGCCAGGC | NA | hsa-miR-5096 | 64 | 36 | 62 | 29 | 30 | 33 | 51 | 44 |
| hsa-miR-5100 | ATCCCAGCGGTGCCTCCA | MIMAT0022259 | hsa-miR-5100 | 355 | 313 | 967 | 388 | 168 | 132 | 255 | 175 |
| hsa-miR-511-5p | GTGTCTTTTGCTCTGCAGTC | MIMAT0002808 | hsa-miR-511-5p | 0 | 0 | 3 | 0 | 3 | 1 | 0 | 0 |
| hsa-miR-512-3p | AAGTGCTGTCATAGCTGAGGTC | MIMAT0002823 | hsa-miR-512-3p | 0 | 0 | 0 | 1 | 0 | 2 | 0 | 0 |
| hsa-miR-513c-5p | TTCTCAAGGAGGTGTCGTTTATG | MIMAT0005789 | hsa-miR-513c-5p | 0 | 0 | 0 | 0 | 1 | 0 | 0 | 0 |
| hsa-miR-514a-3p | AUUGACACUUCUGUGAGUAGA | MIMAT0002883 | hsa-miR-514a-3p | 0 | 0 | 0 | 0 | 0 | 0 | 0 | 0 |
| hsa-miR-517-5p | CCTCTAGATGGAAGCACTGTC | MIMAT0002851 | hsa-miR-517-5p | 0 | 0 | 1 | 0 | 0 | 0 | 0 | 0 |
| hsa-miR-517b-3p | ATCGTGCATCCCTTTAGAGTGT | MIMAT0002857 | hsa-miR-517b-3p | 1 | 0 | 0 | 0 | 0 | 0 | 0 | 0 |
| hsa-miR-517c-3p | ATCGTGCATCCTTTTAGAGTGT | MIMAT0002866 | hsa-miR-517c-3p | 0 | 0 | 0 | 0 | 0 | 0 | 0 | 0 |
| hsa-miR-5187-3p | CTCCACTGAATCCTCTTTTCCTC | MIMAT0021118 | hsa-miR-5187-3p | 0 | 0 | 0 | 0 | 0 | 0 | 0 | 0 |
| hsa-miR-5187-5p | TGGGATGAGGGATTGAAG | MIMAT0021117 | hsa-miR-5187-5p | 37 | 60 | 44 | 60 | 91 | 80 | 114 | 101 |
| hsa-miR-5188 | AATCGGACCCATTTAAACCGG | MIMAT0021119 | hsa-miR-5188 | 0 | 1 | 0 | 0 | 0 | 0 | 0 | 0 |
| hsa-miR-5189-3p | TGCCAACCGTCAGAGCCC | MIMAT0027088 | hsa-miR-5189-3p | 0 | 0 | 0 | 0 | 0 | 0 | 0 | 0 |
| hsa-miR-5189-5p | TGGGCACAGGCGGATGGACAGG | MIMAT0021120 | hsa-miR-5189-5p | 0 | 12 | 3 | 3 | 12 | 10 | 36 | 22 |
| hsa-miR-518a-3p | AAAGCGCTTCCCTTTGCTGGAT | MIMAT0002863 | hsa-miR-518a-3p | 0 | 0 | 0 | 0 | 0 | 0 | 0 | 0 |
| hsa-miR-518b | CAAAGCGCUCCCCUUUAGAGGU | MIMAT0002844 | hsa-miR-518b | 0 | 0 | 0 | 0 | 0 | 0 | 0 | 0 |
| hsa-miR-5190 | TGAGCTGGAGCAAGGGCC | MIMAT0021121 | hsa-miR-5190 | 0 | 1 | 0 | 0 | 0 | 0 | 0 | 0 |
| hsa-miR-5191 | AGGATAGGAAGAATGAAGTGC | MIMAT0021122 | hsa-miR-5191 | 2 | 2 | 8 | 3 | 0 | 0 | 0 | 0 |
| hsa-miR-5192 | AGGAGAGTGGATTCCAGG | MIMAT0021123 | hsa-miR-5192 | 1 | 1 | 6 | 1 | 0 | 0 | 0 | 0 |
| hsa-miR-5193 | TCCTCCTCTACCTCATCCCAGT | MIMAT0021124 | hsa-miR-5193 | 2 | 3 | 4 | 5 | 1 | 8 | 0 | 2 |
| hsa-miR-5194 | TGAGGGGTTTGGAATGGGAT | MIMAT0021125 | hsa-miR-5194 | 0 | 3 | 2 | 0 | 0 | 0 | 0 | 4 |
| hsa-miR-5196-3p | TCATCCTCGTCTCCCTCCCAGT | MIMAT0021129 | hsa-miR-5196-3p | 2 | 4 | 8 | 3 | 1 | 0 | 0 | 1 |
| hsa-miR-519b-5p | CTCTAGAGGGAAGCGCTTTCTGT | MIMAT0005454 | hsa-miR-519b-5p | 7 | 5 | 7 | 3 | 17 | 11 | 31 | 14 |
| hsa-miR-519d-3p | CAAAGTGCCTCCCTTTAGAGTGT | MIMAT0002853 | hsa-miR-519d-3p | 0 | 0 | 0 | 0 | 0 | 0 | 0 | 0 |
| hsa-miR-520b | AAAGTGCTTCCTTTTAGAGGG | MIMAT0002843 | hsa-miR-520b@ | 0 | 0 | 0 | 0 | 0 | 0 | 0 | 0 |
| hsa-miR-520g-3p | ACAAAGUGCUUCCCUUUAGAGUGU | MIMAT0002858 | hsa-miR-520g-3p@ | 0 | 0 | 0 | 0 | 0 | 0 | 0 | 0 |
| hsa-miR-522-3p | AAAATGGTTCCCTTTAGAGTGT | MIMAT0002868 | hsa-miR-522-3p | 5 | 7 | 2 | 5 | 3 | 0 | 0 | 0 |
| hsa-miR-525-5p | CTCCAGAGGGATGCACTTTCTC | MIMAT0002838 | hsa-miR-525-5p | 0 | 0 | 0 | 0 | 0 | 0 | 0 | 0 |
| hsa-miR-526b-5p | CTCTTGAGGGAAGCACTTTCT | MIMAT0002835 | hsa-miR-526b-5p | 0 | 2 | 0 | 0 | 0 | 0 | 0 | 0 |
| hsa-miR-532-3p | CCTCCCACACCCAAGGCTTGC | MIMAT0004780 | hsa-miR-532-3p | 3383 | 697 | 926 | 959 | 2120 | 2227 | 802 | 1775 |
| hsa-miR-532-5p | CATGCCTTGAGTGTAGGACCGT | MIMAT0002888 | hsa-miR-532-5p | 22839 | 34851 | 24324 | 27772 | 28676 | 24835 | 39421 | 36082 |
| hsa-miR-539-3p | ATCATACAAGGACAATTTCTTT | MIMAT0022705 | hsa-miR-539-3p | 0 | 7 | 0 | 0 | 0 | 1 | 0 | 0 |
| hsa-miR-539-5p | GGAGAAATTATCCTTGGTGTGTT | MIMAT0003163 | hsa-miR-539-5p | 313 | 451 | 416 | 308 | 354 | 289 | 485 | 459 |
| hsa-miR-541-3p | TGGTGGGCACAGAATCTGGACT | MIMAT0004920 | hsa-miR-541-3p | 19 | 36 | 19 | 27 | 59 | 57 | 91 | 68 |
| hsa-miR-541-5p | AAAGGATTCTGCTGTCGGTC | MIMAT0004919 | hsa-miR-541-5p | 22 | 43 | 25 | 24 | 50 | 45 | 50 | 78 |
| hsa-miR-542-3p | TGTGACAGATTGATAACTGAAA | MIMAT0003389 | hsa-miR-542-3p | 0 | 0 | 0 | 0 | 0 | 0 | 0 | 0 |
| hsa-miR-542-5p | TCGGGGATCATCATGTCACG | MIMAT0003340 | hsa-miR-542-5p | 1187 | 1722 | 1174 | 1621 | 2600 | 2191 | 4606 | 2273 |
| hsa-miR-543 | AAACATTCGCGGTGCACTTCTT | MIMAT0004954 | hsa-miR-543 | 3198 | 6153 | 3242 | 3718 | 3149 | 2550 | 1917 | 3986 |
| hsa-miR-544b | GACCTGAGGTTGTGCATTTCT | MIMAT0015004 | hsa-miR-544b | 6 | 9 | 6 | 8 | 6 | 0 | 0 | 9 |
| hsa-miR-548aa@ | AACCACAATTACTTTTGCACCAA | NA | hsa-miR-548aa@ | 1 | 0 | 3 | 0 | 0 | 0 | 0 | 0 |
| hsa-miR-548ad-5p@ | CAAAAGTAGTTGTGGTTTTTGCC | NA | hsa-miR-548ad-5p@ | 11 | 32 | 21 | 18 | 12 | 3 | 0 | 10 |
| hsa-miR-548ae-3p | ATGGCCAAAACTGCAATTACTT | MIMAT0018954 | hsa-miR-548ae-3p | 0 | 0 | 0 | 0 | 0 | 0 | 0 | 0 |
| hsa-miR-548ag | GTGCAAAGGTAATTGTGGTTT | MIMAT0018969 | hsa-miR-548ag | 0 | 0 | 0 | 0 | 0 | 0 | 0 | 1 |
| hsa-miR-548ah-3p@ | CCAAAACTGCAGTTACTTTTGC | NA | hsa-miR-548ah-3p@ | 3 | 0 | 3 | 3 | 9 | 6 | 0 | 4 |
| hsa-miR-548aj-5p@ | TTGGTGCAAAAGTAATTG | NA | hsa-miR-548aj-5p@ | 0 | 0 | 0 | 0 | 0 | 0 | 0 | 0 |
| hsa-miR-548al | AACGGCAGTGACTTTTGTACC | MIMAT0019024 | hsa-miR-548al | 2 | 3 | 3 | 1 | 1 | 1 | 0 | 4 |
| hsa-miR-548am-3p | CAAAAACTGCAGTTACTTTTGT | MIMAT0019076 | hsa-miR-548am-3p | 0 | 0 | 2 | 0 | 0 | 3 | 0 | 0 |
| hsa-miR-548an | AAAAGGCATTGTGGTTTTTGT | MIMAT0019079 | hsa-miR-548an | 0 | 0 | 0 | 2 | 0 | 0 | 0 | 0 |
| hsa-miR-548ao-3p | AAAGACCGTGACTACTTTTGC | MIMAT0021030 | hsa-miR-548ao-3p | 0 | 0 | 0 | 7 | 0 | 0 | 0 | 0 |
| hsa-miR-548ap-5p@ | AAAAGTAATTGCGGTCTTTGGT | NA | hsa-miR-548ap-5p@ | 17 | 16 | 14 | 11 | 10 | 13 | 34 | 14 |
| hsa-miR-548aq-3p | TGCCATTACTTTTGCACCAA | MIMAT0022264 | hsa-miR-548aq-3p | 0 | 0 | 0 | 0 | 0 | 0 | 1 | 0 |
| hsa-miR-548aq-5p | GAAAGTAATTGCTGTTTTTGCC | MIMAT0022263 | hsa-miR-548aq-5p | 0 | 0 | 0 | 0 | 0 | 0 | 0 | 0 |
| hsa-miR-548ar-3p | UAAAACUGCAGUUAUUUUUGC | MIMAT0022266 | hsa-miR-548ar-3p | 0 | 0 | 0 | 0 | 0 | 0 | 0 | 0 |
| hsa-miR-548as-5p | AAGTAATTGCGGGTTTTGCCTT | MIMAT0022267 | hsa-miR-548as-5p | 0 | 0 | 1 | 1 | 0 | 0 | 0 | 0 |
| hsa-miR-548at-5p | AAAAGTTATTGCGGTTTTGGCT | MIMAT0022277 | hsa-miR-548at-5p | 2 | 2 | 7 | 4 | 5 | 2 | 0 | 4 |
| hsa-miR-548au-5p@ | CAAAAGTAATTGCGGTTTTGGC | NA | hsa-miR-548au-5p@ | 2 | 0 | 0 | 0 | 0 | 0 | 0 | 0 |
| hsa-miR-548av-5p@ | AAAAGTACTTGCGGATTTTGCT | NA | hsa-miR-548av-5p@ | 33 | 75 | 33 | 40 | 45 | 44 | 72 | 60 |
| hsa-miR-548aw | CGGTGCAAAAGTCATCACGGT | MIMAT0022471 | hsa-miR-548aw | 0 | 2 | 0 | 0 | 10 | 10 | 35 | 10 |
| hsa-miR-548ay-3p | CAAAACCGCGATTACTCTTGCC | MIMAT0025453 | hsa-miR-548ay-3p | 0 | 0 | 0 | 0 | 0 | 0 | 0 | 0 |
| hsa-miR-548az-5p | AAGTGATTGTGGTTTTTGCC | MIMAT0025456 | hsa-miR-548az-5p | 0 | 0 | 0 | 0 | 2 | 1 | 0 | 0 |
| hsa-miR-548b-3p | CAAGAACCTCAGTTGCTTTTGT | MIMAT0003254 | hsa-miR-548b-3p | 0 | 0 | 2 | 0 | 0 | 0 | 0 | 0 |
| hsa-miR-548b-5p | CAAAAGTAATTGTGGTTTTGGT | MIMAT0004798 | hsa-miR-548b-5p | 2 | 2 | 9 | 2 | 4 | 3 | 0 | 0 |
| hsa-miR-548d-3p | CAAAAACCACAGTTTCTTTTGC | MIMAT0003323 | hsa-miR-548d-3p | 0 | 0 | 0 | 0 | 0 | 0 | 0 | 0 |
| hsa-miR-548e-3p | AAAAACTGAGACTACTTTTGC | MIMAT0005874 | hsa-miR-548e-3p | 0 | 0 | 0 | 0 | 0 | 0 | 0 | 0 |
| hsa-miR-548e-5p | GTACAAAAGCAATCGCGGTTTTT | MIMAT0026736 | hsa-miR-548e-5p | 0 | 0 | 0 | 0 | 0 | 0 | 0 | 0 |
| hsa-miR-548f-5p | TGGTGCAAAAGTAATCGCAGTT | MIMAT0026739 | hsa-miR-548f-5p | 0 | 12 | 3 | 0 | 0 | 0 | 0 | 0 |
| hsa-miR-548h-5p | AAAAGTAATCGCAGTTTTTGT | MIMAT0005928 | hsa-miR-548h-5p | 2 | 0 | 3 | 0 | 3 | 3 | 6 | 1 |
| hsa-miR-548i | AAAAGUAAUUGCGGAUUUUGCC | MIMAT0005935 | hsa-miR-548i | 0 | 0 | 1 | 0 | 0 | 0 | 0 | 0 |
| hsa-miR-548l | AAAAGTATTTGCGGGTTTTGT | MIMAT0005889 | hsa-miR-548l | 5 | 5 | 6 | 2 | 10 | 10 | 17 | 7 |
| hsa-miR-548m | CAAAGGUAUUUGUGGUUUUUG | MIMAT0005917 | hsa-miR-548m | 0 | 0 | 0 | 0 | 0 | 0 | 0 | 0 |
| hsa-miR-548n | AAAAGTAATTGTGGGTTTTGT | MIMAT0005916 | hsa-miR-548n | 0 | 1 | 0 | 3 | 2 | 0 | 0 | 0 |
| hsa-miR-548q | CTGGTGCAAAAGTAATGGCGGT | MIMAT0011163 | hsa-miR-548q | 15 | 26 | 24 | 39 | 27 | 13 | 10 | 14 |
| hsa-miR-548s | ATGGCCAAAACTGCAGTTATTT | MIMAT0014987 | hsa-miR-548s | 69 | 102 | 67 | 109 | 97 | 71 | 20 | 117 |
| hsa-miR-548t-5p | CAAAAGTGATCGTGGTTTTTG | MIMAT0015009 | hsa-miR-548t-5p | 2 | 0 | 3 | 2 | 0 | 0 | 0 | 1 |
| hsa-miR-548v | AGCTACAGTTACTTTTGCACCAT | MIMAT0015020 | hsa-miR-548v | 0 | 0 | 0 | 0 | 0 | 0 | 0 | 0 |
| hsa-miR-548w | AAAAGTAACTGCGGTTTTTGCC | MIMAT0015060 | hsa-miR-548w | 14 | 4 | 11 | 18 | 23 | 12 | 22 | 23 |
| hsa-miR-548y | AAAAGTAATCACGGTTTTTGCC | MIMAT0018354 | hsa-miR-548y | 0 | 0 | 0 | 0 | 0 | 0 | 0 | 0 |
| hsa-miR-548z | ACCGCCATTACTTTTGCACCA | MIMAT0018446 | hsa-miR-548z | 65 | 113 | 58 | 80 | 77 | 81 | 74 | 95 |
| hsa-miR-549a | GTGACAACTATGGATGAGCTCT | NA | hsa-miR-549a | 8 | 12 | 15 | 6 | 10 | 6 | 1 | 5 |
| hsa-miR-550a-3-5p@ | AGTGCCTGAGGGAGTAAGAGCC | NA | hsa-miR-550a-3-5p@ | 189 | 310 | 226 | 258 | 234 | 211 | 166 | 259 |
| hsa-miR-550a-3p | TCTTACTCCCTCAGGCACATC | MIMAT0003257 | hsa-miR-550a-3p | 0 | 2 | 0 | 0 | 0 | 0 | 0 | 0 |
| hsa-miR-551a | GCGACCCACTCTTGGTTTCC | MIMAT0003214 | hsa-miR-551a | 0 | 0 | 0 | 0 | 0 | 0 | 0 | 0 |
| hsa-miR-551b-3p | GCGACCCATACTTGGTTTCAGT | MIMAT0003233 | hsa-miR-551b-3p | 3 | 0 | 1 | 0 | 1 | 3 | 0 | 0 |
| hsa-miR-551b-5p | GAAATCAAGCGTGGGTGAGACCT | MIMAT0004794 | hsa-miR-551b-5p | 6 | 10 | 6 | 7 | 2 | 6 | 0 | 3 |
| hsa-miR-556-3p | ATATTACCATTAGCTCATC | MIMAT0004793 | hsa-miR-556-3p | 0 | 0 | 0 | 0 | 0 | 0 | 0 | 0 |
| hsa-miR-556-5p | GATGAGCTCATTGTAATATG | MIMAT0003220 | hsa-miR-556-5p | 0 | 0 | 0 | 0 | 0 | 0 | 0 | 0 |
| hsa-miR-5580-3p | CACATATGAAGTGAGCCAGCAC | MIMAT0022274 | hsa-miR-5580-3p | 0 | 0 | 0 | 0 | 2 | 0 | 0 | 0 |
| hsa-miR-5580-5p | TGCTGGCTCATTTCATATGTG | MIMAT0022273 | hsa-miR-5580-5p | 0 | 3 | 0 | 0 | 0 | 0 | 0 | 0 |
| hsa-miR-5581-3p | TTCCATGCCTCCTAGAAGTTCC | MIMAT0022276 | hsa-miR-5581-3p | 5 | 6 | 5 | 8 | 1 | 4 | 0 | 2 |
| hsa-miR-5582-3p | TTAAAACTTTAAGTGTGCCT | MIMAT0022280 | hsa-miR-5582-3p | 2 | 3 | 2 | 0 | 0 | 0 | 0 | 2 |
| hsa-miR-5583-3p | GAATATGGGTATATTAGTT | MIMAT0022282 | hsa-miR-5583-3p | 0 | 0 | 0 | 2 | 0 | 0 | 0 | 0 |
| hsa-miR-5585-3p | CTGAATAGCTGGGACTACAGGT | MIMAT0022286 | hsa-miR-5585-3p | 4 | 4 | 16 | 11 | 10 | 14 | 29 | 8 |
| hsa-miR-5585-5p | TGAAGTACCAGCTACTCGAG | MIMAT0022285 | hsa-miR-5585-5p | 0 | 0 | 0 | 0 | 0 | 0 | 0 | 0 |
| hsa-miR-5587-3p | GCCCCGGGCAGTGTGATCATC | MIMAT0022290 | hsa-miR-5587-3p | 4 | 4 | 7 | 3 | 0 | 4 | 0 | 0 |
| hsa-miR-5587-5p | ATGGTCACCTCCGGGACTCAGC | MIMAT0022289 | hsa-miR-5587-5p | 0 | 0 | 0 | 3 | 0 | 0 | 0 | 0 |
| hsa-miR-5588-3p | TAAAAGTCCCACTAATGCCAGC | MIMAT0022296 | hsa-miR-5588-3p | 3 | 0 | 1 | 4 | 4 | 3 | 0 | 9 |
| hsa-miR-5588-5p | ACTGGCATTAGTGGGACTTTTT | MIMAT0022295 | hsa-miR-5588-5p | 1 | 0 | 0 | 0 | 2 | 1 | 0 | 3 |
| hsa-miR-561-3p | ATCAAGGATCTTAAACTTTGCCAG | MIMAT0003225 | hsa-miR-561-3p | 0 | 0 | 0 | 0 | 0 | 0 | 0 | 0 |
| hsa-miR-561-5p | AUCAAGGAUCUUAAACUUUGCC | MIMAT0022706 | hsa-miR-561-5p | 0 | 0 | 0 | 0 | 0 | 0 | 0 | 1 |
| hsa-miR-564 | CAGGCACGGTGTCAGCAGGC | MIMAT0003228 | hsa-miR-564 | 0 | 0 | 0 | 0 | 0 | 0 | 0 | 0 |
| hsa-miR-566 | GGCGGGCGCCTGTAATCC | NA | hsa-miR-566 | 0 | 0 | 0 | 0 | 2 | 0 | 0 | 0 |
| hsa-miR-5684 | ACTCCAGCCTGAGCAACA | MIMAT0022473 | hsa-miR-5684 | 0 | 0 | 0 | 0 | 0 | 0 | 0 | 0 |
| hsa-miR-5685 | TCACAGCCCTGCAGTTATCACG | MIMAT0022475 | hsa-miR-5685 | 1 | 1 | 4 | 1 | 0 | 0 | 0 | 0 |
| hsa-miR-5687 | TTAGAACGTTTTAGGGTCAA | MIMAT0022478 | hsa-miR-5687 | 0 | 0 | 0 | 0 | 0 | 0 | 0 | 0 |
| hsa-miR-5689 | GCATGCACCTGTAGTCCT | MIMAT0022481 | hsa-miR-5689 | 0 | 0 | 0 | 0 | 4 | 4 | 15 | 4 |
| hsa-miR-5690 | TCAGCTACTACCTCTATTAGGA | MIMAT0022482 | hsa-miR-5690 | 2 | 0 | 0 | 0 | 4 | 2 | 0 | 0 |
| hsa-miR-5693 | GGAGTGGCTCTGAAATGA | MIMAT0022486 | hsa-miR-5693 | 0 | 0 | 0 | 0 | 0 | 0 | 0 | 0 |
| hsa-miR-5695 | GACTCCAAGAAGAATCTAGACA | MIMAT0022488 | hsa-miR-5695 | 0 | 0 | 0 | 0 | 0 | 0 | 0 | 0 |
| hsa-miR-5697 | AAGTAGTTTCATGATAAAGGGT | MIMAT0022490 | hsa-miR-5697 | 0 | 0 | 0 | 0 | 0 | 0 | 0 | 2 |
| hsa-miR-5698 | TGGGGGAGTGCAGTGATTG | MIMAT0022491 | hsa-miR-5698 | 11 | 26 | 23 | 12 | 20 | 9 | 0 | 2 |
| hsa-miR-5699-3p | TGTCTTTCCTTGTTGGAGCAGG | MIMAT0022492 | hsa-miR-5699-3p | 14 | 10 | 16 | 21 | 15 | 15 | 36 | 19 |
| hsa-miR-5699-5p | TGCCCCAACAAGGAAGGACA | MIMAT0027103 | hsa-miR-5699-5p | 4 | 0 | 0 | 0 | 0 | 0 | 0 | 0 |
| hsa-miR-5701 | GATTGTCACGTTCTGATT | MIMAT0022494 | hsa-miR-5701 | 1 | 3 | 0 | 10 | 13 | 10 | 23 | 11 |
| hsa-miR-5703 | AGGAGAAGTCGGGAAGGTGGC | MIMAT0022496 | hsa-miR-5703 | 0 | 0 | 0 | 0 | 0 | 0 | 0 | 0 |
| hsa-miR-5708 | ATGAGCCACTGTGCCTGAC | MIMAT0022502 | hsa-miR-5708 | 0 | 0 | 0 | 0 | 0 | 0 | 0 | 0 |
| hsa-miR-572 | GTCCGCTCGGCGGTGGCCCAGC | MIMAT0003237 | hsa-miR-572 | 0 | 0 | 0 | 0 | 0 | 0 | 0 | 0 |
| hsa-miR-574-3p | CACGCTCATGCACACACCCAC | MIMAT0003239 | hsa-miR-574-3p | 61149 | 112497 | 53776 | 70572 | 89731 | 59917 | 62046 | 81051 |
| hsa-miR-574-5p | TGAGTGTGTGTGTGTGAGTGTGT | MIMAT0004795 | hsa-miR-574-5p | 69133 | 154263 | 73476 | 147111 | 212382 | 214527 | 166305 | 182163 |
| hsa-miR-576-3p | AAGATGTGGAAAAATTGGAATC | MIMAT0004796 | hsa-miR-576-3p | 0 | 0 | 0 | 0 | 3 | 0 | 0 | 0 |
| hsa-miR-576-5p | ATTCTAATTTCTCCACGTCTTTG | MIMAT0003241 | hsa-miR-576-5p | 48 | 95 | 44 | 38 | 49 | 60 | 64 | 69 |
| hsa-miR-577 | UAGAUAAAAUAUUGGUACCUG | MIMAT0003242 | hsa-miR-577 | 0 | 0 | 0 | 0 | 0 | 0 | 0 | 0 |
| hsa-miR-5787 | GGGGGCTGGGGCGCGGGGG | MIMAT0023252 | hsa-miR-5787 | 0 | 0 | 0 | 0 | 0 | 0 | 0 | 0 |
| hsa-miR-579-3p | TTCATTTGGTATAAACCGCGATT | MIMAT0003244 | hsa-miR-579-3p | 0 | 0 | 0 | 1 | 0 | 0 | 0 | 0 |
| hsa-miR-579-5p | TCGCGGTTTGTGCCAGATGACG | MIMAT0026616 | hsa-miR-579-5p | 60 | 92 | 72 | 97 | 105 | 73 | 112 | 103 |
| hsa-miR-582-3p | TAACTGGTTGAACAACTGAACC | MIMAT0004797 | hsa-miR-582-3p | 0 | 0 | 0 | 0 | 0 | 0 | 0 | 0 |
| hsa-miR-582-5p | TTACAGTTGTTCAACCAGTTACT | MIMAT0003247 | hsa-miR-582-5p | 103 | 231 | 112 | 196 | 141 | 91 | 34 | 148 |
| hsa-miR-584-3p | TCAGTTCCAGGCCAACCAGGC | MIMAT0022708 | hsa-miR-584-3p | 186 | 222 | 246 | 150 | 108 | 81 | 50 | 86 |
| hsa-miR-584-5p | TTATGGTTTGCCTGGGACTG | MIMAT0003249 | hsa-miR-584-5p | 13050 | 14763 | 12826 | 10970 | 13951 | 12379 | 21222 | 16001 |
| hsa-miR-585-3p | TGGACGTATCTGTATGCTAGGG | MIMAT0003250 | hsa-miR-585-3p | 1471 | 542 | 1294 | 655 | 324 | 371 | 397 | 265 |
| hsa-miR-585-5p | TAGCACACAGATACGCCCAG | MIMAT0026618 | hsa-miR-585-5p | 5 | 9 | 10 | 10 | 14 | 6 | 21 | 6 |
| hsa-miR-588 | TGGCCACAATGGGTTAGAAC | MIMAT0003255 | hsa-miR-588 | 0 | 0 | 0 | 0 | 0 | 0 | 0 | 0 |
| hsa-miR-589-3p | TCAGAACAAATGCCGGTTCCC | MIMAT0003256 | hsa-miR-589-3p | 65 | 102 | 93 | 84 | 94 | 80 | 93 | 65 |
| hsa-miR-589-5p | TGAGAACCACGTCTGCTCTG | MIMAT0004799 | hsa-miR-589-5p | 453 | 640 | 680 | 551 | 501 | 471 | 757 | 484 |
| hsa-miR-590-3p | TAATTTTATGTATAAGCTAGT | MIMAT0004801 | hsa-miR-590-3p | 16 | 31 | 18 | 12 | 4 | 7 | 6 | 8 |
| hsa-miR-590-5p | GAGCTTATTCATAAAAGTGCAG | MIMAT0003258 | hsa-miR-590-5p | 0 | 0 | 0 | 0 | 1 | 0 | 0 | 0 |
| hsa-miR-592 | TTGTGTCAATATGCGATGATGT | MIMAT0003260 | hsa-miR-592 | 0 | 0 | 1 | 0 | 0 | 0 | 0 | 0 |
| hsa-miR-597-3p | TGGTTCTCTTGTGGCTCAAGCG | MIMAT0026619 | hsa-miR-597-3p | 52 | 26 | 43 | 26 | 33 | 21 | 63 | 28 |
| hsa-miR-598-3p | TACGTCATCGTTGTCATCGTC | MIMAT0003266 | hsa-miR-598-3p | 26 | 28 | 26 | 37 | 32 | 33 | 40 | 26 |
| hsa-miR-598-5p | GCGGTGATCCCGATGGTGTGAG | MIMAT0026620 | hsa-miR-598-5p | 0 | 0 | 0 | 0 | 0 | 0 | 0 | 0 |
| hsa-miR-600 | ACTTACAGACAAGAGCCTTGC | MIMAT0003268 | hsa-miR-600 | 0 | 0 | 0 | 0 | 0 | 0 | 0 | 0 |
| hsa-miR-602 | GACACGGGCGACAGCTGCGGCT | MIMAT0003270 | hsa-miR-602 | 4 | 4 | 4 | 1 | 11 | 11 | 37 | 11 |
| hsa-miR-605-3p | AGAGGGCACTATGAGATTT | MIMAT0026621 | hsa-miR-605-3p | 1 | 2 | 1 | 0 | 0 | 0 | 0 | 7 |
| hsa-miR-605-5p | TAAATCCCATGGTGCCTTCTCC | MIMAT0003273 | hsa-miR-605-5p | 345 | 800 | 352 | 342 | 322 | 231 | 265 | 438 |
| hsa-miR-6068 | ATCCGGCGGTGGCTTG | MIMAT0023693 | hsa-miR-6087 | 81 | 174 | 110 | 201 | 532 | 481 | 1109 | 520 |
| hsa-miR-6087 | GTGAGGCGGGGGGGCGAGCC | NA | hsa-miR-6088 | 0 | 0 | 0 | 0 | 5 | 5 | 18 | 5 |
| hsa-miR-6088 | TGAAGCGGGGGGGTGGGG | MIMAT0023713 | hsa-miR-6089 | 0 | 0 | 0 | 2 | 2 | 0 | 0 | 0 |
| hsa-miR-6089 | CGGGGCGGGGCGGGGCGGGG | MIMAT0023714 | hsa-miR-6090 | 0 | 0 | 0 | 0 | 0 | 0 | 0 | 0 |
| hsa-miR-610 | TGTGAGCTAAATGTGTGCTGGG | MIMAT0003278 | hsa-miR-610 | 0 | 0 | 0 | 0 | 3 | 1 | 3 | 1 |
| hsa-miR-612 | GCTGGGCAGGGCTTCTGAGCT | MIMAT0003280 | hsa-miR-612 | 0 | 0 | 0 | 0 | 0 | 0 | 0 | 0 |
| hsa-miR-6125 | AGGCGGAGCGGCGGATCTG | MIMAT0024598 | hsa-miR-6125 | 1 | 11 | 4 | 1 | 3 | 0 | 0 | 0 |
| hsa-miR-6126 | GUGAAGGCCCGGCGGAGA | MIMAT0024599 | hsa-miR-6126 | 0 | 0 | 0 | 0 | 2 | 0 | 0 | 0 |
| hsa-miR-6129 | TGAGGGAGTAGGGTGTATAG | MIMAT0024613 | hsa-miR-6129 | 0 | 0 | 3 | 0 | 0 | 0 | 0 | 0 |
| hsa-miR-6130 | TGAGGGAGTAGATTGTAT | MIMAT0024614 | hsa-miR-6130 | 0 | 0 | 0 | 0 | 0 | 0 | 0 | 0 |
| hsa-miR-6131 | GGCTGGTCCGATGGGAG | MIMAT0024615 | hsa-miR-6131 | 4 | 2 | 0 | 0 | 0 | 1 | 0 | 0 |
| hsa-miR-6132 | AGCAGGGCTGGGGATTGCAGT | MIMAT0024616 | hsa-miR-6132 | 0 | 3 | 0 | 7 | 2 | 3 | 0 | 5 |
| hsa-miR-615-3p | TCCGAGCCTGGGTCTCCCTCTT | MIMAT0003283 | hsa-miR-615-3p | 694 | 1276 | 597 | 1077 | 2157 | 1969 | 4538 | 2084 |
| hsa-miR-615-5p | GGGGGTCCCCGGTGCTCGGATC | MIMAT0004804 | hsa-miR-615-5p | 17 | 11 | 13 | 12 | 39 | 31 | 89 | 42 |
| hsa-miR-616-3p | AAGTCATTGGAGGGTTTGAGC | MIMAT0004805 | hsa-miR-616-3p | 39 | 54 | 36 | 85 | 85 | 87 | 108 | 74 |
| hsa-miR-616-5p | ACTCAAAACCCTTCAGTGACTTCC | MIMAT0003284 | hsa-miR-616-5p | 1 | 5 | 0 | 1 | 4 | 0 | 0 | 5 |
| hsa-miR-618 | AAACTCTACTTGTCCTTCTG | MIMAT0003287 | hsa-miR-618 | 8 | 1 | 6 | 6 | 12 | 3 | 3 | 6 |
| hsa-miR-619-5p | GCTGGGATTACAGGCGTGAGCC | MIMAT0026622 | hsa-miR-619-5p | 45 | 39 | 52 | 42 | 25 | 45 | 5 | 37 |
| hsa-miR-624-5p | TAGTACCAGTACCTTGTGTTC | MIMAT0003293 | hsa-miR-624-5p | 63 | 86 | 61 | 98 | 81 | 52 | 45 | 43 |
| hsa-miR-625-3p | GACTATAGAACTTTCCCCCTC | MIMAT0004808 | hsa-miR-625-3p | 77 | 126 | 59 | 129 | 119 | 169 | 61 | 110 |
| hsa-miR-625-5p | AGGGGGAAAGTTCTATAGTCC | MIMAT0003294 | hsa-miR-625-5p | 4609 | 8818 | 5493 | 6473 | 9625 | 9042 | 17023 | 11640 |
| hsa-miR-627-3p | TCTTTTCTTTGAGACTCACTA | MIMAT0026623 | hsa-miR-627-3p | 0 | 0 | 0 | 0 | 0 | 0 | 0 | 0 |
| hsa-miR-627-5p | TGAGTCTCTAAGAAAAGAGGAGG | MIMAT0003296 | hsa-miR-627-5p | 0 | 0 | 0 | 0 | 0 | 2 | 0 | 0 |
| hsa-miR-628-3p | TCTAGTAAGAGTGGCAGTCGA | MIMAT0003297 | hsa-miR-628-3p | 160 | 107 | 125 | 89 | 134 | 95 | 32 | 90 |
| hsa-miR-628-5p | ATGCTGACATATTTACTAGAGG | MIMAT0004809 | hsa-miR-628-5p | 0 | 0 | 1 | 3 | 7 | 4 | 0 | 0 |
| hsa-miR-629-3p | TTCTCCCAACGTAAGCCCAGC | MIMAT0003298 | hsa-miR-629-3p | 212 | 295 | 163 | 170 | 146 | 119 | 32 | 201 |
| hsa-miR-629-5p | TGGGTTTACGTTGGGAGAACTT | MIMAT0004810 | hsa-miR-629-5p | 89 | 134 | 50 | 129 | 287 | 324 | 311 | 237 |
| hsa-miR-631 | GACCTGGCCCAGACCTCAG | MIMAT0003300 | hsa-miR-631 | 0 | 0 | 0 | 0 | 0 | 0 | 0 | 0 |
| hsa-miR-632 | TTGTGTCTGCTTCCTGTGGGA | MIMAT0003302 | hsa-miR-632 | 0 | 0 | 0 | 0 | 0 | 0 | 0 | 0 |
| hsa-miR-636 | TGTGCTTGCTCGTCCCGCCCGC | MIMAT0003306 | hsa-miR-636 | 137 | 246 | 127 | 127 | 148 | 135 | 312 | 200 |
| hsa-miR-639 | GCGGTTGCGAGCGCTGTAGGG | MIMAT0003309 | hsa-miR-639 | 0 | 0 | 0 | 0 | 0 | 0 | 0 | 0 |
| hsa-miR-641 | AAAGACATAGGATAGAGTCACCT | MIMAT0003311 | hsa-miR-641 | 58 | 70 | 84 | 73 | 121 | 66 | 55 | 87 |
| hsa-miR-642a-3p | GACACATTTGGAGAGGGAACCTT | MIMAT0020924 | hsa-miR-642a-3p | 8 | 9 | 21 | 22 | 4 | 1 | 0 | 19 |
| hsa-miR-642a-5p | GTCCCTCTCCAAATGTGTCTTG | MIMAT0003312 | hsa-miR-642a-5p | 78 | 195 | 55 | 115 | 138 | 174 | 123 | 151 |
| hsa-miR-643 | ACTTGTATGCTAGCTCAGGT | MIMAT0003313 | hsa-miR-643 | 4 | 0 | 0 | 3 | 0 | 0 | 0 | 1 |
| hsa-miR-6500-3p | ACACTTGTTGGGATGACCTGC | MIMAT0025455 | hsa-miR-6500-3p | 7 | 21 | 14 | 27 | 18 | 19 | 45 | 27 |
| hsa-miR-6501-3p | CCAGAGCAGCCTGCGGTAACAGT | MIMAT0025459 | hsa-miR-6501-3p | 15 | 17 | 20 | 14 | 4 | 6 | 0 | 15 |
| hsa-miR-6501-5p | AGTTGCCAGGGCTGCCTTTGGTGT | MIMAT0025458 | hsa-miR-6501-5p | 2 | 0 | 0 | 0 | 1 | 0 | 0 | 0 |
| hsa-miR-6502-5p | AGCTCTAGAAAGATTGTTGACC | MIMAT0025460 | hsa-miR-6502-5p | 0 | 0 | 0 | 0 | 2 | 0 | 0 | 0 |
| hsa-miR-6503-5p | AGGTCTGCATTCAAATCCCCAGT | MIMAT0025462 | hsa-miR-6503-5p | 0 | 0 | 0 | 0 | 0 | 0 | 0 | 0 |
| hsa-miR-6504-5p | GTCTGGCTGTGCTGTAATGCA | MIMAT0025464 | hsa-miR-6504-5p | 0 | 0 | 0 | 0 | 0 | 0 | 0 | 0 |
| hsa-miR-6505-3p | TGACTTCTACCTCTTCCAAAG | MIMAT0025467 | hsa-miR-6505-3p | 0 | 0 | 0 | 0 | 0 | 0 | 0 | 0 |
| hsa-miR-6505-5p | TTGGAATAGGGGATATCTCAGC | MIMAT0025466 | hsa-miR-6505-5p | 0 | 0 | 1 | 0 | 6 | 4 | 14 | 8 |
| hsa-miR-6508-3p | TGGGCCATGCATTTCTAGAACTC | MIMAT0025473 | hsa-miR-6508-3p | 1 | 10 | 4 | 6 | 3 | 13 | 0 | 3 |
| hsa-miR-6508-5p | UCUAGAAAUGCAUGACCCACC | MIMAT0025472 | hsa-miR-6508-5p | 0 | 0 | 0 | 0 | 0 | 0 | 0 | 0 |
| hsa-miR-6509-3p | TTCCACTGCCACTACCTAATTT | MIMAT0025475 | hsa-miR-6509-3p | 2 | 6 | 1 | 3 | 10 | 4 | 0 | 14 |
| hsa-miR-6509-5p | ATTAGGTAGTGGCAGTGGAAC | MIMAT0025474 | hsa-miR-6509-5p | 8 | 8 | 1 | 3 | 3 | 1 | 0 | 1 |
| hsa-miR-651-5p | UUUAGGAUAAGCUUGACUUUUG | MIMAT0003321 | hsa-miR-651-5p | 0 | 0 | 0 | 0 | 0 | 0 | 1 | 0 |
| hsa-miR-6510-3p | CACCGACTCTGTCTCCTGCAGT | MIMAT0025477 | hsa-miR-6510-3p | 0 | 2 | 0 | 0 | 1 | 1 | 0 | 1 |
| hsa-miR-6510-5p | CAGCAGGGGAGAGAGAGGAGTCT | MIMAT0025476 | hsa-miR-6510-5p | 0 | 0 | 1 | 0 | 0 | 0 | 0 | 0 |
| hsa-miR-6511a-3p | CCTCACCATCCCTTCTGCCTGC | MIMAT0025479 | hsa-miR-6511a-3p | 239 | 388 | 500 | 387 | 260 | 322 | 216 | 222 |
| hsa-miR-6511a-5p@ | CTGCAGGCAGAAGTGGGGCTG | NA | hsa-miR-6511a-5p@ | 52 | 123 | 63 | 131 | 121 | 160 | 174 | 125 |
| hsa-miR-6511b-3p | TCACCACCCCTTCTGCCTGCAGT | MIMAT0025848 | hsa-miR-6511b-3p | 246 | 398 | 212 | 389 | 511 | 580 | 492 | 360 |
| hsa-miR-6513-3p | TCAAGTGTCATCTGTCCCTAGT | MIMAT0025483 | hsa-miR-6513-3p | 4 | 0 | 0 | 1 | 6 | 6 | 14 | 5 |
| hsa-miR-6513-5p | TTTGGGATTGACGCCACATGTC | MIMAT0025482 | hsa-miR-6513-5p | 37 | 31 | 41 | 49 | 42 | 38 | 59 | 40 |
| hsa-miR-6514-3p | CTGCCTGTTCTTCCACTCC | MIMAT0025485 | hsa-miR-6514-3p | 19 | 25 | 24 | 21 | 23 | 23 | 54 | 23 |
| hsa-miR-6514-5p | ATGGAGTGGACTTTCAGCTGGC | MIMAT0025484 | hsa-miR-6514-5p | 72 | 132 | 94 | 94 | 145 | 110 | 148 | 131 |
| hsa-miR-6515-3p | TCTCTTCATCTACCCCCCAG | MIMAT0025487 | hsa-miR-6515-3p | 0 | 0 | 2 | 0 | 0 | 0 | 0 | 0 |
| hsa-miR-6515-5p | TTGGAGGGTGTGGAAGACAT | MIMAT0025486 | hsa-miR-6515-5p | 2 | 7 | 0 | 2 | 25 | 12 | 27 | 13 |
| hsa-miR-6516-5p | TTTGCAGTAACAGGTGTGAGC | MIMAT0030417 | hsa-miR-6516-5p | 8 | 14 | 8 | 5 | 4 | 8 | 8 | 8 |
| hsa-miR-652-3p | AATGGCGCCACTAGGGTTGTGT | MIMAT0003322 | hsa-miR-652-3p | 40024 | 60321 | 39299 | 38935 | 57914 | 53013 | 97872 | 56143 |
| hsa-miR-652-5p | ACAACCCTAGGAGAGGGTGCCATTC | MIMAT0022709 | hsa-miR-652-5p | 7 | 27 | 9 | 12 | 19 | 16 | 0 | 26 |
| hsa-miR-654-3p | TATGTCTGCTGACCATCACC | MIMAT0004814 | hsa-miR-654-3p | 5322 | 9948 | 4846 | 5908 | 6491 | 4886 | 7721 | 7300 |
| hsa-miR-654-5p | TGGTGGGCCGCAGAACATGTGC | MIMAT0003330 | hsa-miR-654-5p | 11454 | 17261 | 11192 | 10674 | 12626 | 10495 | 12353 | 14717 |
| hsa-miR-655-3p | ATAATACATGGTTAACCTCTTT | MIMAT0003331 | hsa-miR-655-3p | 1 | 2 | 0 | 0 | 3 | 2 | 0 | 0 |
| hsa-miR-655-5p | AGAGGTTATCCGTGTTATGTTC | MIMAT0026626 | hsa-miR-655-5p | 30 | 53 | 56 | 41 | 35 | 23 | 0 | 16 |
| hsa-miR-656-3p | AATATTATACAGTCAACCTCT | MIMAT0003332 | hsa-miR-656-3p | 9 | 11 | 6 | 27 | 17 | 9 | 0 | 25 |
| hsa-miR-656-5p | AGGTTGCCTGTGAGGTGTTC | MIMAT0026627 | hsa-miR-656-5p | 23 | 25 | 21 | 24 | 65 | 68 | 142 | 57 |
| hsa-miR-658 | AGGGAAGTAGGTCCGT | MIMAT0003336 | hsa-miR-658 | 0 | 0 | 0 | 0 | 0 | 0 | 0 | 0 |
| hsa-miR-659-3p | TTGGTTCAGGGAGGGTCCCC | MIMAT0003337 | hsa-miR-659-3p | 1 | 4 | 0 | 6 | 12 | 4 | 0 | 4 |
| hsa-miR-659-5p | AGGACCTTCCCTGAACCAAGGAA | MIMAT0022710 | hsa-miR-659-5p | 0 | 0 | 3 | 0 | 0 | 1 | 0 | 0 |
| hsa-miR-660-3p | ACCTCCTGTGTGCATGGATTAC | MIMAT0022711 | hsa-miR-660-3p | 9 | 22 | 16 | 23 | 15 | 12 | 8 | 11 |
| hsa-miR-660-5p | TACCCATTGCATATCGGAGTTGT | MIMAT0003338 | hsa-miR-660-5p | 165 | 294 | 137 | 234 | 179 | 177 | 95 | 184 |
| hsa-miR-663a | CCAGGCGGGGCGCCGCGGGACCGC | MIMAT0003326 | hsa-miR-663a | 2 | 2 | 9 | 9 | 8 | 4 | 15 | 4 |
| hsa-miR-663b | GGTGGCCCGGCCGTGCCTGAGG | MIMAT0005867 | hsa-miR-663b | 6012 | 2332 | 4323 | 1567 | 711 | 619 | 1497 | 947 |
| hsa-miR-664a-3p | TATTCATTTATCCCCAGCCTAC | MIMAT0005949 | hsa-miR-664a-3p | 30 | 49 | 40 | 56 | 97 | 48 | 20 | 50 |
| hsa-miR-664a-5p | ACTGGCTAGGGAAAATGAT | MIMAT0005948 | hsa-miR-664a-5p | 454 | 571 | 302 | 623 | 922 | 717 | 446 | 673 |
| hsa-miR-664b-3p | TTCATTTGCCTCCCAGCCTAC | MIMAT0022272 | hsa-miR-664b-3p | 92 | 147 | 74 | 133 | 136 | 72 | 12 | 61 |
| hsa-miR-664b-5p | TGGGCTAAGGGAGATGATTGGGT | MIMAT0022271 | hsa-miR-664b-5p | 78 | 201 | 30 | 102 | 301 | 212 | 182 | 232 |
| hsa-miR-665 | ACCAGGAGGCTGAGGCCCCT | MIMAT0004952 | hsa-miR-665 | 9 | 11 | 28 | 70 | 22 | 14 | 0 | 3 |
| hsa-miR-668-3p | TGTCACTCGGCTCGGCCCACTACC | MIMAT0003881 | hsa-miR-668-3p | 702 | 1296 | 859 | 1103 | 1139 | 915 | 1287 | 1214 |
| hsa-miR-668-5p | TAAGTGCGCCTCGGGTGAGCATG | MIMAT0026636 | hsa-miR-668-5p | 0 | 2 | 0 | 0 | 0 | 4 | 0 | 0 |
| hsa-miR-671-3p | TCCGGTTCTCAGGGCTCCACC | MIMAT0004819 | hsa-miR-671-3p | 121 | 187 | 106 | 156 | 168 | 164 | 220 | 153 |
| hsa-miR-6715a-3p | CCAAACCGGTCGTGCCTG | MIMAT0025841 | hsa-miR-671-5p | 1090 | 1778 | 624 | 1353 | 2973 | 3610 | 5031 | 3062 |
| hsa-miR-6715b-3p | CTCAAACCGGCTGTGCCTG | MIMAT0025843 | hsa-miR-6715a-3p | 0 | 0 | 0 | 0 | 2 | 0 | 0 | 0 |
| hsa-miR-671-5p | AGGAAGCCCTGGAGGGGCTGGAGG | MIMAT0003880 | hsa-miR-6715b-3p | 10 | 0 | 0 | 2 | 0 | 0 | 0 | 0 |
| hsa-miR-6716-3p | TCCGAACTCTCCATTCCTCTGC | MIMAT0025845 | hsa-miR-6716-3p | 4 | 11 | 2 | 4 | 12 | 8 | 16 | 4 |
| hsa-miR-6716-5p | TGGGAATGGGGGTAAGGGCCT | MIMAT0025844 | hsa-miR-6716-5p | 24 | 31 | 6 | 6 | 26 | 14 | 47 | 27 |
| hsa-miR-6717-5p | TGGGGATGTAGAGAGAAC | MIMAT0025846 | hsa-miR-6717-5p | 1 | 1 | 4 | 1 | 0 | 0 | 0 | 0 |
| hsa-miR-6718-5p | TAGTGGTCAGAGGGCTTATGAT | MIMAT0025849 | hsa-miR-6718-5p | 2 | 4 | 0 | 1 | 0 | 0 | 0 | 0 |
| hsa-miR-6720-3p | CGCGCCTGCAGGAACTGGTAGT | MIMAT0025851 | hsa-miR-6720-3p | 7 | 13 | 2 | 13 | 12 | 16 | 0 | 8 |
| hsa-miR-6720-5p | TTCCAGCCCTGGTAGGCGCCGCGT | MIMAT0027345 | hsa-miR-6720-5p | 128 | 140 | 136 | 101 | 94 | 79 | 79 | 103 |
| hsa-miR-6721-5p | TGGGCAGGGGCTTATTGTAGGAGT | MIMAT0025852 | hsa-miR-6721-5p | 154 | 410 | 204 | 241 | 213 | 204 | 31 | 234 |
| hsa-miR-6723-5p | CGGGGTAGTCCGAGTAACGTCGGGGC | NA | hsa-miR-6723-5p | 41 | 92 | 70 | 55 | 61 | 77 | 12 | 92 |
| hsa-miR-6724-5p | CTGGGCCCGCGGCGGGCGTGGGG | MIMAT0025856 | hsa-miR-6724-5p | 267 | 201 | 188 | 177 | 293 | 268 | 542 | 273 |
| hsa-miR-6726-3p | TGCTCGCCCTGTCTCCCGCT | MIMAT0027354 | hsa-miR-6726-3p | 15 | 8 | 8 | 5 | 10 | 11 | 23 | 11 |
| hsa-miR-6726-5p | CGGGAGCTGGGGTCTGCAGGT | MIMAT0027353 | hsa-miR-6726-5p | 69 | 70 | 72 | 60 | 61 | 74 | 125 | 82 |
| hsa-miR-6727-3p | CTCCTGCCACCTCCTCCGCAG | MIMAT0027356 | hsa-miR-6727-3p | 1 | 1 | 4 | 1 | 5 | 0 | 0 | 0 |
| hsa-miR-6727-5p | CTCGGGGCAGGCGGCTGGGAGCG | MIMAT0027355 | hsa-miR-6727-5p | 21 | 16 | 5 | 3 | 17 | 10 | 18 | 13 |
| hsa-miR-6728-3p | TTCTCTGCTCTGCTCTCCCCAG | MIMAT0027358 | hsa-miR-6728-3p | 0 | 0 | 1 | 0 | 0 | 0 | 0 | 0 |
| hsa-miR-6728-5p | TTGGGATGGTAGGACCAGAGG | MIMAT0027357 | hsa-miR-6728-5p | 50 | 6 | 11 | 4 | 0 | 7 | 0 | 3 |
| hsa-miR-6729-3p | TCATCCCCCTCGCCCTCTCAG | MIMAT0027360 | hsa-miR-6729-3p | 0 | 0 | 0 | 0 | 0 | 0 | 0 | 0 |
| hsa-miR-6729-5p | TGGGCGAGGGCGGCTGAGCGGC | MIMAT0027359 | hsa-miR-6729-5p | 19 | 23 | 12 | 12 | 15 | 12 | 14 | 7 |
| hsa-miR-6730-3p | CCTGACACCCCATCTGCCCTCT | MIMAT0027362 | hsa-miR-6730-3p | 0 | 0 | 0 | 0 | 0 | 0 | 0 | 0 |
| hsa-miR-6730-5p | AAAGGTGGAGGGGTTGTCAG | MIMAT0027361 | hsa-miR-6730-5p | 48 | 75 | 69 | 55 | 42 | 57 | 38 | 82 |
| hsa-miR-6732-3p | TAACCCTGTCCTCTCCCTCCC | MIMAT0027366 | hsa-miR-6732-3p | 9 | 8 | 7 | 8 | 9 | 5 | 0 | 4 |
| hsa-miR-6732-5p | TAGGGGGTGGCAGGCTGGCC | MIMAT0027365 | hsa-miR-6732-5p | 15 | 24 | 17 | 13 | 8 | 13 | 6 | 19 |
| hsa-miR-6733-3p | UCAGUGUCUGGAUUUCCUAG | MIMAT0027368 | hsa-miR-6733-3p | 0 | 0 | 0 | 0 | 0 | 0 | 0 | 0 |
| hsa-miR-6734-3p | CCCTTCCCTCACTCTTCTCTCAG | MIMAT0027370 | hsa-miR-6734-3p | 0 | 1 | 0 | 0 | 0 | 4 | 0 | 0 |
| hsa-miR-6734-5p | TTGAGGGGAGAATGAGG | MIMAT0027369 | hsa-miR-6734-5p | 46 | 100 | 61 | 72 | 124 | 112 | 162 | 102 |
| hsa-miR-6735-3p | AGGCCTGTGGCTCCTCCCTCAG | MIMAT0027372 | hsa-miR-6735-3p | 2 | 9 | 6 | 1 | 0 | 0 | 0 | 0 |
| hsa-miR-6735-5p | CAGGGCAGAGGGCACAGGAATCTG | MIMAT0027371 | hsa-miR-6735-5p | 12 | 23 | 12 | 16 | 39 | 34 | 44 | 32 |
| hsa-miR-6736-3p | TCAGCTCCTCTCTACCCACAG | MIMAT0027374 | hsa-miR-6736-3p | 0 | 0 | 0 | 0 | 0 | 0 | 0 | 0 |
| hsa-miR-6736-5p | CTGGGTGAGGGCATCTGTGGT | MIMAT0027373 | hsa-miR-6736-5p | 61 | 76 | 46 | 80 | 184 | 185 | 407 | 208 |
| hsa-miR-6737-3p | TCTGTGCTTCACCCCTACCCAG | MIMAT0027376 | hsa-miR-6737-3p | 3 | 6 | 7 | 2 | 0 | 0 | 0 | 0 |
| hsa-miR-6737-5p | TGGGGTGGTCGGCCCTGGAGGGG | MIMAT0027375 | hsa-miR-6737-5p | 2 | 4 | 1 | 3 | 7 | 4 | 15 | 9 |
| hsa-miR-6738-3p | CTTCTGCCTGCATTCTACTCCC | MIMAT0027378 | hsa-miR-6738-3p | 9 | 16 | 15 | 24 | 14 | 10 | 17 | 21 |
| hsa-miR-6738-5p | CGAGGGGTAGAAGAGCACAGGGG | MIMAT0027377 | hsa-miR-6738-5p | 5 | 9 | 10 | 3 | 9 | 9 | 12 | 3 |
| hsa-miR-6739-3p | ATTGTTCTGTCTTTCTCCC | MIMAT0027380 | hsa-miR-6739-3p | 0 | 0 | 0 | 0 | 0 | 2 | 0 | 0 |
| hsa-miR-6740-3p | TGTCTTCTCTCCTCCCAAACAG | MIMAT0027382 | hsa-miR-6740-3p | 0 | 0 | 2 | 0 | 0 | 0 | 0 | 0 |
| hsa-miR-6740-5p | AGTTTGGGATGGAGAGAGG | MIMAT0027381 | hsa-miR-6740-5p | 3 | 3 | 0 | 2 | 5 | 9 | 0 | 2 |
| hsa-miR-6741-3p | TCGGCTCTCTCCCTCACCCTAG | MIMAT0027384 | hsa-miR-6741-3p | 0 | 8 | 0 | 0 | 9 | 11 | 0 | 2 |
| hsa-miR-6741-5p | GTGGGTGCTGGTGGGAGCCGTG | MIMAT0027383 | hsa-miR-6741-5p | 25 | 34 | 13 | 15 | 60 | 69 | 156 | 74 |
| hsa-miR-6742-3p | CACCTGGGTTGTCCCCTCTAG | MIMAT0027386 | hsa-miR-6742-3p | 0 | 0 | 0 | 0 | 0 | 0 | 0 | 0 |
| hsa-miR-6742-5p | TGGGGTGGGACCCAGCTGTTG | MIMAT0027385 | hsa-miR-6742-5p | 1 | 1 | 3 | 0 | 17 | 15 | 53 | 22 |
| hsa-miR-6743-3p | GAGCCGCTCTTCTCCCTGCCCAC | MIMAT0027388 | hsa-miR-6743-3p | 23 | 48 | 30 | 30 | 41 | 33 | 44 | 32 |
| hsa-miR-6743-5p | AGGGGCAGGGACGGGTGGCC | MIMAT0027387 | hsa-miR-6743-5p | 12 | 19 | 7 | 9 | 14 | 13 | 34 | 10 |
| hsa-miR-6745 | CCTGGGTGGAAGAAGGTCTGGTT | MIMAT0027391 | hsa-miR-6745 | 0 | 0 | 0 | 0 | 0 | 0 | 0 | 3 |
| hsa-miR-6746-3p | CAGCCGCCGCCTGTCTCCAC | MIMAT0027393 | hsa-miR-6746-3p | 176 | 257 | 136 | 182 | 222 | 240 | 116 | 232 |
| hsa-miR-6746-5p | CCGGGAGAAGGAGGTGGCC | MIMAT0027392 | hsa-miR-6746-5p | 149 | 92 | 92 | 101 | 184 | 201 | 361 | 174 |
| hsa-miR-6747-3p | TCCTGCCTTCCTCTGCACCAG | MIMAT0027395 | hsa-miR-6747-3p | 11 | 15 | 37 | 16 | 8 | 11 | 0 | 3 |
| hsa-miR-6747-5p | GGGTGTGGAAAGAGGCAGAAC | MIMAT0027394 | hsa-miR-6747-5p | 0 | 4 | 0 | 0 | 0 | 0 | 0 | 0 |
| hsa-miR-6748-3p | TCCTGTCCCTGTCTCCTACAG | MIMAT0027397 | hsa-miR-6748-3p | 1 | 1 | 5 | 1 | 0 | 0 | 0 | 0 |
| hsa-miR-6748-5p | TGTGGGTGGGAAGGACTGGATT | MIMAT0027396 | hsa-miR-6748-5p | 5 | 11 | 6 | 5 | 8 | 2 | 0 | 23 |
| hsa-miR-6749-3p | CTCCTCCCCTGCCTGGCCCAG | MIMAT0027399 | hsa-miR-6749-3p | 42 | 36 | 106 | 38 | 15 | 18 | 18 | 21 |
| hsa-miR-6749-5p | TCGGGCCTGGGGTTGGGGGAGCTTT | MIMAT0027398 | hsa-miR-6749-5p | 0 | 0 | 3 | 2 | 0 | 0 | 0 | 0 |
| hsa-miR-675-3p | CTGTATGCCCTCACCGCTCAGC | MIMAT0006790 | hsa-miR-675-3p | 2 | 4 | 0 | 0 | 5 | 5 | 17 | 5 |
| hsa-miR-675-5p | TGGTGCGGAGAGGGCCCACAGT | MIMAT0004284 | hsa-miR-675-5p | 0 | 4 | 0 | 0 | 3 | 0 | 0 | 0 |
| hsa-miR-6750-3p | GAACTCACCCTCTGCTCCCAG | MIMAT0027401 | hsa-miR-6750-3p | 0 | 3 | 1 | 0 | 1 | 2 | 1 | 0 |
| hsa-miR-6750-5p | TCAGGGAACAGCTGGGTGAGCT | MIMAT0027400 | hsa-miR-6750-5p | 0 | 0 | 2 | 0 | 0 | 0 | 0 | 0 |
| hsa-miR-6751-3p | AGACTGAGCCTCTCTCTCTCC | MIMAT0027403 | hsa-miR-6751-3p | 0 | 9 | 0 | 0 | 8 | 6 | 23 | 6 |
| hsa-miR-6751-5p | TTGGGGGTGAGGTTGGTGTC | MIMAT0027402 | hsa-miR-6751-5p | 27 | 55 | 37 | 43 | 57 | 56 | 63 | 67 |
| hsa-miR-6752-3p | TCCCTGCCCCCATACTCCCAG | MIMAT0027405 | hsa-miR-6752-3p | 0 | 0 | 0 | 0 | 0 | 0 | 0 | 0 |
| hsa-miR-6753-3p | TGGTCTGTCTCTGCCCTGGCAT | MIMAT0027407 | hsa-miR-6753-3p | 5 | 14 | 10 | 5 | 16 | 12 | 42 | 16 |
| hsa-miR-6753-5p | CACCAGGGCAGAGCAGGGCTGA | MIMAT0027406 | hsa-miR-6753-5p | 0 | 0 | 0 | 0 | 0 | 0 | 0 | 0 |
| hsa-miR-6754-3p | TCTTCACCTGCCTCTGCCTGC | MIMAT0027409 | hsa-miR-6754-3p | 1 | 1 | 4 | 4 | 5 | 3 | 0 | 3 |
| hsa-miR-6754-5p | TGCCAGGGAGGCTGGTTTGGAGGAGT | MIMAT0027408 | hsa-miR-6754-5p | 0 | 0 | 0 | 0 | 0 | 0 | 0 | 0 |
| hsa-miR-6755-3p | TGTTGTCATGTTTTTTCCCTAG | MIMAT0027411 | hsa-miR-6755-3p | 0 | 0 | 0 | 0 | 0 | 0 | 0 | 0 |
| hsa-miR-6755-5p | TAGGGTAGACACTGACAACGT | MIMAT0027410 | hsa-miR-6755-5p | 0 | 0 | 0 | 0 | 2 | 0 | 0 | 0 |
| hsa-miR-6756-3p | TCCCCTTCCTCCCTGCCC | MIMAT0027413 | hsa-miR-6756-3p | 2 | 2 | 8 | 3 | 0 | 0 | 0 | 0 |
| hsa-miR-6756-5p | TGGGGCTGGAGGTGGGGCTGAGG | MIMAT0027412 | hsa-miR-6756-5p | 0 | 0 | 0 | 0 | 0 | 0 | 0 | 0 |
| hsa-miR-6757-3p | AACACTGGCCTTGCTATCCCCAT | MIMAT0027415 | hsa-miR-6757-3p | 15 | 27 | 27 | 29 | 21 | 23 | 5 | 30 |
| hsa-miR-6757-5p | TAGGGATGGGAGGCCAGGATG | MIMAT0027414 | hsa-miR-6757-5p | 51 | 49 | 36 | 32 | 61 | 56 | 32 | 84 |
| hsa-miR-6758-5p | TAGAGAGGGGAAGGATGTGATGTG | MIMAT0027416 | hsa-miR-6758-5p | 2 | 5 | 7 | 3 | 1 | 0 | 0 | 9 |
| hsa-miR-6759-3p | TGACCTTTGCCTCTCCCCTC | MIMAT0027419 | hsa-miR-6759-3p | 0 | 0 | 3 | 0 | 3 | 3 | 10 | 3 |
| hsa-miR-6759-5p | GTTGTGGGTGGGCAGAAGTCTG | MIMAT0027418 | hsa-miR-6759-5p | 2 | 0 | 0 | 3 | 14 | 4 | 11 | 5 |
| hsa-miR-676-3p | CTGTCCTAAGGTTGTTGAGTT | MIMAT0018204 | hsa-miR-676-3p | 10 | 19 | 17 | 20 | 13 | 14 | 14 | 7 |
| hsa-miR-676-5p | CTCTTCAACCTCAGGACTTGCA | MIMAT0018203 | hsa-miR-676-5p | 0 | 0 | 0 | 0 | 0 | 0 | 0 | 0 |
| hsa-miR-6761-3p | CCTCCTACGCTGCTCTCTCAC | MIMAT0027423 | hsa-miR-6761-3p | 2 | 2 | 0 | 4 | 0 | 2 | 0 | 3 |
| hsa-miR-6761-5p | TCTGAGAGAGCTCGATGGCAGG | MIMAT0027422 | hsa-miR-6761-5p | 16 | 5 | 4 | 1 | 2 | 2 | 0 | 0 |
| hsa-miR-6762-3p | CTGGCTGCTTCCCTTGGTCTCC | MIMAT0027425 | hsa-miR-6762-3p | 6 | 2 | 9 | 6 | 14 | 14 | 29 | 10 |
| hsa-miR-6762-5p | CGGGGCCATGGAGCAGCCTGTGT | MIMAT0027424 | hsa-miR-6762-5p | 0 | 2 | 0 | 0 | 3 | 0 | 0 | 0 |
| hsa-miR-6763-3p | CTCCCTGGCCTCTGCCCCCAG | MIMAT0027427 | hsa-miR-6763-3p | 3 | 0 | 3 | 0 | 0 | 0 | 0 | 2 |
| hsa-miR-6763-5p | TCCTGGGGAGTGGCTGGGG | MIMAT0027426 | hsa-miR-6763-5p | 8 | 4 | 13 | 11 | 38 | 31 | 103 | 49 |
| hsa-miR-6764-3p | TCTCTGGTCTTTCCTTGAC | MIMAT0027429 | hsa-miR-6764-3p | 0 | 0 | 0 | 0 | 0 | 0 | 0 | 0 |
| hsa-miR-6764-5p | TCCCAGGGTCTGGTCAGAGTTG | MIMAT0027428 | hsa-miR-6764-5p | 6 | 16 | 4 | 15 | 12 | 4 | 6 | 8 |
| hsa-miR-6765-3p | TCACCTGGCTGGCCCGCCCAG | MIMAT0027431 | hsa-miR-6765-3p | 0 | 0 | 0 | 0 | 1 | 0 | 0 | 0 |
| hsa-miR-6765-5p | GTGAGGCGGGGCCAGGAGGGTGTGT | MIMAT0027430 | hsa-miR-6765-5p | 0 | 0 | 0 | 0 | 0 | 2 | 0 | 0 |
| hsa-miR-6766-3p | TGATTGTCTTCCCCCACCCTC | MIMAT0027433 | hsa-miR-6766-3p | 24 | 34 | 20 | 35 | 48 | 51 | 96 | 54 |
| hsa-miR-6766-5p | CGGGTGGGAGCAGATCTTATTG | MIMAT0027432 | hsa-miR-6766-5p | 3 | 12 | 1 | 0 | 0 | 3 | 0 | 0 |
| hsa-miR-6767-3p | TCCCACGTGCTTCTCTTTCCGCA | MIMAT0027435 | hsa-miR-6767-3p | 0 | 0 | 0 | 0 | 0 | 0 | 0 | 0 |
| hsa-miR-6767-5p | TCGCAGACAGGGACACA | MIMAT0027434 | hsa-miR-6767-5p | 1 | 0 | 1 | 17 | 2 | 0 | 0 | 4 |
| hsa-miR-6768-5p | ACACAGGAAAAGCGGGGCCC | MIMAT0027436 | hsa-miR-6768-5p | 0 | 5 | 0 | 0 | 7 | 4 | 0 | 7 |
| hsa-miR-6769a-3p | GAGCCCCTCTCTGCTCTCCAG | MIMAT0027439 | hsa-miR-6769a-3p | 0 | 0 | 0 | 0 | 0 | 0 | 0 | 0 |
| hsa-miR-6769a-5p | AGGTGGGTATGGAGGAGCC | MIMAT0027438 | hsa-miR-6769a-5p | 2 | 0 | 0 | 0 | 1 | 0 | 0 | 3 |
| hsa-miR-6769b-3p | CCCTCTCTGTCCCACCCATAG | MIMAT0027621 | hsa-miR-6769b-3p | 0 | 1 | 0 | 0 | 0 | 0 | 0 | 0 |
| hsa-miR-6769b-5p | CTGGTGGGTGGGGAGG | MIMAT0027620 | hsa-miR-6769b-5p | 0 | 0 | 0 | 2 | 3 | 3 | 10 | 3 |
| hsa-miR-6770-3p | CCTGGCGGCTGTGTCTTCAC | MIMAT0027441 | hsa-miR-6770-3p | 19 | 53 | 29 | 31 | 70 | 47 | 63 | 51 |
| hsa-miR-6770-5p | TGAGAAGGCACAGCTTGCACGTG | MIMAT0027440 | hsa-miR-6770-5p | 6 | 13 | 8 | 10 | 24 | 17 | 29 | 22 |
| hsa-miR-6771-3p | CCAAACCCCTGTCTACCCGCA | MIMAT0027443 | hsa-miR-6771-3p | 0 | 0 | 0 | 0 | 1 | 3 | 0 | 2 |
| hsa-miR-6771-5p | CCTCGGGAGGGCATGGGCCAGG | MIMAT0027442 | hsa-miR-6771-5p | 7 | 7 | 5 | 9 | 1 | 1 | 0 | 4 |
| hsa-miR-6772-3p | TTGCTCCTGACTCTGTGCCCAC | MIMAT0027445 | hsa-miR-6772-3p | 3 | 6 | 2 | 11 | 2 | 4 | 0 | 8 |
| hsa-miR-6772-5p | CTGGGTGTAGGCTGGAGCTG | MIMAT0027444 | hsa-miR-6772-5p | 0 | 3 | 0 | 2 | 0 | 0 | 0 | 0 |
| hsa-miR-6773-5p | UUGGGCCCAGGAGUAAACAGGAU | MIMAT0027446 | hsa-miR-6773-5p | 0 | 0 | 0 | 0 | 0 | 0 | 0 | 0 |
| hsa-miR-6775-3p | AGGCCCTGTCCTCTGCCCC | MIMAT0027451 | hsa-miR-6775-3p | 13 | 7 | 14 | 9 | 4 | 1 | 2 | 0 |
| hsa-miR-6775-5p | CGGGGCATGGGGGAGGGAGGCTG | MIMAT0027450 | hsa-miR-6775-5p | 0 | 0 | 0 | 0 | 0 | 0 | 0 | 0 |
| hsa-miR-6776-3p | CAACCACCACTGTCTCTCCCCAT | MIMAT0027453 | hsa-miR-6776-3p | 6 | 7 | 14 | 4 | 1 | 2 | 0 | 4 |
| hsa-miR-6776-5p | TCTGGGTGCAGTGGGGGTT | MIMAT0027452 | hsa-miR-6776-5p | 0 | 0 | 0 | 0 | 0 | 0 | 0 | 2 |
| hsa-miR-6777-3p | TCCACTCTCCTGGCCCCCAG | MIMAT0027455 | hsa-miR-6777-3p | 0 | 0 | 3 | 0 | 0 | 1 | 0 | 3 |
| hsa-miR-6777-5p | ACGGGGAGTCAGGCAGTGGTGGAGT | MIMAT0027454 | hsa-miR-6777-5p | 39 | 55 | 41 | 34 | 55 | 25 | 0 | 40 |
| hsa-miR-6778-5p | AGTGGGAGGACAGGAGGCAGGTA | MIMAT0027456 | hsa-miR-6778-5p | 0 | 0 | 0 | 0 | 0 | 0 | 0 | 0 |
| hsa-miR-6779-3p | CAAGCCCTGTCTCCTCCCATC | MIMAT0027459 | hsa-miR-6779-3p | 0 | 10 | 2 | 2 | 1 | 0 | 0 | 0 |
| hsa-miR-6779-5p | TGGGAGGGGCTGGGTTTGGC | MIMAT0027458 | hsa-miR-6779-5p | 29 | 26 | 32 | 13 | 25 | 48 | 62 | 73 |
| hsa-miR-6780a-3p | CUCCUCUGUUUUCUUUCCUAG | MIMAT0027461 | hsa-miR-6780a-3p | 0 | 0 | 0 | 0 | 0 | 0 | 0 | 0 |
| hsa-miR-6780a-5p | TTGGGAGGGAAGACAGCTGGAGAGT | MIMAT0027460 | hsa-miR-6780a-5p | 3 | 27 | 2 | 27 | 5 | 28 | 0 | 37 |
| hsa-miR-6780b-3p | TCCCTTGTCTCCTTTCCCT | MIMAT0027573 | hsa-miR-6780b-3p | 20 | 50 | 41 | 15 | 19 | 25 | 28 | 25 |
| hsa-miR-6780b-5p | TGGGGAAGGCTTGGCAGGGA | MIMAT0027572 | hsa-miR-6780b-5p | 78 | 43 | 48 | 29 | 45 | 22 | 43 | 31 |
| hsa-miR-6781-3p | TGCCTCTTTTCCACGGCCTCAG | MIMAT0027463 | hsa-miR-6781-3p | 0 | 0 | 3 | 0 | 0 | 5 | 0 | 0 |
| hsa-miR-6781-5p | CGGGCCGGAGGTCAAGGGCGTCG | MIMAT0027462 | hsa-miR-6781-5p | 3 | 19 | 12 | 5 | 0 | 5 | 0 | 2 |
| hsa-miR-6782-3p | CACCTTTGTGTCCCCATCCTGCT | MIMAT0027465 | hsa-miR-6782-3p | 0 | 0 | 0 | 0 | 0 | 0 | 0 | 0 |
| hsa-miR-6782-5p | TAGGGGTGGGGGAATTCAGGGGTGT | MIMAT0027464 | hsa-miR-6782-5p | 0 | 4 | 0 | 1 | 0 | 0 | 0 | 2 |
| hsa-miR-6783-3p | TCCTGGGCTTCTCCTCTGTAG | MIMAT0027467 | hsa-miR-6783-3p | 7 | 12 | 17 | 5 | 7 | 4 | 14 | 5 |
| hsa-miR-6783-5p | TAGGGGAAAAGTCCTGATCCGGG | MIMAT0027466 | hsa-miR-6783-5p | 2 | 2 | 7 | 2 | 3 | 3 | 3 | 0 |
| hsa-miR-6784-3p | TCTCACCCCAACTCTGCCCC | MIMAT0027469 | hsa-miR-6784-3p | 0 | 0 | 0 | 0 | 0 | 1 | 0 | 3 |
| hsa-miR-6784-5p | CCGGGGCTTTGGGTGAGGGA | MIMAT0027468 | hsa-miR-6784-5p | 0 | 0 | 0 | 0 | 0 | 0 | 0 | 0 |
| hsa-miR-6785-3p | CACATCGCCCCACCTTCCCC | MIMAT0027471 | hsa-miR-6785-3p | 0 | 0 | 0 | 0 | 2 | 0 | 0 | 0 |
| hsa-miR-6785-5p | CTGGGAGGGCGTGGATGA | MIMAT0027470 | hsa-miR-6785-5p | 2 | 5 | 3 | 5 | 2 | 0 | 0 | 4 |
| hsa-miR-6786-3p | TGACGCCCCTTCTGATTCTGCC | MIMAT0027473 | hsa-miR-6786-3p | 10 | 49 | 17 | 21 | 62 | 58 | 119 | 46 |
| hsa-miR-6786-5p | TGCGGTGGGGCCGGAGGGGCGTC | MIMAT0027472 | hsa-miR-6786-5p | 4 | 5 | 1 | 3 | 8 | 9 | 23 | 30 |
| hsa-miR-6787-3p | TCTCAGCTGCTGCCCTCTCCAG | MIMAT0027475 | hsa-miR-6787-3p | 5 | 14 | 10 | 3 | 4 | 4 | 0 | 4 |
| hsa-miR-6787-5p | TGGCGGGGGTAGAGCTGGCTG | MIMAT0027474 | hsa-miR-6787-5p | 5 | 4 | 0 | 4 | 6 | 15 | 16 | 4 |
| hsa-miR-6788-3p | TTCGCCACTTCCCTCCCTGC | MIMAT0027477 | hsa-miR-6788-3p | 14 | 14 | 11 | 15 | 11 | 10 | 7 | 12 |
| hsa-miR-6788-5p | GGCTGGGAGAAGAGTGGTGA | MIMAT0027476 | hsa-miR-6788-5p | 2 | 1 | 1 | 4 | 9 | 4 | 0 | 0 |
| hsa-miR-6789-3p | CGGCGCCCGTGTCTCCTCCAG | MIMAT0027479 | hsa-miR-6789-3p | 97 | 120 | 195 | 90 | 47 | 45 | 71 | 86 |
| hsa-miR-6789-5p | GTAGGGGCGTCCCGGGCGCGCGGG | MIMAT0027478 | hsa-miR-6789-5p | 25 | 13 | 30 | 18 | 5 | 13 | 0 | 16 |
| hsa-miR-6790-3p | CCTCGGCGACCCCTCACTCACC | MIMAT0027481 | hsa-miR-6790-3p | 0 | 0 | 0 | 0 | 0 | 0 | 0 | 0 |
| hsa-miR-6790-5p | GTGAGTGTGGATTTGGCGGGGT | MIMAT0027480 | hsa-miR-6790-5p | 3 | 3 | 3 | 0 | 0 | 0 | 0 | 2 |
| hsa-miR-6791-3p | TGCCTCCTTGGTCTCCGGCAG | MIMAT0027483 | hsa-miR-6791-3p | 1 | 13 | 4 | 1 | 3 | 1 | 0 | 0 |
| hsa-miR-6791-5p | CCCCTGGGGCTGGGCAGGCGGA | MIMAT0027482 | hsa-miR-6791-5p | 1 | 3 | 4 | 1 | 0 | 0 | 0 | 0 |
| hsa-miR-6793-3p | TCCCCAACCCCTGCCCGCAG | MIMAT0027487 | hsa-miR-6793-3p | 0 | 10 | 1 | 0 | 2 | 4 | 0 | 2 |
| hsa-miR-6793-5p | TGTGGGTTCTGGGTTGGGGTG | MIMAT0027486 | hsa-miR-6793-5p | 14 | 25 | 19 | 26 | 30 | 32 | 37 | 51 |
| hsa-miR-6794-3p | TGCTCACTCTCAGTTCCTCCCT | MIMAT0027489 | hsa-miR-6794-3p | 0 | 0 | 0 | 0 | 0 | 0 | 0 | 0 |
| hsa-miR-6794-5p | CGCAGGGGGACTGGGGGTGAGC | MIMAT0027488 | hsa-miR-6794-5p | 0 | 0 | 0 | 0 | 0 | 0 | 0 | 0 |
| hsa-miR-6795-3p | CCACCCCTCGTTTCTTCCCCC | MIMAT0027491 | hsa-miR-6795-3p | 7 | 12 | 17 | 5 | 9 | 12 | 31 | 17 |
| hsa-miR-6795-5p | TTGGGGGGACAGGATGAGAGGCT | MIMAT0027490 | hsa-miR-6795-5p | 6 | 11 | 7 | 7 | 13 | 2 | 0 | 15 |
| hsa-miR-6796-3p | GAAGCTCTCCCCTCCCCGC | MIMAT0027493 | hsa-miR-6796-3p | 0 | 0 | 0 | 0 | 0 | 4 | 0 | 0 |
| hsa-miR-6796-5p | TTGTGGGGTTGGAGAGCTGGCT | MIMAT0027492 | hsa-miR-6796-5p | 12 | 17 | 10 | 9 | 17 | 8 | 27 | 22 |
| hsa-miR-6797-3p | TGCATGACCCTTCCCTCCCC | MIMAT0027495 | hsa-miR-6797-3p | 7 | 21 | 10 | 18 | 2 | 3 | 0 | 12 |
| hsa-miR-6797-5p | AGGAGGGAAGGGGCTGAGAACAGGA | MIMAT0027494 | hsa-miR-6797-5p | 0 | 2 | 0 | 0 | 0 | 0 | 0 | 0 |
| hsa-miR-6798-3p | CTACCCCCCATCCCCCTGT | MIMAT0027497 | hsa-miR-6798-3p | 0 | 2 | 2 | 0 | 0 | 2 | 0 | 0 |
| hsa-miR-6798-5p | GCCAGGGGGATGGGCGAGCTTG | MIMAT0027496 | hsa-miR-6798-5p | 1 | 1 | 4 | 1 | 0 | 0 | 0 | 12 |
| hsa-miR-6799-3p | TGCCCTGCATGGTGTCCCCACAG | MIMAT0027499 | hsa-miR-6799-3p | 0 | 0 | 0 | 2 | 0 | 0 | 0 | 0 |
| hsa-miR-6799-5p | AGGGGAGGTGTGCAGGGC | MIMAT0027498 | hsa-miR-6799-5p | 17 | 22 | 18 | 5 | 0 | 2 | 0 | 7 |
| hsa-miR-6800-3p | CACCTCTCCTGGCATCGCCCCC | MIMAT0027501 | hsa-miR-6800-3p | 1 | 18 | 6 | 1 | 4 | 4 | 0 | 4 |
| hsa-miR-6800-5p | TAGGTGACAGTCAGGGGCGGGGTG | MIMAT0027500 | hsa-miR-6800-5p | 7 | 2 | 7 | 10 | 4 | 7 | 0 | 2 |
| hsa-miR-6801-3p | ACCCCTGCCACCCACTGGCCCC | MIMAT0027503 | hsa-miR-6801-3p | 0 | 2 | 2 | 0 | 0 | 2 | 0 | 0 |
| hsa-miR-6801-5p | TGGTCAGAGGCAGCAGGAAATG | MIMAT0027502 | hsa-miR-6801-5p | 9 | 18 | 2 | 2 | 0 | 0 | 0 | 4 |
| hsa-miR-6802-3p | TCTTCACCCCTCTCACCTAAGC | MIMAT0027505 | hsa-miR-6802-3p | 12 | 15 | 13 | 18 | 18 | 15 | 0 | 1 |
| hsa-miR-6802-5p | GCTAGGTGGGGGGCTTGAAGCC | MIMAT0027504 | hsa-miR-6802-5p | 2 | 4 | 0 | 2 | 0 | 4 | 0 | 0 |
| hsa-miR-6803-3p | TCCCTCGCCTTCTCACCCTCAG | MIMAT0027507 | hsa-miR-6803-3p | 10 | 15 | 24 | 10 | 12 | 11 | 0 | 2 |
| hsa-miR-6803-5p | CTGGGGGTGGGGGGCTGGGC | MIMAT0027506 | hsa-miR-6803-5p | 0 | 4 | 0 | 0 | 2 | 0 | 0 | 0 |
| hsa-miR-6804-3p | CGCACCTGCCTCTCACCCAC | MIMAT0027509 | hsa-miR-6804-3p | 0 | 2 | 2 | 0 | 0 | 0 | 0 | 7 |
| hsa-miR-6804-5p | TGAGGGTGTCAGCAGGTGACGGT | MIMAT0027508 | hsa-miR-6804-5p | 43 | 101 | 69 | 52 | 44 | 41 | 65 | 76 |
| hsa-miR-6805-3p | TTGCTCTGCTCCCCCGCCCCCAG | MIMAT0027511 | hsa-miR-6805-3p | 4 | 11 | 11 | 3 | 0 | 0 | 0 | 0 |
| hsa-miR-6805-5p | TAGGGGGCGGCTTGTGGAGTGT | MIMAT0027510 | hsa-miR-6805-5p | 32 | 102 | 44 | 61 | 70 | 48 | 41 | 159 |
| hsa-miR-6806-3p | TGAAGCTCTGACATTCCTGC | MIMAT0027513 | hsa-miR-6806-3p | 0 | 0 | 0 | 0 | 0 | 0 | 0 | 0 |
| hsa-miR-6806-5p | TGTAGGCATGAGGCAGGGCCAAGG | MIMAT0027512 | hsa-miR-6806-5p | 0 | 0 | 0 | 0 | 0 | 0 | 0 | 0 |
| hsa-miR-6807-3p | CACTGCATTCCTGCTTGGCCC | MIMAT0027515 | hsa-miR-6807-3p | 2 | 0 | 0 | 0 | 0 | 0 | 0 | 0 |
| hsa-miR-6807-5p | GTGAGCCAGTGGAATGGAGAGG | MIMAT0027514 | hsa-miR-6807-5p | 0 | 1 | 0 | 0 | 3 | 3 | 4 | 1 |
| hsa-miR-6808-3p | GTGTGACCACCGTTCCTGCAG | MIMAT0027517 | hsa-miR-6808-3p | 18 | 21 | 49 | 23 | 0 | 2 | 0 | 2 |
| hsa-miR-6808-5p | AGGCAGGGAGGTGGGACCA | MIMAT0027516 | hsa-miR-6808-5p | 2 | 2 | 8 | 5 | 0 | 0 | 0 | 6 |
| hsa-miR-6809-3p | CTTCTCTTCTCTCCTTCCC | MIMAT0027519 | hsa-miR-6809-3p | 0 | 0 | 0 | 0 | 0 | 0 | 0 | 0 |
| hsa-miR-6809-5p | TGGCAAGGAAAGAAGAGGATC | MIMAT0027518 | hsa-miR-6809-5p | 8 | 6 | 11 | 8 | 32 | 20 | 32 | 31 |
| hsa-miR-6810-3p | TCCCCTGCTCCCTTGTTCCCCAG | MIMAT0027521 | hsa-miR-6810-3p | 0 | 0 | 0 | 0 | 0 | 2 | 0 | 0 |
| hsa-miR-6810-5p | ATGGGGACAGGGATCAGCATGGC | MIMAT0027520 | hsa-miR-6810-5p | 4 | 8 | 2 | 13 | 4 | 9 | 3 | 5 |
| hsa-miR-6811-5p | TATGCAGGCCTGTGTACAGCACT | MIMAT0027522 | hsa-miR-6811-5p | 1 | 0 | 0 | 0 | 1 | 2 | 0 | 0 |
| hsa-miR-6812-3p | CCGCTCTTCCCCTGACCCC | MIMAT0027525 | hsa-miR-6812-3p | 0 | 2 | 2 | 1 | 0 | 4 | 0 | 1 |
| hsa-miR-6812-5p | TGGGGTGAGATGGGGAGGAGC | MIMAT0027524 | hsa-miR-6812-5p | 1 | 6 | 4 | 3 | 11 | 2 | 9 | 5 |
| hsa-miR-6813-3p | AACCTTGGCCCCTCTCCCC | MIMAT0027527 | hsa-miR-6813-3p | 13 | 11 | 7 | 8 | 3 | 4 | 0 | 9 |
| hsa-miR-6813-5p | CAGGGGCTGGGGTTTCAGGTT | MIMAT0027526 | hsa-miR-6813-5p | 12 | 12 | 17 | 7 | 6 | 6 | 11 | 6 |
| hsa-miR-6814-3p | ACTCGCATCCTTCCCTTGGC | MIMAT0027529 | hsa-miR-6814-3p | 0 | 3 | 0 | 0 | 0 | 0 | 0 | 0 |
| hsa-miR-6814-5p | UCCCAAGGGUGAGAUGCUGCCA | MIMAT0027528 | hsa-miR-6814-5p | 0 | 0 | 0 | 0 | 0 | 0 | 0 | 0 |
| hsa-miR-6815-3p | TGGCTTCTCTTGCACACCC | MIMAT0027531 | hsa-miR-6815-3p | 0 | 0 | 0 | 0 | 0 | 0 | 0 | 0 |
| hsa-miR-6815-5p | TAGGTGGCGCCGGAGGAGTCATT | MIMAT0027530 | hsa-miR-6815-5p | 101 | 169 | 106 | 115 | 310 | 288 | 717 | 367 |
| hsa-miR-6816-3p | AAGGACCTGCACCTTCGCC | MIMAT0027533 | hsa-miR-6816-3p | 0 | 11 | 2 | 2 | 5 | 4 | 12 | 5 |
| hsa-miR-6816-5p | AGTGGGGCGGGGCAGGTCC | MIMAT0027532 | hsa-miR-6816-5p | 0 | 7 | 0 | 0 | 0 | 2 | 0 | 0 |
| hsa-miR-6817-3p | TCTCTCTGACTCCATGGCAG | MIMAT0027535 | hsa-miR-6817-3p | 17 | 24 | 24 | 12 | 12 | 3 | 1 | 17 |
| hsa-miR-6817-5p | TTCTGCCATAGGAAGCTTGGAGTG | MIMAT0027534 | hsa-miR-6817-5p | 0 | 0 | 0 | 0 | 0 | 0 | 0 | 0 |
| hsa-miR-6818-3p | TTGTTGTCTCTTGTTCCTCACAC | MIMAT0027537 | hsa-miR-6818-3p | 0 | 3 | 1 | 0 | 0 | 0 | 0 | 0 |
| hsa-miR-6818-5p | TTGTGTGAGTACAGAGAGCATC | MIMAT0027536 | hsa-miR-6818-5p | 0 | 0 | 0 | 0 | 0 | 0 | 0 | 0 |
| hsa-miR-6819-3p | AAGCCTCTGTCCCCACCCC | MIMAT0027539 | hsa-miR-6819-3p | 16 | 31 | 23 | 24 | 2 | 10 | 0 | 13 |
| hsa-miR-6819-5p | TTGGGGTGGAGGGCCAAGG | MIMAT0027538 | hsa-miR-6819-5p | 11 | 3 | 11 | 3 | 6 | 3 | 12 | 9 |
| hsa-miR-6820-3p | TGTGACTTCTCCCCTGCCAC | MIMAT0027541 | hsa-miR-6820-3p | 0 | 2 | 1 | 0 | 0 | 3 | 0 | 0 |
| hsa-miR-6820-5p | TGCGGCAGAGCTGGGGTCACC | MIMAT0027540 | hsa-miR-6820-5p | 75 | 125 | 69 | 54 | 82 | 69 | 70 | 77 |
| hsa-miR-6821-3p | TGACCTCTCCGCTCCGCAC | MIMAT0027543 | hsa-miR-6821-3p | 1 | 1 | 4 | 2 | 0 | 0 | 0 | 1 |
| hsa-miR-6821-5p | TGCGTGGTGGCTCGAGGCG | MIMAT0027542 | hsa-miR-6821-5p | 9 | 0 | 3 | 3 | 22 | 22 | 57 | 29 |
| hsa-miR-6822-5p | CCAGGGAACCAGTTGGGGCTT | MIMAT0027544 | hsa-miR-6822-5p | 0 | 0 | 0 | 0 | 0 | 1 | 0 | 0 |
| hsa-miR-6823-3p | TGAGCCTCTCCTTCCCTCC | MIMAT0027547 | hsa-miR-6823-3p | 1 | 3 | 2 | 4 | 2 | 0 | 0 | 0 |
| hsa-miR-6823-5p | TCAGGGTTGGTAGGGGTTGCT | MIMAT0027546 | hsa-miR-6823-5p | 10 | 12 | 12 | 4 | 19 | 12 | 22 | 20 |
| hsa-miR-6824-3p | TCTCTGGTCTTGCCACCCC | MIMAT0027549 | hsa-miR-6824-3p | 16 | 21 | 2 | 9 | 17 | 16 | 40 | 16 |
| hsa-miR-6824-5p | AGGGGAGGTTGGGCCAGGGAT | MIMAT0027548 | hsa-miR-6824-5p | 9 | 11 | 7 | 9 | 18 | 12 | 7 | 12 |
| hsa-miR-6825-3p | CGCTGACCCGCCTTCTCCGC | MIMAT0027551 | hsa-miR-6825-3p | 1 | 7 | 0 | 4 | 6 | 5 | 0 | 1 |
| hsa-miR-6825-5p | TGGGGAGGTGTGGAGTCAGCA | MIMAT0027550 | hsa-miR-6825-5p | 44 | 33 | 43 | 26 | 16 | 33 | 0 | 17 |
| hsa-miR-6826-3p | CTCCCCTCTCTTTCCTGTTCAG | MIMAT0027553 | hsa-miR-6826-3p | 0 | 1 | 1 | 1 | 2 | 0 | 0 | 0 |
| hsa-miR-6826-5p | CAACAGGAAAGAGGTGGGAC | MIMAT0027552 | hsa-miR-6826-5p | 2 | 0 | 0 | 0 | 0 | 0 | 0 | 3 |
| hsa-miR-6827-3p | ACCGTCTCTTCTGTTCCCC | MIMAT0027555 | hsa-miR-6827-3p | 2 | 6 | 8 | 3 | 9 | 10 | 25 | 15 |
| hsa-miR-6827-5p | TGGGAGCCATGAGGGTCTGTGCT | MIMAT0027554 | hsa-miR-6827-5p | 14 | 24 | 22 | 43 | 46 | 32 | 79 | 28 |
| hsa-miR-6828-3p | CATCTGCTCTCTTGTTCCCAG | MIMAT0027557 | hsa-miR-6828-3p | 0 | 1 | 1 | 0 | 0 | 0 | 0 | 0 |
| hsa-miR-6828-5p | AGGAAGCAAGAGAACCCTG | MIMAT0027556 | hsa-miR-6828-5p | 0 | 0 | 0 | 0 | 0 | 0 | 0 | 0 |
| hsa-miR-6829-3p | TGCCTCCTCCGTGGCCTCAG | MIMAT0027559 | hsa-miR-6829-3p | 42 | 45 | 139 | 41 | 12 | 4 | 10 | 4 |
| hsa-miR-6829-5p | CGTGGGCTGCTGAGAAGGGGCAGG | MIMAT0027558 | hsa-miR-6829-5p | 1 | 1 | 6 | 1 | 0 | 0 | 0 | 0 |
| hsa-miR-6830-3p | TGTCTTTCTTCTCTCCCTTGC | MIMAT0027561 | hsa-miR-6830-3p | 0 | 2 | 1 | 3 | 0 | 0 | 0 | 0 |
| hsa-miR-6830-5p | CCAAGGAAGGAGGCTGGACAT | MIMAT0027560 | hsa-miR-6830-5p | 0 | 1 | 0 | 0 | 0 | 0 | 0 | 0 |
| hsa-miR-6831-5p | TAGGTAGAGTGTGAGGAGGAGG | MIMAT0027562 | hsa-miR-6831-5p | 0 | 1 | 0 | 0 | 0 | 2 | 0 | 0 |
| hsa-miR-6832-3p | ACCCTTTTTCTCTTTCCCAG | MIMAT0027565 | hsa-miR-6832-3p | 0 | 0 | 0 | 0 | 0 | 0 | 0 | 0 |
| hsa-miR-6832-5p | AGTAGAGAGGAAAAGTTAGGGTC | MIMAT0027564 | hsa-miR-6832-5p | 0 | 2 | 2 | 2 | 9 | 6 | 2 | 7 |
| hsa-miR-6833-3p | TTTCTCTCTCCACTTCCTCAG | MIMAT0027567 | hsa-miR-6833-3p | 3 | 0 | 3 | 0 | 0 | 0 | 0 | 0 |
| hsa-miR-6833-5p | GTGTGGAAGATGGGAGGAGAA | MIMAT0027566 | hsa-miR-6833-5p | 2 | 2 | 7 | 2 | 0 | 0 | 0 | 4 |
| hsa-miR-6834-5p | GTGAGGGACTGGGATTTG | MIMAT0027568 | hsa-miR-6834-5p | 2 | 0 | 0 | 10 | 2 | 3 | 0 | 2 |
| hsa-miR-6835-3p | AAAAGCACUUUUCUGUCUCCCAG | MIMAT0027571 | hsa-miR-6835-3p | 0 | 0 | 0 | 0 | 0 | 0 | 0 | 0 |
| hsa-miR-6835-5p | AGGGGGTAGAAAGTGGCTGAAGT | MIMAT0027570 | hsa-miR-6835-5p | 3 | 5 | 3 | 0 | 0 | 2 | 0 | 0 |
| hsa-miR-6836-3p | ATGCCTCCCCCGGCCCCGCAG | MIMAT0027575 | hsa-miR-6836-3p | 5 | 8 | 17 | 7 | 4 | 9 | 0 | 6 |
| hsa-miR-6836-5p | TCCGCAGGGCCCTGGCGCAGGCAT | MIMAT0027574 | hsa-miR-6836-5p | 25 | 70 | 28 | 40 | 26 | 12 | 15 | 37 |
| hsa-miR-6837-3p | CCTTCACTGTGACTCTGCTGC | MIMAT0027577 | hsa-miR-6837-3p | 0 | 0 | 0 | 0 | 1 | 1 | 0 | 0 |
| hsa-miR-6837-5p | ACCAGGGCCAGCAGGGAATGTC | MIMAT0027576 | hsa-miR-6837-5p | 61 | 49 | 28 | 58 | 59 | 45 | 52 | 47 |
| hsa-miR-6838-3p | AAGTCCTGCTTCTGTTGC | MIMAT0027579 | hsa-miR-6838-3p | 0 | 0 | 0 | 0 | 0 | 0 | 0 | 0 |
| hsa-miR-6838-5p | AAGCAGCAGTGGCAAGACTCCT | MIMAT0027578 | hsa-miR-6838-5p | 4 | 0 | 1 | 4 | 2 | 3 | 0 | 0 |
| hsa-miR-6840-3p | CCCAGGACTTTGTGCGGGGTGCC | MIMAT0027583 | hsa-miR-6840-3p | 0 | 0 | 0 | 0 | 0 | 0 | 0 | 0 |
| hsa-miR-6840-5p | CACCCCCGGGCAAAGACCTGC | MIMAT0027582 | hsa-miR-6840-5p | 0 | 0 | 0 | 0 | 0 | 0 | 0 | 0 |
| hsa-miR-6842-3p | TTGGCTGGTCTCTGCTCCGC | MIMAT0027587 | hsa-miR-6842-3p | 34 | 25 | 54 | 50 | 26 | 37 | 51 | 53 |
| hsa-miR-6842-5p | TGGGGGTGGTCTCTAGCCAAGG | MIMAT0027586 | hsa-miR-6842-5p | 4 | 0 | 0 | 2 | 1 | 0 | 0 | 2 |
| hsa-miR-6843-3p | ATGGTCTCCTGTTCTCTGCAG | MIMAT0027588 | hsa-miR-6843-3p | 0 | 2 | 0 | 0 | 0 | 0 | 0 | 0 |
| hsa-miR-6845-3p | CCTCTCCTCCCTGTGCCCCAG | MIMAT0027591 | hsa-miR-6845-3p | 0 | 0 | 0 | 0 | 0 | 2 | 0 | 0 |
| hsa-miR-6845-5p | CGGGGCCAGAGCAGAGAGC | MIMAT0027590 | hsa-miR-6845-5p | 2 | 4 | 9 | 13 | 5 | 4 | 16 | 8 |
| hsa-miR-6846-5p | TGGGGGCTGGATGGGGTAGAGT | MIMAT0027592 | hsa-miR-6846-5p | 0 | 0 | 0 | 2 | 4 | 4 | 15 | 4 |
| hsa-miR-6847-3p | CTCATGTGTCTGTCCTCTTCCT | MIMAT0027595 | hsa-miR-6847-3p | 0 | 0 | 0 | 0 | 0 | 0 | 0 | 2 |
| hsa-miR-6847-5p | ACAGAGGACAGTGGAGTGTGAGCT | MIMAT0027594 | hsa-miR-6847-5p | 1 | 11 | 2 | 11 | 6 | 7 | 0 | 6 |
| hsa-miR-6848-3p | CTGTGGTCTCTTGGCCCCCAGT | MIMAT0027597 | hsa-miR-6848-3p | 0 | 0 | 0 | 0 | 0 | 0 | 0 | 0 |
| hsa-miR-6848-5p | TGGGGGCTGGGATGGGCCATGGTG | MIMAT0027596 | hsa-miR-6848-5p | 20 | 31 | 15 | 15 | 26 | 18 | 18 | 24 |
| hsa-miR-6849-3p | ACCAGCCTGTGTCCACCTCCAG | MIMAT0027599 | hsa-miR-6849-3p | 8 | 7 | 17 | 5 | 7 | 1 | 0 | 9 |
| hsa-miR-6849-5p | AGTGGATAGGGGAGTGTG | MIMAT0027598 | hsa-miR-6849-5p | 0 | 0 | 0 | 0 | 0 | 2 | 0 | 0 |
| hsa-miR-6850-5p | GTGCGGAACGCTGGCCGGGGCG | MIMAT0027600 | hsa-miR-6850-5p | 60 | 86 | 67 | 50 | 44 | 34 | 44 | 82 |
| hsa-miR-6851-3p | TGGCCCTTTGTACCCCTCCAG | MIMAT0027603 | hsa-miR-6851-3p | 33 | 60 | 73 | 42 | 32 | 20 | 16 | 25 |
| hsa-miR-6851-5p | GAGGAGGTGGTACTAGGGGCCAGT | MIMAT0027602 | hsa-miR-6851-5p | 91 | 89 | 73 | 53 | 92 | 85 | 170 | 140 |
| hsa-miR-6852-3p | TGATGTCCTCTGTTCCTC | MIMAT0027605 | hsa-miR-6852-3p | 1 | 5 | 6 | 1 | 0 | 0 | 0 | 0 |
| hsa-miR-6852-5p | CCCTGGGGTTCTGAGGACATGC | MIMAT0027604 | hsa-miR-6852-5p | 42 | 49 | 19 | 51 | 90 | 87 | 131 | 71 |
| hsa-miR-6853-3p | TGTTCATTGGAACCCTGCGCAG | MIMAT0027607 | hsa-miR-6853-3p | 27 | 37 | 82 | 30 | 5 | 8 | 17 | 10 |
| hsa-miR-6854-3p | TGCGTTTCTCCTCTTGAGCAG | MIMAT0027609 | hsa-miR-6854-3p | 0 | 0 | 0 | 0 | 0 | 0 | 0 | 0 |
| hsa-miR-6855-3p | AGACTGACCTTCAACCCCACAG | MIMAT0027611 | hsa-miR-6855-3p | 0 | 2 | 0 | 0 | 0 | 1 | 0 | 0 |
| hsa-miR-6855-5p | TTGGGGTTTGGGGTGCAGACATTGC | MIMAT0027610 | hsa-miR-6855-5p | 21 | 70 | 31 | 49 | 78 | 50 | 62 | 75 |
| hsa-miR-6856-3p | TCGCTACAGCCCTGTGATCTTTCC | MIMAT0027613 | hsa-miR-6856-3p | 0 | 1 | 1 | 0 | 5 | 2 | 9 | 4 |
| hsa-miR-6856-5p | AGAGAGGAGCAGTGGTGCTG | MIMAT0027612 | hsa-miR-6856-5p | 0 | 6 | 0 | 0 | 0 | 0 | 0 | 0 |
| hsa-miR-6857-3p | TGACTGAGCTTCTCCCCAC | MIMAT0027615 | hsa-miR-6857-3p | 0 | 0 | 0 | 0 | 0 | 0 | 0 | 0 |
| hsa-miR-6857-5p | TGTTGGGGATTGGGTCAGGCCA | MIMAT0027614 | hsa-miR-6857-5p | 5 | 5 | 6 | 3 | 2 | 0 | 0 | 3 |
| hsa-miR-6858-3p | CAGCCAGCCCCTGCTCACCCC | MIMAT0027617 | hsa-miR-6858-3p | 53 | 102 | 42 | 75 | 77 | 68 | 32 | 91 |
| hsa-miR-6858-5p | TGAGGAGGGGCTGGCAGGGAC | MIMAT0027616 | hsa-miR-6858-5p | 31 | 37 | 31 | 17 | 11 | 22 | 23 | 36 |
| hsa-miR-6859-3p | CCTGACCCCCATGTTGCCTCTGT | MIMAT0027619 | hsa-miR-6859-3p | 1 | 0 | 1 | 0 | 2 | 0 | 0 | 1 |
| hsa-miR-6859-5p | AGAGGAACATGGGCTCAGGAC | MIMAT0027618 | hsa-miR-6859-5p | 0 | 2 | 0 | 1 | 0 | 0 | 0 | 0 |
| hsa-miR-6860 | ACTGGGCAGGGCTGTGGTGAGT | MIMAT0027622 | hsa-miR-6860 | 2 | 0 | 0 | 3 | 7 | 8 | 26 | 9 |
| hsa-miR-6861-3p | TGGACCTCTCCTCCCCAGTCC | MIMAT0027624 | hsa-miR-6861-3p | 0 | 0 | 0 | 4 | 0 | 0 | 0 | 0 |
| hsa-miR-6861-5p | ACTGGGTAGGTGGGGCTCCAGG | MIMAT0027623 | hsa-miR-6861-5p | 11 | 2 | 0 | 0 | 5 | 4 | 16 | 4 |
| hsa-miR-6862-3p | CCTCACCCAGCTCTCTGGCCCTCT | MIMAT0027626 | hsa-miR-6862-3p | 0 | 7 | 0 | 0 | 0 | 0 | 0 | 0 |
| hsa-miR-6862-5p | CGGGCATGCTGGGAGAGACTT | MIMAT0027625 | hsa-miR-6862-5p | 14 | 55 | 21 | 15 | 35 | 38 | 78 | 44 |
| hsa-miR-6864-5p | TTGAAGGGACAAGTCAGATATGC | MIMAT0027628 | hsa-miR-6864-5p | 0 | 0 | 0 | 3 | 0 | 0 | 0 | 0 |
| hsa-miR-6865-3p | ACACCCTCTTTCCCTACCGC | MIMAT0027631 | hsa-miR-6865-3p | 0 | 0 | 0 | 0 | 1 | 2 | 0 | 0 |
| hsa-miR-6865-5p | ATAGGTGGCAGAGGAGGGACTTC | MIMAT0027630 | hsa-miR-6865-5p | 19 | 23 | 16 | 5 | 20 | 17 | 27 | 25 |
| hsa-miR-6866-3p | GATCCCTTTATCTGTCCTCT | MIMAT0027633 | hsa-miR-6866-3p | 0 | 0 | 0 | 0 | 0 | 0 | 0 | 0 |
| hsa-miR-6866-5p | TTAGAGGCTGGAATAGAGATTC | MIMAT0027632 | hsa-miR-6866-5p | 2 | 10 | 6 | 2 | 6 | 2 | 6 | 9 |
| hsa-miR-6867-5p | TGTGTGTGTAGAGGAAGAAGGG | MIMAT0027634 | hsa-miR-6867-5p | 0 | 0 | 0 | 5 | 0 | 0 | 0 | 0 |
| hsa-miR-6868-3p | TTCCTTCTGTTGTCTGTGCAG | MIMAT0027637 | hsa-miR-6868-3p | 0 | 0 | 1 | 0 | 0 | 0 | 0 | 0 |
| hsa-miR-6869-5p | GTGAGTAGTGGCGCGCGGCGG | MIMAT0027638 | hsa-miR-6869-5p | 98 | 107 | 115 | 52 | 56 | 73 | 143 | 97 |
| hsa-miR-6870-3p | GCTCATCCCCATCTCCTTTCAG | MIMAT0027641 | hsa-miR-6870-3p | 1 | 2 | 5 | 1 | 2 | 0 | 0 | 2 |
| hsa-miR-6870-5p | TGGGGGAGATGGGGGTTG | MIMAT0027640 | hsa-miR-6870-5p | 0 | 0 | 0 | 1 | 3 | 3 | 13 | 7 |
| hsa-miR-6871-3p | CAGCACCCTGTGGCTCCCAC | MIMAT0027643 | hsa-miR-6871-3p | 0 | 0 | 0 | 0 | 0 | 4 | 0 | 2 |
| hsa-miR-6871-5p | TCATGGGAGTTCGGGGTGGTTGT | MIMAT0027642 | hsa-miR-6871-5p | 13 | 31 | 33 | 19 | 20 | 12 | 8 | 31 |
| hsa-miR-6872-3p | CATGCCTCCTGCCGCGGTC | MIMAT0027645 | hsa-miR-6872-3p | 3 | 1 | 4 | 1 | 0 | 0 | 1 | 0 |
| hsa-miR-6873-3p | TTCTCTCTGTCTTTCTCTCTC | MIMAT0027647 | hsa-miR-6873-3p | 1 | 2 | 2 | 0 | 2 | 0 | 0 | 3 |
| hsa-miR-6873-5p | GCAGAGGGAATACAGAGGGC | MIMAT0027646 | hsa-miR-6873-5p | 0 | 0 | 0 | 3 | 2 | 4 | 0 | 0 |
| hsa-miR-6874-5p | ATGGAGCTGGAACCAGATCAGGC | MIMAT0027648 | hsa-miR-6874-5p | 0 | 0 | 0 | 7 | 2 | 0 | 0 | 4 |
| hsa-miR-6875-3p | ATTCTTCCTGCCCTGGCTCCAT | MIMAT0027651 | hsa-miR-6875-3p | 25 | 82 | 20 | 33 | 55 | 40 | 82 | 67 |
| hsa-miR-6875-5p | TGAGGGACCCAGGACAGG | MIMAT0027650 | hsa-miR-6875-5p | 39 | 23 | 44 | 34 | 35 | 26 | 33 | 30 |
| hsa-miR-6876-3p | AGCTGTCTGTGTTTTCCTTCTCA | MIMAT0027653 | hsa-miR-6876-3p | 0 | 1 | 2 | 0 | 0 | 0 | 0 | 0 |
| hsa-miR-6876-5p | CAGGAAGGAGACAGGCAGTTC | MIMAT0027652 | hsa-miR-6876-5p | 4 | 5 | 5 | 2 | 11 | 4 | 0 | 0 |
| hsa-miR-6877-3p | CCTCTGCCCTTGGCCTCCCCAG | MIMAT0027655 | hsa-miR-6877-3p | 0 | 0 | 2 | 0 | 0 | 0 | 0 | 1 |
| hsa-miR-6877-5p | AGGGCCGAAGGGTGGAAGCTGC | MIMAT0027654 | hsa-miR-6877-5p | 25 | 59 | 32 | 42 | 68 | 60 | 125 | 92 |
| hsa-miR-6878-3p | CTGGCCTCTTCTTTCTCCTAG | MIMAT0027657 | hsa-miR-6878-3p | 1 | 0 | 1 | 0 | 2 | 0 | 0 | 0 |
| hsa-miR-6878-5p | AGGGAGAAAGCTAGAAGCTGA | MIMAT0027656 | hsa-miR-6878-5p | 0 | 0 | 0 | 0 | 2 | 0 | 0 | 0 |
| hsa-miR-6879-3p | CCTGTCACCCGCTCCTTGCCCAG | MIMAT0027659 | hsa-miR-6879-3p | 8 | 18 | 25 | 7 | 7 | 3 | 13 | 6 |
| hsa-miR-6879-5p | AGGGCAGGGAAGGTGGGAGAG | MIMAT0027658 | hsa-miR-6879-5p | 2 | 0 | 0 | 2 | 0 | 0 | 0 | 0 |
| hsa-miR-6880-3p | CCGCCTTCTCTCCTCCCCC | MIMAT0027661 | hsa-miR-6880-3p | 12 | 27 | 18 | 18 | 10 | 21 | 0 | 16 |
| hsa-miR-6880-5p | TGGTGGAGGAAGAGGGCAGCT | MIMAT0027660 | hsa-miR-6880-5p | 10 | 12 | 8 | 5 | 9 | 7 | 12 | 8 |
| hsa-miR-6881-3p | ATCCTCTTTCGTCCTTCCCACT | MIMAT0027663 | hsa-miR-6881-3p | 6 | 22 | 10 | 9 | 9 | 10 | 11 | 8 |
| hsa-miR-6881-5p | CTGGGGTAAGGATAGGAGGGTC | MIMAT0027662 | hsa-miR-6881-5p | 10 | 28 | 6 | 4 | 7 | 3 | 0 | 6 |
| hsa-miR-6882-3p | TGCTGCCTCTCCTCTTGCCTGC | MIMAT0027665 | hsa-miR-6882-3p | 12 | 10 | 15 | 22 | 17 | 31 | 51 | 24 |
| hsa-miR-6882-5p | TACAAGTCAGGAGCTGAAGCAGC | MIMAT0027664 | hsa-miR-6882-5p | 2 | 2 | 0 | 2 | 2 | 0 | 0 | 2 |
| hsa-miR-6883-5p | AGGGAGGGTGTGGTATGGATGT | MIMAT0027666 | hsa-miR-6883-5p | 0 | 0 | 0 | 0 | 5 | 0 | 0 | 0 |
| hsa-miR-6884-3p | CCCATCACCTTTCCGTCTCCCC | MIMAT0027669 | hsa-miR-6884-3p | 1 | 3 | 4 | 1 | 0 | 0 | 0 | 0 |
| hsa-miR-6884-5p | AGAGGCTGAGAAGGTGATGTTGGC | MIMAT0027668 | hsa-miR-6884-5p | 2 | 0 | 0 | 11 | 11 | 0 | 0 | 0 |
| hsa-miR-6885-3p | CTTTGCTTCCTGCTCCCCT | MIMAT0027671 | hsa-miR-6885-3p | 0 | 2 | 0 | 5 | 4 | 2 | 7 | 2 |
| hsa-miR-6885-5p | GAGGGGGGCACTGCGCAAGC | MIMAT0027670 | hsa-miR-6885-5p | 0 | 0 | 3 | 0 | 2 | 9 | 0 | 1 |
| hsa-miR-6886-3p | TGCCCTTCTCTCCTCCTGCCT | MIMAT0027673 | hsa-miR-6886-3p | 8 | 6 | 2 | 2 | 16 | 14 | 19 | 7 |
| hsa-miR-6886-5p | CCCGCAGGTGAGATGAGGGCT | MIMAT0027672 | hsa-miR-6886-5p | 3 | 9 | 9 | 13 | 14 | 10 | 23 | 10 |
| hsa-miR-6888-3p | ATCTGTCTCGATTGTTTCC | MIMAT0027677 | hsa-miR-6888-3p | 0 | 0 | 0 | 0 | 0 | 0 | 0 | 0 |
| hsa-miR-6889-3p | TCTGTGCCCCTACTTCCCAG | MIMAT0027679 | hsa-miR-6889-3p | 1 | 1 | 5 | 2 | 0 | 0 | 0 | 0 |
| hsa-miR-6889-5p | TCGGGGAGTCTGGGGTCCGGAAT | MIMAT0027678 | hsa-miR-6889-5p | 6 | 9 | 9 | 5 | 11 | 12 | 24 | 13 |
| hsa-miR-6890-3p | CCCACTGCCTATGCCCCAC | MIMAT0027681 | hsa-miR-6890-3p | 0 | 0 | 0 | 4 | 0 | 0 | 0 | 0 |
| hsa-miR-6890-5p | CATGGGGTAGGGCAGAGTAGG | MIMAT0027680 | hsa-miR-6890-5p | 3 | 8 | 13 | 5 | 0 | 1 | 0 | 4 |
| hsa-miR-6891-5p | GTAAGGAGGGGGATGAGGGGT | MIMAT0027682 | hsa-miR-6891-5p | 17 | 22 | 24 | 19 | 13 | 12 | 11 | 36 |
| hsa-miR-6892-3p | TCCCTCTCCCACCCCTTGC | MIMAT0027685 | hsa-miR-6892-3p | 0 | 0 | 0 | 0 | 0 | 0 | 0 | 0 |
| hsa-miR-6892-5p | GTAAGGGACCGGAGAGTAGG | MIMAT0027684 | hsa-miR-6892-5p | 21 | 22 | 36 | 26 | 21 | 15 | 0 | 27 |
| hsa-miR-6893-3p | CCCTGCTGCCTTCACCTGCC | MIMAT0027687 | hsa-miR-6893-3p | 0 | 0 | 2 | 0 | 0 | 2 | 0 | 0 |
| hsa-miR-6893-5p | AGGCAGGTGTAGGGTGGAGCC | MIMAT0027686 | hsa-miR-6893-5p | 6 | 6 | 7 | 4 | 0 | 0 | 0 | 2 |
| hsa-miR-6894-3p | TTGCCTGCCCTCTTCCTCC | MIMAT0027689 | hsa-miR-6894-3p | 9 | 13 | 23 | 29 | 20 | 16 | 18 | 33 |
| hsa-miR-6894-5p | AAGGAGGATGGAGAGCTGGGCCAGT | MIMAT0027688 | hsa-miR-6894-5p | 14 | 19 | 7 | 21 | 59 | 49 | 73 | 40 |
| hsa-miR-6895-3p | TGTCTCTCGCCCTTGGCCTTAG | MIMAT0027691 | hsa-miR-6895-3p | 9 | 10 | 6 | 1 | 11 | 3 | 12 | 6 |
| hsa-miR-6895-5p | CAGGGCCAGGCACAGAGTA | MIMAT0027690 | hsa-miR-6895-5p | 8 | 18 | 0 | 12 | 6 | 2 | 0 | 7 |
| hsa-miR-7-1-3p | CAACAAATCACAGTCTGCCAT | MIMAT0004553 | hsa-miR-7-1-3p | 42 | 101 | 40 | 58 | 44 | 34 | 51 | 64 |
| hsa-miR-7-2-3p | CAACAAATCCCAGTCTACCTA | MIMAT0004554 | hsa-miR-7-2-3p | 0 | 12 | 0 | 4 | 0 | 2 | 0 | 1 |
| hsa-miR-7-5p | TGGAAGACTAGTGATTTTGTTGTT | MIMAT0000252 | hsa-miR-7-5p | 5188 | 12010 | 5318 | 4640 | 4102 | 3585 | 2233 | 8439 |
| hsa-miR-708-3p | AACTAGACTGTGAGCTTC | MIMAT0004927 | hsa-miR-708-3p | 0 | 4 | 0 | 0 | 0 | 0 | 0 | 2 |
| hsa-miR-708-5p | AAGGAGCTTACAATCTAGCTGGG | MIMAT0004926 | hsa-miR-708-5p | 409 | 327 | 290 | 270 | 280 | 233 | 231 | 233 |
| hsa-miR-7106-3p | TCCCTGAATCCCTGTCCCAG | MIMAT0028110 | hsa-miR-7106-3p | 1 | 0 | 0 | 0 | 0 | 0 | 0 | 0 |
| hsa-miR-7106-5p | TTCTGGGAGGAGGGGATCTTGGG | MIMAT0028109 | hsa-miR-7106-5p | 7 | 0 | 0 | 0 | 6 | 4 | 15 | 4 |
| hsa-miR-7107-3p | TCTGTTCCTTCTCTCTTTTTGGCC | MIMAT0028112 | hsa-miR-7107-3p | 1 | 6 | 4 | 2 | 0 | 0 | 0 | 2 |
| hsa-miR-7107-5p | TCGGCCTGGGGAGGAGGAAGGG | MIMAT0028111 | hsa-miR-7107-5p | 2 | 0 | 0 | 0 | 0 | 0 | 0 | 0 |
| hsa-miR-7108-3p | ACCCGCCCGTCTCCCCAC | MIMAT0028114 | hsa-miR-7108-3p | 0 | 0 | 1 | 0 | 3 | 0 | 0 | 4 |
| hsa-miR-7108-5p | GTGTGGCCGGCAGGCGGG | MIMAT0028113 | hsa-miR-7108-5p | 35 | 42 | 55 | 18 | 30 | 26 | 54 | 48 |
| hsa-miR-7109-3p | CAAGCCTCTCCTGCCCTTCCAGT | MIMAT0028116 | hsa-miR-7109-3p | 6 | 23 | 9 | 15 | 18 | 9 | 0 | 9 |
| hsa-miR-7109-5p | CCTGGGGGGAGGAGACCCTGCT | MIMAT0028115 | hsa-miR-7109-5p | 2 | 2 | 2 | 2 | 7 | 7 | 21 | 9 |
| hsa-miR-7110-3p | TCTCTCTCCCACTTCCCTGCAG | MIMAT0028118 | hsa-miR-7110-3p | 0 | 0 | 0 | 4 | 4 | 0 | 0 | 1 |
| hsa-miR-7110-5p | TGGGGGTGTGGGGAGAGAGAGT | MIMAT0028117 | hsa-miR-7110-5p | 0 | 0 | 0 | 0 | 0 | 0 | 0 | 0 |
| hsa-miR-7111-3p | ATCCTCTCTTCCCTCCTCCCAG | MIMAT0028120 | hsa-miR-7111-3p | 0 | 1 | 1 | 1 | 1 | 1 | 0 | 0 |
| hsa-miR-7111-5p | TGGGGGAGGAAGGACAGGCC | MIMAT0028119 | hsa-miR-7111-5p | 17 | 16 | 10 | 12 | 23 | 5 | 3 | 5 |
| hsa-miR-7112-3p | TGCATCACAGCCTTTGGCCCT | MIMAT0028122 | hsa-miR-7112-3p | 2 | 2 | 9 | 2 | 6 | 3 | 13 | 6 |
| hsa-miR-7112-5p | ACGGGCAGGGCAGTGCAA | MIMAT0028121 | hsa-miR-7112-5p | 0 | 0 | 0 | 0 | 0 | 0 | 0 | 0 |
| hsa-miR-7113-3p | ATGGCCTCCCTGCCCGCCTCTCT | MIMAT0028124 | hsa-miR-7113-3p | 0 | 0 | 0 | 0 | 0 | 2 | 0 | 0 |
| hsa-miR-7113-5p | TCCAGGGAGACAGTGTGTGAGGC | MIMAT0028123 | hsa-miR-7113-5p | 8 | 14 | 5 | 17 | 33 | 16 | 33 | 15 |
| hsa-miR-7114-3p | TGACCCACCCCTCTCCACC | MIMAT0028126 | hsa-miR-7114-3p | 3 | 2 | 4 | 3 | 1 | 2 | 0 | 3 |
| hsa-miR-7114-5p | TGTGGAGTGGGGTGCCTGTCC | MIMAT0028125 | hsa-miR-7114-5p | 24 | 40 | 29 | 31 | 84 | 82 | 185 | 92 |
| hsa-miR-7155-3p | ATGGCCCAAGACCTCAGACC | MIMAT0028221 | hsa-miR-7155-3p | 1 | 0 | 0 | 0 | 0 | 0 | 0 | 0 |
| hsa-miR-7155-5p | TCTGGGGTCTTGGGCCATCT | MIMAT0028220 | hsa-miR-7155-5p | 0 | 0 | 0 | 7 | 2 | 0 | 0 | 0 |
| hsa-miR-7161-3p | ATCTTTGACGCTGGCAGTCT | MIMAT0028233 | hsa-miR-7161-3p | 0 | 0 | 0 | 0 | 0 | 0 | 0 | 0 |
| hsa-miR-744-3p | CTGTTGCCACTAACCTCAACC | MIMAT0004946 | hsa-miR-744-3p | 93 | 148 | 121 | 137 | 162 | 112 | 170 | 102 |
| hsa-miR-744-5p | TGCGGGGCTAGGGCTAACAGC | MIMAT0004945 | hsa-miR-744-5p | 18098 | 27937 | 18863 | 27179 | 37084 | 31598 | 42213 | 33149 |
| hsa-miR-758-3p | TTTGTGACCTGGTCCACTAACC | MIMAT0003879 | hsa-miR-758-3p | 221 | 339 | 238 | 262 | 400 | 363 | 888 | 386 |
| hsa-miR-758-5p | ATGGTTGACCAGAGAGCACACG | MIMAT0022929 | hsa-miR-758-5p | 52 | 43 | 29 | 38 | 42 | 38 | 41 | 48 |
| hsa-miR-760 | CGGCTCTGGGTCTGTGGGGAGT | MIMAT0004957 | hsa-miR-760 | 1256 | 1799 | 1184 | 1109 | 1885 | 2176 | 4263 | 2300 |
| hsa-miR-761 | AGGAGCAGCTGGGTGAAA | MIMAT0010364 | hsa-miR-761 | 0 | 0 | 2 | 0 | 0 | 0 | 0 | 0 |
| hsa-miR-762 | GGGGCCGGGGCCGAGGCCGC | MIMAT0010313 | hsa-miR-762 | 2 | 2 | 7 | 8 | 6 | 5 | 18 | 5 |
| hsa-miR-7641 | TCTCGGAAGCTAAGCAGGG | NA | hsa-miR-7641 | 1050 | 757 | 951 | 785 | 594 | 556 | 885 | 675 |
| hsa-miR-765 | TGGAGGAGAAGGAAGGTGATG | MIMAT0003945 | hsa-miR-765 | 1 | 7 | 5 | 14 | 14 | 4 | 0 | 8 |
| hsa-miR-766-3p | ACTCCAGCCCCACAGCCTCAGC | MIMAT0003888 | hsa-miR-766-3p | 648 | 854 | 415 | 548 | 682 | 749 | 253 | 631 |
| hsa-miR-766-5p | AGGAGGAATTGGTGCTGGTCTT | MIMAT0022714 | hsa-miR-766-5p | 112 | 366 | 112 | 186 | 389 | 411 | 631 | 400 |
| hsa-miR-767-3p | TCTGCTCATACCCCATGGTTT | MIMAT0003883 | hsa-miR-767-3p | 0 | 0 | 0 | 0 | 5 | 5 | 19 | 7 |
| hsa-miR-767-5p | TGCACCATGGTTGTCTGAGCATGC | MIMAT0003882 | hsa-miR-767-5p | 1 | 0 | 0 | 0 | 9 | 5 | 0 | 1 |
| hsa-miR-769-3p | TGGGATCTCCGGGGTCTTGGTT | MIMAT0003887 | hsa-miR-769-3p | 2197 | 3800 | 2409 | 2630 | 7569 | 7265 | 18353 | 7362 |
| hsa-miR-769-5p | TGAGACCTCTGGGTTCTGAGCT | MIMAT0003886 | hsa-miR-769-5p | 162 | 274 | 158 | 248 | 637 | 631 | 1524 | 573 |
| hsa-miR-770-5p | TCCAGTACCACGTGTCAGGGCCACT | MIMAT0003948 | hsa-miR-770-5p | 512 | 1039 | 589 | 509 | 715 | 606 | 1128 | 841 |
| hsa-miR-7702 | CTTAGACTGCCAGACTCCCTG | MIMAT0030017 | hsa-miR-7702 | 4 | 1 | 0 | 0 | 3 | 4 | 11 | 4 |
| hsa-miR-7703 | TGCACTCTGGCCTTCTCCCAG | MIMAT0030018 | hsa-miR-7703 | 0 | 0 | 2 | 1 | 1 | 0 | 0 | 0 |
| hsa-miR-7704 | CGGGGTCGGCGGCGACGTC | MIMAT0030019 | hsa-miR-7704 | 11 | 26 | 12 | 87 | 40 | 40 | 110 | 54 |
| hsa-miR-7705 | AATAGCTCAGAATGTCAGTTC | MIMAT0030020 | hsa-miR-7705 | 0 | 0 | 0 | 0 | 0 | 0 | 0 | 0 |
| hsa-miR-7706 | TGAAGCGCCTGTGCTCTGCCG | MIMAT0030021 | hsa-miR-7706 | 1324 | 1387 | 1257 | 1133 | 1602 | 1575 | 3234 | 1719 |
| hsa-miR-7844-5p | AAAACTAGGACTGTGTGGTGT | MIMAT0030419 | hsa-miR-7844-5p | 0 | 0 | 0 | 0 | 0 | 0 | 0 | 0 |
| hsa-miR-7845-5p | AAGGGACAGGGAGGGTCGTGGCG | MIMAT0030420 | hsa-miR-7845-5p | 60 | 72 | 65 | 65 | 115 | 117 | 206 | 89 |
| hsa-miR-7846-3p | CAGCGGAGCCUGGAGAGAAGG | MIMAT0030421 | hsa-miR-7846-3p | 0 | 0 | 0 | 0 | 0 | 0 | 0 | 0 |
| hsa-miR-7847-3p | GGAGGAGGAGGAGGAGGC | MIMAT0030422 | hsa-miR-7847-3p | 1 | 1 | 5 | 1 | 0 | 2 | 0 | 0 |
| hsa-miR-7849-3p | TTGATCTTGGGCCTGATGT | MIMAT0030424 | hsa-miR-7849-3p | 0 | 0 | 0 | 0 | 0 | 0 | 0 | 0 |
| hsa-miR-7850-5p | GTTTGGACATAGTGTGGC | MIMAT0030425 | hsa-miR-7850-5p | 0 | 0 | 0 | 1 | 4 | 1 | 0 | 0 |
| hsa-miR-7851-3p | TACCTGGGAGACTGAGGTTGGAT | MIMAT0030426 | hsa-miR-7851-3p | 0 | 2 | 0 | 3 | 0 | 0 | 0 | 0 |
| hsa-miR-7854-3p | TGAGGTGACCGCAGATGGGAAGG | MIMAT0030429 | hsa-miR-7854-3p | 185 | 133 | 153 | 102 | 105 | 98 | 122 | 139 |
| hsa-miR-7856-5p | TTTTAAGGACACTGAGGGATC | MIMAT0030431 | hsa-miR-7856-5p | 0 | 4 | 0 | 0 | 0 | 0 | 0 | 0 |
| hsa-miR-7974 | AGGCTGTGATGCTCTCCTGAGCC | MIMAT0031177 | hsa-miR-7974 | 2 | 0 | 2 | 2 | 0 | 2 | 0 | 0 |
| hsa-miR-7976 | TGCCCTGAGACTTTTGCTCTA | MIMAT0031179 | hsa-miR-7976 | 0 | 0 | 0 | 0 | 1 | 0 | 0 | 0 |
| hsa-miR-7977 | TTCCCGGCCAACGCACCA | MIMAT0031180 | hsa-miR-7977 | 2568 | 2926 | 6915 | 2997 | 1485 | 650 | 661 | 1118 |
| hsa-miR-802 | TCAGTAACAAAGATTCATCCTTG | MIMAT0004185 | hsa-miR-802 | 0 | 0 | 0 | 0 | 0 | 0 | 0 | 4 |
| hsa-miR-8072 | ATGGCGGCGGGGAGGTAGGC | MIMAT0030999 | hsa-miR-8072 | 4 | 8 | 3 | 4 | 28 | 29 | 57 | 41 |
| hsa-miR-8085 | GGGAGAGAGGACTGTGAGGCATG | MIMAT0031012 | hsa-miR-8085 | 0 | 0 | 0 | 0 | 0 | 0 | 0 | 0 |
| hsa-miR-8089 | GGGAGAGGGGATTGGGGC | MIMAT0031016 | hsa-miR-8089 | 0 | 0 | 0 | 0 | 0 | 0 | 0 | 0 |
| hsa-miR-8485 | CACACACACACACACACACAC | MIMAT0033692 | hsa-miR-8485 | 0 | 1 | 0 | 0 | 0 | 0 | 0 | 0 |
| hsa-miR-874-3p | CTGCCCTGGCCCGAGGGACCGAC | MIMAT0004911 | hsa-miR-874-3p | 490 | 831 | 477 | 893 | 2307 | 2400 | 3700 | 1763 |
| hsa-miR-874-5p | CGGCCCCACGCACCAGGGTA | MIMAT0026718 | hsa-miR-874-5p | 67 | 23 | 39 | 26 | 24 | 21 | 0 | 12 |
| hsa-miR-877-3p | TCCTCTTCTCCCTCCTCCCAG | MIMAT0004950 | hsa-miR-877-3p | 16 | 15 | 32 | 16 | 18 | 12 | 37 | 13 |
| hsa-miR-877-5p | GTAGAGGAGATGGCGCAGGG | MIMAT0004949 | hsa-miR-877-5p | 17664 | 9948 | 14666 | 7059 | 5003 | 4453 | 3882 | 4624 |
| hsa-miR-885-3p | AGGCAGCGGGGTGTAGTGGATT | MIMAT0004948 | hsa-miR-885-3p | 0 | 3 | 0 | 0 | 0 | 4 | 0 | 0 |
| hsa-miR-885-5p | TCCATTACACTACCCTGCCTCT | MIMAT0004947 | hsa-miR-885-5p | 0 | 0 | 0 | 3 | 0 | 0 | 0 | 0 |
| hsa-miR-887-3p | GTGAACGGGCGCCATCCCGAGGCTT | MIMAT0004951 | hsa-miR-887-3p | 1474 | 2454 | 1310 | 1737 | 3055 | 2991 | 5339 | 2836 |
| hsa-miR-887-5p | TCCTTGGGAGCCCTGTTAGAC | MIMAT0026720 | hsa-miR-887-5p | 43 | 53 | 46 | 49 | 52 | 43 | 31 | 32 |
| hsa-miR-888-5p | TACTCAAAAAGCTGTCAGTC | MIMAT0004916 | hsa-miR-888-5p | 0 | 5 | 3 | 0 | 0 | 0 | 0 | 0 |
| hsa-miR-889-3p | TTAATATCGGACAACCATTGT | MIMAT0004921 | hsa-miR-889-3p | 32 | 38 | 33 | 28 | 42 | 21 | 5 | 54 |
| hsa-miR-889-5p | AATGGCTGTCCGTAGTATGGTC | MIMAT0026719 | hsa-miR-889-5p | 144 | 197 | 200 | 158 | 210 | 195 | 346 | 211 |
| hsa-miR-891a-5p | TGCAACGAACCTGAGCCACTG | MIMAT0004902 | hsa-miR-891a-5p | 7 | 16 | 11 | 5 | 17 | 0 | 0 | 7 |
| hsa-miR-892a | CACUGUGUCCUUUCUGCGUAG | MIMAT0004907 | hsa-miR-892a | 0 | 0 | 0 | 0 | 0 | 0 | 0 | 0 |
| hsa-miR-9-3p | ATAAAGCTAGATAACCGAAAGT | MIMAT0000442 | hsa-miR-9-3p | 16 | 9 | 23 | 6 | 0 | 0 | 0 | 7 |
| hsa-miR-9-5p | TCTTTGGTTATCTAGCTGTATG | MIMAT0000441 | hsa-miR-9-5p | 208 | 158 | 307 | 118 | 38 | 35 | 56 | 33 |
| hsa-miR-92a-1-5p | AGGTTGGGATCGGTTGCAATGC | MIMAT0004507 | hsa-miR-92a-1-5p | 141 | 264 | 167 | 185 | 203 | 198 | 337 | 264 |
| hsa-miR-92a-2-5p | GGGTGGGGATTTGTTGCATTAC | MIMAT0004508 | hsa-miR-92a-2-5p | 0 | 0 | 0 | 0 | 0 | 0 | 0 | 0 |
| hsa-miR-92a-3p | TATTGCACTTGTCCCGGCCTGT | MIMAT0000092 | hsa-miR-92a-3p | 320171 | 465161 | 301470 | 310705 | 290903 | 261849 | 207309 | 312881 |
| hsa-miR-92b-3p | TATTGCACTCGTCCCGGCCTCC | MIMAT0003218 | hsa-miR-92b-3p | 134246 | 199220 | 105210 | 96070 | 90998 | 87482 | 71384 | 139172 |
| hsa-miR-92b-5p | AGGGACGGGACGCGGTGCAGTGT | MIMAT0004792 | hsa-miR-92b-5p | 6694 | 11084 | 9229 | 8112 | 11851 | 10645 | 15029 | 15659 |
| hsa-miR-93-3p | ACTGCTGAGCTAGCACTTCCCG | MIMAT0004509 | hsa-miR-93-3p | 13 | 34 | 18 | 17 | 37 | 40 | 72 | 36 |
| hsa-miR-93-5p | CAAAGTGCTGTTCGTGCAGGT | MIMAT0000093 | hsa-miR-93-5p | 9098 | 9672 | 10107 | 9593 | 10191 | 9343 | 9549 | 9337 |
| hsa-miR-933 | TGTGCGCAGGGAGACCTC | MIMAT0004976 | hsa-miR-933 | 0 | 0 | 2 | 0 | 0 | 0 | 0 | 0 |
| hsa-miR-935 | CAGTTACCGCTTCCGCTACCGC | MIMAT0004978 | hsa-miR-935 | 47193 | 86120 | 37468 | 62548 | 72793 | 76508 | 53931 | 60548 |
| hsa-miR-936 | ACAGTAGAGGGAGGAATCGC | MIMAT0004979 | hsa-miR-936 | 0 | 0 | 0 | 0 | 0 | 0 | 0 | 0 |
| hsa-miR-937-3p | ATCCGCGCTCTGACTCTCTGCC | MIMAT0004980 | hsa-miR-937-3p | 321 | 716 | 371 | 351 | 608 | 571 | 1124 | 706 |
| hsa-miR-937-5p | GTGAGTCAGGGTGGGGCTGGCC | MIMAT0022938 | hsa-miR-937-5p | 17 | 16 | 22 | 23 | 41 | 37 | 95 | 49 |
| hsa-miR-939-3p | CTGACCCTGGGCCTCTGCTCCCC | MIMAT0022939 | hsa-miR-939-3p | 0 | 0 | 0 | 2 | 3 | 0 | 0 | 3 |
| hsa-miR-939-5p | CTGGGGAGCTGAGGCTCTGGGGT | MIMAT0004982 | hsa-miR-939-5p | 3 | 8 | 6 | 4 | 6 | 13 | 12 | 5 |
| hsa-miR-940 | AAGGCAGGGCCCCCGCTCCCC | MIMAT0004983 | hsa-miR-940 | 1518 | 2494 | 1218 | 1729 | 2391 | 2163 | 2449 | 2224 |
| hsa-miR-941 | CACCCGGCTGTGTGCACATGTGC | MIMAT0004984 | hsa-miR-941 | 1419 | 2616 | 1638 | 1788 | 2129 | 1760 | 2742 | 2180 |
| hsa-miR-942-3p | CACATGGCCGAAACAGAGAAGT | MIMAT0026734 | hsa-miR-942-3p | 71 | 73 | 64 | 82 | 59 | 52 | 27 | 88 |
| hsa-miR-942-5p | TCTTCTCTGTTTTGGCCATGTG | MIMAT0004985 | hsa-miR-942-5p | 1203 | 968 | 1104 | 643 | 407 | 384 | 486 | 652 |
| hsa-miR-943 | CCTGACTGTTGCCGTCCTCCAGT | MIMAT0004986 | hsa-miR-943 | 16 | 10 | 6 | 9 | 28 | 22 | 56 | 22 |
| hsa-miR-95-3p | TTCAACGGGTATTTATTGAGC | MIMAT0000094 | hsa-miR-95-3p | 66 | 108 | 45 | 82 | 78 | 56 | 36 | 57 |
| hsa-miR-96-5p | TTTGGCACTAGCACATTTTTGC | MIMAT0000095 | hsa-miR-96-5p | 178 | 68 | 218 | 78 | 26 | 36 | 70 | 25 |
| hsa-miR-98-3p | CTATACAACTTACTACTTTCC | MIMAT0022842 | hsa-miR-98-3p | 0 | 0 | 0 | 0 | 0 | 0 | 0 | 0 |
| hsa-miR-98-5p | TGAGGTAGTAAGTTGTATTGTT | MIMAT0000096 | hsa-miR-98-5p | 10196 | 15601 | 9430 | 12863 | 10370 | 8328 | 2448 | 9708 |
| hsa-miR-99a-3p | CAAGCTCGCTTCTATGGGTCTGT | MIMAT0004511 | hsa-miR-99a-3p | 40 | 80 | 36 | 82 | 121 | 117 | 250 | 113 |
| hsa-miR-99a-5p | AACCCGTAGATCCGATCTTGTG | MIMAT0000097 | hsa-miR-99a-5p | 7845 | 10770 | 8209 | 10455 | 12046 | 10456 | 17169 | 10469 |
| hsa-miR-99b-3p | CAAGCTCGTGTCTGTGGGTCCG | MIMAT0004678 | hsa-miR-99b-3p | 5654 | 8643 | 6210 | 6349 | 9045 | 9980 | 16673 | 9818 |
| hsa-miR-99b-5p | CACCCGTAGAACCGACCTTGCG | MIMAT0000689 | hsa-miR-99b-5p | 120659 | 167769 | 129353 | 147053 | 115215 | 90831 | 110567 | 133939 |

**Supplemental Table 3**: Overlap between 50 miRNAs analysed in the microscopy-based screen and the deregulated miRNAs after subjecting cells to the conditions of incubation with low serum concentrations:

| **upregulated** | **downregulated** |
| --- | --- |
| hsa-miR-143-3p | hsa-miR-124-3p |
| hsa-miR-185-5p | hsa-miR-31-3p |
| hsa-miR-423-3p | hsa-miR-23a-3p |
| hsa-miR-193b-5p | hsa-miR-199a-3p |
| hsa-miR-30c-2-3p | hsa-miR-92a-3p |
| hsa-miR-22-3p | hsa-miR-27b-3p |
| hsa-miR-145-5p |  |
| hsa-miR-138-5p |  |
| hsa-miR-141-3p |  |
| hsa-miR-132-3p |  |
| hsa-miR-150-5p |  |

**Supplemental Table 4:** List of the six-miRNA-related genes.

| **Gene-miR-132 targets** | **Gene-miR-138 targets** | **Gene-miR-141 targets** | **Gene-miR-145 targets** | **Gene-miR-150 targets** | **Gene-miR-22 targets** |
| --- | --- | --- | --- | --- | --- |
| KCNMA1 | TCF4 | MAPK10 | KCNMA1 | CACNA1C | BDNF |
| ESRRG | KCNMA1 | ST3GAL3 | IKZF3 | VEGFA | TPM3 |
| BCL2L11 | OPRM1 | FOXP1 | MUC1 | SLC1A2 | TP53 |
| NFIB | LIMCH1 | BCL2L13 | DYRK1A | CARHSP1 | EPB41L2 |
| CASP7 | OPALIN | ZBTB20 | DLG4 | MAP4K4 | SERBP1 |
| ELAVL4 | PAX5 | MEIS2 | MAP4K4 | PIK3R1 | LHX6 |
| NFATC2 | DMKN | ELAVL4 | SRSF10 | TP53 | YWHAZ |
| RUNX1T1 | RNF24 | GSG1 | NDRG2 | ZBTB20 | PAX6 |
| ORC4 | EDC3 | LDLRAD4 | CTNNA2 | CFLAR | TPD52L2 |
| MEF2A | TMEM182 | PTPRD | CACNB2 | PTPA | FOXP1 |
| CALU | CNOT8 | MYT1L | NFIB | PAX5 | KLF7 |
| ZBTB20 | NKAPD1 | RNF38 | SMAD3 | KIAA0930 | CUL3 |
| STX16 | GPATCH2L | MIER1 | SLC1A2 | C19orf12 | TRIM13 |
| TMEM51 | CBFA2T2 | ABL2 | RIMS1 | BTBD9 | NPNT |
| 44082 | ADGRL3 | NTNG1 | JADE1 | NME6 | APBB2 |
| MEIS2 | TNRC6B | AKAP2 | CADM1 | TPM3 | ACVR1C |
| PPM1L | RBBP4 | NSD2 | POGZ | AGAP1 | TACC1 |
| ETV1 | HPCAL4 | YAP1 | RAB3IP | GPR161 | PPARGC1B |
| SSR3 | SPECC1 | SORBS2 | EIF4E3 | DYRK1A | FMN1 |
| PGPEP1 | ADCYAP1R1 | EIF4E | SET | A1CF | IRF5 |
| AFF2 | SCAI | ARNTL | NFATC1 | CLEC2D | PTGS1 |
| FMN1 | RIMS2 | AFF2 | ALG9 | CTNNB1 | MTMR2 |
| RBFOX1 | ZNF148 | OSBPL8 | MTR | LDLR | ZNF706 |
| SLC8A1 | PPARD | TBC1D5 | ETS1 | KIF1A | IKZF4 |
| TJAP1 | RABL2B | ATF7IP | RREB1 | OCIAD1 | NPAS3 |
| MELK | PACSIN2 | MBNL3 | GRB10 | GPATCH2L | IL17RD |
| MAPT | 43891 | NEK6 | SLC25A25 | SEMA4F | RIMS4 |
| SRSF10 | LGALS12 | NFASC | SLC24A4 | PAK3 | BMPR1B |
| NR2C2 | IGF2BP2 | APBB2 | ELAVL4 | CREB5 | LRRC20 |
| ACVR1 | EYA1 | NFIA | MAPK8 | ARFIP2 | ICA1L |
| MAP3K3 | SLC38A1 | TBL1XR1 | CCDC120 | CMKLR1 | RPS6KC1 |
| SOX5 | NFIA | QKI | DLGAP1 | IRAK1 | TRIM66 |
| SAP30L | LYPD5 | RIMS1 | FXR1 | SH3GLB1 | USP47 |
| GAPVD1 | STRA6 | NPAS3 | ERG | HDAC8 | PPP1R9A |
| SCN1A | ZBTB20 | BBX | PPP1R9A | GIGYF2 | SFMBT2 |
| CSDE1 | PPM1L | PPP2R5E | CAMK2D | POU2F1 | SLC30A8 |
| PTPRD | PPARGC1B | NSL1 | ZBTB20 | SEMA6D | PTP4A3 |
| CD164 | ARPIN | PALM2-AKAP2 | EPB41L5 | ZHX3 | SMIM12 |
| PIK3R3 | GAS7 | CARD8 | CD44 | CAMK2G | GUCD1 |
| EMSY | KIAA0930 | SLC11A2 | MSI2 | ENSA | PACRGL |
| SLC26A7 | TMOD2 | EMSY | GDPD5 | PRRT2 | NUSAP1 |
| SAMD12 | RAB3GAP1 | CALU | PCBP2 | EFCAB11 | RUBCN |
| CSNK1G3 | TBL1X | VANGL1 | MAP3K7CL | IRAK4 | ESR1 |
| STIM2 | CD109 | PEX5 | RCAN1 | AMOTL2 | MAX |
| GAB1 | LETM2 | LYPLA1 | ABL2 | MRVI1 | KCNK10 |
| OPRM1 | LAYN | OSBPL6 | MYRF | CYLD | STAG2 |
| ZDHHC23 | GPRIN3 | ST3GAL5 | NEDD9 | EPHB2 | SOCS2 |
| C3orf14 | SCN3B | NR2C2 | RTKN | FOPNL | MON2 |
| SLC10A7 | ZKSCAN3 | STX16 | TPM3 | RNF165 | KAT7 |
| KCNN3 | GPR85 | CLDND1 | SLC8A3 | RAB3IP | CPEB1 |
| BRCA1 | TOM1L2 | SGCD | ZC2HC1C | NFASC | TCF7 |
| DYNC1LI2 | FAM219B | ALKAL2 | UBE2W | EIF4E | PPM1L |
| QKI | OSBP2 | SOX5 | FAM107B | ETF1 | PSEN1 |
| JPT1 | NNAT | NUDT13 | KCNN3 | SERF2 | TLK2 |
| RAB5B | LCOR | NDUFA5 | ACVR1B | TTPAL | CDCA7L |
| CREB5 | ARHGEF3 | MPRIP | ZRANB3 | CBX5 | MAPK10 |
| EHF | RNF38 | PIP5K1A | ARHGAP26 | TMEM92 | USP44 |
| DGKH | ANK1 | HNRNPR | KCNK10 | DCTN5 | ATXN1 |
| KAT7 | ZFAT | CAMSAP1 | TRAF6 | DPYSL5 | CIDEC |
| PIK3R1 | KANK1 | ELAVL2 | PEG10 | ARRB2 | EYA3 |
| RNF24 | SEZ6L2 | NR3C1 | TRIM13 | SAP30L | RNF150 |
| CBFA2T2 | UNC5D | AGFG1 | NFE2L1 | ING4 | ETV1 |
| ZNF280D | PPIP5K1 | MARK1 | CLCN5 | TNS1 | SESN1 |
| TPGS2 | NSMF | DAPK1 | STK25 | CNOT9 | ARPC5 |
| FXR1 | FOXP4 | SNX18 | EYA3 | PLEKHA1 | EDC3 |
| AMD1 | MRAS | IGF2 | SSH2 | AKAP2 | C5orf24 |
| PHF20L1 | ENOX2 | CDK6 | WDR17 | DKK3 | MTHFR |
| SMAD2 | MYO1C | GPM6B | SLC8A1 | PEG10 | VAPB |
| NIPSNAP2 | PDPN | SYNCRIP | TCF4 | SLC20A2 | KCTD10 |
| VMP1 | CCT5 | PHYHIPL | ZNF329 | RNF32 | USP37 |
| PRDM15 | NEBL | BACE1 | SRGAP1 | GRAMD1B | MSL2 |
| SOX6 | CAMTA2 | FOXJ3 | AP1G1 | CHD3 | DNAJB5 |
| KLF7 | PDLIM5 | ANP32E | CDK6 | SLC35A2 | GLIPR2 |
| KCNK2 | CBX5 | RANBP6 | ORC4 | PPP2R2A | VPS53 |
| ZBTB18 | MON2 | PEAK1 | PARP8 | FAM168A | SMG7 |
| ABR | ELMOD3 | PITPNB | AGFG1 | IST1 | GRM5 |
| AEBP2 | EPB41 | TNPO1 | ELMO1 | PEA15 | LRCH1 |
| SMAD5 | SLC7A2 | SLC23A2 | DERL2 | POLDIP3 | RIC8B |
| PTBP2 | PSD3 | STXBP5 | FLT1 | SLC8A1 | KDM6B |
| AGO1 | CMTM3 | NRXN1 | TPT1 | CAMSAP1 | RAPGEFL1 |
| FOXP2 | ELF2 | CCNT2 | SNX27 | ZEB1 | SCYL3 |
| NOVA1 | ZNF827 | SLC39A9 | RFX3 | OSBPL9 | FBXW7 |
| CFL2 | MYD88 | PSEN1 | PAPD4 | ANKRD65 | TRPM7 |
| HIC2 | CDK19 | RAPGEF5 | TBC1D14 | CALCOCO2 | DPP10 |
| WTAP | IGF2BP1 | WDR44 | SIRPA | ZNF346 | MAPK14 |
| TMEM106B | DNAJB6 | SOX6 | CASP10 | TLK2 | NFYA |
| BBX | FOSL1 | SUV39H2 | NTRK2 | AKT3 | PTPN1 |
| L3MBTL3 | PHKG2 | MAP3K7 | GDNF | PTCH1 | HMGB1 |
| PRICKLE2 | LSM14A | ARRB1 | PTBP3 | ARSA | ZNF662 |
| ZEB2 | DESI2 | IL6R | ARHGAP12 | CACNB3 | NHP2 |
| SMN1 | NXN | AGPS | PCYT1B | GOLGA7 | SATB2 |
| NCS1 | MLXIP | KCNC2 | ZNF398 | N4BP2L1 | CELF1 |
| ECT2 | LYPLA1 | ZKSCAN1 | KCNK9 | ZNF793 | ARFIP2 |
| RAB18 | ARRB1 | ZDHHC3 | LNPK | NFIC | PHF23 |
| OLFML2A | AHCYL2 | RAD54L2 | NTNG1 | SMIM14 | CTSC |
| SV2B | MANEAL | SNX27 | RSPO1 | FAM76A | MTF2 |
| GPATCH2L | MIEF2 | DCUN1D4 | CALU | CD84 | IL1RL1 |
| ZNF248 | CNOT6L | XPR1 | CNDP2 | CYTH1 | OPRL1 |
| KIAA1549 | NFIX | PYGO1 | WDR20 | RNF146 | GPR161 |
| TMED5 | GGCX | PTPN2 | AGPAT3 | EPB41L5 | RBFOX2 |
| MEPCE | ZNF74 | RNMT | AR | ZFAND3 | CBX6 |
| TCEB1 | DHDDS | WNT5A | HDLBP | TGIF2 | KAT5 |
| DGCR8 | THRB | ST3GAL6 | CA12 | MANBAL | PHACTR2 |
| RLIM | ST3GAL6 | CLOCK | LSAMP | HPSE2 | BTBD9 |
| TRIP12 | VAPB | PRELID2 | PCBP4 | CPSF7 | FAS |
| SSH2 | C16orf87 | ATXN7L1 | STX16 | HILPDA | GNB4 |
| TSHZ2 | MEAF6 | RAP2C | TSPAN14 | NR2F2 | LIN7C |
| BTRC | RGMA | UBAP1 | PHACTR2 | PXMP4 | RAB5B |
| SIRT1 | CNOT9 | CUL3 | DUSP6 | CREB1 | TET2 |
| ZNF652 | DHRS7 | PPM1L | CTNNBIP1 | MBTD1 | PDIK1L |
| SPAST | BPNT1 | YPEL5 | SMAD2 | PRKAR1A | ARHGEF26 |
| ELOC | ZNF510 | ANKFY1 | SOCS7 | RAD23B | PPARA |
| NAP1L1 | C12orf65 | MAGI2 | MDM2 | MLXIP | PRELID2 |
| KLHL11 | EGFR | TMEM130 | TSPAN6 | SUV39H2 | HNRNPA3 |
| USP8 | KIF21B | RASSF8 | SP1 | ATP8A2 | RFXANK |
| RAB6B | BVES | ZNF281 | UNC5D | IGF2BP1 | VEZF1 |
| SLC30A6 | SPATA13 | PLXNA4 | FBXO28 | FOXO4 | ANGEL2 |
| ETNK1 | TRIM13 | ATP8A2 | ZBTB33 | DCAF6 | STK26 |
| SRGAP1 | ZBTB8A | KATNAL1 | ATP1B4 | RC3H1 | TUB |
| PAIP2 | ST3GAL3 | KIF3A | ABR | PRICKLE2 | EPC1 |
| SREBF1 | CINP | SLC16A7 | ANKFY1 | ENTPD1 | PNISR |
| DCUN1D4 | MPRIP | ATXN1 | PHRF1 | ATP2B1 | TP53INP1 |
| COLQ | ZDHHC3 | RBM24 | DENND5B | BTN3A2 | BCL9L |
| SS18 | HIPK1 | MYH10 | ANO6 | ASB8 | ARHGEF12 |
| ATXN7 | SLC24A2 | MAP2K7 | NFIA | PSMC1 | STYX |
| UBE2W | RASL12 | C21orf91 | DOCK9 | DSTYK | RSBN1 |
| FBN3 | USP39 | FAM168A | FREM1 | CDK2 | KDM3A |
| STAU1 | AFF3 | CACNA1E | HIC2 | LIPG | COPS7B |
| CCSER2 | ACTR8 | GAB1 | RLIM | MBD1 | MED28 |
| CALN1 | BAZ2B | ARL4A | MTX3 | RAPGEF6 | CIITA |
| DIAPH1 | MS4A2 | CYP26B1 | AP2B1 | TLR10 | POGK |
| KCNA6 | RHOC | STK3 | LASP1 | TRIM65 | TNRC6B |
| KLRG1 | GNAI2 | MCL1 | UHMK1 | GRIN2B | WWC2 |
| UNC13A | ZNF607 | CANX | CLCN3 | SV2B | RMDN3 |
| CD300LF | DEK | HLF | CDKN1A | ABI2 | ASB6 |
| ANGEL2 | MAP2K7 | MAP3K3 | TIRAP | EIF2A | GPR107 |
| TMEM164 | JMJD8 | TRPC5OS | POU5F1 | FOSL2 | PCGF5 |
| CUL4B | SNCB | PLEKHA7 | NR1D2 | PGPEP1 | HDAC6 |
| PRR5L | SLC22A23 | CCDC186 | CSRNP3 | TEX2 | CDKN1A |
| INPP5K | SLC39A9 | TGFBR1 | REL | LGALS8 | INSIG1 |
| 43896 | NCBP3 | FOSB | ABI2 | NTRK2 | HIF1A |
| DKK3 | SIGLECL1 | ADAM22 | PSEN1 | ITIH5 | DDX6 |
| FBXO21 | SRRM4 | STEAP2 | FAM104A | REPS2 | ARHGAP26 |
| NUCKS1 | PEG10 | ZNF248 | KIAA1549L | AMN1 | CASP10 |
| TMEM59 | CREB3L2 | TMEM59 | CRK | CCNT1 | SLC24A4 |
| NFIA | ZNF282 | PTP4A1 | SMTNL2 | CERS5 | TRIM67 |
| MIER1 | ZBTB44 | CNR1 | CDK14 | ARRB1 | DISC1 |
| FBXO9 | SLC4A8 | CREB3L2 | RAB18 | DGKI | TMSB15B |
| SESN3 | RIMS3 | AFF1 | PYROXD1 | TP53INP1 | NXT2 |
| ZWINT | CLOCK | ITGA6 | LMBR1L | AZIN1 | GFRA1 |
| NDUFAF6 | RIBC1 | ZMYM2 | PDE4B | PDLIM5 | CCDC152 |
| EMCN | RPS6KA1 | NUMBL | ADGRF1 | PTBP2 | BTBD10 |
| TET2 | SDC3 | RAPGEF6 | PGM3 | AGO1 | CALCR |
| MAP3K20 | TNFSF4 | FAM118B | RAB11FIP1 | EZH1 | WSCD1 |
| SETDB2 | PSMF1 | CBX5 | HMGB3 | SAMD14 | RAPGEF3 |
| FAM19A2 | NSFL1C | DIS3 | NCALD | ZKSCAN1 | PDZD4 |
| USP15 | MAPKBP1 | CALML4 | DYRK1B | ST7L | CSRNP3 |
| MTF1 | EIF3J | AKTIP | ZBTB8A | PARP16 | C12orf66 |
| MLLT3 | USP49 | UBXN8 | AKIRIN1 | ZNF182 | PRRC1 |
| CXorf36 | CCND1 | RBFOX1 | RBM23 | GNL3L | JADE2 |
| HOMER1 | CLEC1A | PPP1R21 | CLOCK | CSF3 | GPSM2 |
| LRRFIP1 | NCOA1 | SMAD2 | TBC1D2B | PSD3 | KMT2A |
| ZNF207 | CCNE1 | CADM2 | PDXK | MR1 | MAP3K12 |
| PSD3 | TRIM67 | CGGBP1 | FUS | MAVS | AK2 |
| UHMK1 | SUFU | HTR7 | CLN8 | TMPPE | EZH1 |
| GUCY1A1 | HSDL2 | PCGF5 | MCPH1 | TMCC1 | EXOC5 |
| FAM184A | KIAA1958 | IDE | TOMM40L | VTA1 | SEMA4F |
| TRDN | TMEM143 | ZEB2 | RNF24 | ST3GAL6 | TMEM50B |
| IL17RD | C12orf43 | YWHAG | PDCD2 | STEAP3 | EDA |
| MITF | ANO6 | E2F3 | TMOD2 | FAM168B | INO80E |
| RBM12 | FCMR | CDC25A | USP3 | SLC39A7 | CCDC149 |
| CTCFL | TRAF5 | PHLPP2 | ACTR2 | R3HCC1L | WDTC1 |
| DCUN1D1 | KLHDC10 | OGT | RPP14 | DTX4 | SLFN5 |
| CRTC1 | SAMD12 | LPP | ST3GAL6 | TKFC | NFAT5 |
| GDF5 | CSRNP3 | KLF12 | GPD2 | TMEM44 | USP8 |
| CRK | C19orf25 | IGF1R | TSHZ2 | EGR2 | KALRN |
| CTDSPL2 | EML1 | PPARA | CC2D2B | ADIPOR2 | CD302 |
| GNL3L | IP6K1 | C18orf25 | LDB1 | GOSR1 | MFN1 |
| ZNF236 | TESMIN | ERO1A | DCUN1D1 | MUC4 | MAP3K13 |
| CYP20A1 | ELOF1 | HIPK2 | PODXL | SPIB | NCOA1 |
| AGO2 | STAMBP | TNS1 | ADD3 | ELK1 | CCNT2 |
| RAP2B | ELP5 | SOX11 | CAMK1D | PLEKHA2 | DDIT4 |
| SIX4 | KDM8 | FOXN3 | CLINT1 | ORAI2 | RCC2 |
| ARID4B | ITM2C | PLEKHA8 | MAP3K3 | PIAS2 | GLIS2 |
| NMNAT2 | HDAC4 | ACOT7 | DDX6 | GATAD2B | STX4 |
| TMEM178B | GOSR1 | CTNND2 | VGLL4 | GJC1 | ACLY |
| MECP2 | HAP1 | GNAI1 | CD40 | SLC43A2 | PHACTR4 |
| SCN3A | ANGPT2 | HGF | SAP30L | BASP1 | CREB1 |
| DYRK2 | ZNF705D | KLF6 | IKZF2 | SBF1 | NYAP2 |
| CPSF6 | CENPN | NRP1 | AKT3 | SHISA4 | WRNIP1 |
| NREP | PHF5A | SLC38A2 | HIPK3 | DUSP13 | DNAJC27 |
| NTNG1 | RELA | POU3F2 | CCDC107 | RETREG3 | C17orf58 |
| TFDP1 | PELI3 | TMEM135 | JPH1 | ITGB3 | SH3PXD2A |
| CBLL1 | TMEM40 | CERS6 | CLIP1 | CMTM6 | PRDM16 |
| ZNF516 | DUOXA1 | NFYB | CSRNP2 | USP49 | PLBD2 |
| BTBD7 | STPG4 | DCP2 | TAGLN2 | STX5 | KLF6 |
| GRSF1 | FHIT | ERG | BRCC3 | ATP13A3 | SLC26A9 |
| BNC2 | FADS1 | TMEM170B | SLC7A1 | SYNPO2 | PRPF38A |
| ANP32A | EID1 | DUSP3 | HTR2A | MRPS27 | LRRC73 |
| TADA2B | RELN | MAP2K4 | FNDC3A | TEP1 | FUT9 |
| CACNG2 | TP53INP2 | FRMD6 | TBC1D15 | GGA2 | ARRB1 |
| LZTS3 | MXD1 | GPR137C | KIF3A | TRAF3IP2 | PRCP |
| ADCYAP1 | PLEKHG4B | EXOC5 | GCLM | FKBP9 | DEUP1 |
| VAPA | BCL11A | BEND4 | PAFAH1B2 | TMEM33 | IL6R |
| FOXO3 | BAG1 | ATP8A1 | PRPSAP2 | PLA2G16 | FAM53C |
| ZNF521 | GM2A | BNC2 | KLHL28 | PAIP2B | PAFAH1B2 |
| NACC2 | MFAP3 | EVI5L | YTHDC1 | JDP2 | TAGLN |
| RND3 | PWWP2A | SRSF10 | KANK2 | FAM13B | MPZL3 |
| ATL3 | CREB1 | CRLF3 | TPM4 | CD96 | KIRREL3 |
| CDH3 | WWC1 | DCUN1D3 | SLC16A2 | TRIOBP | PRKAR2A |
| FAM199X | UNC5A | RALGPS2 | CNTN4 | CDS2 | NRAS |
| CHRNA5 | UIMC1 | ABHD18 | SEL1L3 | DPP9 | WARS2 |
| FUT1 | VEZF1 | FNIP2 | RUNX3 | KIF3A | CNOT6L |
| SEC61A1 | SYT13 | C6orf120 | MPZL1 | CCDC69 | CAV3 |
| CR2 | KBTBD4 | CD47 | RAPGEF2 | LARP1 | SRSF10 |
| ADGRF4 | ANKS6 | STRN | QKI | PARP11 | NCMAP |
| NBN | SNX30 | GPR6 | TBPL1 | ZNF592 | ZNF704 |
| NCKAP1 | NOVA2 | PEG3 | NR4A2 | UNKL | SYNE1 |
| C6orf106 | CALN1 | DOLPP1 | MDFI | IKZF2 | SMLR1 |
| SLC38A2 | CDK6 | TSC1 | DFFA | DNPEP | CTIF |
| PLAGL2 | MBTPS2 | FAM168B | MTMR14 | GREB1 | AMMECR1L |
| BMPER | THAP1 | ACVR2A | SP7 | NRXN3 | MAOA |
| DCAF17 | RB1CC1 | KLF5 | DEK | NWD1 | RBSN |
| DDX47 | SESN3 | RASSF2 | ZNF772 | MAP3K3 | MIS18BP1 |
| WDR93 | VPS26A | ARL5B | ZFAND3 | NSL1 | ARID5B |
| FAM126B | ZNF275 | ZNF805 | UTP15 | TMEM164 | ZNF217 |
| UNC80 | TRPS1 | ZNF621 | GALNT14 | GPR26 | SRSF7 |
| ZNF507 | PAN3 | MCTP2 | GPHN | NPR3 | E2F2 |
| CDC42BPA | FEM1C | LRRC28 | PTGR2 | TGFBR1 | CDK6 |
| RHOU | KDM5A | CREB1 | NWD1 | KSR2 | TMEM178B |
| HNRNPU | KIF13A | VWC2L | MACC1 | MLEC | MMP14 |
| VPS41 | EFNB3 | SFXN1 | RPS6KA6 | POM121 | TMEM201 |
| SLK | GNG2 | SIM2 | COMMD9 | ADAM22 | FOSL2 |
| CACUL1 | 44085 | PPM1A | CCL28 | RBM48 | ITGB8 |
| TEAD1 | SH3GL2 | PRR11 | TGFBR1 | FAM133B | RPL36A-HNRNPH2 |
| POM121 | RDH8 | DDX5 | PRKD2 | SEPHS1 | ENO1 |
| MEF2C | ADGRL1 | LBR | SLC25A11 | P2RY8 | TNFRSF1B |
| REPS2 | TSR1 | IRF2BP2 | BTF3L4 | RASSF8 | FAM173B |
| SRGAP2 | CHST10 | ITGB8 | TMEM63B | DNAJC22 | CAVIN4 |
| ZYG11A | PPARGC1A | XIAP | FBXO9 | TRABD2B | MICALCL |
| TMEM68 | SZRD1 | MPPED2 | TSKU | KANK4 | FGFR1OP2 |
| MTMR1 | NKAIN1 | PDCD4 | NDUFC2 | SENP6 | FMNL3 |
| TMEM64 | TAOK1 | FAM149A | PHF20L1 | PLAG1 | C1orf21 |
| ZHX1 | RHBDL3 | FSD1L | BAZ2A | KCNN3 | NAV1 |
| GPRIN3 | VPS37A | KIAA1958 | FAM19A2 | MYPN | COA7 |
| PAN3 | BNIP3L | CACNA1B | ATP6V0B | ZC3HAV1 | NT5E |
| HMGA2 | ILDR2 | GEMIN8 | MCF2L | TMEM178B | CADM3 |
| FRS2 | XPR1 | TXLNG | UBTF | MAMLD1 | RUFY2 |
| CHST11 | JOSD1 | SYTL5 | CFL2 | ALPK1 | OTUD6B |
| MCOLN3 | TTLL12 | PYROXD1 | FAM46C | MTUS2 | ANXA13 |
| ABHD13 | SNAP25 | YAF2 | SRGAP2B | KLHL3 | OLA1 |
| FAM71F2 | CTDSPL2 | PPHLN1 | SLC4A4 | ACVR1B | RXFP2 |
| FAM222B | L3MBTL3 | ELK4 | CNTLN | BSDC1 | CCNI2 |
| SRGAP2B | LMAN1 | DTL | ZNF423 | CLSPN | ZBTB16 |
| CERS2 | DENND6A | C6orf141 | ELOVL7 | ELOVL1 | CADM1 |
| SRP72 | ST6GALNAC4 | RNF170 | CCDC149 | BORA | H2AFX |
| ARMC1 | ZER1 | TFAM | SH2D5 | AOC3 | ACVRL1 |
| TMEM50B | RPRD1B | EIF4EBP2 | TTC25 | ZNF445 | ARHGAP9 |
| ABCG1 | SYS1 | TPK1 | SLC7A2 | SLC4A4 | ALDH5A1 |
| C16orf87 | FAM210B | NOM1 | STC1 | ISLR | MTF1 |
| ZDHHC18 | HIF1AN | MAP7D3 | DNAJA3 | SYP | SUGT1 |
| SGCZ | DTX4 | HTATSF1 | POGK | NLGN3 | ARHGAP27 |
| ADRA1A | TERT | MAP3K20 | RASAL2 | GSG1L | NXPH3 |
| ZNF704 | BLCAP | WIPF1 | APH1B | USB1 | MIEF2 |
| MAF | CBY1 | GLS | EIF2AK2 | ARHGEF28 | DCAF1 |
| FAM227A | PPM1D | ZNF793 | NXPE3 | SETD5 | NOP9 |
| TMEM200C | CELF1 | DYRK2 | EPN1 | NIPAL3 | ITGA10 |
| INAFM2 | POLR2E | FRS2 | ADNP | CEP85 | USP46 |
| TMEM87A | CAMK2A | CNOT2 | BZW1 | NEFM | MEGF11 |
| AAK1 | NOP2 | LYRM7 | KLHL6 | ITPRIPL2 | ELAVL1 |
| CLVS2 | ERBB4 | S100PBP | CBFB | NFAT5 | ICOSLG |
| DCBLD2 | PRPF4B | PPCS | IGF1R | PATZ1 | DGCR8 |
| EPB41L5 | SLC25A36 | POT1 | DDX17 | FAM227A | RAB3C |
| FITM2 | GK5 | COX18 | SERINC5 | TNRC6C | KCNJ13 |
| KCNS1 | KCNK3 | AAGAB | TGFBR2 | TMEM87A | N4BP2 |
| OSBPL8 | DLST | TBC1D2B | MAP4K2 | NDOR1 | ATP5IF1 |
| MTMR10 | SEL1L | AR | ERBB4 | SLC16A12 | NEFM |
| MAPK1 | MBTD1 | ZNF629 | NUFIP2 | PANK1 | CA7 |
| ARID2 | TMEM104 | FUS | PSAT1 | BTRC | MLKL |
| BRWD1 | ARMC7 | MYLK3 | BTG1 | FADS1 | FASLG |
| SDF2 | TP53BP2 | C16orf87 | CPEB4 | ST6GAL1 | SEC14L4 |
| ASF1A | AKT3 | NECAP2 | PTP4A2 | SP1 | EDNRA |
| EIF2S3 | TRPC5 | MACO1 | PIGF | PDCD4 | ZNF106 |
| KPNA1 | DCAF10 | CNP | PLAGL2 | VPS53 | TMOD3 |
| SH3TC2 | FAM120AOS | SHISA9 | PRDM2 | PRKCA | FAM96A |
| SEC62 | VPS53 | HSDL1 | ADAM19 | TRPS1 | DSE |
| SOX11 | CCDC186 | KLHL36 | GABARAPL1 | MAPK13 | HEY2 |
| FOXN3 | CACUL1 | POGK | FAM174B | CYCS | SMAD4 |
| CAPRIN1 | CCNL2 | VAMP4 | SLC25A36 | CORO2A | WDR33 |
| EBF3 | SLC35E2 | TNFSF4 | ZFYVE1 | PLEKHM3 | APCDD1L |
| SLC6A3 | KSR2 | RAPGEF2 | DNAL1 | LRRC27 | HDAC4 |
| SLC31A1 | PCLO | MFAP3L | AREL1 | ZNF426 | MYCBP |
| YWHAG | SFMBT2 | WWC2 | SPTLC2 | CAMK4 | SIRT1 |
| LIN28B | CCDC3 | TNRC6C | CRTC1 | CCND2 | CSF1R |
| C11orf87 | ZNF37A | ZNF12 | HECW2 | TAF5L | TIAM1 |
| KDM7A | GPR143 | PRTG | VEZF1 | CDK13 | BTG1 |
| DUSP9 | TXLNG | FAM161A | TRIM44 | ELOVL3 | VSNL1 |
| PYURF | SOX5 | AAK1 | PCSK5 | NOVA2 | NTRK2 |
| C19orf47 | KCNH1 | TRDN | TMOD1 | TRIM66 | MTDH |
| KITLG | GAB2 | RSPO3 | GFRA1 | CALCR | PPM1K |
| PRDM6 | GATAD2B | SEH1L | ZFHX4 | SMARCD1 | TBC1D12 |
| SNIP1 | GBA | RIOK3 | CASZ1 | IPO9 | SFXN1 |
| DOCK4 | FAM122C | MAPRE2 | RAB14 | GRHL2 | WNT1 |
| MED9 | PDP1 | SYT4 | NAA25 | E2F3 | TMED4 |
| NLK | ST3GAL1 | SNTN | YWHAG | PRR3 | TNFRSF10D |
| ABHD5 | PDK1 | WDR33 | LARP4B | MYOCD | RAP2B |
| CELSR3 | DNAJC10 | MBD5 | CELSR1 | GABRA4 | EFR3B |
| PNN | KPNA3 | USP49 | ARPC5 | RAB11A | CCNA2 |
| SGK3 | ZNF780B | ZNF334 | PLXNA2 | SZRD1 | MAPK8IP3 |
| KCMF1 | MEGF10 | LMLN | NSUN4 | CAPZB | TTC9 |
| TCF7L1 | AMIGO1 | BRD3 | SH3BGRL2 | UBFD1 | DGKE |
| ZSWIM6 | ATF7 | PTEN | TMEM135 | NR1D2 | CCDC47 |
| FBXL20 | TSPAN31 | HOXB5 | SCAMP3 | WDR5B | BATF3 |
| FAM167A | E2F3 | PPP1R15B | VPS26A | ONECUT2 | PROX1 |
| SOS1 | LIX1 | ZBTB34 | EIF4EBP2 | FOXP1 | HOXA4 |
| SLC25A28 | SNIP1 | TET1 | KDM7A | STX3 | ZNRF2 |
| PLPBP | RIMKLA | GATA6 | TET2 | IGF2 | KCNE5 |
| ARF6 | CCDC103 | EPHA7 | ARL11 | PURB | IL13RA1 |
| CSTF2T | SH3GL1 | SLC35D1 | AP3S1 | CISH | TRUB1 |
| POLK | ZBTB47 | SCD5 | SEMA6A | XPR1 | CACUL1 |
| MLLT10 | VANGL1 | IRF2BPL | C5orf15 | PPM1A | LZIC |
| SYT14 | CORIN | RAB8B | SKP1 | BMP8B | STOX2 |
| AASDHPPT | RAB11A | PRKAA2 | DDX46 | BCAS4 | PLCXD3 |
| FAM81A | LMAN2L | CHD2 | PTGFR | C18orf32 | GABBR2 |
| TUSC2 | ESS2 | FAM126B | NECTIN1 | TNFAIP8L1 | FBXO46 |
| SH3BGRL | ZDHHC8 | TMEM237 | RAB5C | FHDC1 | FTL |
| EBPL | RP2 | FOXC1 | ATXN7L1 | CAVIN1 | H3F3C |
| SYNE3 | NLRP2B | WWTR1 | MDFIC | HMGB1 | SMAP1 |
| KRTAP4-11 | ITGAL | P2RY1 | CLMN | TNFSF15 | KAT6A |
| MMP13 | GNAO1 | RHPN2 | SMCR8 | SLC35G1 | EIF4EBP2 |
| B3GAT1 | GBX2 | PPM1E | KLHL18 | RBM3 | PALD1 |
| PRX | LRRFIP1 | KCNJ2 | ZNF512B | POFUT1 | DUSP13 |
| ADAMTSL1 | SRGAP3 | RRP15 | SRGAP3 | DNAJB13 | MECP2 |
| NDUFB10 | E2F2 | ADD3 | PHACTR4 | FFAR4 | ODF1 |
| HOOK3 | GPR3 | RBM20 | TAOK1 | IP6K2 | CDX2 |
| ART4 | MGRN1 | ADRB1 | GIT1 | SMIM7 | EIF4EBP3 |
| ZNF280B | DEPDC5 | FAM160B1 | RAB11FIP4 | WWC2 | SLC6A17 |
| OCLN | APOBEC3B | SLC18A2 | KLHL11 | SEC14L4 | CBL |
| IER3IP1 | RSPO4 | CFAP97 | USP31 | PPIE | ZBTB39 |
| GID4 | SLC23A2 | HMGCS1 | ZNF704 | ZNF551 | CENPV |
| APBA1 | RNF150 | FBXW2 | CRISPLD2 | TMOD2 | ZNF609 |
| FGF2 | RNASEH2C | HCAR2 | RASSF2 | SLFN13 | MTHFD2 |
| MMP9 | MYO15B | HCAR3 | TM9SF4 | BCL11B | MAT2A |
| COL4A4 | USP14 | CACNA2D1 | VAPA | SAR1A | HUNK |
| PPP2R5E | MYEF2 | PROSER2 | TULP4 | WNT7B | HOMER1 |
| NOXRED1 | MYO5C | DIP2B | RNF216 | CXorf38 | PDS5A |
| DYNLL2 | MYH15 | NUCKS1 | BLOC1S6 | XPOT | PADI2 |
| C1orf115 | CALHM5 | IRAK1BP1 | NAA50 | HIF1AN | CLIC4 |
| SPTY2D1 | CTIF | GPAT4 | FBXO40 | SNX2 | UNC5D |
| ZBTB5 | CCDC68 | SOX17 | ZNF521 | XIAP | KIAA0040 |
| DCAF10 | DCP1A | CCDC6 | ONECUT2 | ZNF7 | PRCD |
| CNTNAP3 | NSUN3 | ARID5B | NRARP | TLR7 | TTYH3 |
| CNTNAP3B | YEATS2 | RBM33 | NAA40 | ACOT9 | ACVR2B |
| PTAR1 | LPP | SDC2 | STAT1 | RBM41 | TYRO3 |
| GOLM1 | ROCK2 | ARHGAP24 | F11R | LGSN | GATM |
| VTI1A | CCND3 | UNC5C | NIPSNAP1 | UBOX5 | CD207 |
| C5orf51 | HIF1A | TRHDE | MMP14 | ZMAT3 | PAIP2B |
| SUDS3 | SENP1 | HS2ST1 | BRAF | TMEM127 | NUS1 |
| POM121C | EIF4EBP1 | TMEM56 | PADI1 | SLC35F5 | ADAT2 |
| SHOX | MAP3K11 | MFSD14A | AGTRAP | KIAA1549 | ASB14 |
| FRMPD4 | SEMA4C | CBL | WSB1 | DNAL1 | PCNP |
| PDE3A | IGLON5 | STAT5B | BCLAF3 | ENTPD4 | FMNL2 |
| ARF3 | FOXC1 | CBX1 | CCDC85C | RAI1 | EMILIN3 |
| GPR37L1 | SOX9 | ZNF516 | MYOCD | LYRM7 | TNKS2 |
| ZNF451 | MEX3A | ARHGEF18 | FXN | LRRC15 | FBXO45 |
| CFAP206 | ARL5B | YY1 | CCND2 | ACOX1 | BMP7 |
| TACC1 | C7orf33 | ZNF660 | KCNA6 | NANOG | LBP |
| REEP3 | LIN52 | FOXA1 | PHB2 | GPBP1 | CLPTM1L |
| ESYT2 | NR3C1 | SLAIN2 | PLEKHM3 | ISY1 | SCD |
| MMGT1 | CASTOR2 | KIAA1211 | EPHA4 | SERINC3 | RMND5A |
| RAD21 | PLEKHM1 | IGDCC4 | ADAMTS2 | PCDH1 | RBL1 |
| DNAJB14 | RPA1 | PRELID3B | GK5 | TIGAR | CYCS |
| PPP3CA | YIPF5 | IFNAR1 | FANCA | JPT2 | TTC33 |
| SLC7A1 | DUSP16 | SON | ATAD2B | RHBDD1 | CXCR2 |
| CCER2 | SLCO3A1 | SRCAP | SLC5A6 | SSR1 | DAD1 |
| RASSF3 | SPEG | SREK1IP1 | ALDH6A1 | PIK3CB | N4BP3 |
| METTL25 | DGKE | UGT1A6 | EML5 | PFN2 | C6orf201 |
| NRG2 | APPBP2 | EDEM1 | CYB561 | PSEN1 | KCNAB1 |
| HS2ST1 | CDR2L | NIPAL3 | TMEM206 | ELMSAN1 | AGBL5 |
| TRMT13 | STOX2 | PPP2R2A | LRCH2 | ZDHHC22 | SUSD6 |
| TP53AIP1 | TMEM38B | ITPRIPL2 | ZDHHC9 | DIO2 | VASH1 |
| APLP2 | PAPPA | ZNF704 | PHF21A | PDE4C | UTP18 |
| NEUROD4 | DENND1A | TADA1 | FRMD3 | YPEL2 | DUSP10 |
| ELOVL2 | RALGPS1 | RNF185 | IPPK | STRADA | AIDA |
| ZNF362 | TEAD1 | PLAGL2 | TRAM1 | TLR5 | STUM |
| PRR15L | SH2B3 | FOXJ1 | PRLR | C1orf198 | SOSTDC1 |
| SALL3 | BAZ1B | TAB2 | HCN1 | HNRNPU | AIFM1 |
| KCNJ12 | CACNA2D1 | NXPH1 | MOCS2 | PDE1C | ELF5 |
| SLC25A20 | DVL2 | FBXL2 | PPP3R2 | TMED4 | PRDM11 |
| INSM2 | ARRDC3 | CLASP2 | PTPN3 | PRPS1 | FNBP4 |
| SV2A | PLXNB2 | OXSR1 | KIAA1958 | NCR3LG1 | CNTNAP3 |
| SLAIN2 | RASSF8 | MAPK6 | TLR4 | TRAF6 | CNTNAP3B |
| PDGFRA | IPO8 | OSBPL11 | ZBTB43 | PI15 | STN1 |
| RUFY3 | SERTAD4 | KIAA0408 | MRPL17 | CLCN6 | SORCS1 |
| ERMP1 | PCMTD1 | MIB1 | DDX54 | LRRC38 | PHF13 |
| CCDC171 | ATP11C | TTR | RILPL1 | PLEKHG4B | AGMAT |
| ARIH1 | EBPL | TRAPPC8 | STEAP2 | TMEM245 | RGS3 |
| ANKRD34C | PSD2 | DUSP7 | ENKUR | ERCC1 | TNFSF15 |
| ZNF333 | SLC6A17 | PTPRG | ITPR2 | OPA3 | MVB12B |
| POLR1A | VPS26B | RYBP | GXYLT1 | PPFIA3 | UNC119B |
| RPL31 | C6orf47 | SPOPL | KCNH1 | ATF5 | GALNT17 |
| C22orf39 | HS6ST3 | NXPH2 | CC2D1B | NUP98 | TBL2 |
| GRK3 | PDAP1 | FKBP5 | CD109 | HCAR1 | CERK |
| PAPD5 | LHFPL3 | ELMO2 | FUT9 | POM121C | PDK3 |
| PCGF3 | CELF5 | PGAM4 | UVRAG | NMT2 | SLC48A1 |
| SEPSECS | KLHL18 | PEX11A | PAK1 | DENND5B | TESK2 |
| PAX7 | NFIB | C16orf58 | SESN3 | RAB3B | TRABD2B |
| HMGN2 | ZNF609 | MDM4 | TRPC6 | CC2D1B | CDKN2C |
| CACNB1 | PLEKHO2 | DPYSL3 | CHCHD3 | LEXM | CDC5L |
| SEC14L5 | TCF7L1 | SH3TC2 | LAMTOR3 | LEPROT | OPN5 |
| DCUN1D3 | ENC1 | GEMIN5 | GJA3 | SLC35D1 | ZNF451 |
| CDH8 | FAM169A | FNDC9 | LYPD5 | CLIC5 | GATAD2B |
| HMGXB4 | NPPC | SLCO3A1 | TOMM40 | BACH2 | MEX3A |
| ELFN2 | PCGF3 | STRADB | MDM1 | B3GNT6 | TSPAN33 |
| SMARCA5 | EPHA8 | SSR1 | FRS2 | AMOTL1 | DGKI |
| H3F3B | PDIK1L | PHC3 | TMCC3 | ZNF365 | CNGA2 |
| MRM2 | FNDC8 | MBOAT2 | FER | SAMD8 | WDFY3 |
| EPM2AIP1 | ERI1 | FAM84A | SLC25A46 | MECP2 | TMEM155 |
| CHP1 | SLX4 | SIX1 | BCL10 | SPRED3 | NEMP2 |
| ARGFX | ZNF704 | ZNF431 | GRIK4 | HMGA2 | FGD6 |
| PLXND1 | THAP11 | DYNLL2 | VWA5A | TMBIM4 | HTR3A |
| PRKD3 | TERF2 | APPBP2 | HOXC11 | CPM | ACVR1B |
| RHOQ | USP10 | TTYH2 | EEF1AKMT3 | DCN | ZNF740 |
| TCF21 | HMGXB4 | IBA57 | ATXN7L3 | MIGA1 | ANKRD52 |
| PDE7B | GTPBP1 | EGLN1 | KPNB1 | KMT2A | RIOK2 |
| C18orf25 | RTL6 | HOXA11 | CAV2 | BCL9L | YARS |
| SFMBT1 | GALNTL6 | JAZF1 | EIF5 | C2CD2L | KIAA0319L |
| ZNF264 | H3F3B | NOX1 | FAM189A1 | CLMP | INPP5B |
| UBAC1 | ACVR2B | FBXO3 | TVP23C-CDRT4 | MAP3K12 | ABHD13 |
| NCKAP5 | TCF12 | RAG1 | FAM222B | FNDC5 | NBR1 |
| PTEN | SLC35F1 | ZFAND5 | DALRD3 | RIMKLA | IGF2BP1 |
| DNMBP | ASXL3 | LRRC27 | VANGL1 | DSEL | ZNF652 |
| CWF19L1 | PXK | KIF1B | PDE7A | ZADH2 | WFIKKN2 |
| TTC14 | LENG8 | KAZN | FAHD2A | CELF5 | PDAP1 |
| TPRG1 | SOGA1 | PDZD2 | ADRA2B | ZNF557 | CCDC71L |
|  | VSTM2L | NIPBL | PRELID3B | CTC1 | CCDC85C |
|  | KCNK15 | MOCS2 | UBE2G2 | GID4 | OTUD7A |
|  | ARFGEF2 | RAD23B | CNEP1R1 | GRAP | NDEL1 |
|  | ZMIZ1 | RABGAP1 | MARVELD2 | SEC22C | TMEM199 |
|  | LPL | OPA3 | TNPO1 | CCR2 | ZBTB47 |
|  | CDH2 | TMEM41B | ABLIM2 | LRRC2 | UHRF2 |
|  | S100A1 | GIT2 | CCR7 | PLXNB1 | ARIH1 |
|  | TSTD2 | EGFR | MTUS1 | ARIH2 | HCN4 |
|  | C12orf49 | VKORC1L1 | ZNF395 | TUFT1 | KIAA0556 |
|  | PFN2 | LMTK2 | MAF | ACO1 | HTT |
|  | NFKB1 | FRMD4A | PRR14L | UBE2R2 | DOK7 |
|  | YAP1 | PRRG1 | DUSP15 | ZNF700 | UBE2K |
|  | NPLOC4 | PTHLH | PCDH10 | TACR1 | IFFO2 |
|  | VASH1 | TFAP2B | SMARCA5 | ATOH8 | TRNAU1AP |
|  | ZNF705A | BAG2 | LRAT | PTK6 | ZNHIT3 |
|  | ARL10 | TBX18 | UBALD2 | GRK3 | PIP4K2B |
|  | ZNF354C | HOOK3 | EMILIN2 | NLN | CDC6 |
|  | FOXL2NB | FZD4 | STXBP5L | SREK1 | DLGAP2 |
|  | MC1R | ASH1L | PCYOX1 | LRPAP1 | CHRNA2 |
|  | DNMT3A | DDIT4L | PDE7B | PPARGC1A | SHISA9 |
|  | FOSL2 | B3GALT1 | RPRD1A | KLHDC7A | ERI2 |
|  | TMED10 | SESTD1 | ZNF550 | OTUD3 | EEF2K |
|  | FAM104A | FBRSL1 | INHBB | MYOM3 | THAP11 |
|  | MIF4GD | FLT1 | HLA-DOA | RNF135 | CABP7 |
|  | WNT9A | USPL1 | TBC1D22B | SOCS7 | TRIOBP |
|  | EEPD1 | KBTBD6 | PHF20 | LASP1 | MGAT3 |
|  | LPAR4 | ZNF230 | CPEB3 | ZNF500 | ULBP1 |
|  | NXF2 | BBS10 | BLOC1S2 | SEC14L5 | THSD7A |
|  | NXF2B | AP3S1 | DTX4 | ARL6IP1 | GLDN |
|  | CHRDL1 | C5orf63 | MYO6 | CYB5B | ONECUT1 |
|  | MRM3 | C5orf24 | TMOD3 | AP1G1 | CD80 |
|  | GPR26 | PURA | FSCN1 | GLG1 | VIT |
|  | SKP2 | ST6GALNAC5 | SERPINE1 | KREMEN1 | FIBCD1 |
|  | C11orf21 | AMPD2 | ADAM17 | H1F0 | MALL |
|  | MMAB | PAFAH1B2 | ABRACL | DZANK1 | PTGIS |
|  | MSI1 | MPZL2 | MYO5A | TP53INP2 | ZFP91 |
|  | B3GNT4 | SNIP1 | ACTB | GAB1 | HNRNPUL2 |
|  | BRI3BP | HS6ST3 | SOX11 | VAPA | LPP |
|  | POM121 | TFEC | SMAD4 | VHL |  |
|  | TMEM167A | EIF5 | CTNND1 | CCDC174 |  |
|  | MFSD4A | TOM1L2 | ARF6 | ZSCAN29 |  |
|  | EFCAB14 | SNRK | SNX24 | TRPM7 |  |
|  | CD2AP | PDGFRA | ANKRD28 | BTLA |  |
|  | SIM1 | THAP9 | SLC26A2 | GSK3B |  |
|  | NSD3 | SLC1A1 | ZNF451 | KALRN |  |
|  | PGR | ZDHHC21 | MTDH | MTA3 |  |
|  | S100A2 | OAZ2 | KLF4 | GFPT1 |  |
|  | UBE2Q1 | PAQR5 | CDH2 | CCDC68 |  |
|  | VSTM4 | LONRF2 | MAP2K6 | NISCH |  |
|  | EIF4EBP2 | MAPK1 | TMEM9B | EPHA6 |  |
|  | TMEM178B | PIN4 | FAM3C | PCNP |  |
|  | REPIN1 | NKD1 | SAMD5 | ALCAM |  |
|  | MECP2 | SETD9 | UBR7 | CACNG8 |  |
|  | C4orf54 | PCGF3 | RPS6KA3 | GALP |  |
|  | BTG1 | TMEM129 | SENP1 | DUXA |  |
|  | WDR55 | FAM184B | GPRC5A | ZNF805 |  |
|  | BCL9L | LCORL | SPSB4 | TBC1D13 |  |
|  | MSANTD2 | PAQR7 | SELENOI | VAV2 |  |
|  | SNX19 | ZNHIT3 | BBOF1 | KCNJ3 |  |
|  | GALNT6 | TNKS | KIAA0355 | SAYSD1 |  |
|  | SMIM13 | TRMT9B | MBTD1 | SOGA1 |  |
|  | GAMT | METTL9 | EXOC8 | SLC12A5 |  |
|  | SPRED1 | IGSF6 | FAM126A | DVL3 |  |
|  | NLGN2 | ASTN1 | CREB5 | EP300 |  |
|  | ZBTB4 | STX6 | BRWD3 | CBL |  |
|  | NTN1 | H1F0 | QSER1 | ZBTB7A |  |
|  | FYCO1 | PRND | CAPRIN1 | MMP14 |  |
|  | GNAT1 | CDS2 | RBM20 | NKD1 |  |
|  | RTN1 | POFUT1 | TRIO | TTLL12 |  |
|  | PTGFRN | PCDH10 | ZBTB6 | PLXDC1 |  |
|  | PPP1R42 | SLC10A7 | IPO7 | GPR182 |  |
|  | SGSM2 | POU4F2 | UNC119B | ZNF514 |  |
|  | RETSAT | DNAH17 | AKAP9 | FRAT2 |  |
|  | FAHD2A | C1GALT1 | STAM | ATCAY |  |
|  | PTK6 | SLC30A4 | ARHGAP21 | GK5 |  |
|  | WDR4 | TLN2 | PRKX | ULK2 |  |
|  | MICAL3 | CD80 | KLHL15 | LNPK |  |
|  | TTC28 | MTA3 | ELK4 | LTBP2 |  |
|  | FAAH2 | TMEM17 | SSBP3 | SYNJ2BP |  |
|  | SNX20 | SERTAD2 | DENND4B | CYTIP |  |
|  | FTO | PRDM1 | UBN2 | TIMM50 |  |
|  | COQ9 | METTL24 | CSMD3 | NMNAT1 |  |
|  | MAST4 | CLVS2 | ZHX2 | PCP4L1 |  |
|  | SSH2 | FAM210A | SLC25A30 | SLC7A11 |  |
|  | LZTS1 | STIMATE | LOX | PIGR |  |
|  | CHMP7 | TMF1 | CYR61 | EXO5 |  |
|  | EBF2 | ZNF677 | LRRC8B | TSPAN11 |  |
|  | COG7 | IL1A | PAN2 | TEAD1 |  |
|  | COTL1 | RBMS1 | MTF1 | DDOST |  |
|  | APOBEC3G | MDGA1 | NDFIP2 | ZFP14 |  |
|  | HSPA4L | SYS1 | SLITRK6 | ZMAT2 |  |
|  | SSSCA1 | C2orf69 | POTEM | ADAM19 |  |
|  | WDR45B |  | DOK6 | CHD2 |  |
|  | ULBP1 |  | EFNB3 | GCM2 |  |
|  | TMEM181 |  | PMP22 | IGFBPL1 |  |
|  | DYNLT1 |  | SLC7A8 | PAPPA |  |
|  | RSPH3 |  | FBXO34 | NDC1 |  |
|  | FRMD5 |  | EXOC5 | FOXD3 |  |
|  | MINDY2 |  | USP46 | PKHD1 |  |
|  | DAPK2 |  | UBA6 | FZD4 |  |
|  | RUVBL1 |  | BMP3 | GDI1 |  |
|  | ZNF705E |  | GLIS3 | MMP16 |  |
|  | PTPN2 |  | CORO2B | TSPYL5 |  |
|  | ZSCAN22 |  | SLC35E1 | METAP1 |  |
|  | ZNF584 |  | RGPD3 | PURA |  |
|  | INHBB |  | RGPD4 | CYB561D1 |  |
|  | FANCE |  | GCC2 | BSN |  |
|  | HNF4A |  | MRGBP | ZNF317 |  |
|  | WFDC10A |  | CRKL | PAFAH1B1 |  |
|  | RAB22A |  | RBM3 | TET3 |  |
|  | PDLIM1 |  | PAPD5 | PAPD5 |  |
|  | TTC14 |  | NUDT21 | ZSWIM6 |  |
|  | PIGG |  | ZNF436 | C6orf120 |  |
|  |  |  | MYO1D | ZNF865 |  |
|  |  |  | PSD3 | PKP4 |  |
|  |  |  | CCDC25 | MDGA1 |  |
|  |  |  | ESCO2 | FLT3 |  |
|  |  |  | RBPMS | SRCIN1 |  |
|  |  |  | MOSMO | SMUG1 |  |
|  |  |  | RRP7A | COX19 |  |
|  |  |  | ABCE1 | FGD6 |  |
|  |  |  | TLL1 | ABHD15 |  |
|  |  |  | ACTG1 | SKIDA1 |  |
|  |  |  | ARHGAP28 | MIS18A |  |
|  |  |  | INO80 | MED16 |  |
|  |  |  | CHP1 | MMAB |  |
|  |  |  | PLXND1 | KLHL21 |  |
|  |  |  | EYA4 | PIGM |  |
|  |  |  | GALNT1 | RRP1B |  |
|  |  |  | APPL1 | CAMK1D |  |
|  |  |  | HS6ST1 | TRAPPC10 |  |
|  |  |  | ACVR2A | KCNK3 |  |
|  |  |  | NUDT3 | CXorf21 |  |
|  |  |  | ERLIN1 | PNRC1 |  |
|  |  |  | TTC14 | POLQ |  |
|  |  |  | SENP2 | PIK3C2A |  |
|  |  |  | SOX2 | FAXC |  |
|  |  |  | MYC | PWWP2A |  |
|  |  |  | IFNB1 | C16orf58 |  |
|  |  |  | FZD7 | APOPT1 |  |
|  |  |  | EIF4E | ZNF582 |  |
|  |  |  | CDK4 | TMOD3 |  |
|  |  |  | VEGFA | SLC33A1 |  |
|  |  |  | ESR1 | RABIF |  |
|  |  |  | JAG1 | KCNK5 |  |
|  |  |  | CTGF | DNASE2 |  |
|  |  |  | ALPPL2 | ABHD2 |  |
|  |  |  | CEP19 | SPPL3 |  |
|  |  |  | KREMEN1 | NPHS1 |  |
|  |  |  | EPAS1 | PRPF38A |  |
|  |  |  | HMGA2 | RUNDC1 |  |
|  |  |  | SESN2 | ESR2 |  |
|  |  |  | SLC16A10 | QRFPR |  |
|  |  |  | CYP2C19 | C12orf49 |  |
|  |  |  | IGFBP5 | ZNF578 |  |
|  |  |  | ODR4 | NOL9 |  |
|  |  |  | SLC22A9 | METTL8 |  |
|  |  |  | ZNF445 | STX4 |  |
|  |  |  | PANK1 | ANKS4B |  |
|  |  |  | HBEGF | GNB5 |  |
|  |  |  | TGFBI | SH3BP5 |  |
|  |  |  | MCM2 | NUDT3 |  |
|  |  |  | SPTLC1 | GPRIN3 |  |
|  |  |  | LRP6 | ETV3 |  |
|  |  |  | WDR73 | STAC2 |  |
|  |  |  | HDDC3 | CDIPT |  |
|  |  |  | ORC2 | RAB21 |  |
|  |  |  | RETREG2 | GOLGA2 |  |
|  |  |  | PAX3 | ATP9A |  |
|  |  |  | UBTD2 | PRKAB1 |  |
|  |  |  | SFXN1 | TMEM174 |  |
|  |  |  | MAPK9 | NEK8 |  |
|  |  |  | C6orf201 | ATAD2B |  |
|  |  |  | SNRNP48 | STAT5B |  |
|  |  |  | PAQR9 | PHLDA1 |  |
|  |  |  | PFN4 | KPNA6 |  |
|  |  |  | DNMT3A | PROSER2 |  |
|  |  |  | RAD51B | THAP6 |  |
|  |  |  | ALKBH1 | TPMT |  |
|  |  |  | USP32 | ZNF70 |  |
|  |  |  | APPBP2 | MPPE1 |  |
|  |  |  | ERN1 | SP2 |  |
|  |  |  | MTURN | SH3TC2 |  |
|  |  |  | C11orf58 | FAM117B |  |
|  |  |  | RCN1 | KCNE4 |  |
|  |  |  | IGFBPL1 | SH3BP5L |  |
|  |  |  | NINJ1 | RRM2 |  |
|  |  |  | SHTN1 | ANGEL1 |  |
|  |  |  | RNF207 | SPTLC2 |  |
|  |  |  | AGMAT | ZC3H14 |  |
|  |  |  | CMBL | THAP8 |  |
|  |  |  | ERP44 | VEZF1 |  |
|  |  |  | ELP1 | DYNLL2 |  |
|  |  |  | UGCG | METTL2A |  |
|  |  |  | C5AR1 | FDXR |  |
|  |  |  | CARD8 | ACBD3 |  |
|  |  |  | C12orf76 | IBA57 |  |
|  |  |  | AUTS2 | FKBP14 |  |
|  |  |  | DHTKD1 | NUDCD3 |  |
|  |  |  | MED21 | POF1B |  |
|  |  |  | CCNT1 | CAPN6 |  |
|  |  |  | CDC73 | SOWAHD |  |
|  |  |  | RASSF5 | PRDM11 |  |
|  |  |  | PRPF38A | FAM180B |  |
|  |  |  | TCTEX1D1 | DNAJB5 |  |
|  |  |  | PKHD1 | SUFU |  |
|  |  |  | HMGCLL1 | SORCS3 |  |
|  |  |  | IRAK1BP1 | VTI1A |  |
|  |  |  | GJB7 | B3GALT6 |  |
|  |  |  | FAXC | DFFA |  |
|  |  |  | SLN | FAM105A |  |
|  |  |  | SIK2 | ERP44 |  |
|  |  |  | PLXNA4 | SMC2 |  |
|  |  |  | TTC26 | ZNF483 |  |
|  |  |  | SHH | GATD1 |  |
|  |  |  | CDR1 | PARVA |  |
|  |  |  | MFSD6 | VSIG10 |  |
|  |  |  | ZNF605 | GLT1D1 |  |
|  |  |  | B3GLCT | GTF2IRD2 |  |
|  |  |  | N4BP2L1 | GTF2IRD2B |  |
|  |  |  | SERTM1 | PDK4 |  |
|  |  |  | LLPH | TECPR1 |  |
|  |  |  | CCDC59 | RASGEF1A |  |
|  |  |  | ELK3 | BCLAF3 |  |
|  |  |  | KYAT3 | AEBP2 |  |
|  |  |  | S1PR1 | METTL7A |  |
|  |  |  | ETS2 | C1orf21 |  |
|  |  |  | ACAD8 | CDC73 |  |
|  |  |  | ERBB3 | LRRN2 |  |
|  |  |  | ANKRD52 | CYP4A11 |  |
|  |  |  | ALDH5A1 | CYP4X1 |  |
|  |  |  | SFPQ | DST |  |
|  |  |  | GPR180 | POU3F2 |  |
|  |  |  | GPATCH8 | STAR |  |
|  |  |  | MEIOC | TENM4 |  |
|  |  |  | HOXB6 | CHRNB2 |  |
|  |  |  | SYNE3 | FCRL2 |  |
|  |  |  | TECPR2 | EIF4EBP2 |  |
|  |  |  | BSN | PALD1 |  |
|  |  |  | ARHGAP5 | SLC35B4 |  |
|  |  |  | HIST2H2BF | FIGN |  |
|  |  |  | GABRA4 | CSRNP3 |  |
|  |  |  | TMPRSS11B | SESTD1 |  |
|  |  |  | ERMP1 | FREM2 |  |
|  |  |  | ACER2 | SLC25A15 |  |
|  |  |  | MOB3B | IRGQ |  |
|  |  |  | PEAK1 | ZNF229 |  |
|  |  |  | NOTO | SLC35E3 |  |
|  |  |  | IL18R1 | BEST3 |  |
|  |  |  | ZBTB46 | CXCL14 |  |
|  |  |  | NEXMIF | LMO4 |  |
|  |  |  | ZKSCAN2 | DBT |  |
|  |  |  | HS3ST4 | ZW10 |  |
|  |  |  | MIER3 | GALNT6 |  |
|  |  |  | RAB3C | ZC3H10 |  |
|  |  |  | TWIST2 | ANKRD52 |  |
|  |  |  | SNED1 | RDH16 |  |
|  |  |  | PI4K2B | CTDSP2 |  |
|  |  |  | RBM47 | RANBP9 |  |
|  |  |  | SH2D4A | RIOK2 |  |
|  |  |  | ADAM28 | RND2 |  |
|  |  |  | CLEC16A | GPATCH8 |  |
|  |  |  | XYLT1 | PNPO |  |
|  |  |  | EEF2K | NXPH3 |  |
|  |  |  | HEY1 | MRPL27 |  |
|  |  |  | TAT | PDAP1 |  |
|  |  |  | GCSH | PPP1R3A |  |
|  |  |  | ATF6 | CALU |  |
|  |  |  | CABP7 | TMX3 |  |
|  |  |  | DRG1 | GADD45B |  |
|  |  |  | CRNKL1 | EIF5 |  |
|  |  |  | SCLT1 | GABRB3 |  |
|  |  |  | ELF2 | NLK |  |
|  |  |  | SNX15 | SLC38A3 |  |
|  |  |  | CABP4 | NUBPL |  |
|  |  |  | SMCHD1 | FAM212B |  |
|  |  |  | TMEM184A | TBX15 |  |
|  |  |  | TP53BP1 | OTUD7B |  |
|  |  |  | C2CD4A | SLAIN2 |  |
|  |  |  | MAATS1 | FAM47E-STBD1 |  |
|  |  |  | RABL3 | SCD5 |  |
|  |  |  | PNPT1 | PYCR3 |  |
|  |  |  | VPS54 | CNTLN |  |
|  |  |  | MEIS1 | ACER2 |  |
|  |  |  | APLF | SMU1 |  |
|  |  |  | PIAS2 | ELAVL1 |  |
|  |  |  | KBTBD8 | TSR1 |  |
|  |  |  | HTR1F | RAP1GAP2 |  |
|  |  |  | NFKBIZ | FAHD2A |  |
|  |  |  | RGPD5 | TSPEAR |  |
|  |  |  | RGPD8 | LRRC74B |  |
|  |  |  | ACTR3 | SNRPD3 |  |
|  |  |  | WDR33 | SLC16A2 |  |
|  |  |  | RPL7L1 | SEPHS2 |  |
|  |  |  | KCNB1 | STX1B |  |
|  |  |  | UBE2V1 | ITGAX |  |
|  |  |  | WNT8B | SREK1IP1 |  |
|  |  |  | MS4A6A | MAP1B |  |
|  |  |  | DAGLA | FBXO42 |  |
|  |  |  | NLGN1 | IFFO2 |  |
|  |  |  | USP13 | TRNAU1AP |  |
|  |  |  | MAP3K13 | CDK5R1 |  |
|  |  |  | FBXO45 | CCDC25 |  |
|  |  |  |  | RFLNB |  |
|  |  |  |  | USP31 |  |
|  |  |  |  | PRKCB |  |
|  |  |  |  | CHMP4C |  |
|  |  |  |  | KCNJ10 |  |
|  |  |  |  | FMO2 |  |
|  |  |  |  | DRG1 |  |
|  |  |  |  | SYNGR1 |  |
|  |  |  |  | ABHD12 |  |
|  |  |  |  | GINS1 |  |
|  |  |  |  | RNASEH2C |  |
|  |  |  |  | RAB12 |  |
|  |  |  |  | CCDC170 |  |
|  |  |  |  | IRAK2 |  |
|  |  |  |  | SUSD5 |  |
|  |  |  |  | ANKRD63 |  |
|  |  |  |  | SHC4 |  |
|  |  |  |  | PCYOX1 |  |
|  |  |  |  | HEY2 |  |
|  |  |  |  | PERP |  |
|  |  |  |  | ROCK1 |  |
|  |  |  |  | PSTPIP2 |  |
|  |  |  |  | SMAD4 |  |
|  |  |  |  | GLYCTK |  |
|  |  |  |  | GPR107 |  |
|  |  |  |  | SPACA9 |  |
|  |  |  |  | FBLN7 |  |
|  |  |  |  | SRSF3 |  |
|  |  |  |  | GLP1R |  |
|  |  |  |  | JPH2 |  |
|  |  |  |  | ZNF335 |  |
|  |  |  |  | LDB3 |  |
|  |  |  |  | IFIT5 |  |
|  |  |  |  | GOLGA7B |  |
|  |  |  |  | SEC31B |  |
|  |  |  |  | TMEM41A |  |
|  |  |  |  | LPP |  |
|  |  |  |  | XXYLT1 |  |

**Supplemental Table 5.** The overlapping target genes among 6 miRNAs (miR-132-3p, miR-138-5p, miR-141-3p, miR-145-5p, miR-150-5p, and miR-22-3p):

| **Genes** | **Amount of miRNA** | **miR-132** | **miR-138** | **miR-141** | **miR-145** | **miR-150** | **miR-22** |
| --- | --- | --- | --- | --- | --- | --- | --- |
| ZBTB20 | 5 | ● | ● | ● | ● | ● |  |
| ZNF704 | 5 | ● | ● | ● | ● |  | ● |
| EIF4EBP2 | 5 |  | ● | ● | ● | ● | ● |
| CALU | 4 | ● |  | ● | ● | ● |  |
| PPM1L | 4 | ● | ● | ● |  |  | ● |
| SRSF10 | 4 | ● |  | ● | ● |  | ● |
| MAP3K3 | 4 | ● |  | ● | ● | ● |  |
| NFIA | 4 | ● | ● | ● | ● |  |  |
| PSD3 | 4 | ● | ● |  | ● | ● |  |
| TMEM178B | 4 | ● | ● |  |  | ● | ● |
| MECP2 | 4 | ● | ● |  |  | ● | ● |
| ARRB1 | 4 |  | ● | ● |  | ● | ● |
| ST3GAL6 | 4 |  | ● | ● | ● | ● |  |
| CSRNP3 | 4 |  | ● |  | ● | ● | ● |
| CREB1 | 4 |  | ● | ● |  | ● | ● |
| VEZF1 | 4 |  | ● |  | ● | ● | ● |
| CDK6 | 4 |  | ● | ● | ● |  | ● |
| LPP | 4 |  | ● | ● |  | ● | ● |
| PSEN1 | 4 |  |  | ● | ● | ● | ● |
| KCNMA1 | 3 | ● | ● |  | ● |  |  |
| NFIB | 3 | ● | ● |  | ● |  |  |
| ELAVL4 | 3 | ● |  | ● | ● |  |  |
| STX16 | 3 | ● |  | ● | ● |  |  |
| SLC8A1 | 3 | ● |  |  | ● | ● |  |
| SOX5 | 3 | ● | ● | ● |  |  |  |
| SAP30L | 3 | ● |  |  | ● | ● |  |
| GAB1 | 3 | ● |  | ● |  | ● |  |
| KCNN3 | 3 | ● |  |  | ● | ● |  |
| QKI | 3 | ● |  | ● | ● |  |  |
| CREB5 | 3 | ● |  |  | ● | ● |  |
| RNF24 | 3 | ● | ● |  | ● |  |  |
| SMAD2 | 3 | ● |  | ● | ● |  |  |
| GPATCH2L | 3 | ● | ● |  |  | ● |  |
| SSH2 | 3 | ● | ● |  | ● |  |  |
| SESN3 | 3 | ● | ● |  | ● |  |  |
| TET2 | 3 | ● |  |  | ● |  | ● |
| MTF1 | 3 | ● |  |  | ● |  | ● |
| NTNG1 | 3 | ● |  | ● | ● |  |  |
| VAPA | 3 | ● |  |  | ● | ● |  |
| PLAGL2 | 3 | ● |  | ● | ● |  |  |
| CACUL1 | 3 | ● | ● |  |  |  | ● |
| TEAD1 | 3 | ● | ● |  |  | ● |  |
| POM121 | 3 | ● | ● |  |  | ● |  |
| GPRIN3 | 3 | ● | ● |  |  | ● |  |
| HMGA2 | 3 | ● |  |  | ● | ● |  |
| FRS2 | 3 | ● |  | ● | ● |  |  |
| C16orf87 | 3 | ● | ● | ● |  |  |  |
| EPB41L5 | 3 | ● |  |  | ● | ● |  |
| SH3TC2 | 3 | ● |  | ● |  | ● |  |
| SOX11 | 3 | ● |  | ● | ● |  |  |
| YWHAG | 3 | ● |  | ● | ● |  |  |
| SNIP1 | 3 | ● | ● | ● |  |  |  |
| DYNLL2 | 3 | ● |  | ● |  | ● |  |
| ZNF451 | 3 | ● |  |  | ● |  | ● |
| SLAIN2 | 3 | ● |  | ● |  | ● |  |
| PAPD5 | 3 | ● |  |  | ● | ● |  |
| PCGF3 | 3 | ● | ● | ● |  |  |  |
| TTC14 | 3 | ● | ● |  | ● |  |  |
| TMOD2 | 3 |  | ● |  | ● | ● |  |
| UNC5D | 3 |  | ● |  | ● |  | ● |
| CBX5 | 3 |  | ● | ● |  | ● |  |
| IGF2BP1 | 3 |  | ● |  |  | ● | ● |
| TRIM13 | 3 |  | ● |  | ● |  | ● |
| PEG10 | 3 |  | ● |  | ● | ● |  |
| CLOCK | 3 |  | ● | ● | ● |  |  |
| USP49 | 3 |  | ● | ● |  | ● |  |
| KIAA1958 | 3 |  | ● | ● | ● |  |  |
| XPR1 | 3 |  | ● | ● |  | ● |  |
| DTX4 | 3 |  | ● |  | ● | ● |  |
| GK5 | 3 |  | ● |  | ● | ● |  |
| MBTD1 | 3 |  | ● |  | ● | ● |  |
| AKT3 | 3 |  | ● |  | ● | ● |  |
| VPS53 | 3 |  | ● |  |  | ● | ● |
| GATAD2B | 3 |  | ● |  |  | ● | ● |
| E2F3 | 3 |  | ● | ● |  | ● |  |
| VANGL1 | 3 |  | ● | ● | ● |  |  |
| APPBP2 | 3 |  | ● | ● | ● |  |  |
| RASSF8 | 3 |  | ● | ● |  | ● |  |
| PDAP1 | 3 |  | ● |  |  | ● | ● |
| FOSL2 | 3 |  | ● |  |  | ● | ● |
| BTG1 | 3 |  | ● |  | ● |  | ● |
| BCL9L | 3 |  | ● |  |  | ● | ● |
| FAHD2A | 3 |  | ● |  | ● | ● |  |
| FOXP1 | 3 |  |  | ● |  | ● | ● |
| EIF4E | 3 |  |  | ● | ● | ● |  |
| KIF3A | 3 |  |  | ● | ● | ● |  |
| TGFBR1 | 3 |  |  | ● | ● | ● |  |
| EXOC5 | 3 |  |  | ● | ● |  | ● |
| SFXN1 | 3 |  |  | ● | ● |  | ● |
| POGK | 3 |  |  | ● | ● |  | ● |
| WWC2 | 3 |  |  | ● |  | ● | ● |
| WDR33 | 3 |  |  | ● | ● |  | ● |
| CBL | 3 |  |  | ● |  | ● | ● |
| PAFAH1B2 | 3 |  |  | ● | ● |  | ● |
| EIF5 | 3 |  |  | ● | ● | ● |  |
| TPM3 | 3 |  |  |  | ● | ● | ● |
| ACVR1B | 3 |  |  |  | ● | ● | ● |
| NTRK2 | 3 |  |  |  | ● | ● | ● |
| MMP14 | 3 |  |  |  | ● | ● | ● |
| TMOD3 | 3 |  |  |  | ● | ● | ● |
| SMAD4 | 3 |  |  |  | ● | ● | ● |
| PRPF38A | 3 |  |  |  | ● | ● | ● |
| ANKRD52 | 3 |  |  |  | ● | ● | ● |

**Supplemental Table 6. T**he three most interacting potential targets, so called “hub genes” and six miRNAs. Target genes indicated in Bold are validated by strong experimental methods (report/western blot/qRT-PCR):

| **miR-22** | **miR-150** | **miR-145** | **miR-141** | **miR-138** | **miR-132** |
| --- | --- | --- | --- | --- | --- |
| target top 3 gene | target top 3 gene | target top 3 gene | target top 3 gene | target top 3 gene | target top 3 gene |
| TP53 | BTRC | **MYC** | EGFR | **CCND1** | BTRC |
| **SIRT1** | VHL | **VEGFA** | CBL | CREB1 | TCEB1 |
| **ESR1** | KLHL21 | **SMAD2** | ARRB1 | **HIF1A** | **KLHL11** |
|  |  |  |  |  |  |
| **Common durgs** | **Common durgs** | **Common durgs** | **Common durgs** | **Common durgs** | **Common durgs** |
| RESVERATROL | NA | MYC and "VEGFA": | EGFR and "CBL": | NA | NA |
|  |  | CISPLATIN | ERLOTINIB |  |  |
|  |  |  | GEMCITABINE |  |  |
|  |  |  | DASATINIB |  |  |
